# Supplementary material for: Tectonic and climatic implications of the Aleutian Arc initiation ≥56 million years ago
Source: Nat Commun. 2026 May 30;17:4865. doi: 10.1038/s41467-026-73363-y (PMC13226694; doi:10.1038/s41467-026-73363-y)
Supplement: Supplementary file 1 — Supplementary Information [file 41467_2026_73363_MOESM1_ESM.pdf]

Supplementary Information for

Aleutian Arc Initiation at 56 Ma: Tectonic and climatic implications

K. Hoernle, corresponding author, B. Jicha, M. Portnyagin, S. Zahirovic, D. Müller, F. Hauff, C. Timm, T. W. Höfig, G. Yogodzinski, M. Guillong, C. Berndt, D. Savelyev, R. Bezard and B. Baranov

Corresponding author: khoernle@geomar.de

The PDF file includes:
Fig. 1 to 3
Tables 1 to 5

Supplemantary Fig. 1. U/Pb age plots. a) U/Pb Concordia diagrams for dated samples on the left side and weighted age means on the right side.

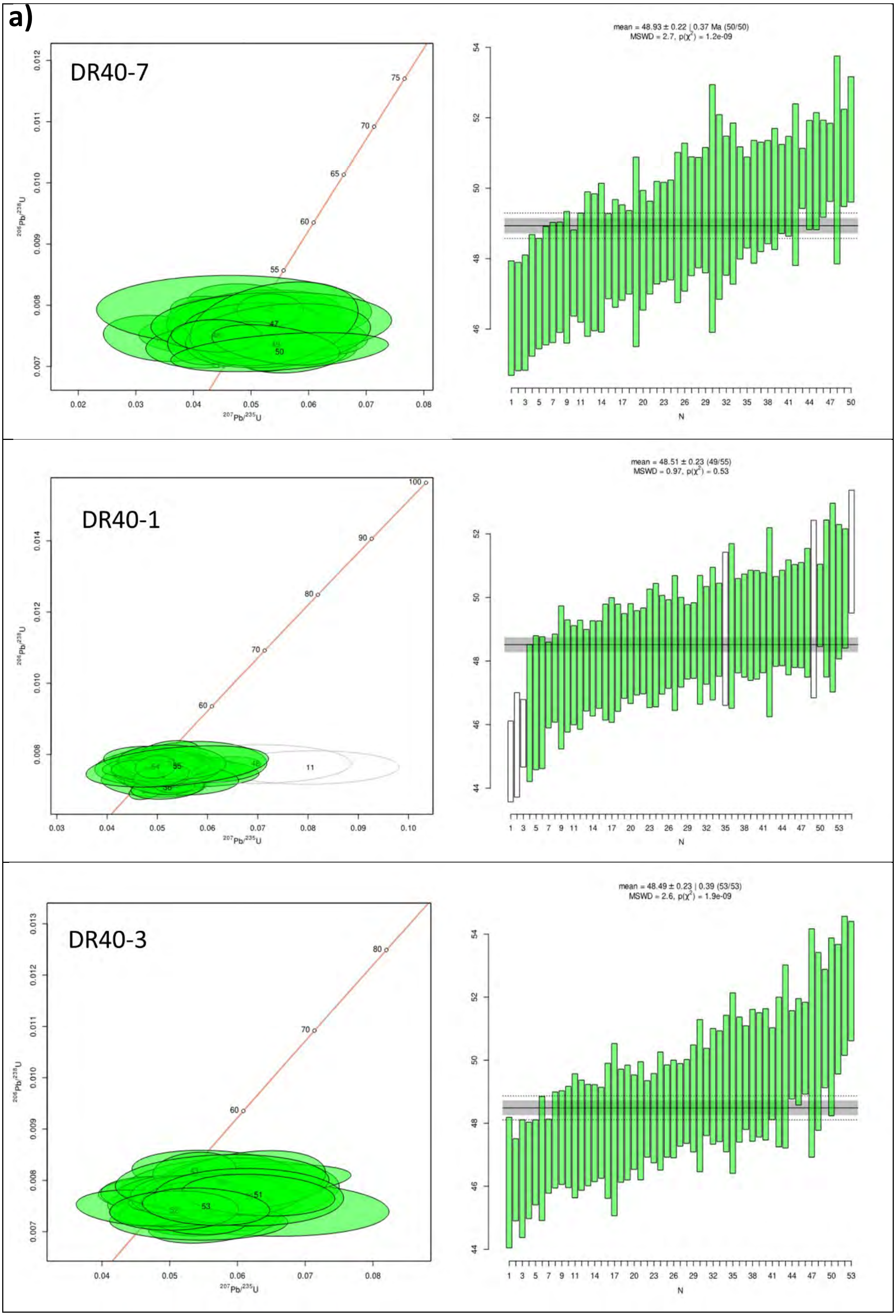

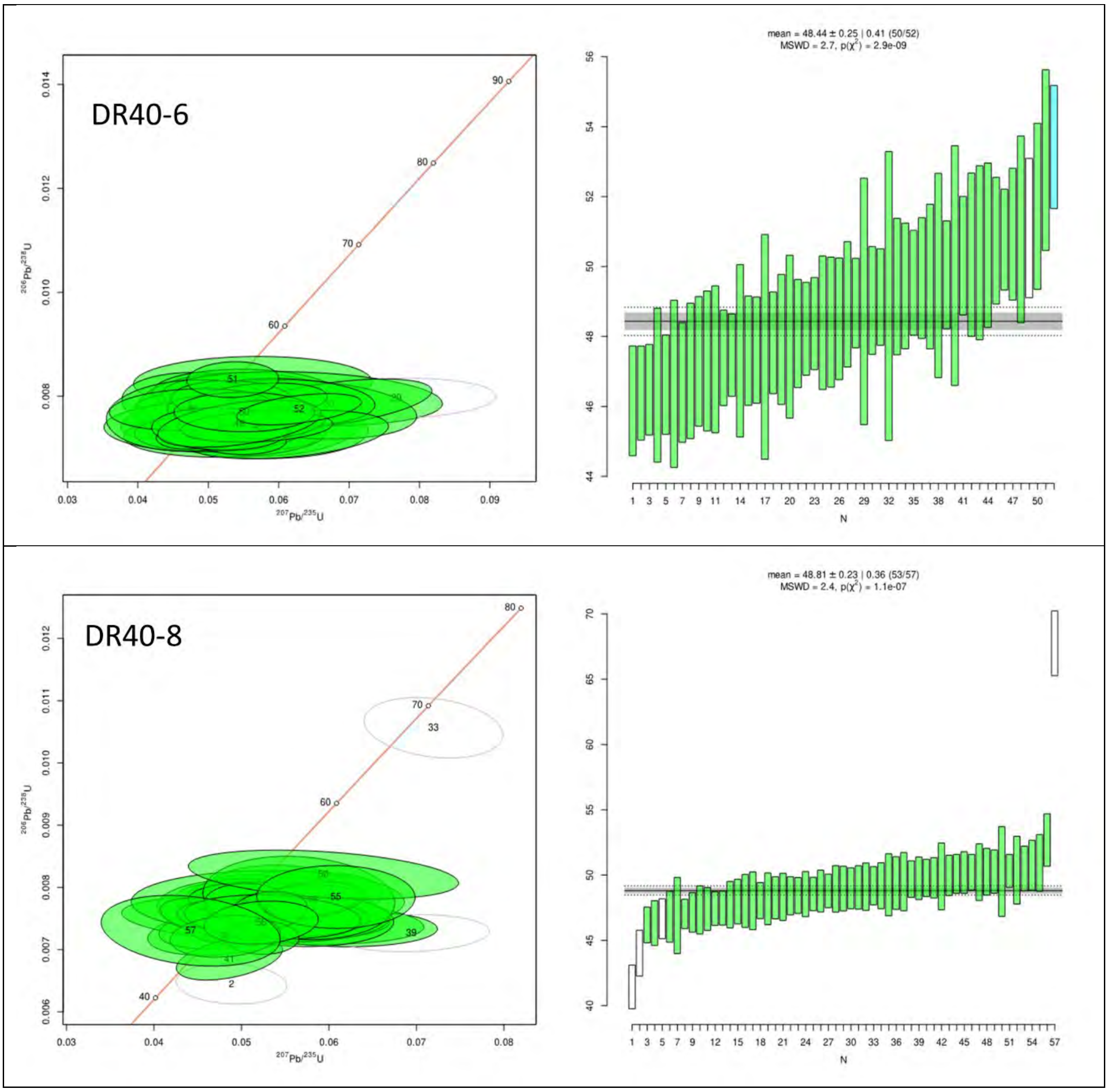
DR40-6
mean = 48.44 ± 0.25 | 0.41 (50/52)
MSWD = 2.7, p(χ²) = 2.9e-09
²⁰⁶Pb/²³⁸U
²⁰⁷Pb/²³⁵U
N
DR40-8
mean = 48.81 ± 0.23 | 0.36 (53/57)
MSWD = 2.4, p(χ²) = 1.1e-07
²⁰⁶Pb/²³⁸U
²⁰⁷Pb/²³⁵U
N

Supplemantary Fig. 1b. Reference Zircons analyzed with zircon samples.

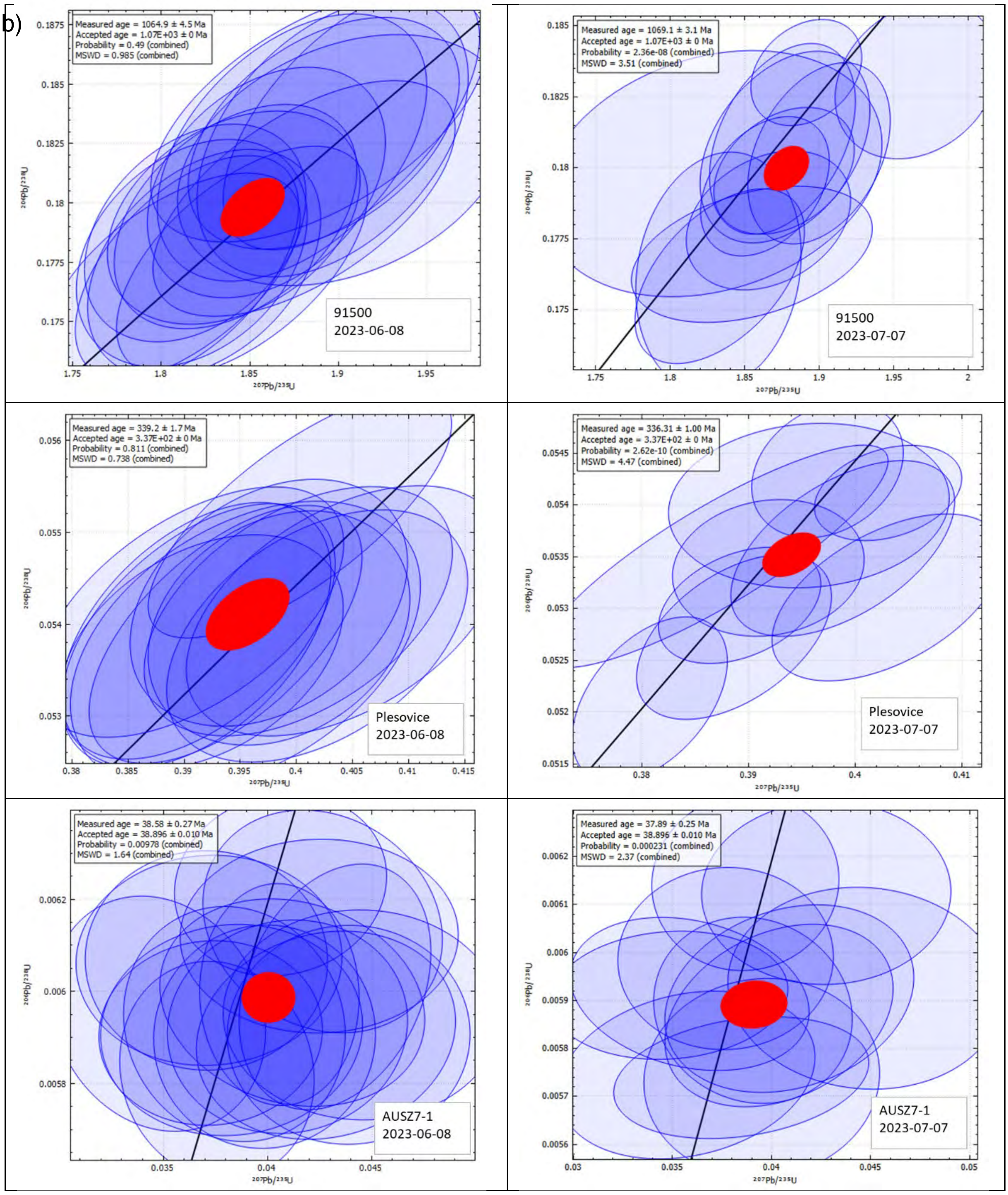

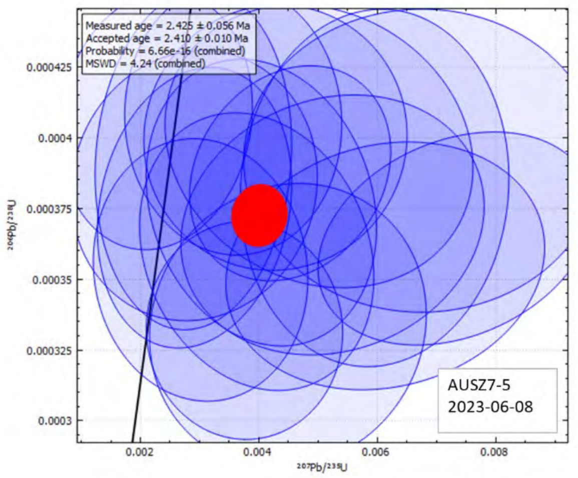
Measured age = 2.425 ± 0.056 Ma
Accepted age = 2.410 ± 0.010 Ma
Probability = 6.66e-16 (combined)
MSWD = 4.24 (combined)
0.000425
0.0004
0.000375
0.00035
0.000325
0.0003
206Pb/238U
0.002
0.004
0.006
0.008
207Pb/235U
AUSZ7-5
2023-06-08

Supplementary Fig. 2. Ar-Ar age data. Ar-Ar age spectrum plots for each dated sample containing apparent age (in Ma), %40Ar and K/Ca ratio plots for each measurement step. Below each group of stepwise heating plots are the isochron diagrams for the same sample analyses.

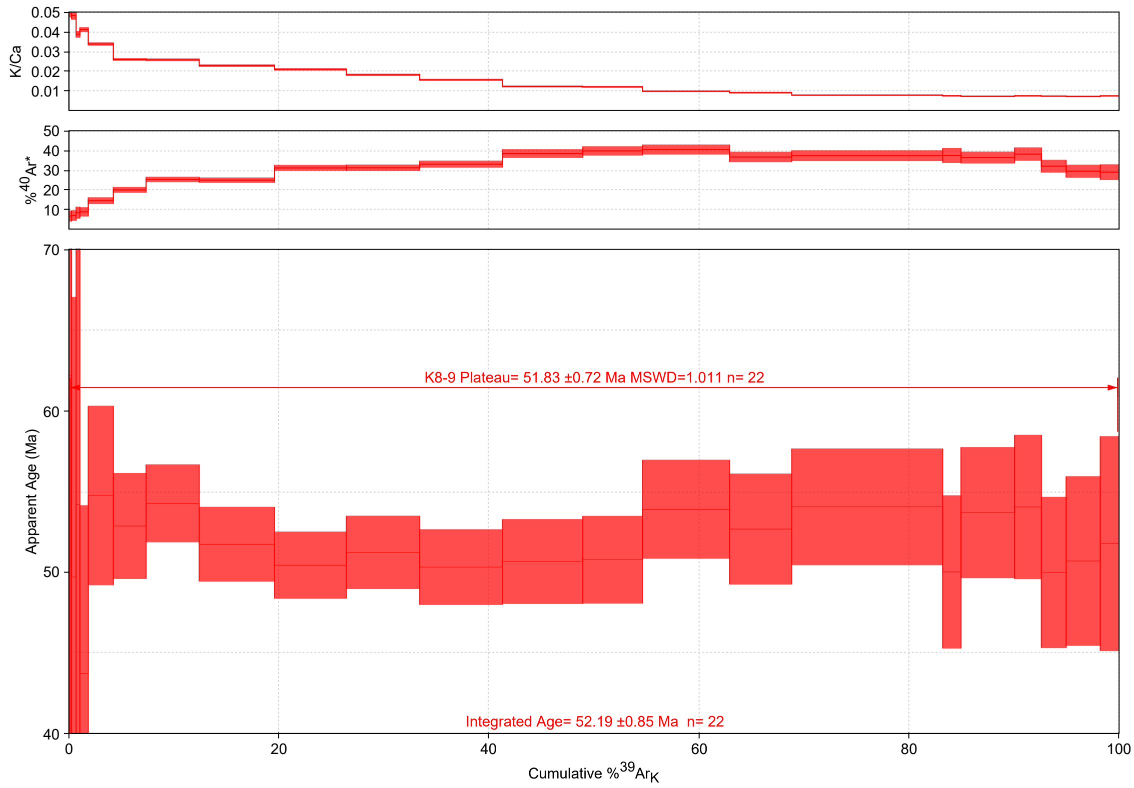

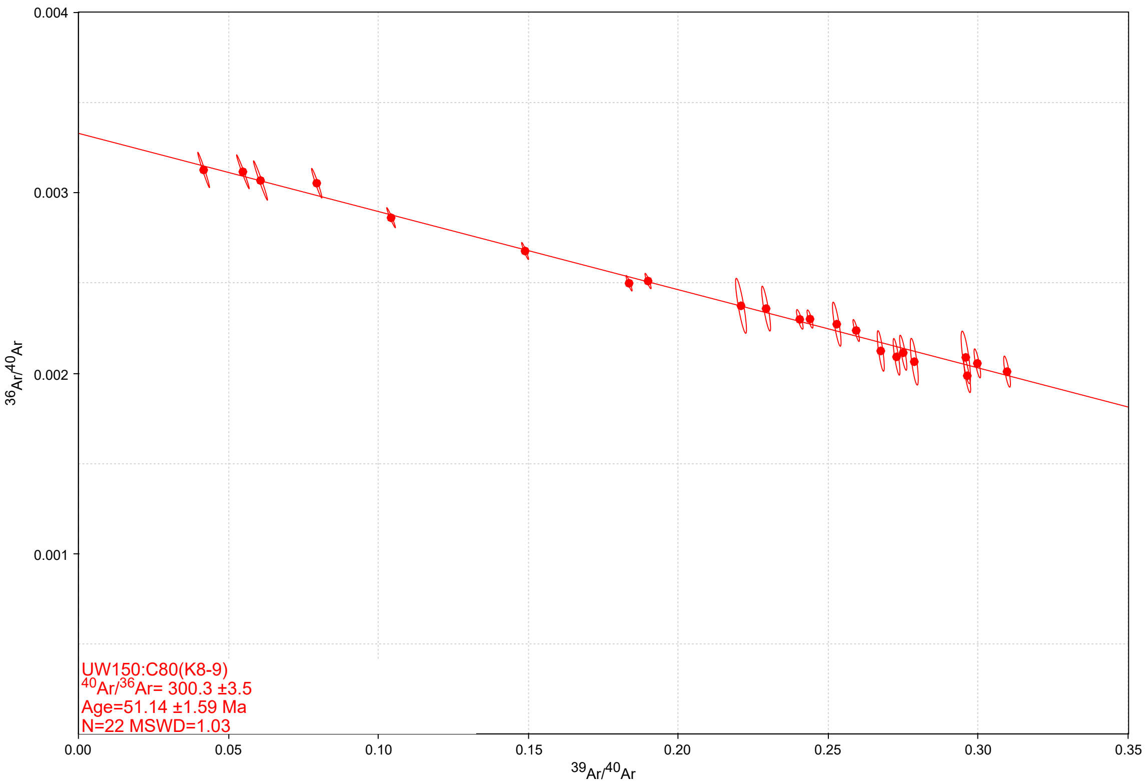

UW150:C80(K8-9)
$^{40}Ar/^{36}Ar$= 300.3 ±3.5
Age=51.14 ±1.59 Ma
N=22 MSWD=1.03
$^{36}Ar/^{40}Ar$
$^{39}Ar/^{40}Ar$
0.004
0.003
0.002
0.001
0.00
0.05
0.10
0.15
0.20
0.25
0.30
0.35

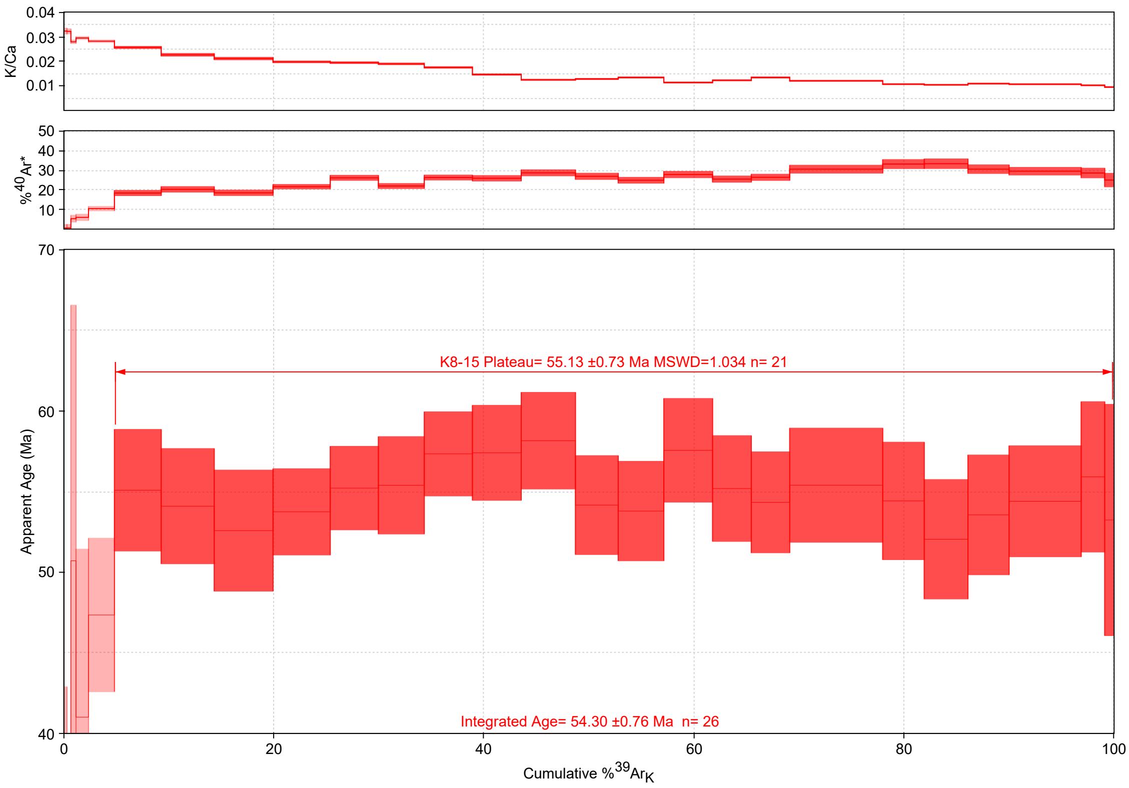

K/Ca
%40Ar*
Apparent Age (Ma)
K8-15 Plateau= 55.13 ±0.73 Ma MSWD=1.034 n= 21
Integrated Age= 54.30 ±0.76 Ma n= 26
Cumulative %39ArK

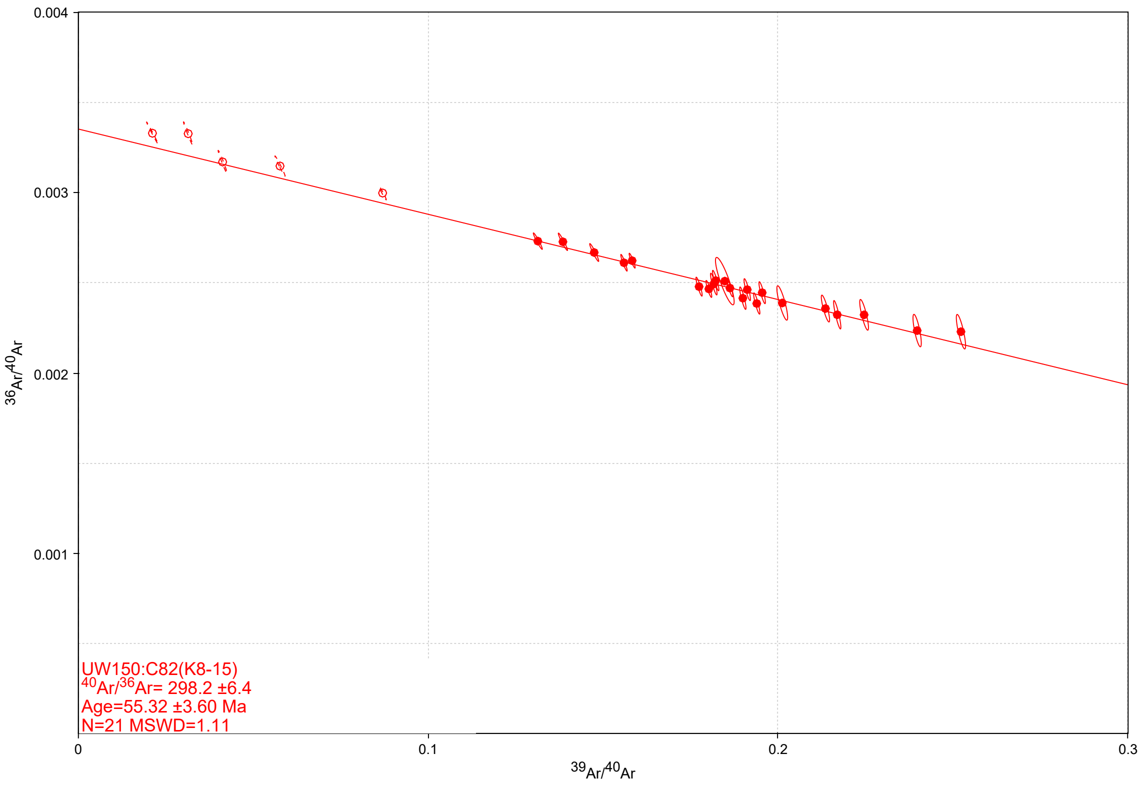

0.004
0.003
0.002
0.001
$^{36}Ar/^{40}Ar$
0
0.1
0.2
0.3
$^{39}Ar/^{40}Ar$
UW150:C82(K8-15)
$^{40}Ar/^{36}Ar$= 298.2 ±6.4
Age=55.32 ±3.60 Ma
N=21 MSWD=1.11

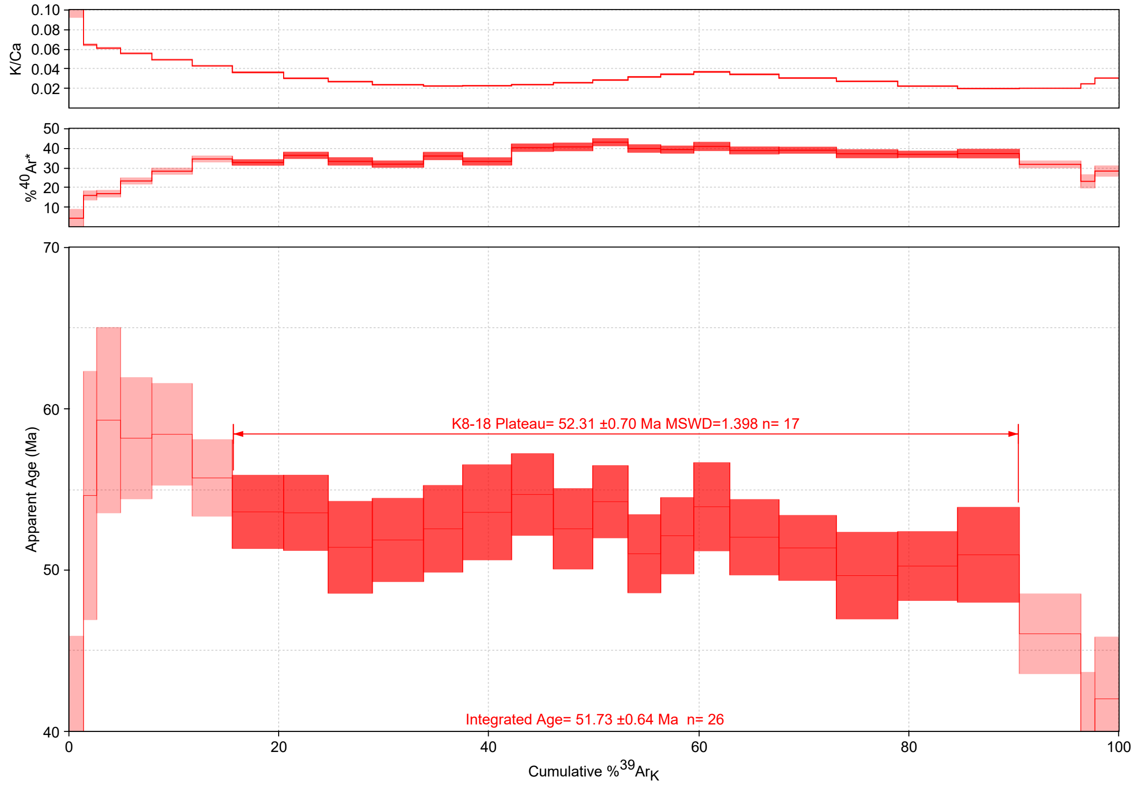

K/Ca
0.10
0.08
0.06
0.04
0.02
%^{40}Ar*
50
40
30
20
10
Apparent Age (Ma)
70
60
50
40
K8-18 Plateau= 52.31 ±0.70 Ma MSWD=1.398 n= 17
Integrated Age= 51.73 ±0.64 Ma n= 26
0
20
40
60
80
100
Cumulative %^{39}Ar$_K$

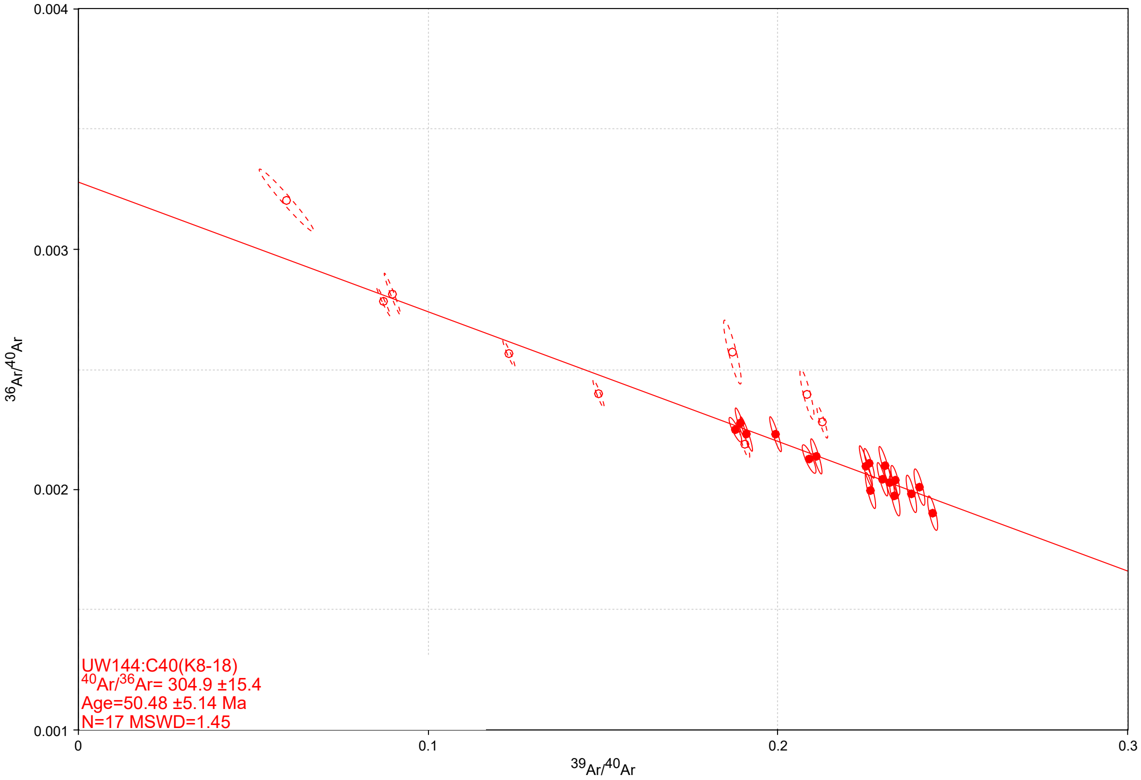

0.004
0.003
0.002
0.001
$^{36}Ar/^{40}Ar$
0
0.1
0.2
0.3
$^{39}Ar/^{40}Ar$
UW144:C40(K8-18)
$^{40}Ar/^{36}Ar$= 304.9 ±15.4
Age=50.48 ±5.14 Ma
N=17 MSWD=1.45

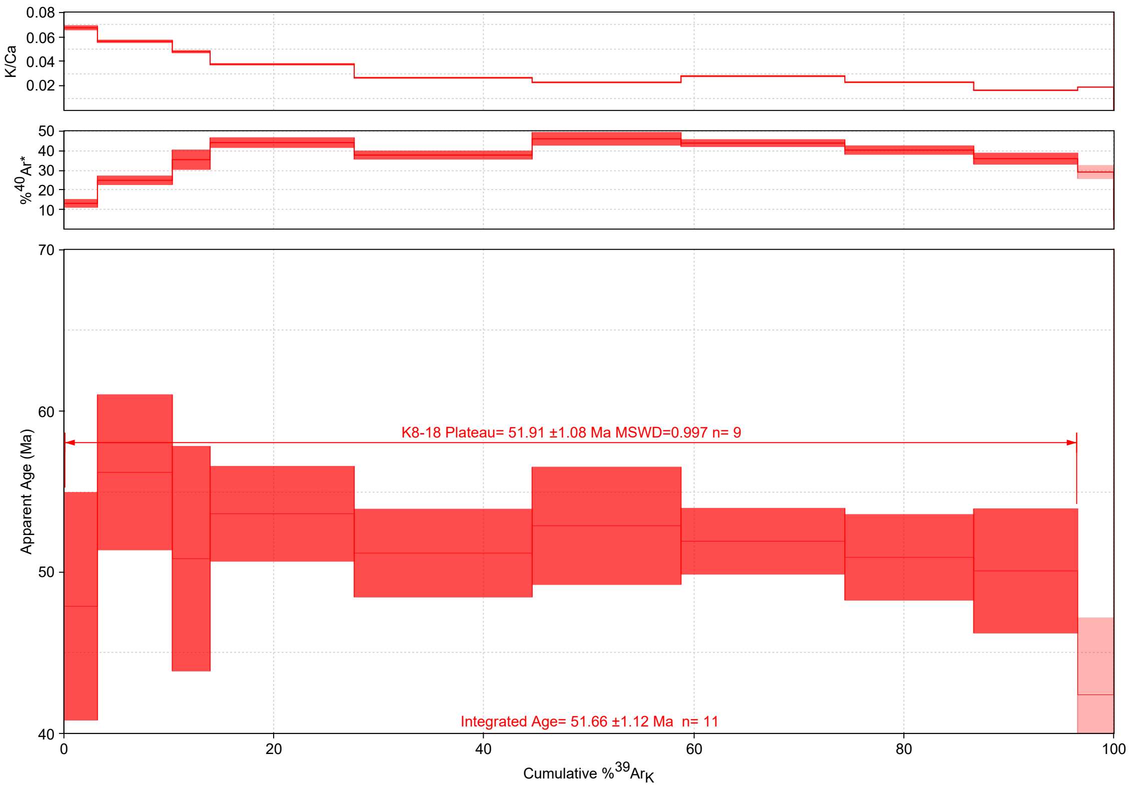

K/Ca
0.08
0.06
0.04
0.02
%^{40}Ar*
50
40
30
20
10
70
60
50
40
Apparent Age (Ma)
K8-18 Plateau= 51.91 ±1.08 Ma MSWD=0.997 n= 9
Integrated Age= 51.66 ±1.12 Ma n= 11
0
20
40
60
80
100
Cumulative %^{39}Ar$_K$

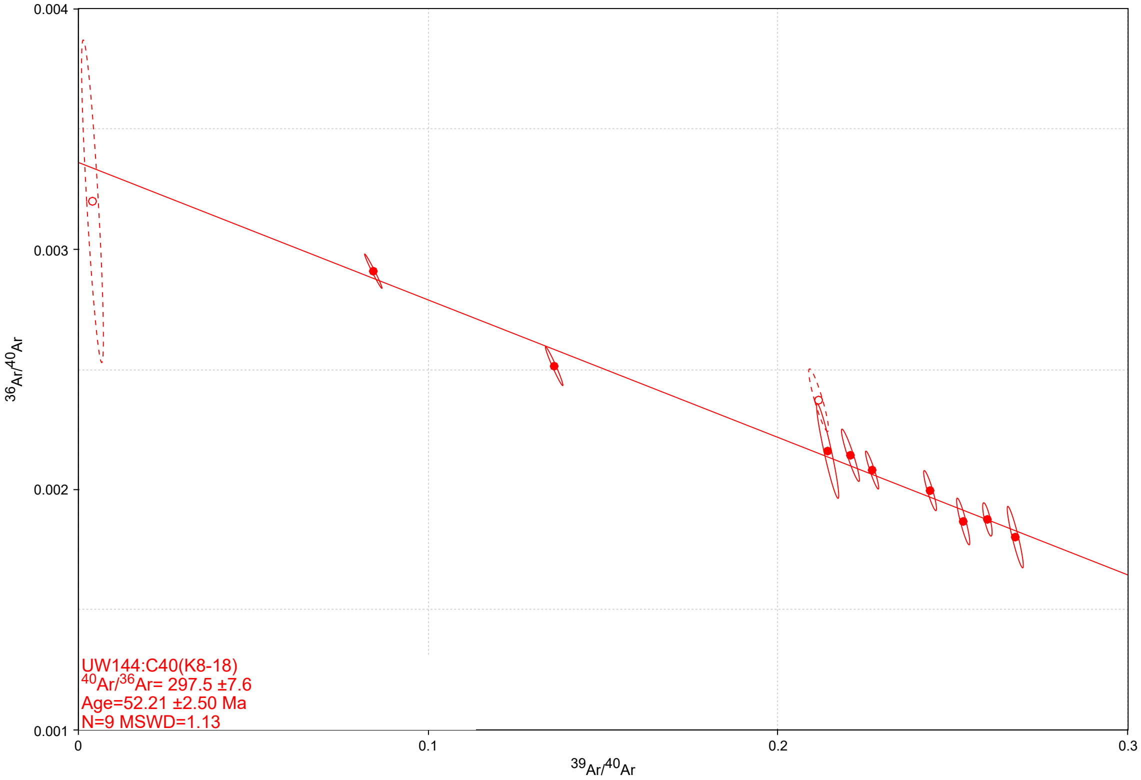

0.004
0.003
0.002
0.001
0
0.1
0.2
0.3
36Ar/40Ar
39Ar/40Ar
UW144:C40(K8-18)
40Ar/36Ar= 297.5 ±7.6
Age=52.21 ±2.50 Ma
N=9 MSWD=1.13

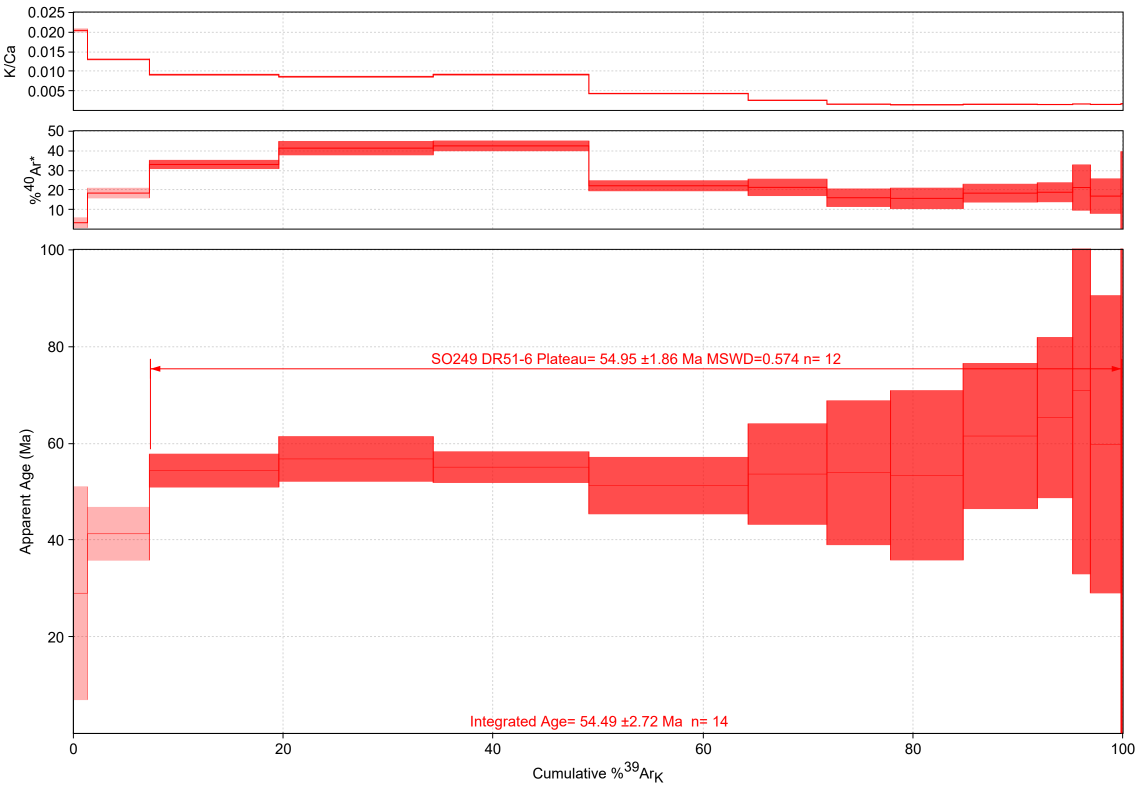

K/Ca
0.025
0.020
0.015
0.010
0.005
%^{40}Ar*
50
40
30
20
10
Apparent Age (Ma)
100
80
60
40
20
SO249 DR51-6 Plateau= 54.95 ±1.86 Ma MSWD=0.574 n= 12
Integrated Age= 54.49 ±2.72 Ma n= 14
0
20
40
60
80
100
Cumulative %^{39}Ar$_K$

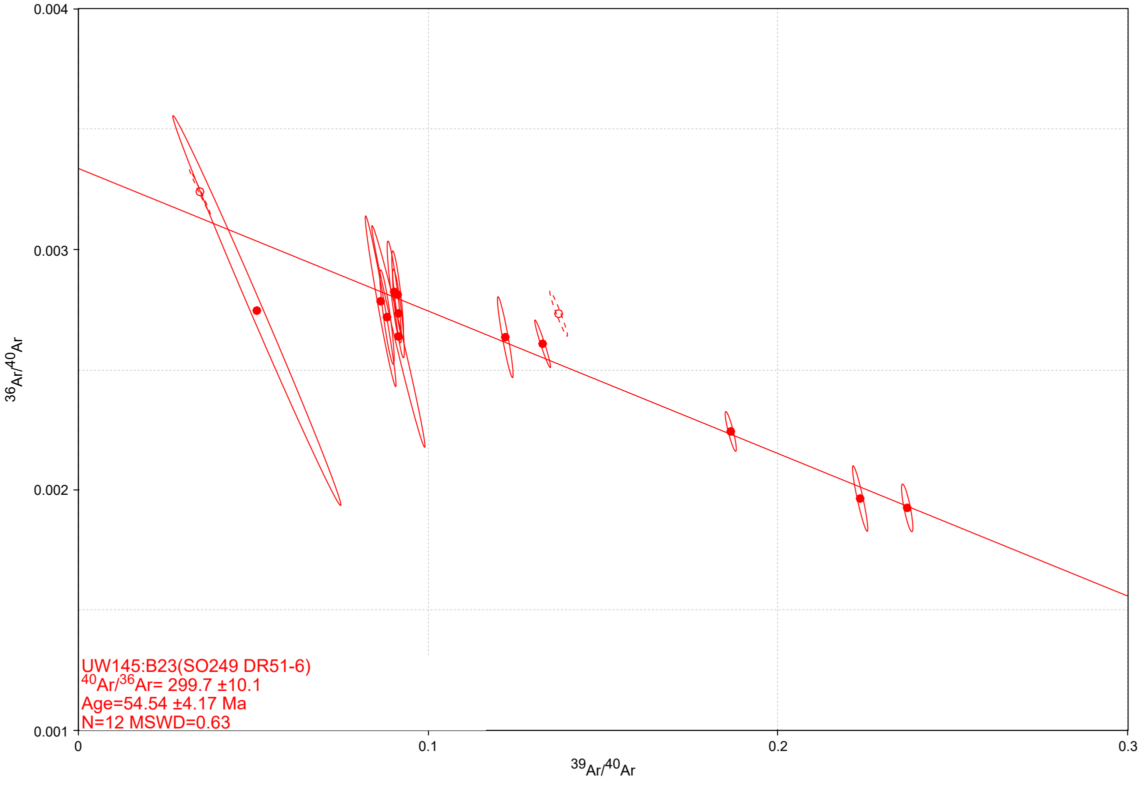
0.004
0.003
0.002
0.001
0
0.1
0.2
0.3
$^{36}Ar/^{40}Ar$
$^{39}Ar/^{40}Ar$
UW145:B23(SO249 DR51-6)
$^{40}Ar/^{36}Ar$= 299.7 ±10.1
Age=54.54 ±4.17 Ma
N=12 MSWD=0.63

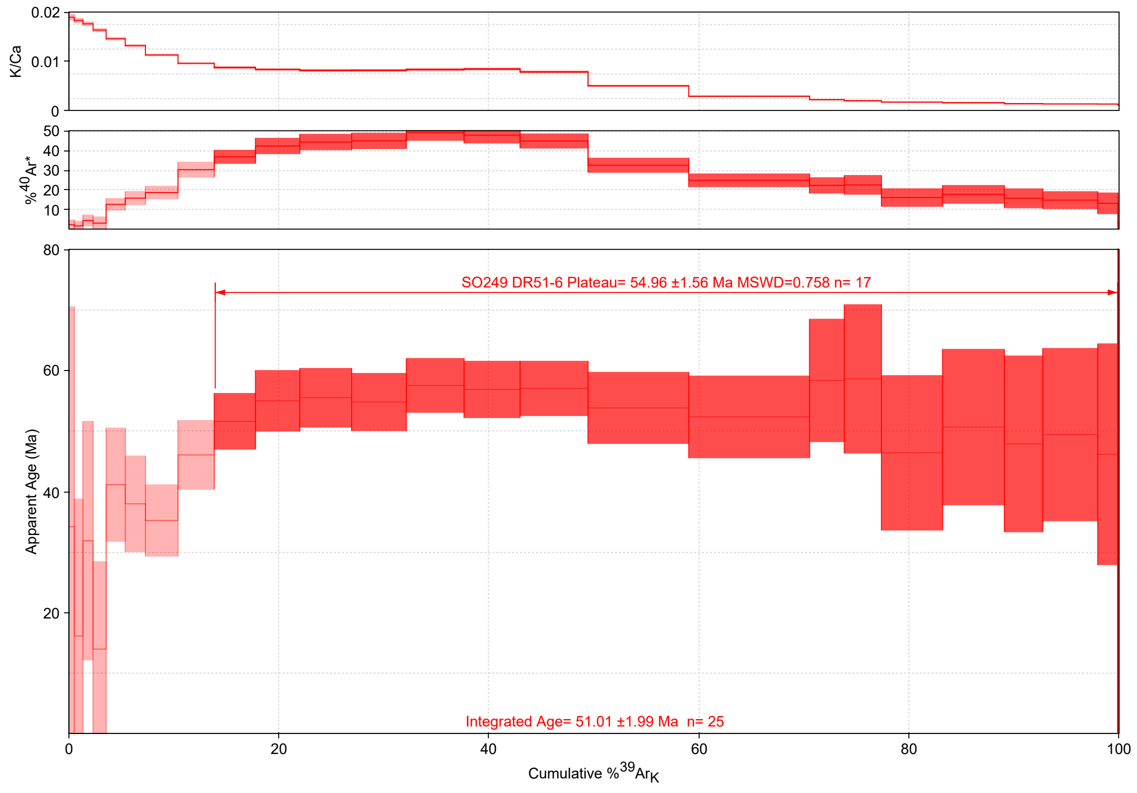
0.02
0.01
0
K/Ca
50
40
30
20
10
%40Ar*
80
60
40
20
Apparent Age (Ma)
SO249 DR51-6 Plateau= 54.96 ±1.56 Ma MSWD=0.758 n= 17
Integrated Age= 51.01 ±1.99 Ma n= 25
0
20
40
60
80
100
Cumulative %39ArK

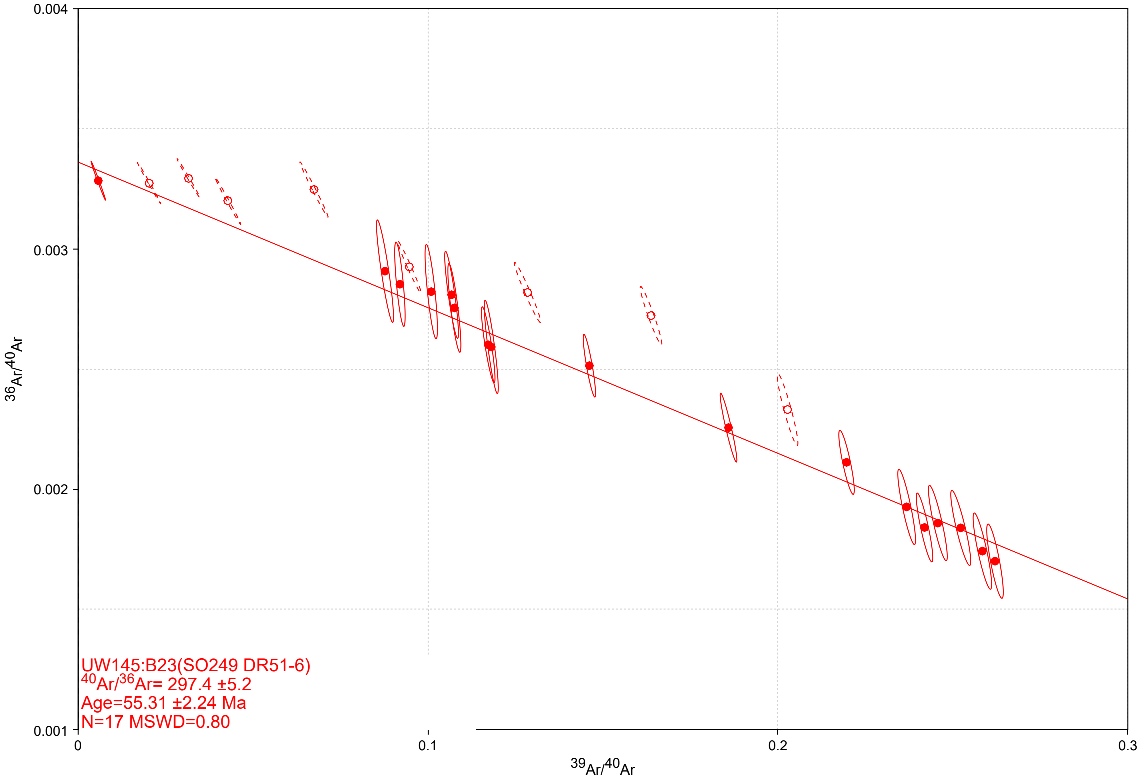

0.004
0.003
0.002
0.001
0
0.1
0.2
0.3
$^{36}Ar/^{40}Ar$
$^{39}Ar/^{40}Ar$
UW145:B23(SO249 DR51-6)
$^{40}Ar/^{36}Ar$= 297.4 ±5.2
Age=55.31 ±2.24 Ma
N=17 MSWD=0.80

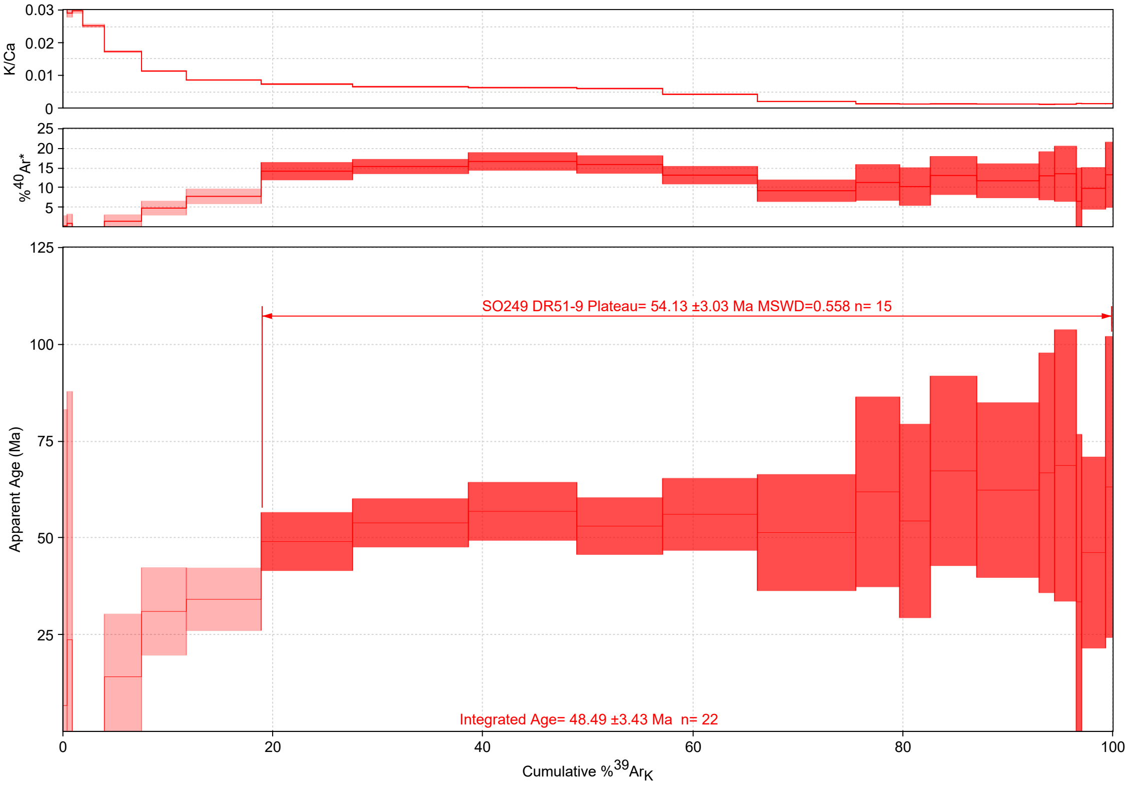

K/Ca
0.03
0.02
0.01
0
%^{40}Ar*
25
20
15
10
5
125
100
75
50
25
Apparent Age (Ma)
SO249 DR51-9 Plateau= 54.13 ±3.03 Ma MSWD=0.558 n= 15
Integrated Age= 48.49 ±3.43 Ma n= 22
0
20
40
60
80
100
Cumulative %^{39}Ar$_K$

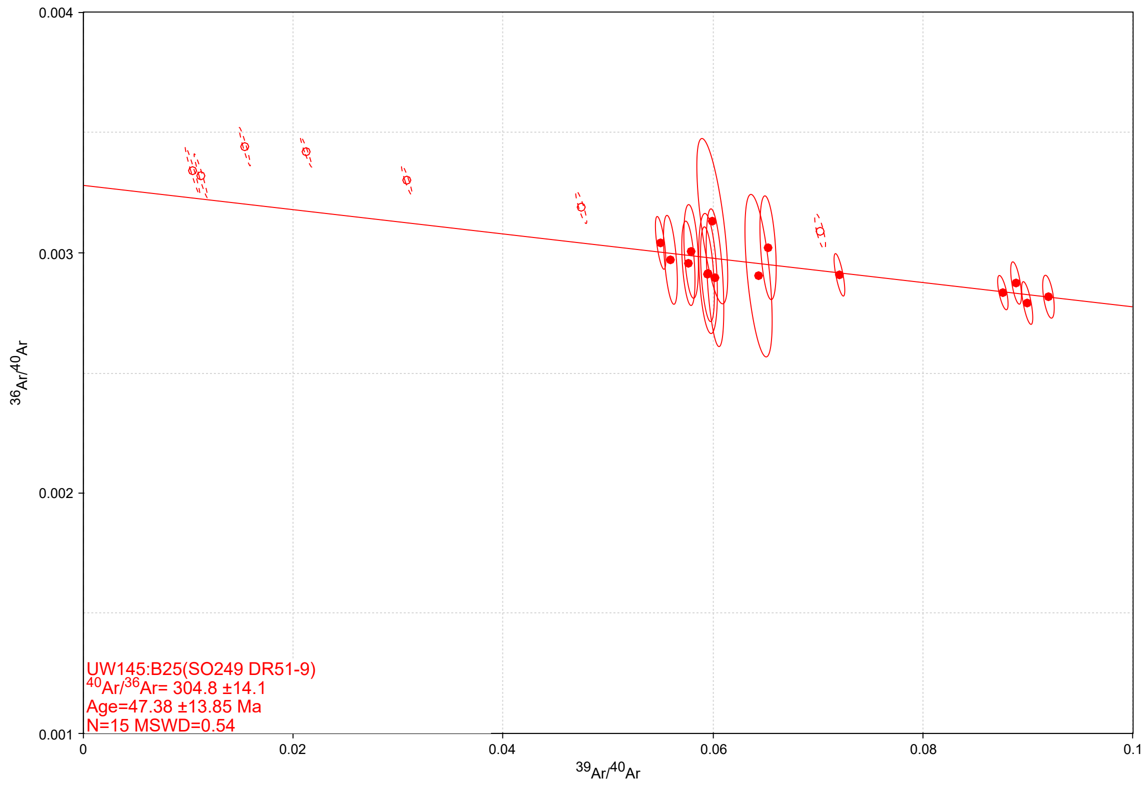

0.004
0.003
0.002
0.001
0
0.02
0.04
0.06
0.08
0.1
$^{36}Ar/^{40}Ar$
$^{39}Ar/^{40}Ar$
UW145:B25(SO249 DR51-9)
$^{40}Ar/^{36}Ar$= 304.8 ±14.1
Age=47.38 ±13.85 Ma
N=15 MSWD=0.54

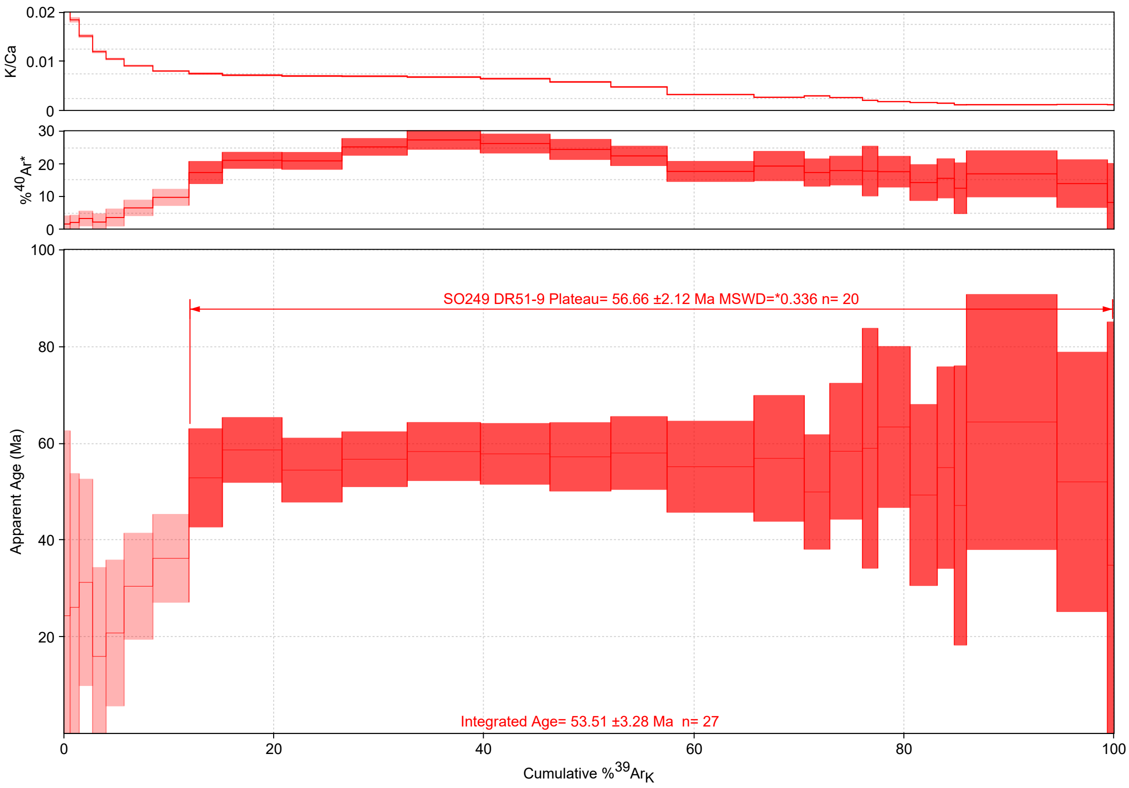

K/Ca
0.02
0.01
0
%^{40}Ar*
30
20
10
0
SO249 DR51-9 Plateau= 56.66 ±2.12 Ma MSWD=*0.336 n= 20
Apparent Age (Ma)
100
80
60
40
20
Integrated Age= 53.51 ±3.28 Ma n= 27
0
20
40
60
80
100
Cumulative %^{39}Ar$_K$

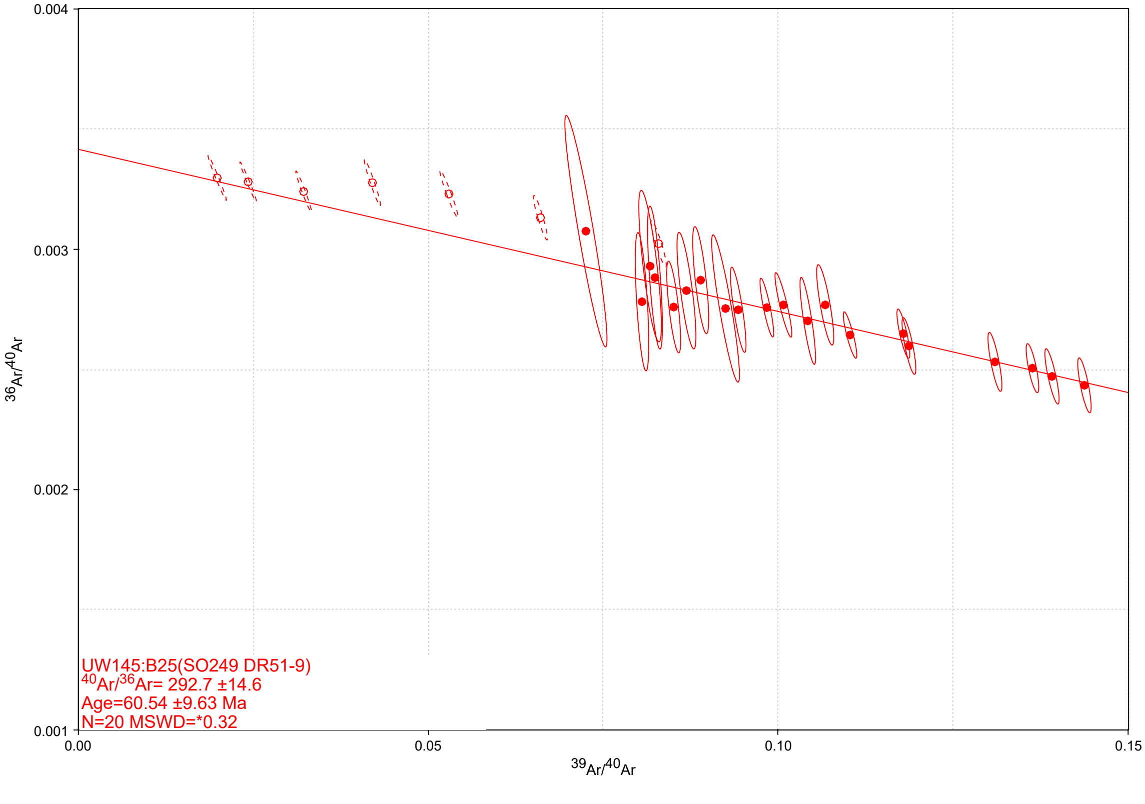
0.004
0.003
0.002
0.001
0.00
0.05
0.10
0.15
$^{36}Ar/^{40}Ar$
$^{39}Ar/^{40}Ar$
UW145:B25(SO249 DR51-9)
$^{40}Ar/^{36}Ar$= 292.7 ±14.6
Age=60.54 ±9.63 Ma
N=20 MSWD=*0.32

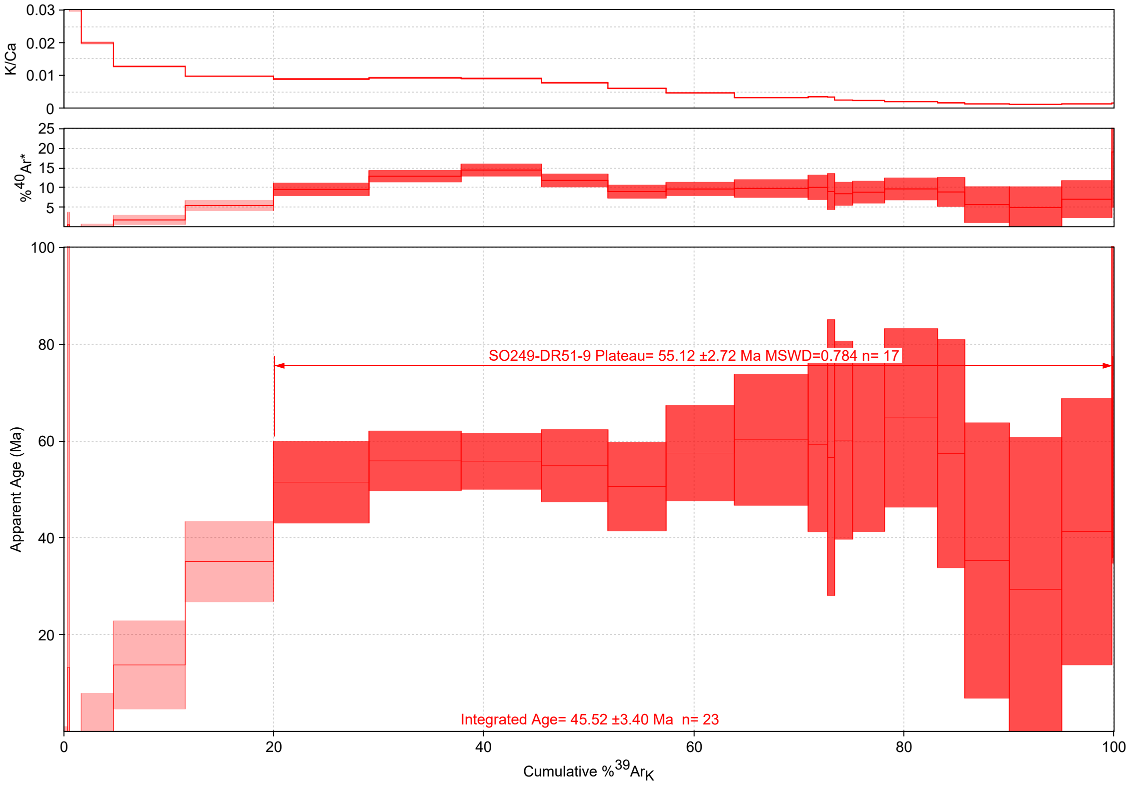

K/Ca
0.03
0.02
0.01
0
%40Ar*
25
20
15
10
5
Apparent Age (Ma)
100
80
60
40
20
SO249-DR51-9 Plateau= 55.12 ±2.72 Ma MSWD=0.784 n= 17
Integrated Age= 45.52 ±3.40 Ma n= 23
0
20
40
60
80
100
Cumulative %39ArK

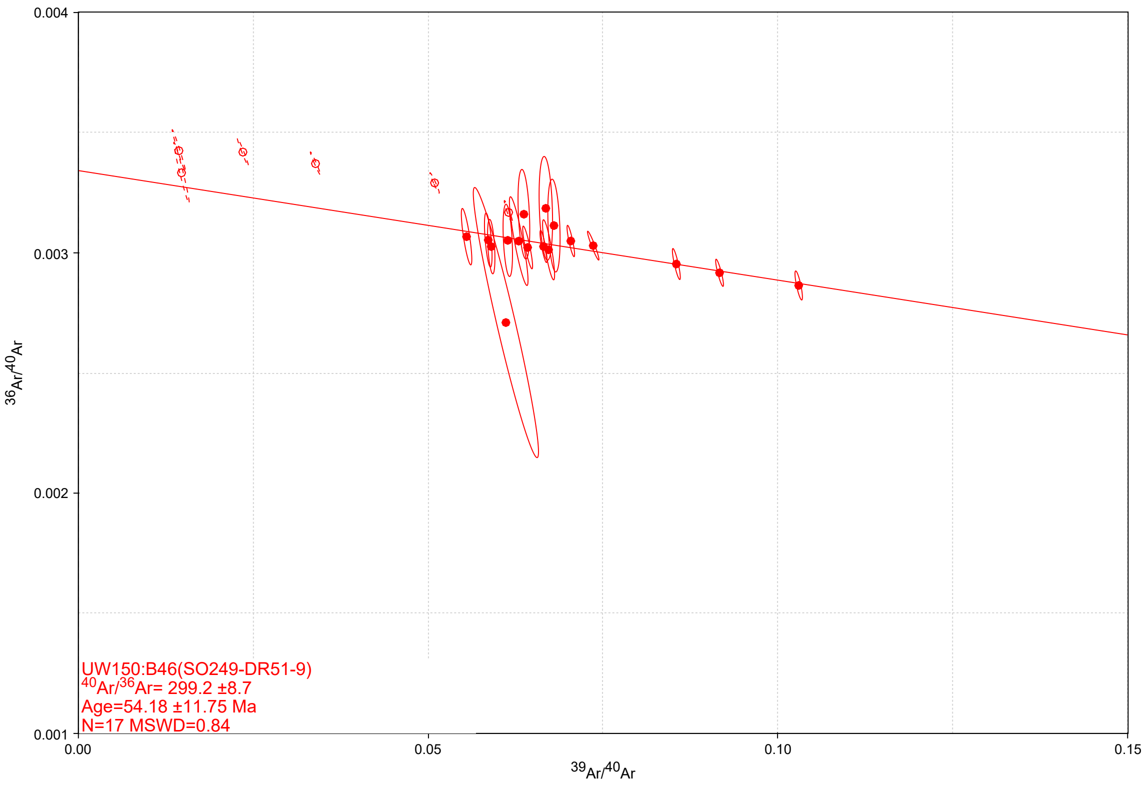
0.004
0.003
0.002
0.001
0.00
0.05
0.10
0.15
$^{36}Ar/^{40}Ar$
$^{39}Ar/^{40}Ar$
UW150:B46(SO249-DR51-9)
$^{40}Ar/^{36}Ar$= 299.2 ±8.7
Age=54.18 ±11.75 Ma
N=17 MSWD=0.84

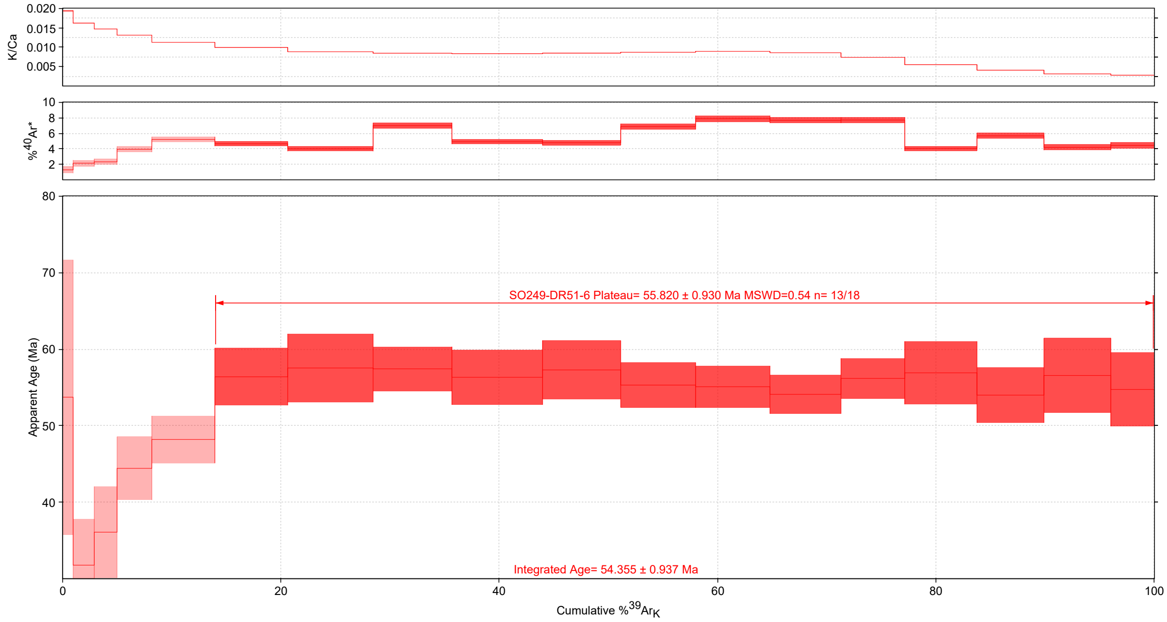

K/Ca
0.020
0.015
0.010
0.005
%^{40}Ar*
10
8
6
4
2
Apparent Age (Ma)
80
70
60
50
40
SO249-DR51-6 Plateau= 55.820 ± 0.930 Ma MSWD=0.54 n= 13/18
Integrated Age= 54.355 ± 0.937 Ma
0
20
40
60
80
100
Cumulative %^{39}Ar$_K$

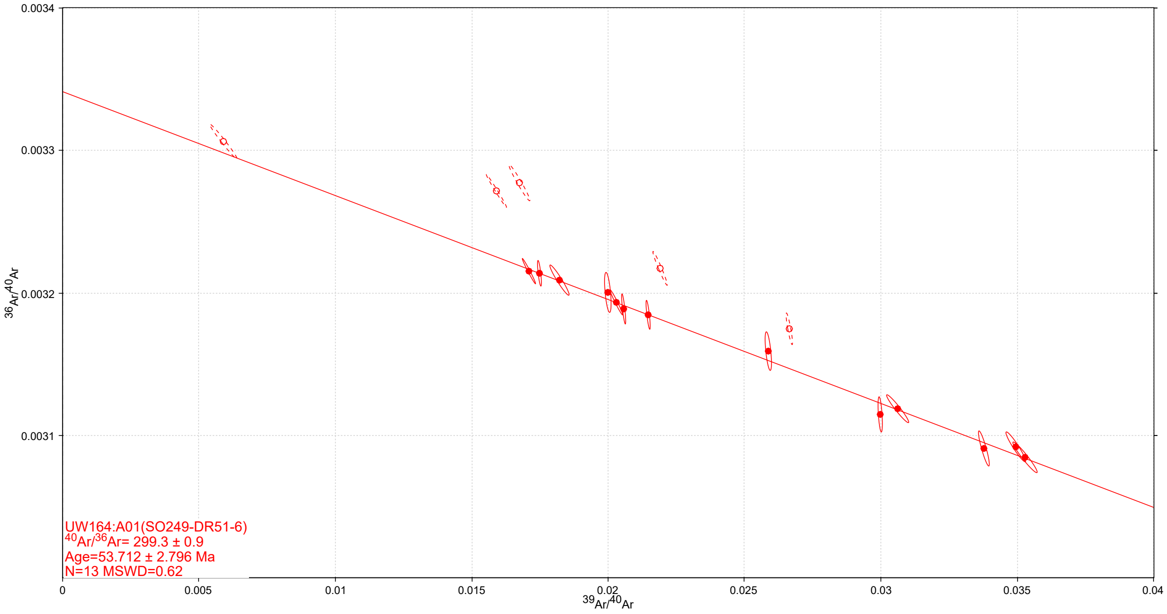

0.0034
0.0033
0.0032
0.0031
$^{36}Ar/^{40}Ar$
0
0.005
0.01
0.015
0.02
0.025
0.03
0.035
0.04
$^{39}Ar/^{40}Ar$
UW164:A01(SO249-DR51-6)
$^{40}Ar/^{36}Ar$= 299.3 ± 0.9
Age=53.712 ± 2.796 Ma
N=13 MSWD=0.62

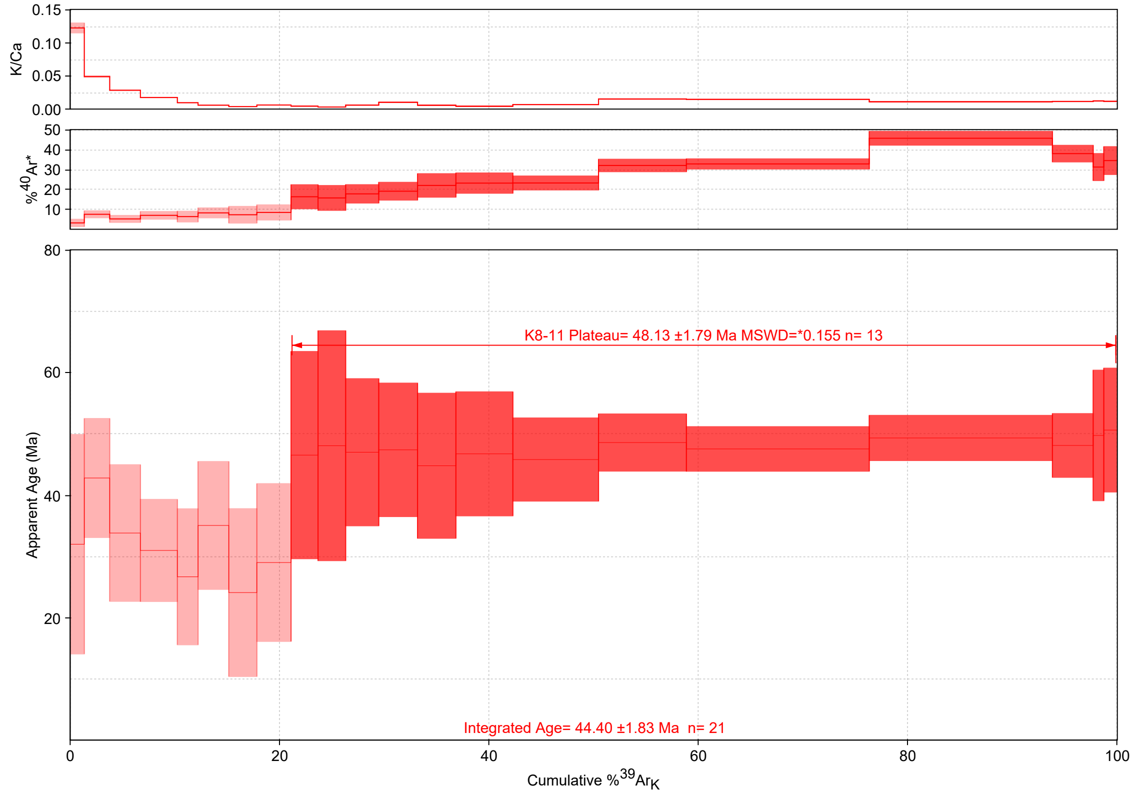

K/Ca
0.15
0.10
0.05
0.00
%40Ar*
50
40
30
20
10
Apparent Age (Ma)
80
60
40
20
K8-11 Plateau= 48.13 ±1.79 Ma MSWD=*0.155 n= 13
Integrated Age= 44.40 ±1.83 Ma n= 21
0
20
40
60
80
100
Cumulative %39ArK

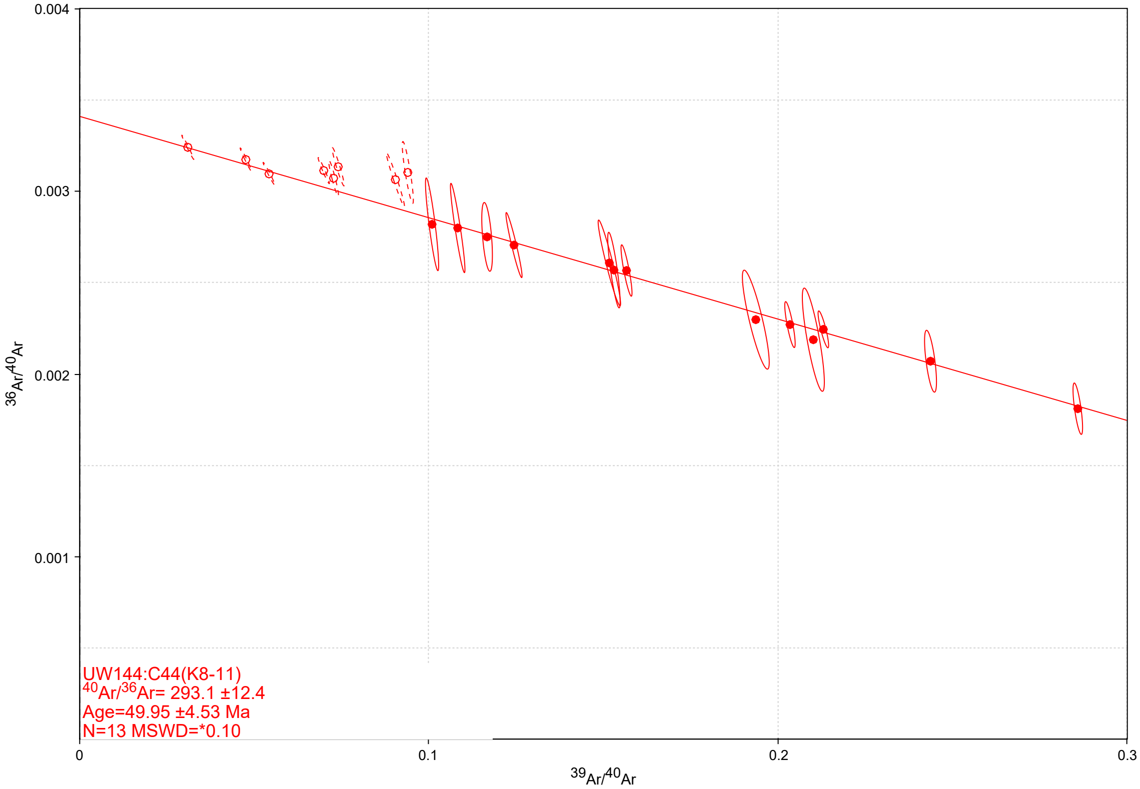
0.004
0.003
0.002
0.001
$^{36}Ar/^{40}Ar$
0
0.1
0.2
0.3
$^{39}Ar/^{40}Ar$
UW144:C44(K8-11)
$^{40}Ar/^{36}Ar$= 293.1 ±12.4
Age=49.95 ±4.53 Ma
N=13 MSWD=*0.10

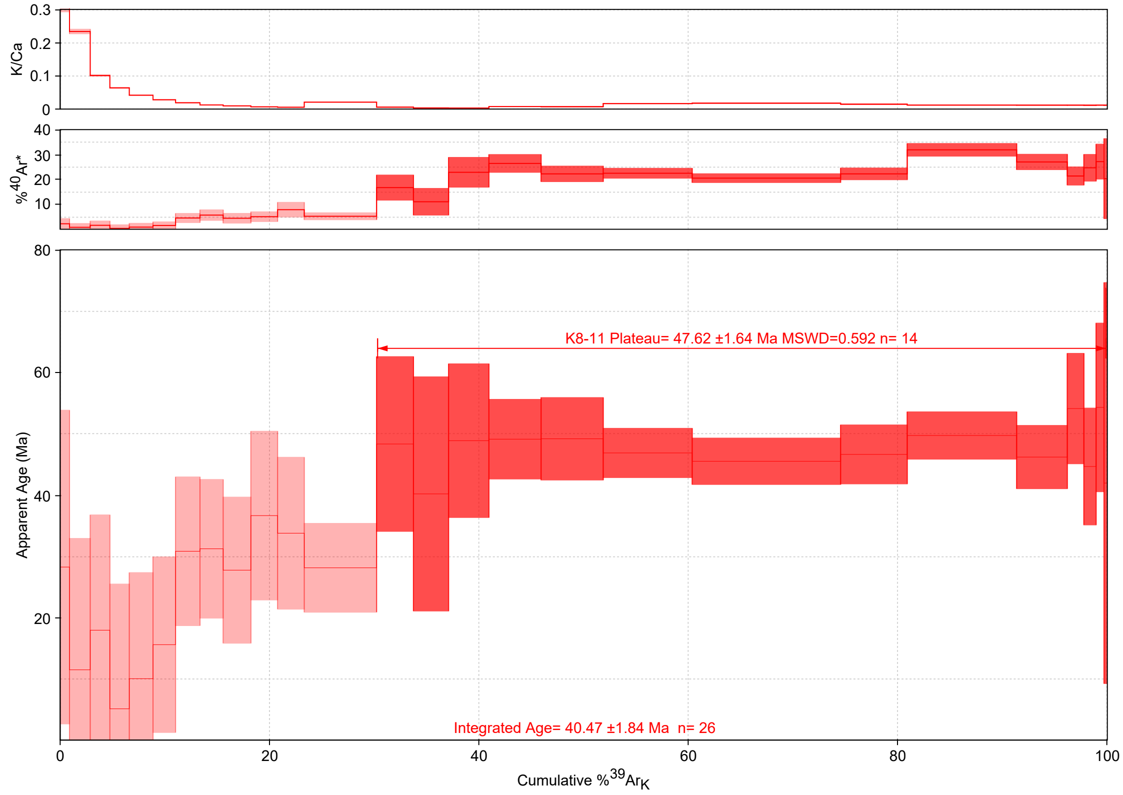

K/Ca
0.3
0.2
0.1
0
%40Ar*
40
30
20
10
Apparent Age (Ma)
80
60
40
20
K8-11 Plateau= 47.62 ±1.64 Ma MSWD=0.592 n= 14
Integrated Age= 40.47 ±1.84 Ma n= 26
0
20
40
60
80
100
Cumulative %39ArK

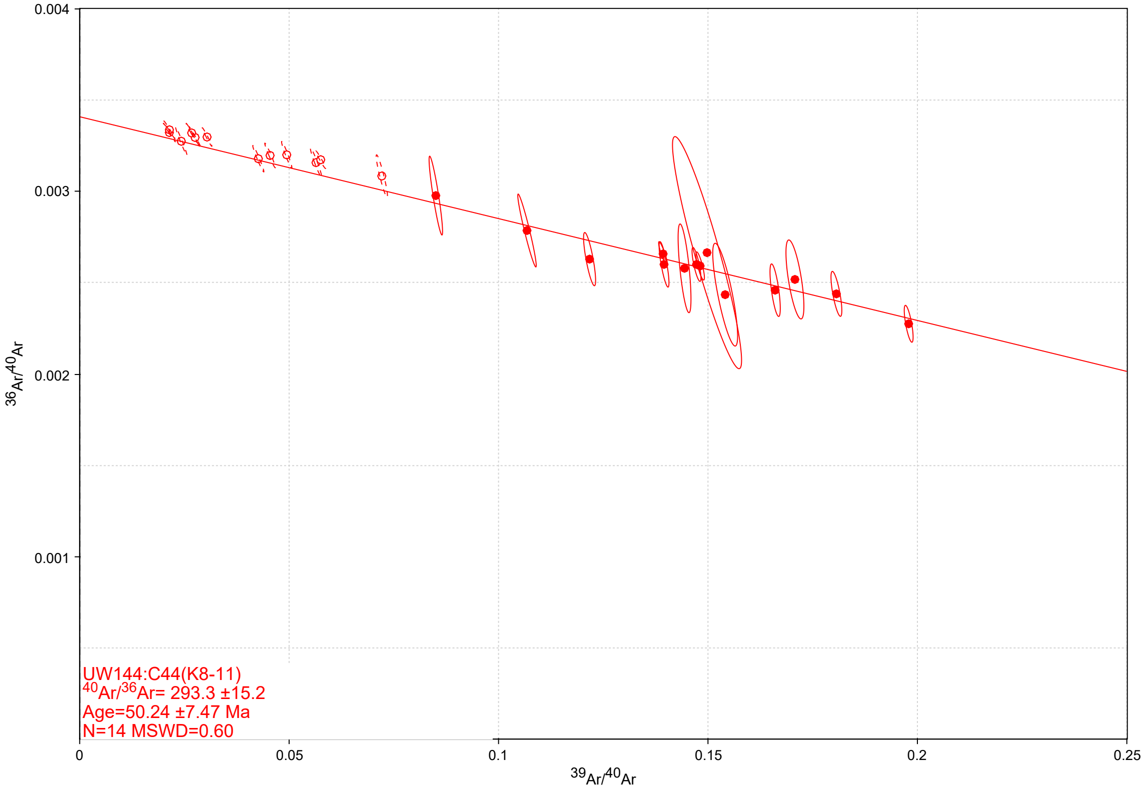

0.004
0.003
0.002
0.001
$^{36}Ar/^{40}Ar$
UW144:C44(K8-11)
$^{40}Ar/^{36}Ar$= 293.3 ±15.2
Age=50.24 ±7.47 Ma
N=14 MSWD=0.60
0
0.05
0.1
0.15
0.2
0.25
$^{39}Ar/^{40}Ar$

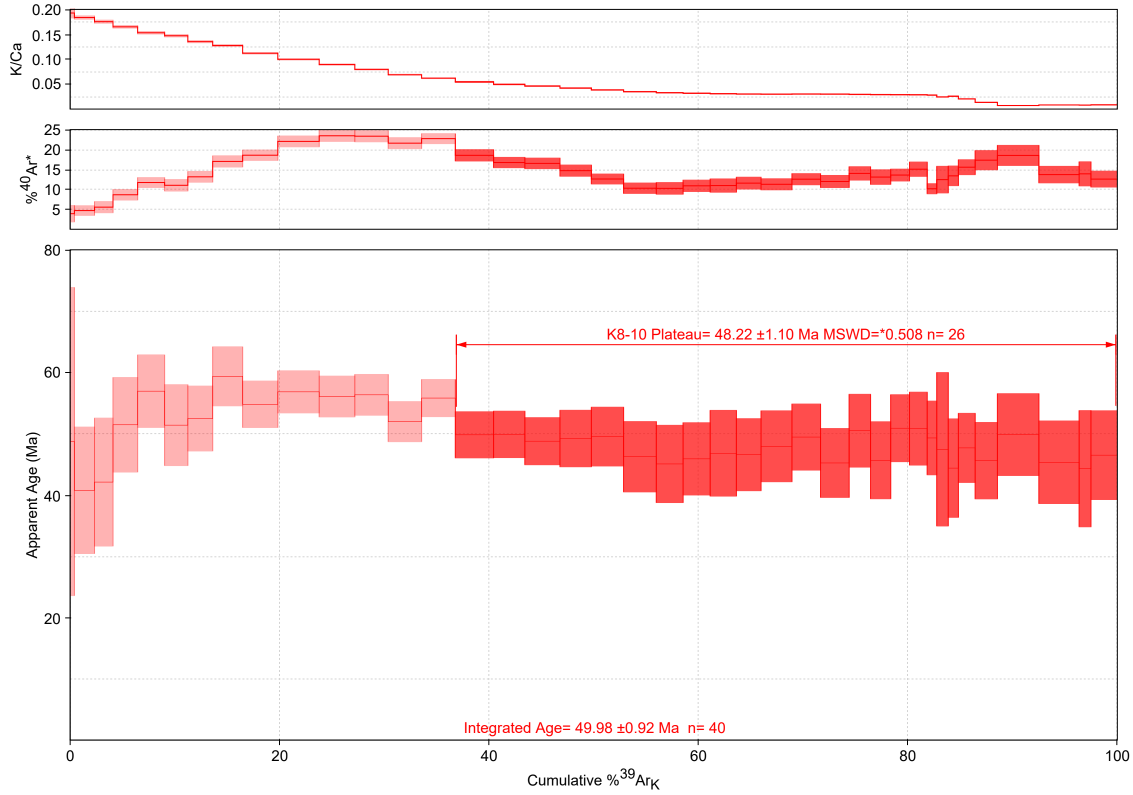

K/Ca
0.20
0.15
0.10
0.05
%40Ar*
25
20
15
10
5
Apparent Age (Ma)
80
60
40
20
K8-10 Plateau= 48.22 ±1.10 Ma MSWD=*0.508 n= 26
Integrated Age= 49.98 ±0.92 Ma n= 40
0
20
40
60
80
100
Cumulative %39ArK

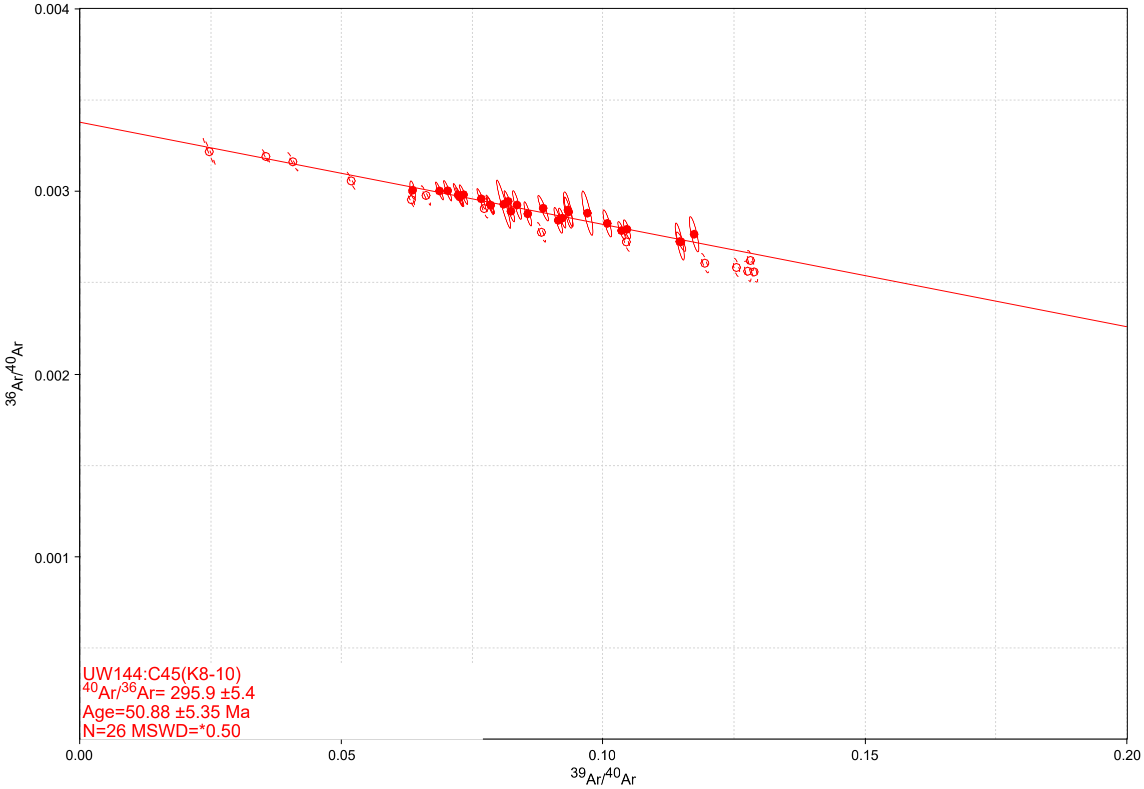
0.004
0.003
0.002
0.001
$^{36}Ar/^{40}Ar$
UW144:C45(K8-10)
$^{40}Ar/^{36}Ar$= 295.9 ±5.4
Age=50.88 ±5.35 Ma
N=26 MSWD=*0.50
0.00
0.05
0.10
0.15
0.20
$^{39}Ar/^{40}Ar$

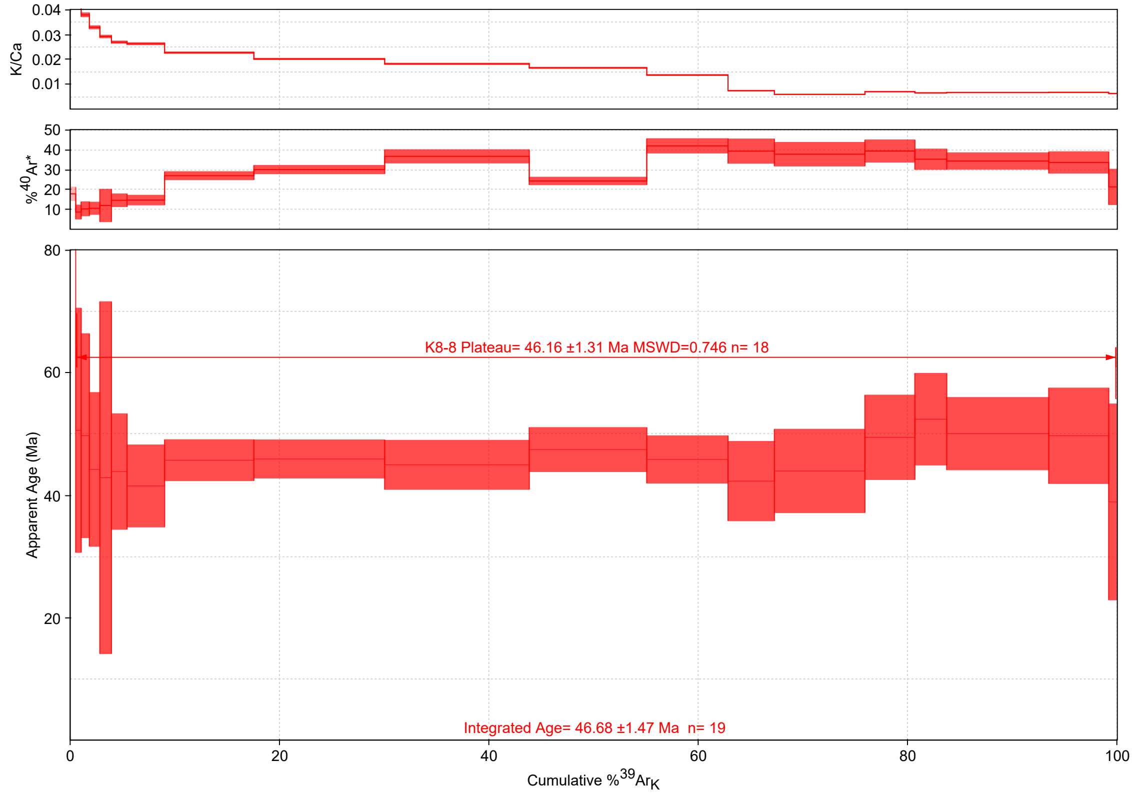

K/Ca
0.04
0.03
0.02
0.01
%40Ar*
50
40
30
20
10
Apparent Age (Ma)
80
60
40
20
K8-8 Plateau= 46.16 ±1.31 Ma MSWD=0.746 n= 18
Integrated Age= 46.68 ±1.47 Ma n= 19
0
20
40
60
80
100
Cumulative %39ArK

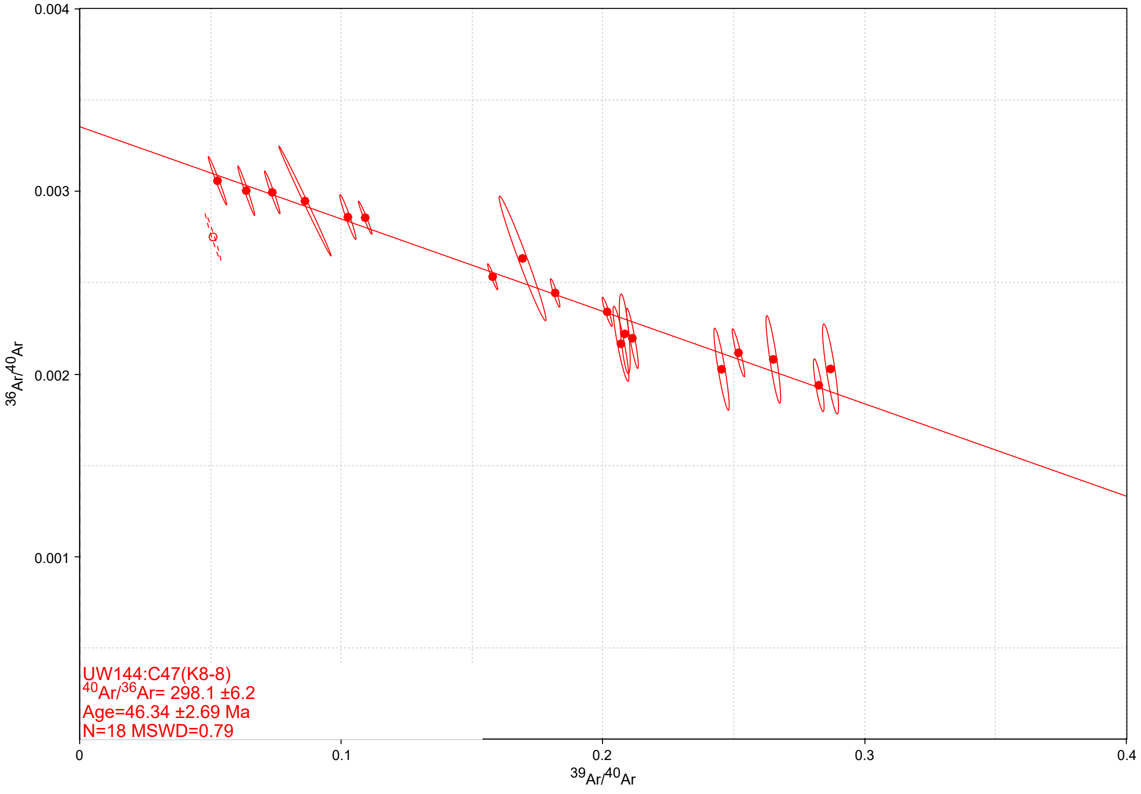

0.004
0.003
0.002
0.001
$^{36}Ar/^{40}Ar$
UW144:C47(K8-8)
$^{40}Ar/^{36}Ar$= 298.1 ±6.2
Age=46.34 ±2.69 Ma
N=18 MSWD=0.79
0
0.1
0.2
0.3
0.4
$^{39}Ar/^{40}Ar$

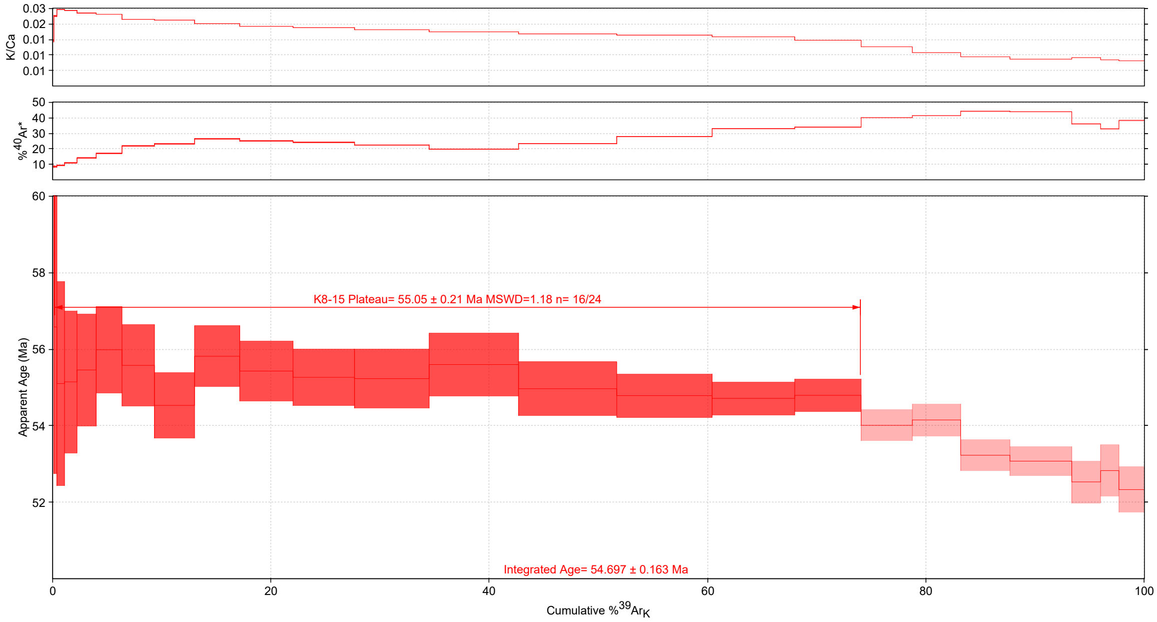

K/Ca
%40Ar*
Apparent Age (Ma)
K8-15 Plateau= 55.05 ± 0.21 Ma MSWD=1.18 n= 16/24
Integrated Age= 54.697 ± 0.163 Ma
Cumulative %39ArK

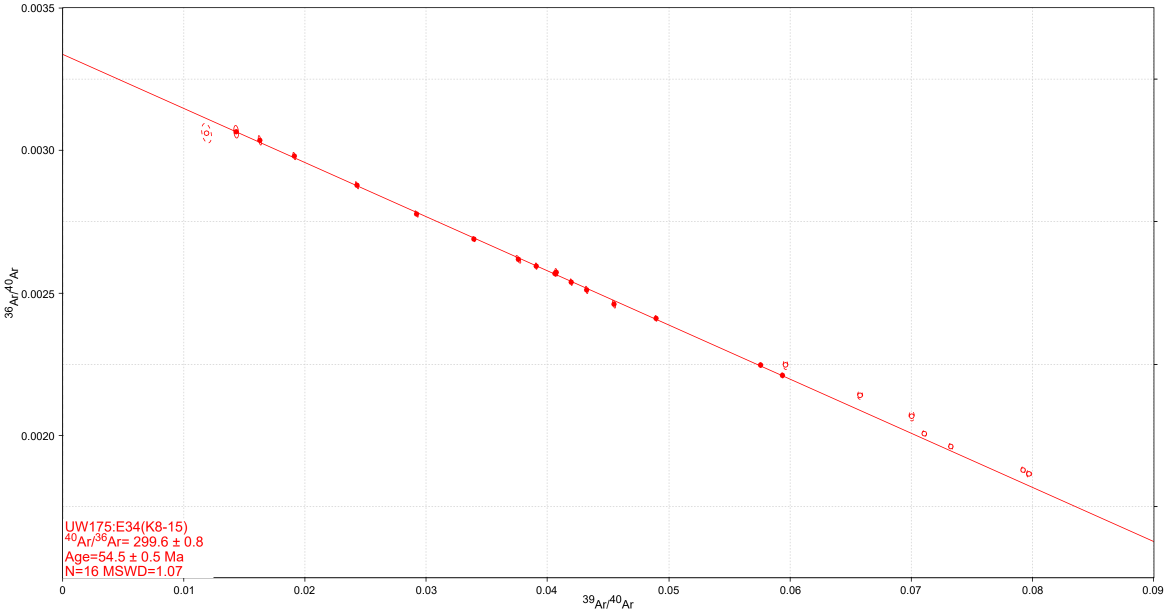
0.0035
0.0030
0.0025
0.0020
$^{36}Ar/^{40}Ar$
UW175:E34(K8-15)
$^{40}Ar/^{36}Ar$= 299.6 ± 0.8
Age=54.5 ± 0.5 Ma
N=16 MSWD=1.07
0
0.01
0.02
0.03
0.04
0.05
0.06
0.07
0.08
0.09
$^{39}Ar/^{40}Ar$

Supplementary Fig. 3: GPlates Orthographic projections for Pacific realm followed by Albers Equal Area projections for North Pacific realm from 40 to 60 Myr ago for every million years in a fixed Antarctic reference frame. Latitude and longitude lines are spaced every 15°. All panels cover the same latitudes and longitudes as shown on the 40 Ma panels. Paleogeographic reconstruction data modified from (*69,70*) licensed under CC BY 4.0 (https://creativecommons.org/licenses/by/4.0/).

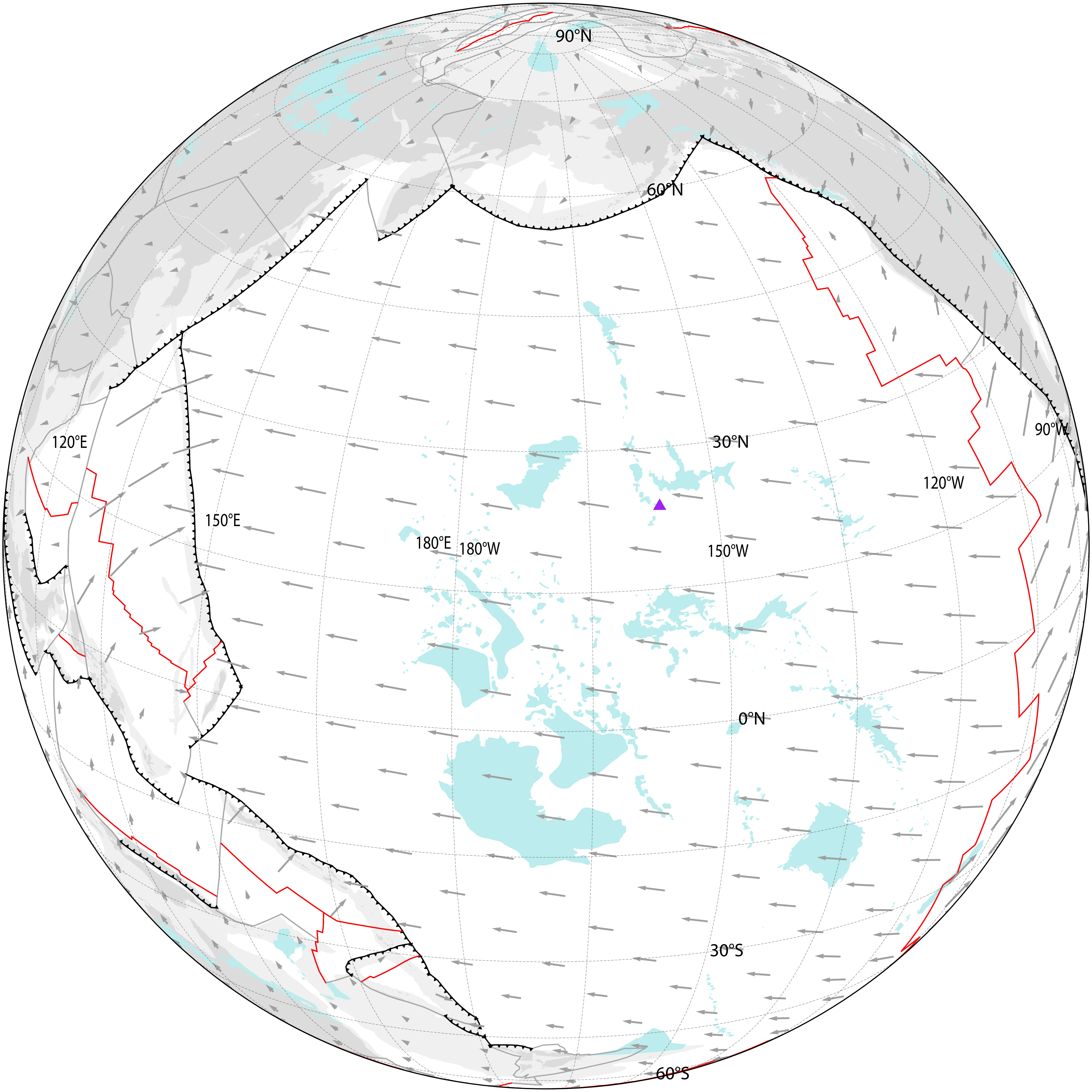

40 Ma

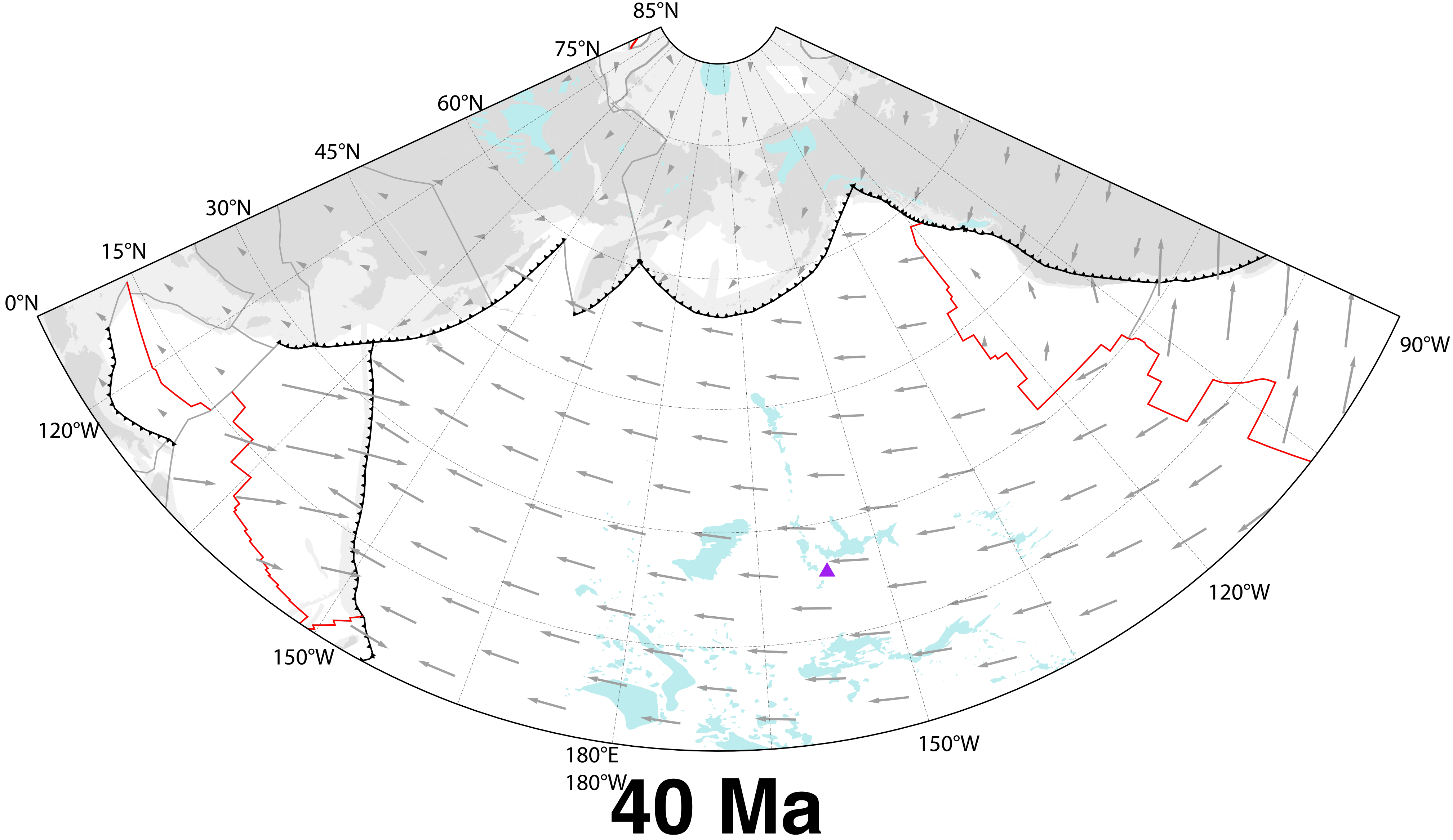
85°N
75°N
60°N
45°N
30°N
15°N
0°N
120°W
150°W
180°E
180°W
150°W
120°W
90°W
40 Ma

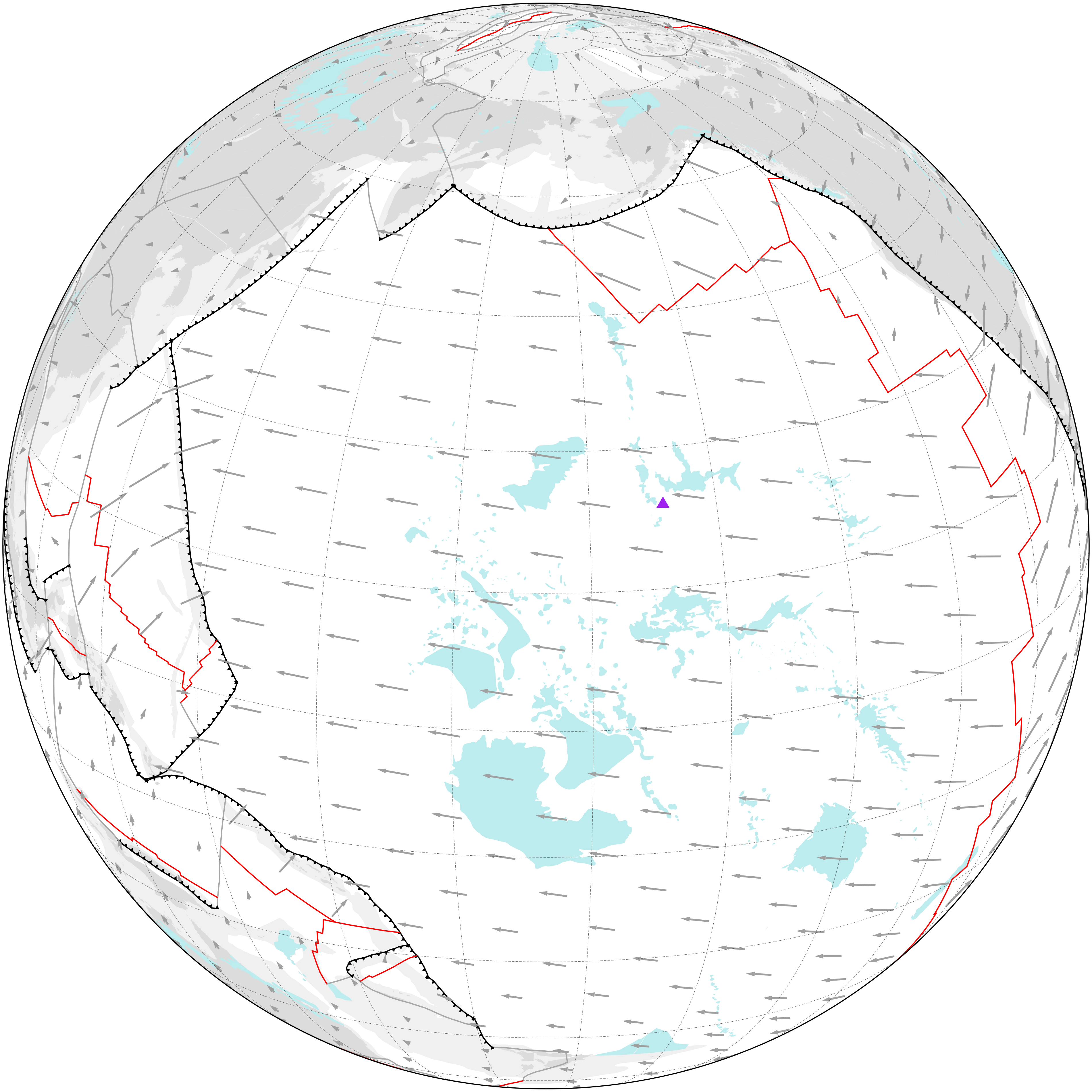

41 Ma

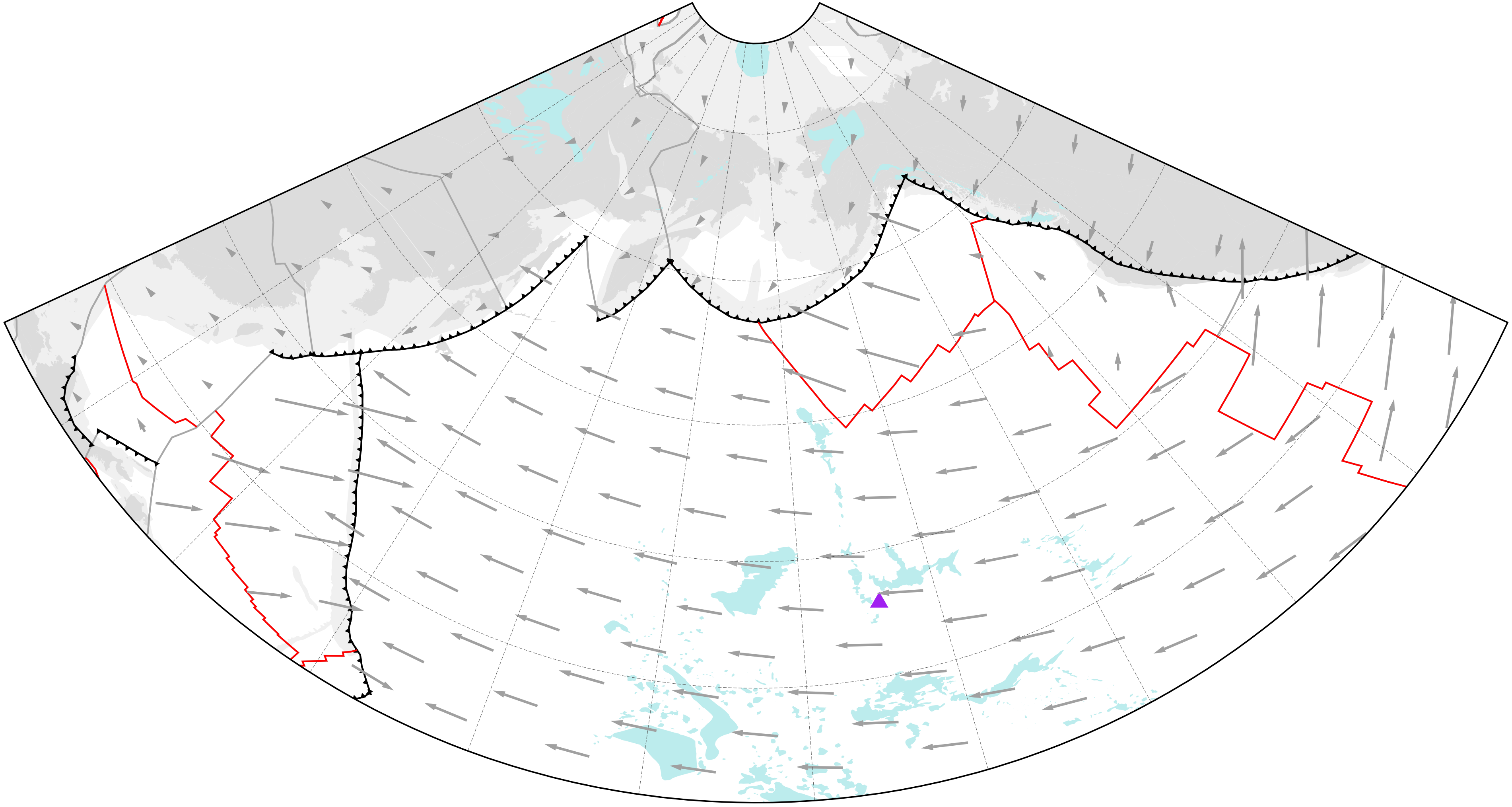

41 Ma

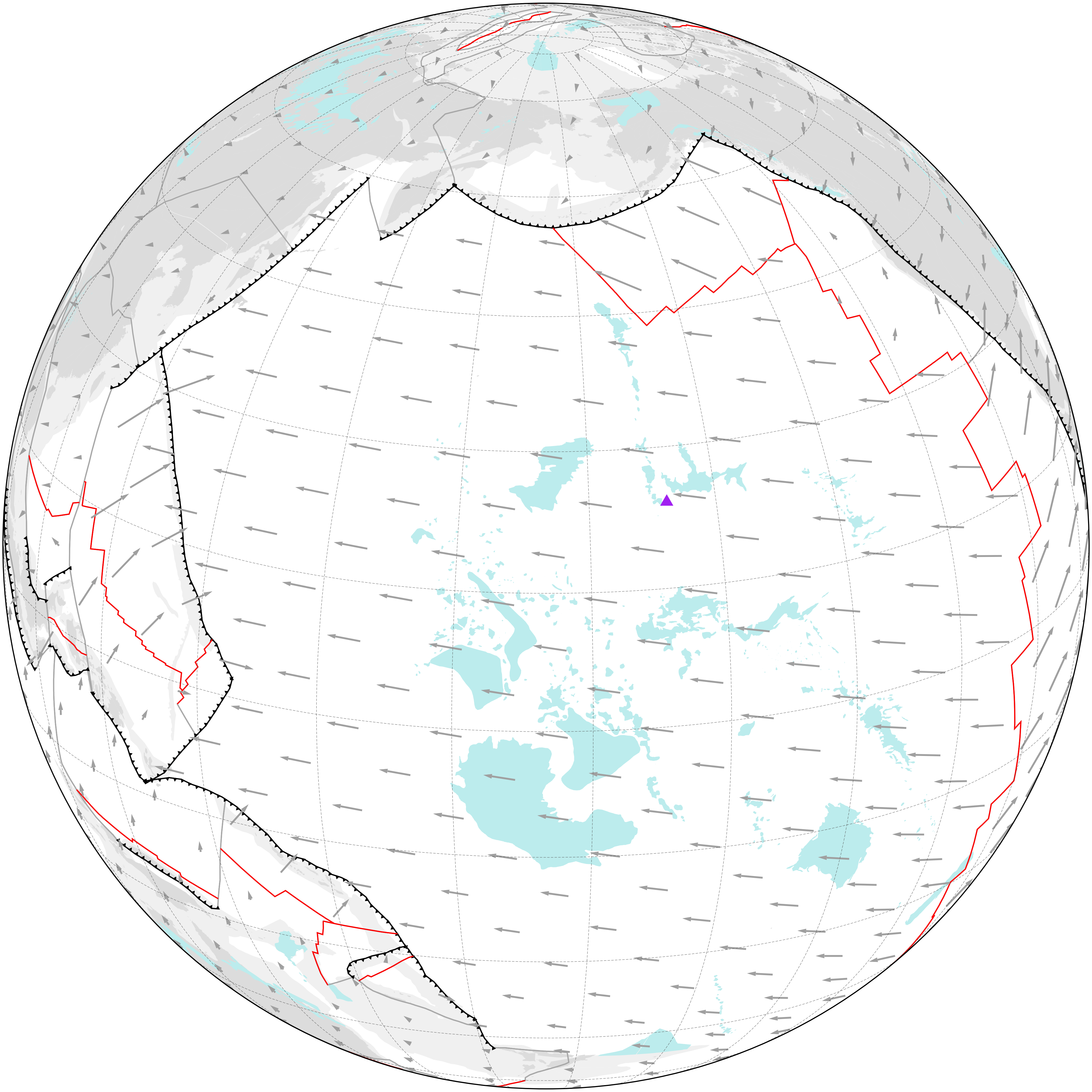

42 Ma

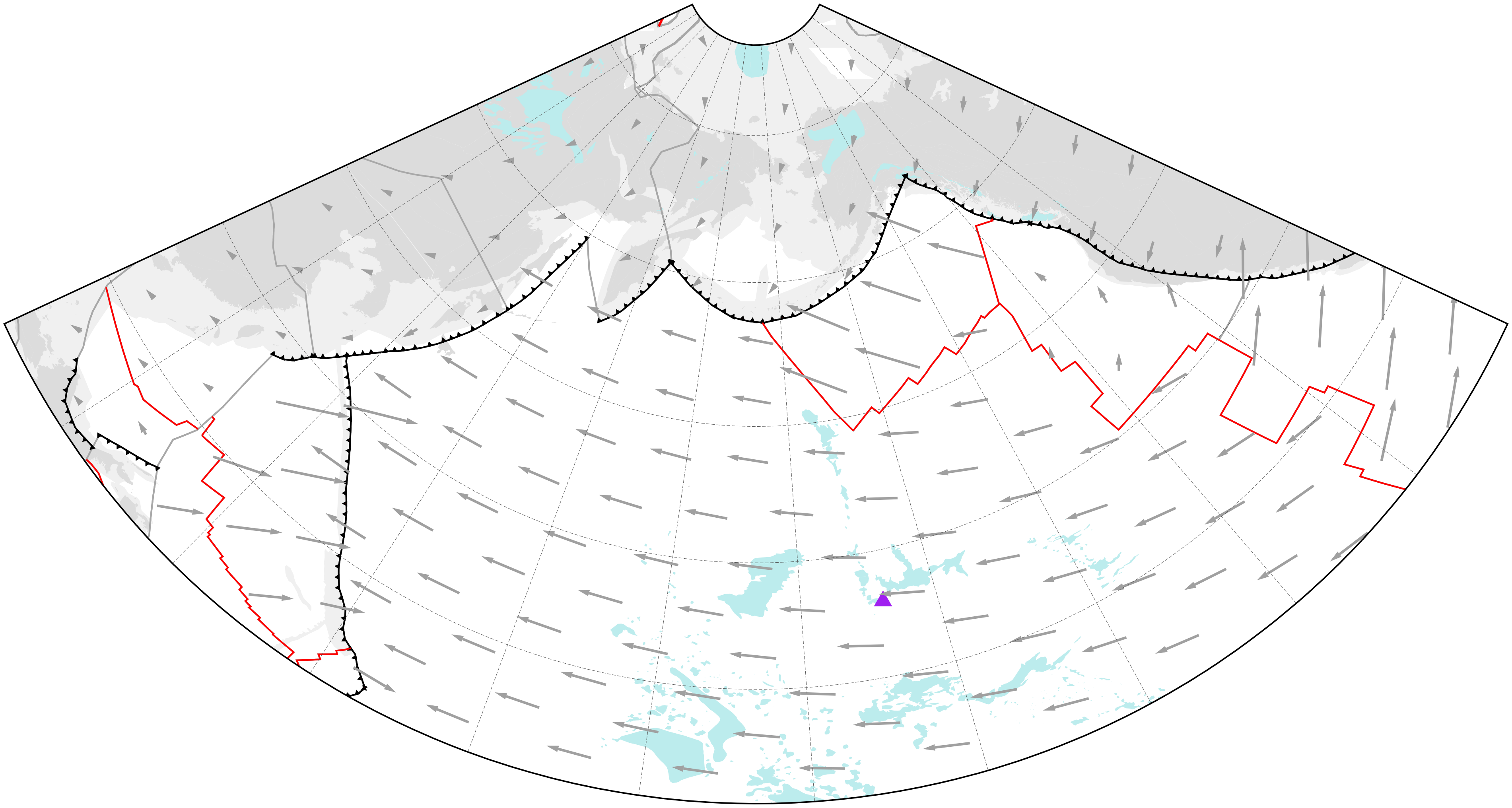

42 Ma

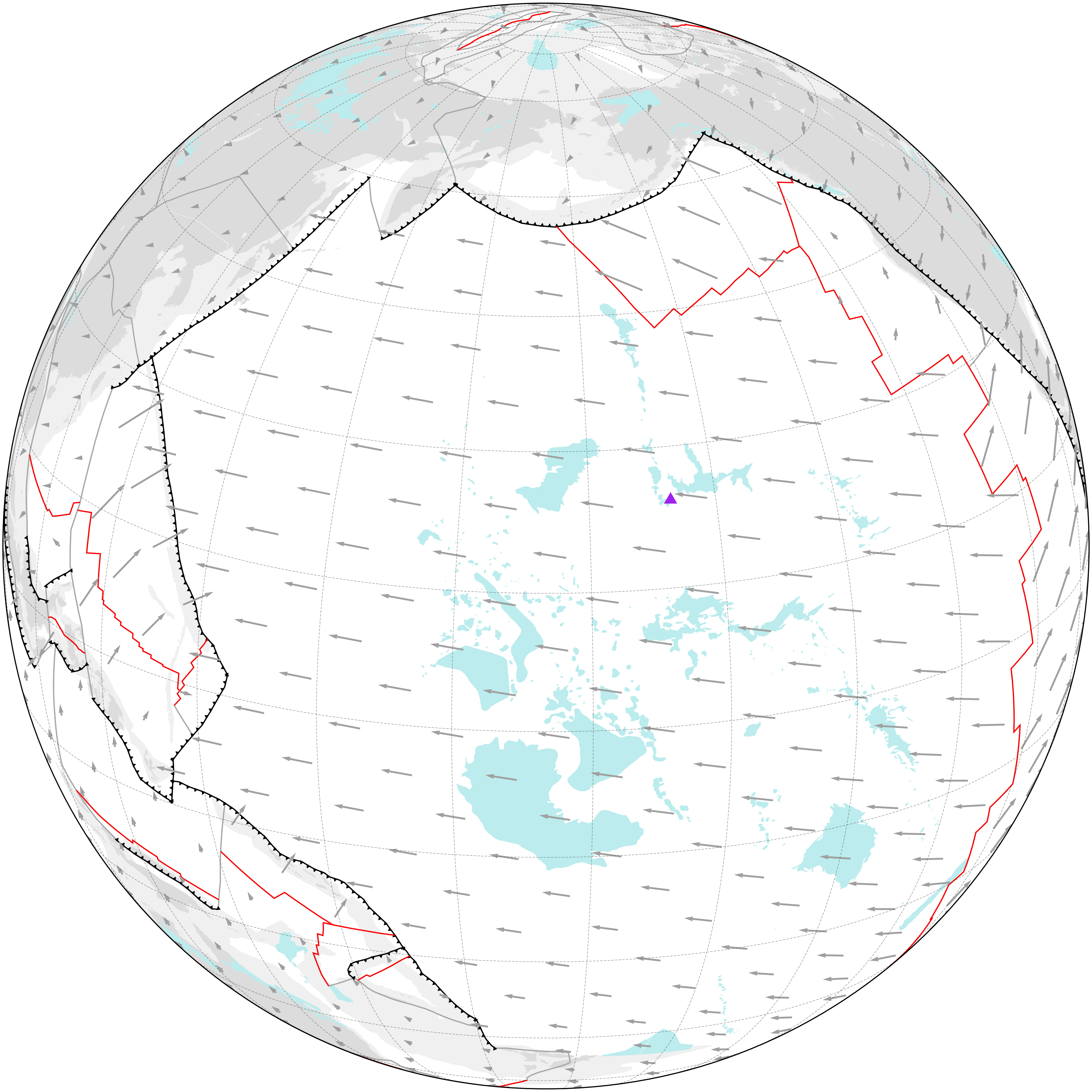

43 Ma

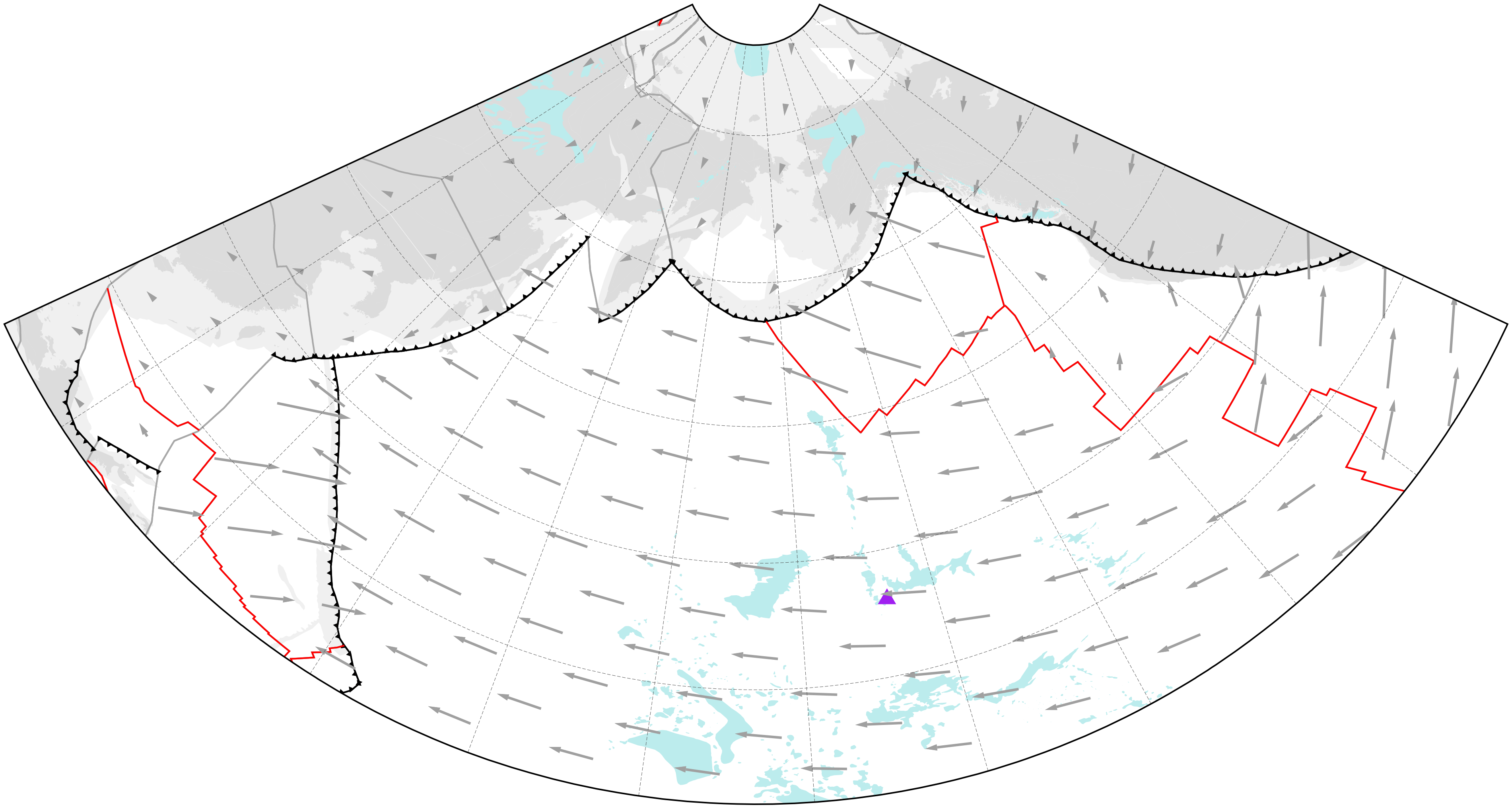

43 Ma

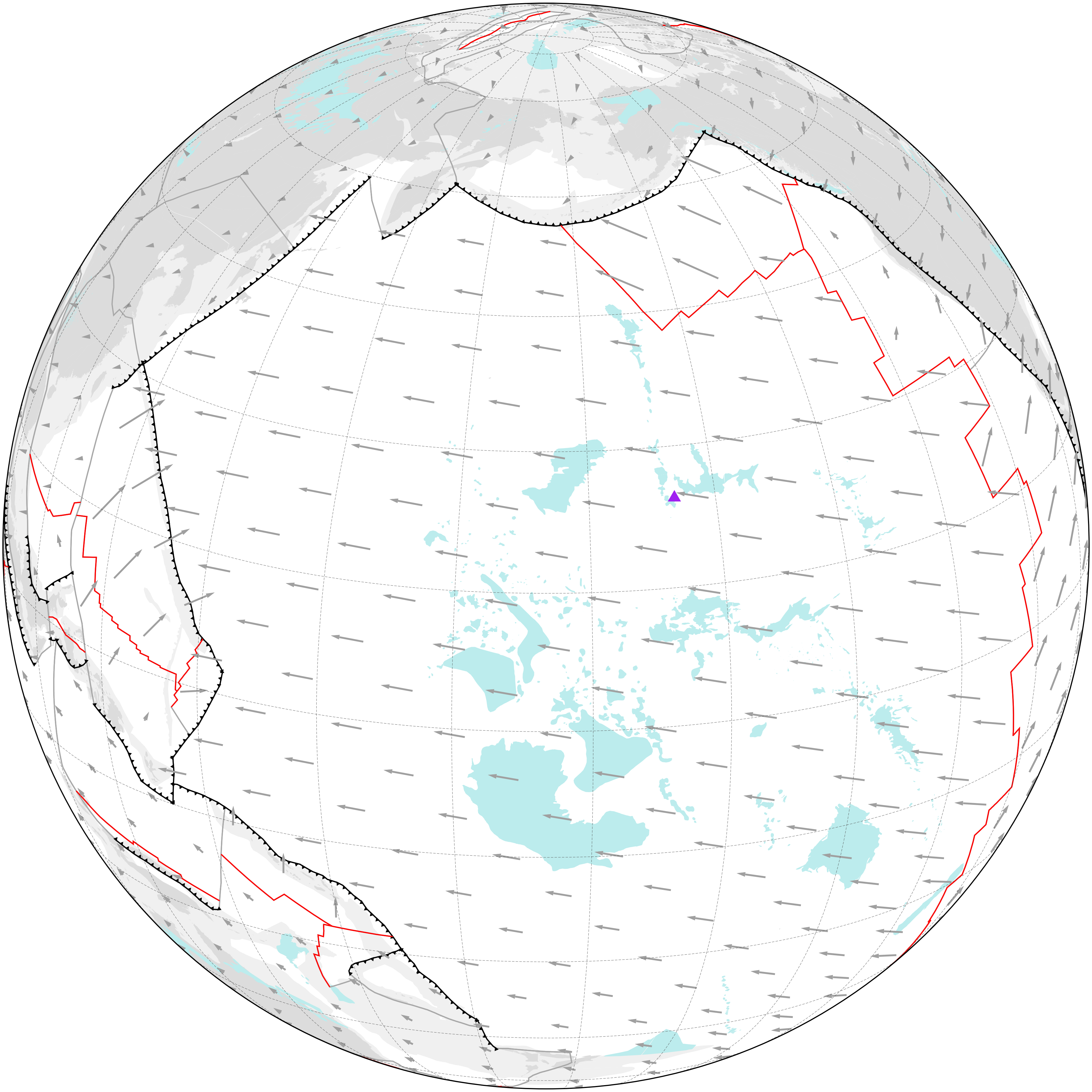

44 Ma

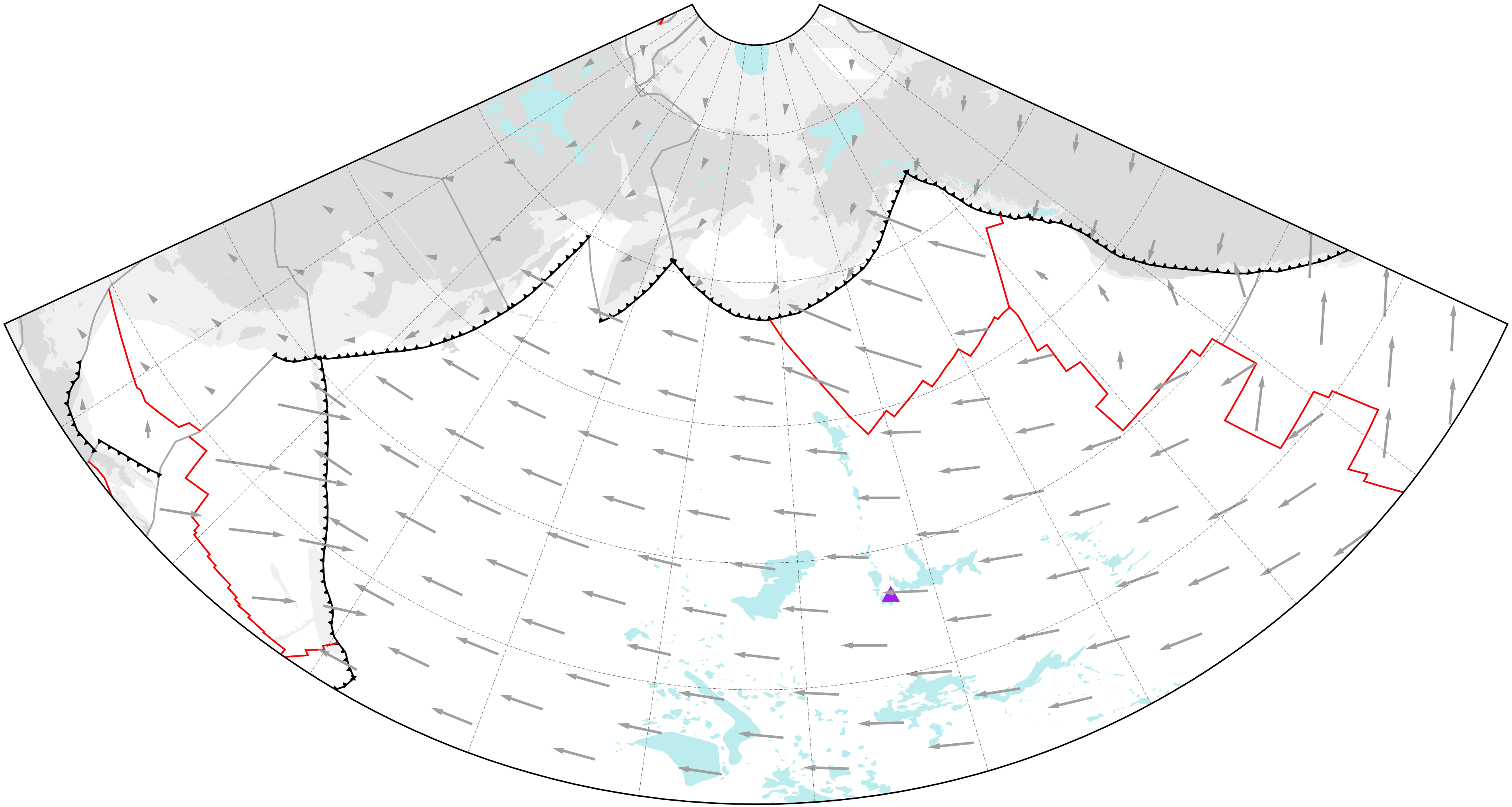

44 Ma

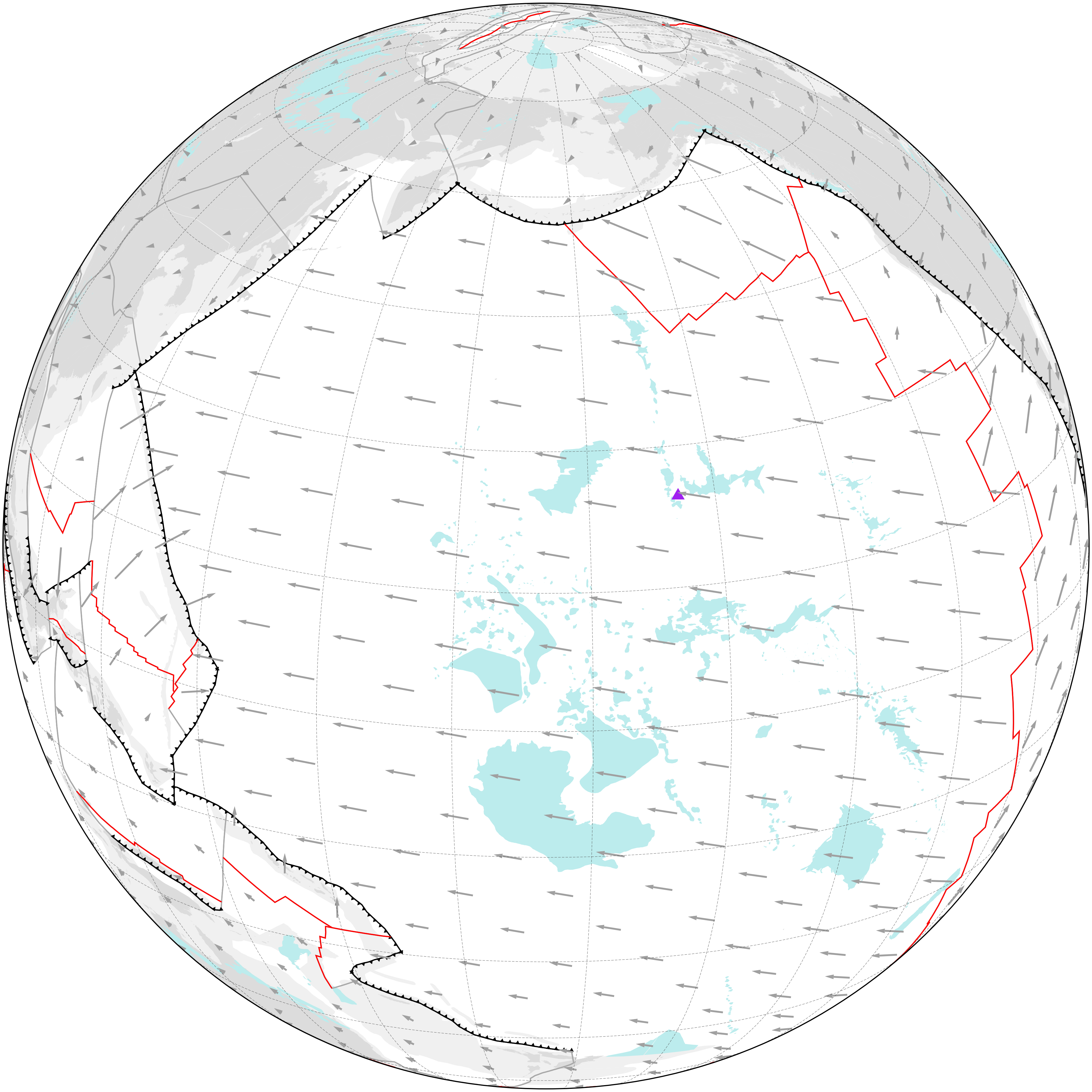

45 Ma

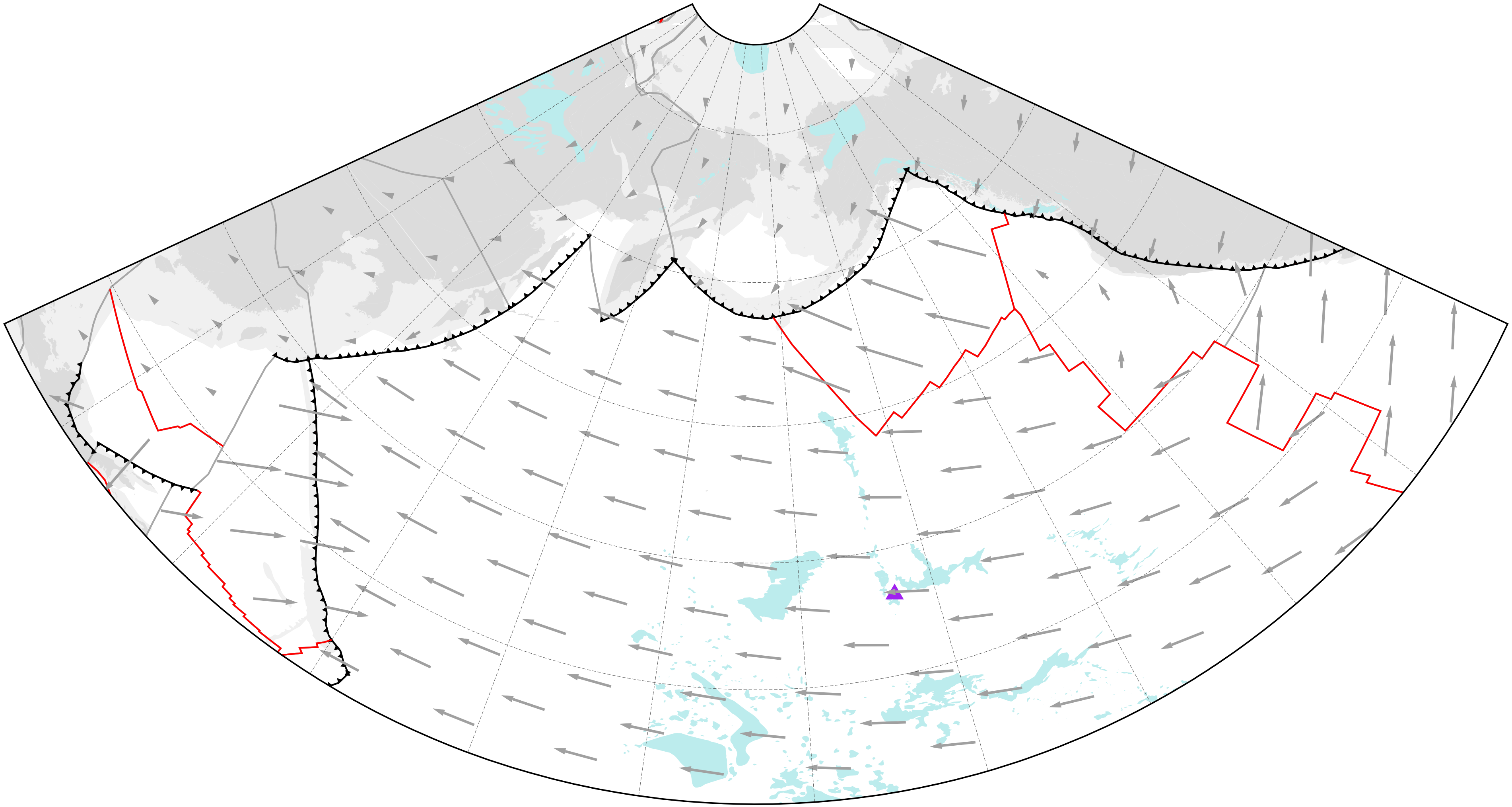

45 Ma

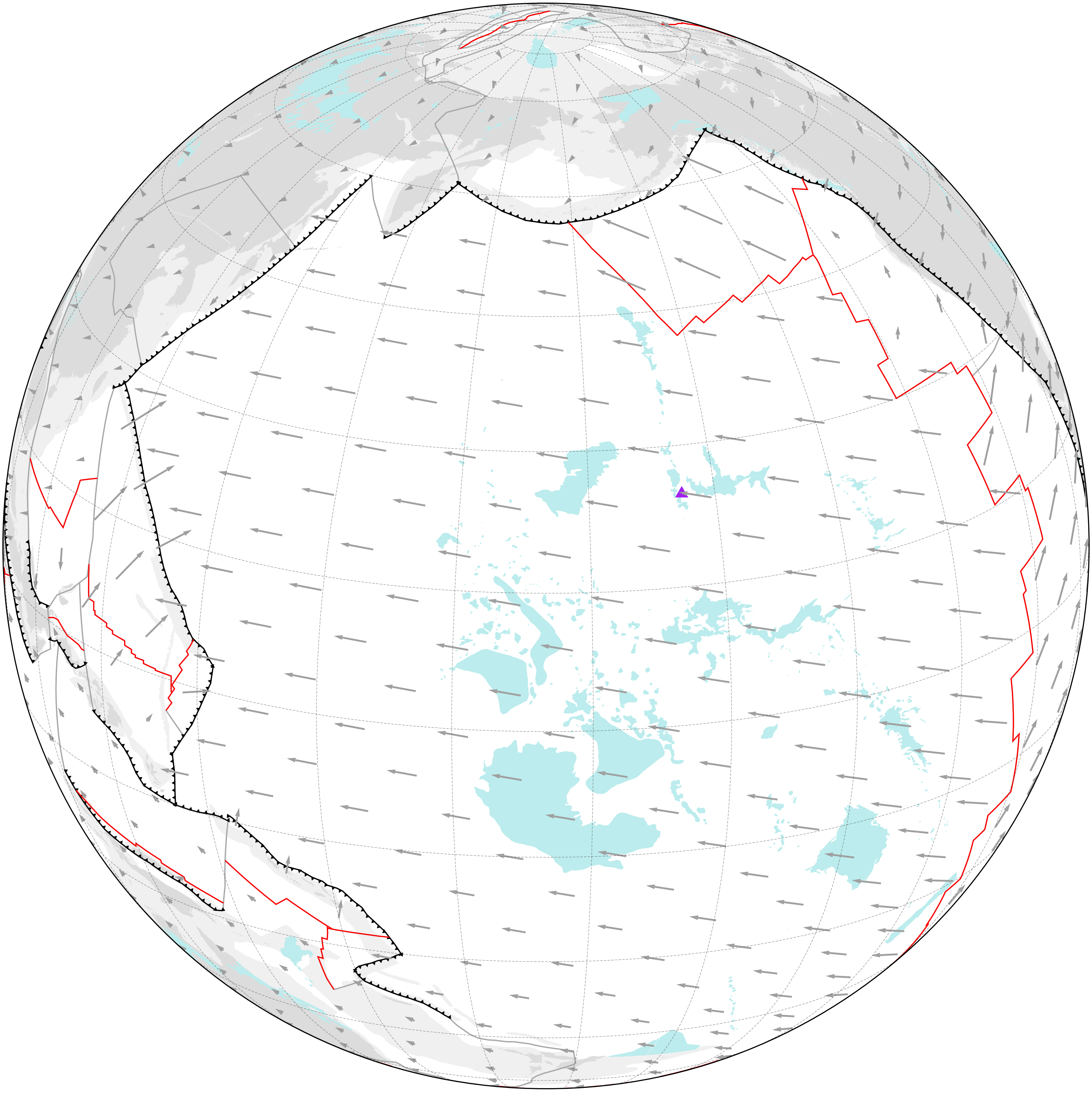

46 Ma

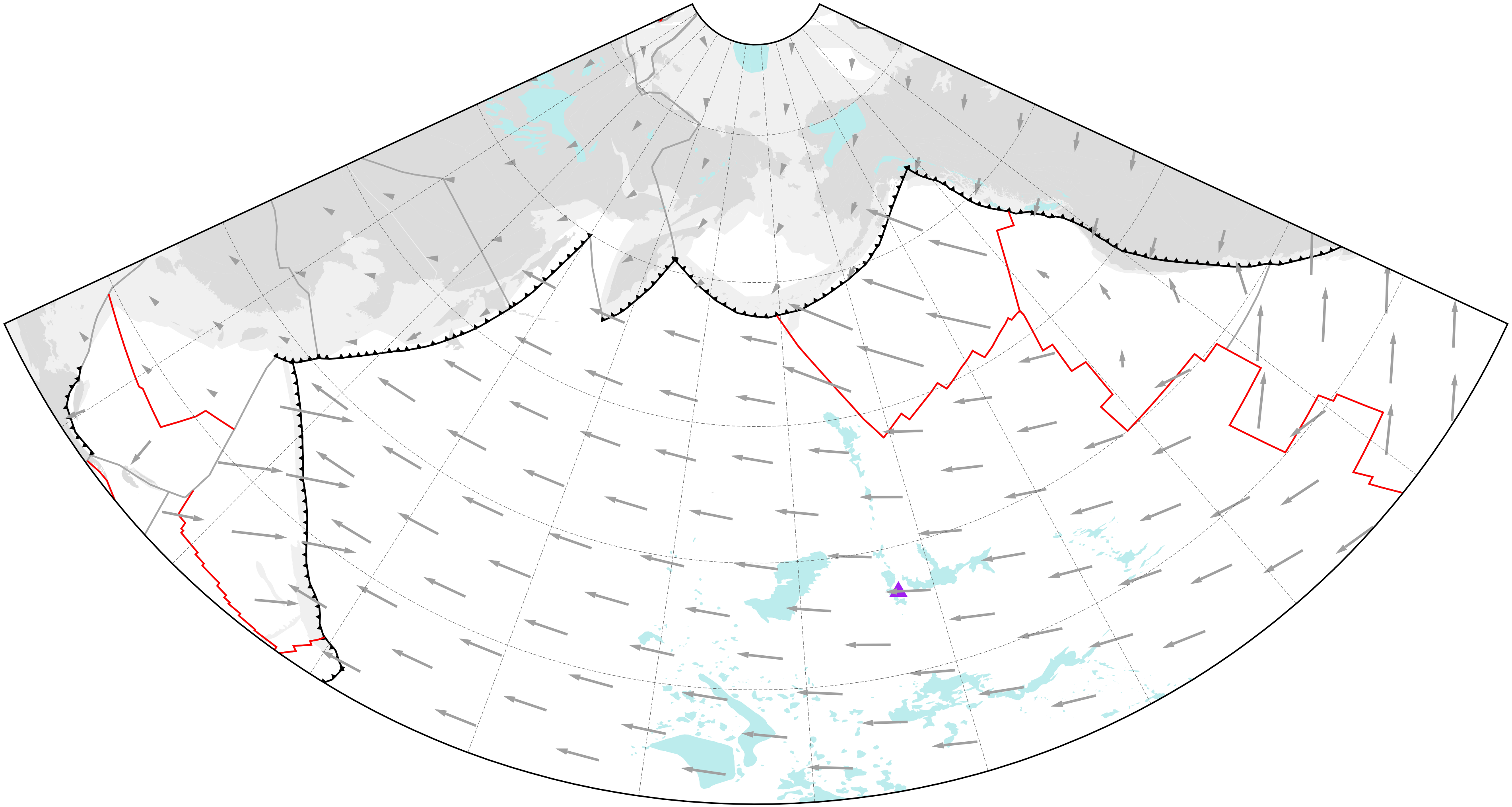

46 Ma

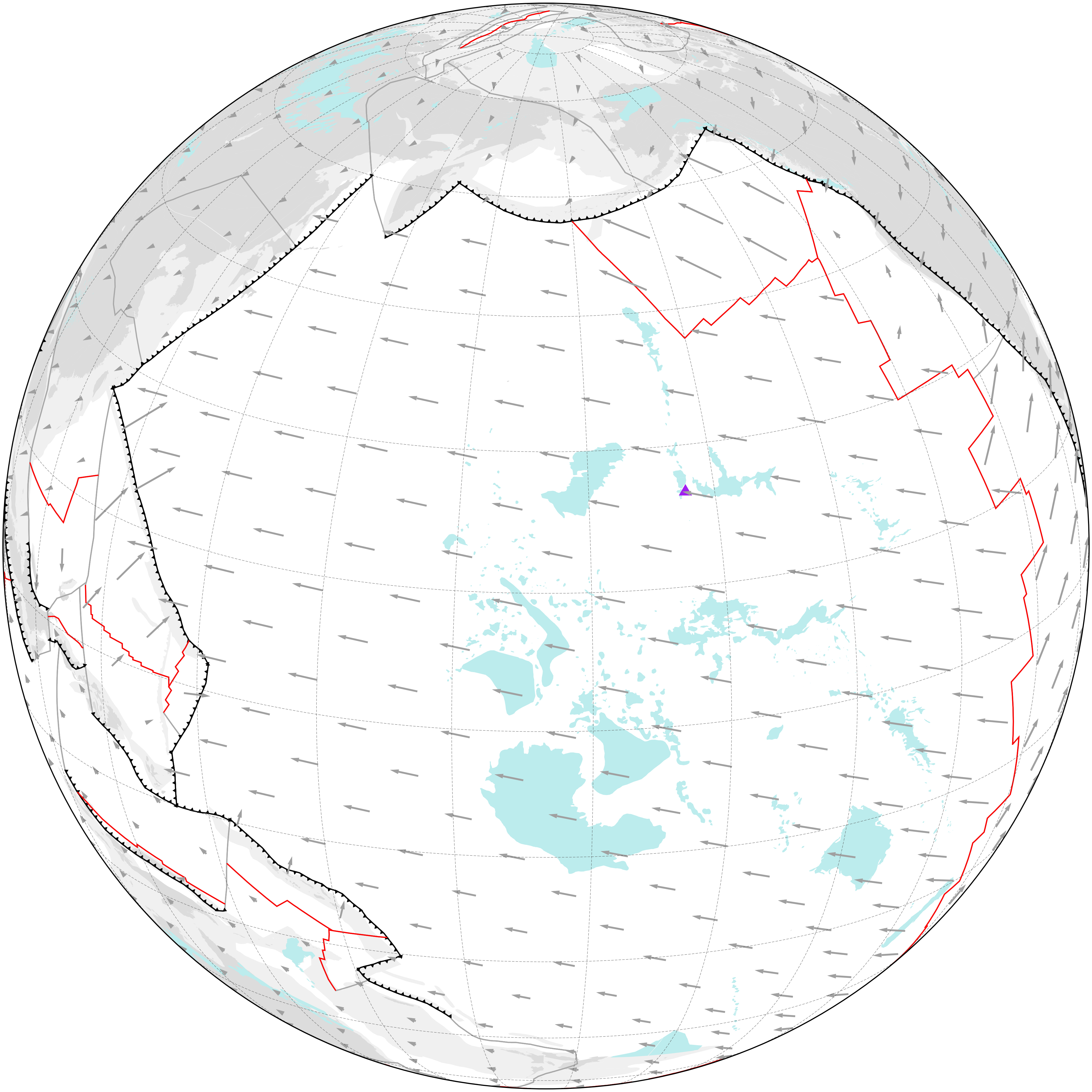

47 Ma

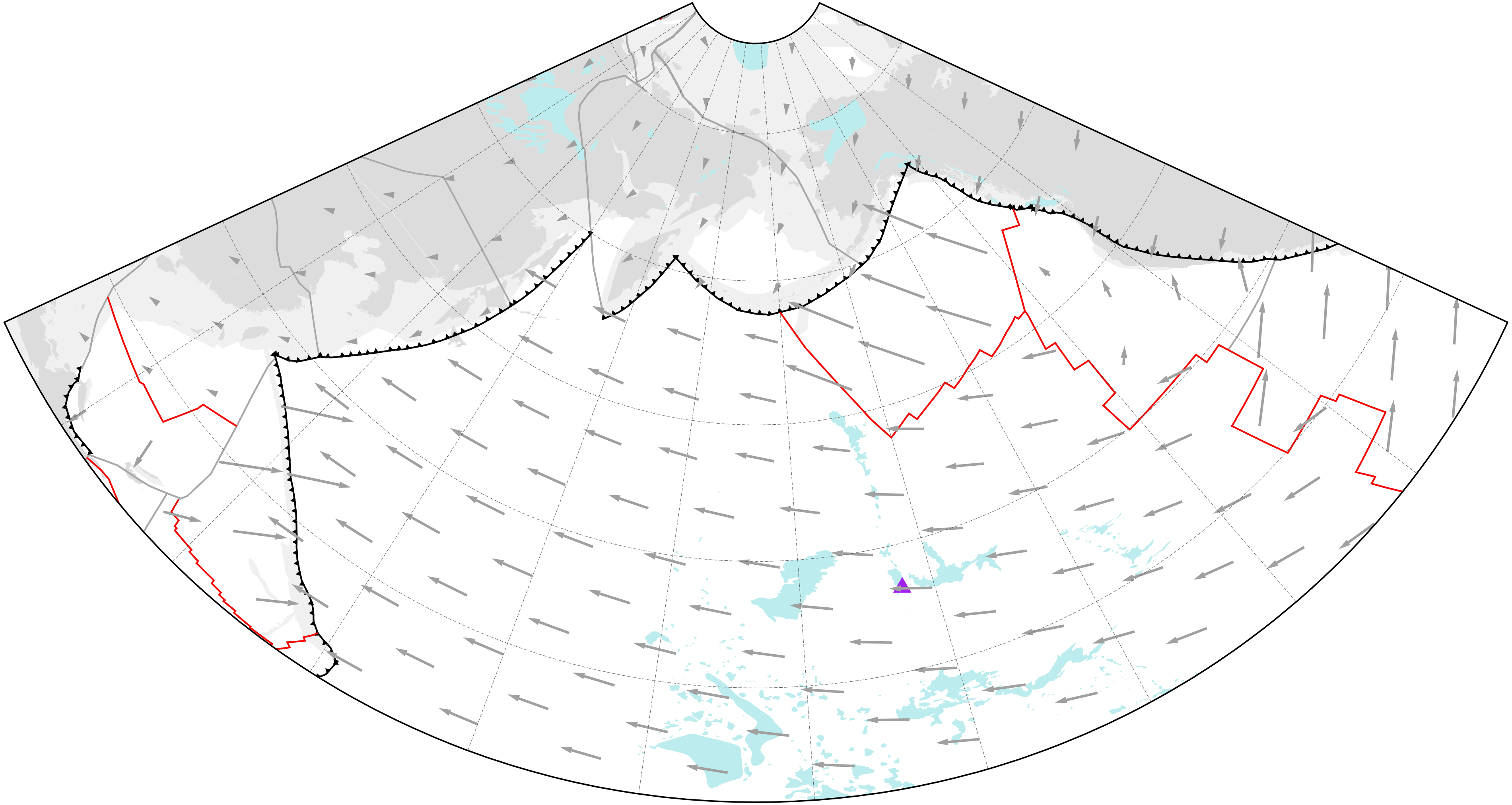

47 Ma

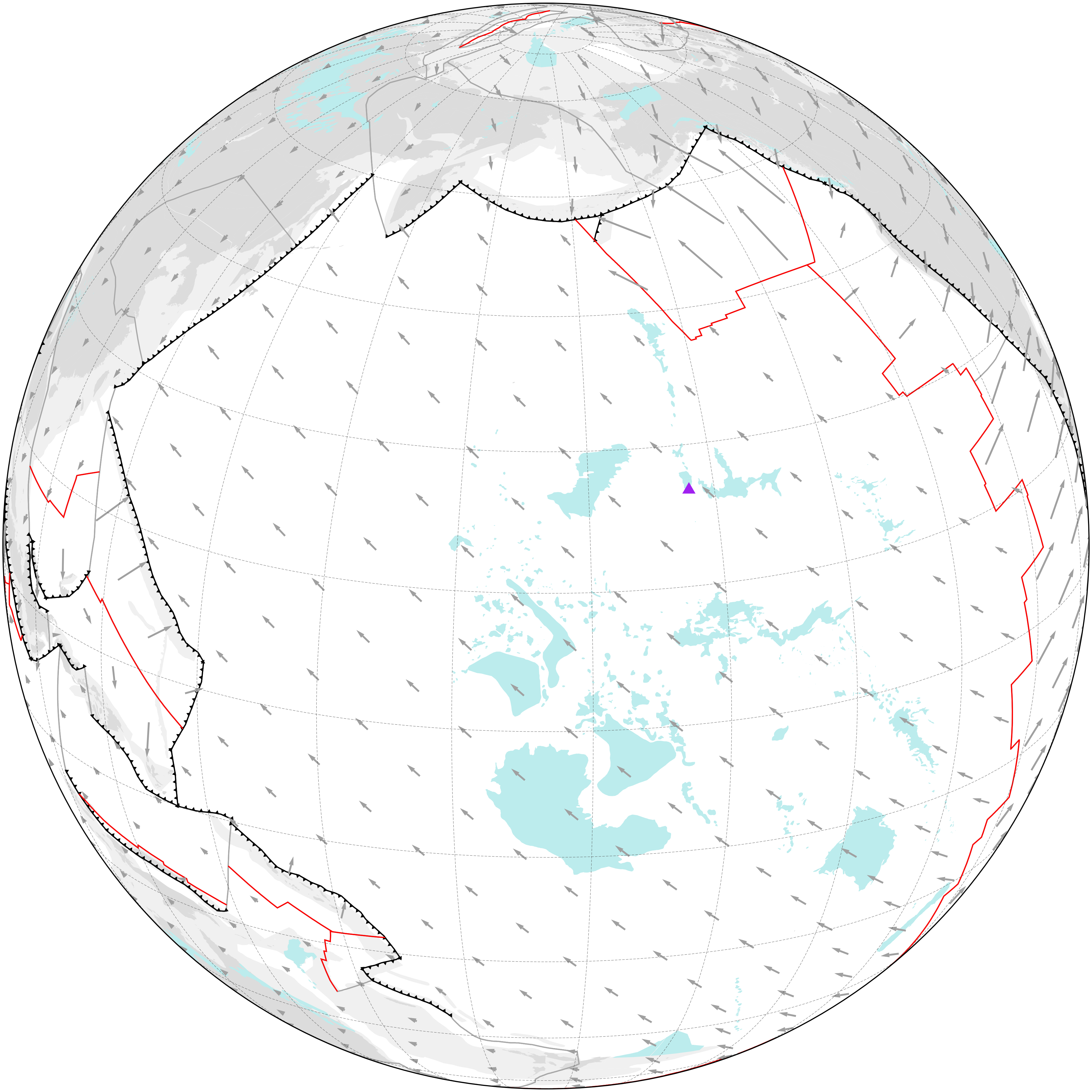

48 Ma

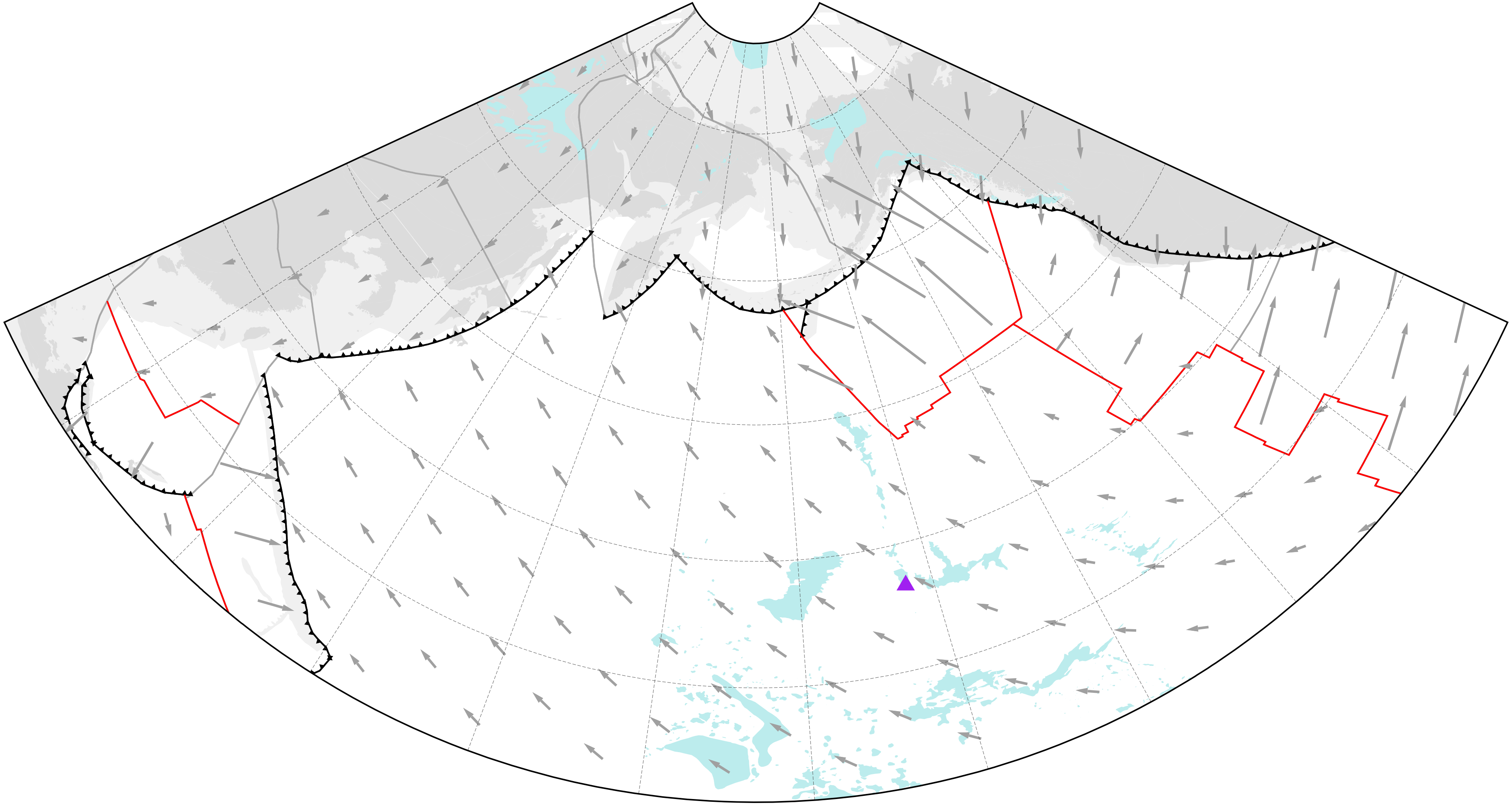

48 Ma

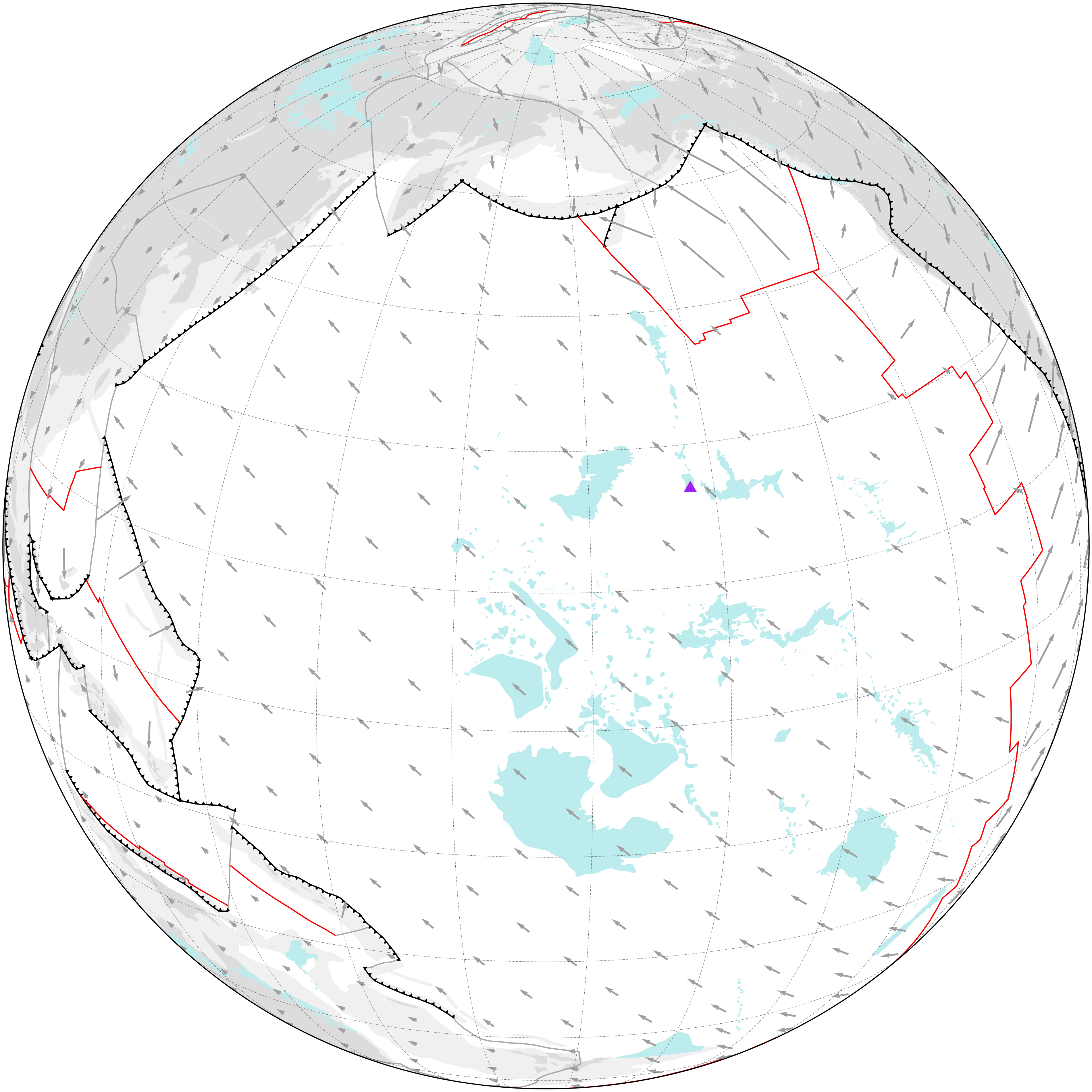

49 Ma

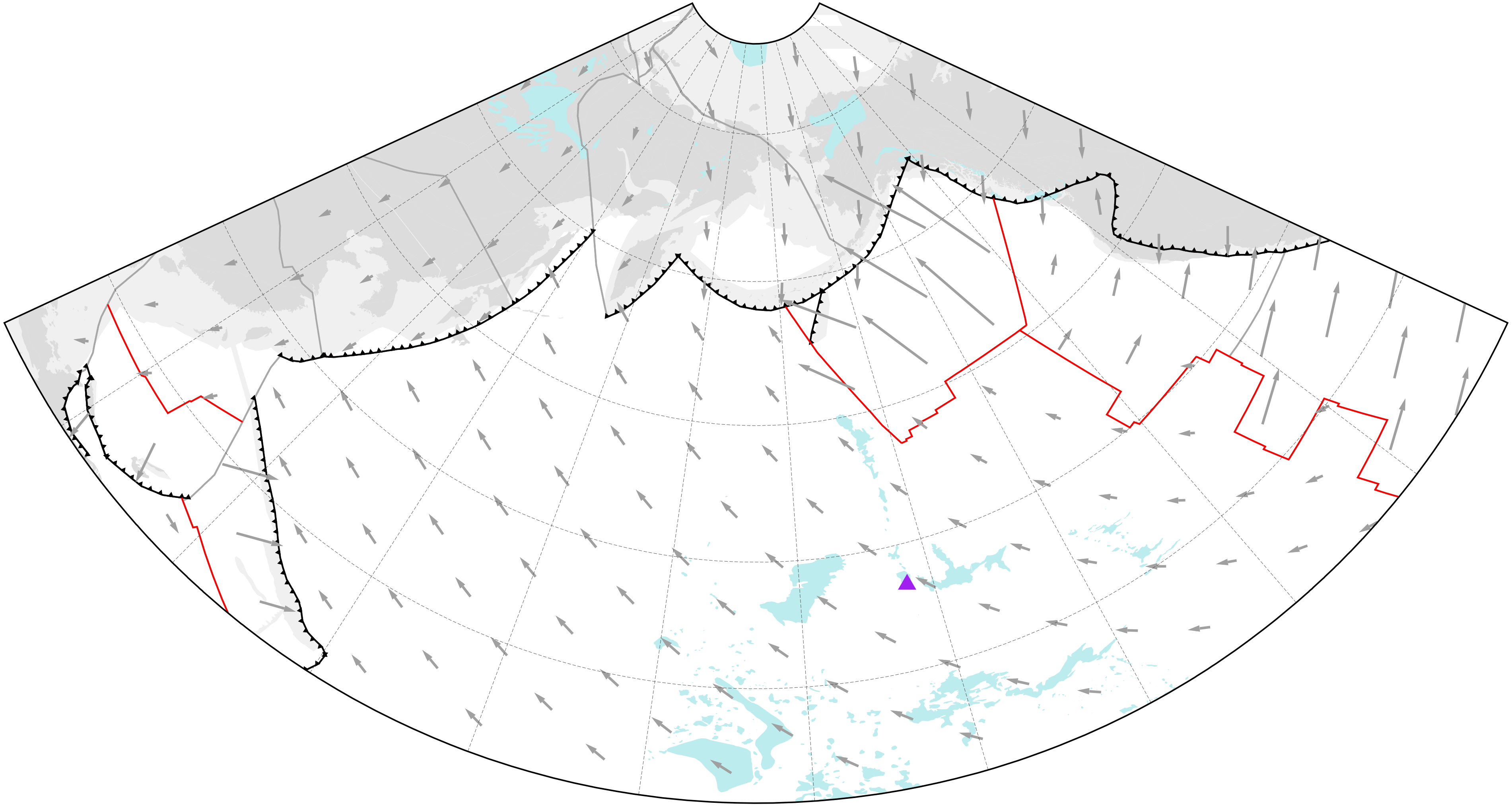

49 Ma

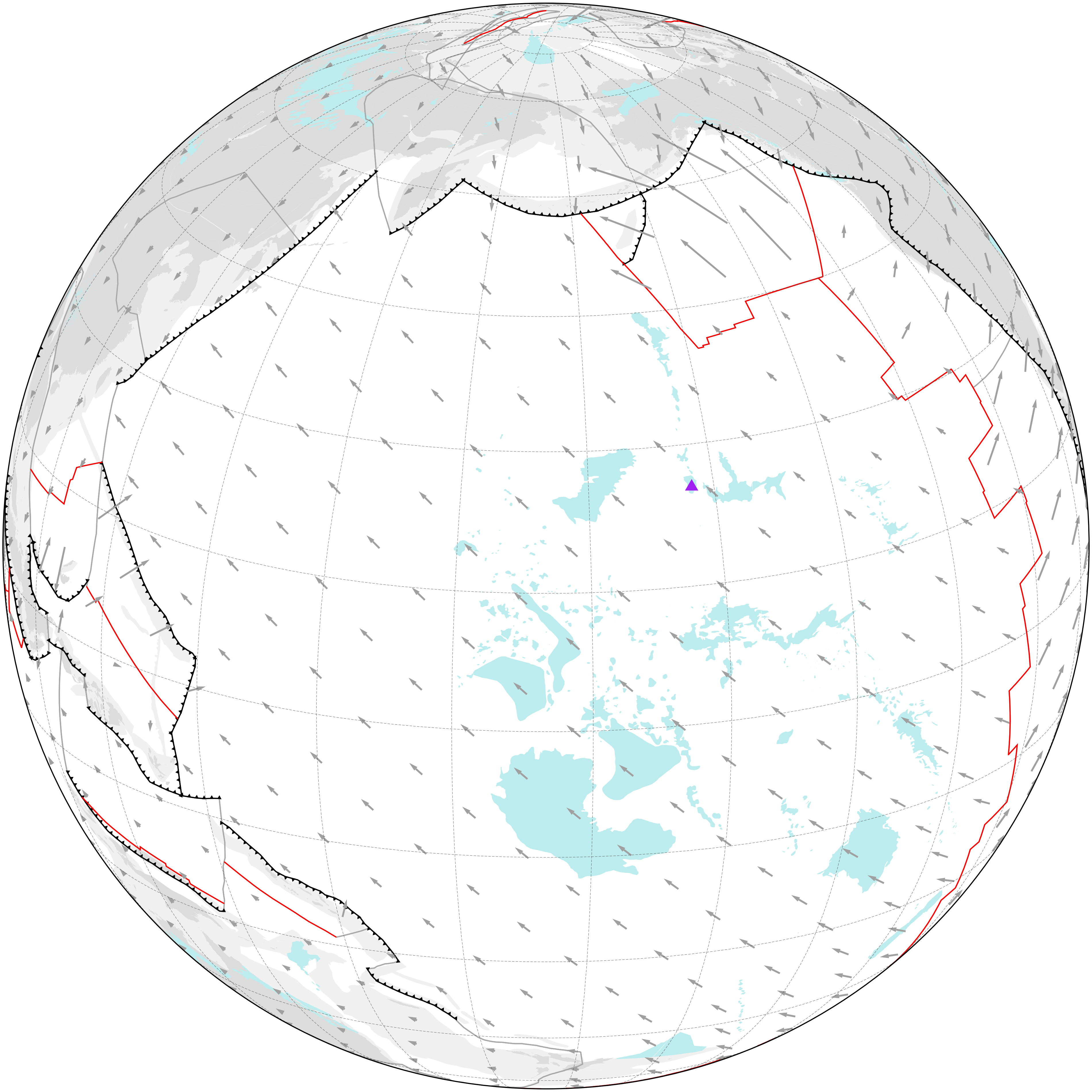

50 Ma

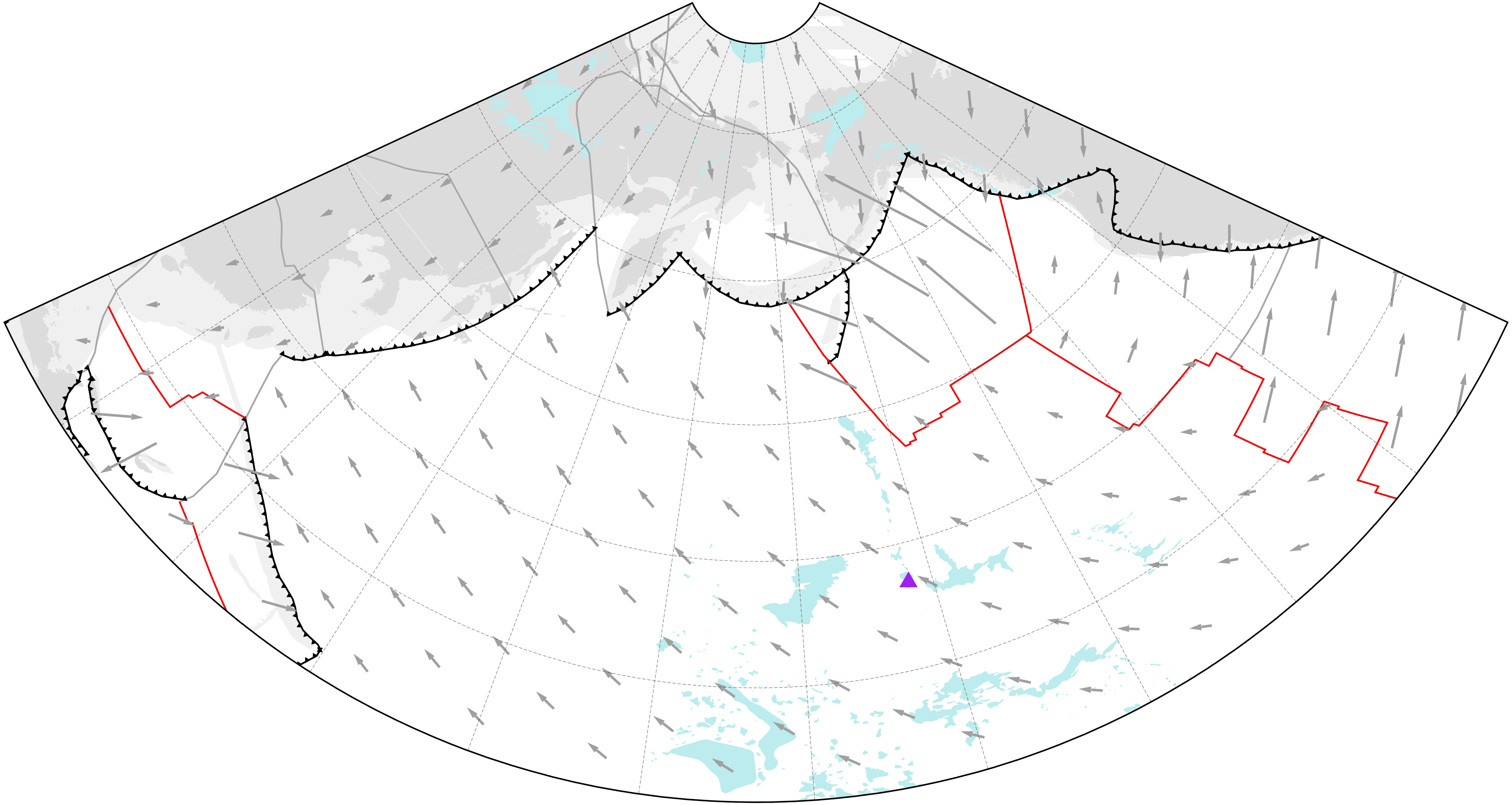

50 Ma

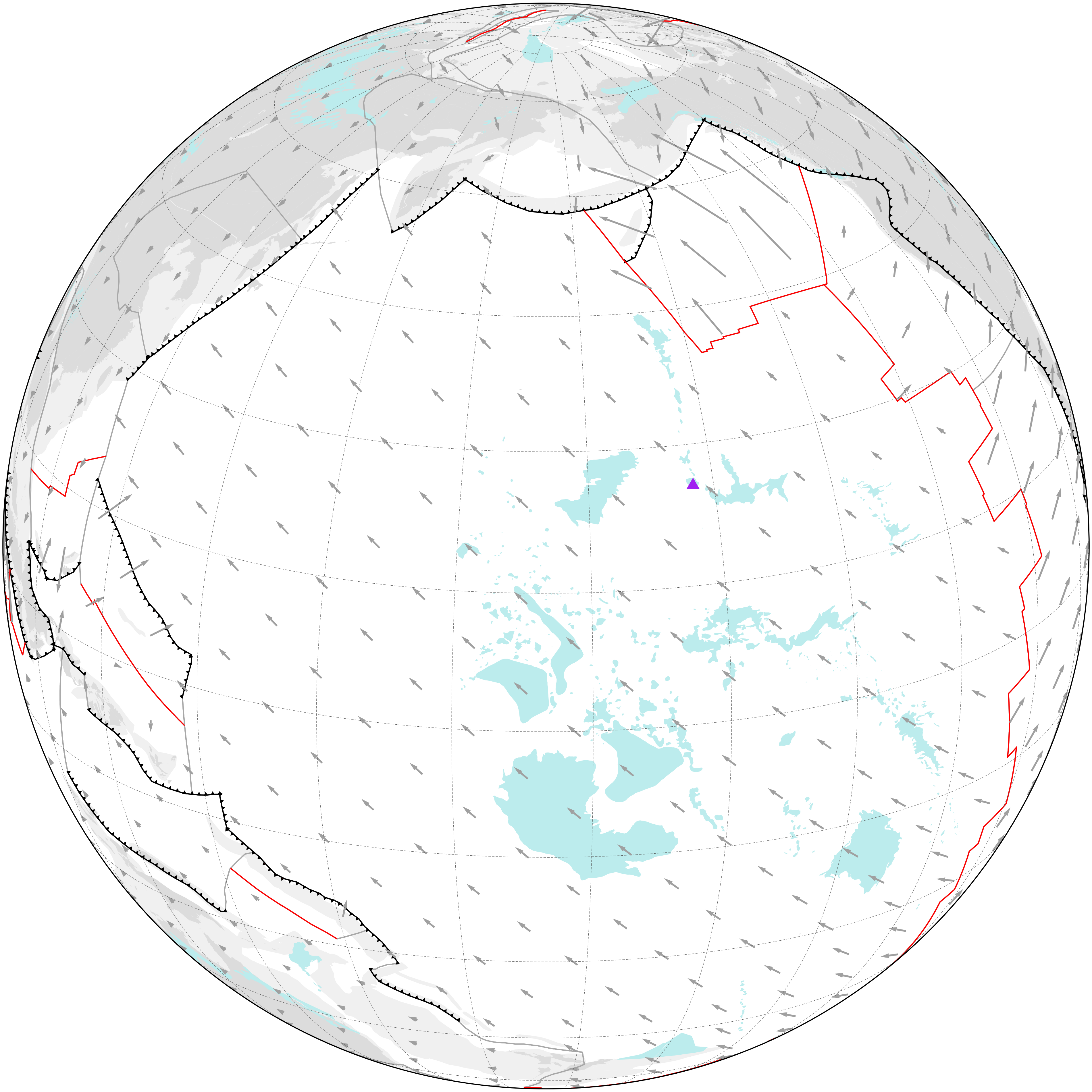

51 Ma

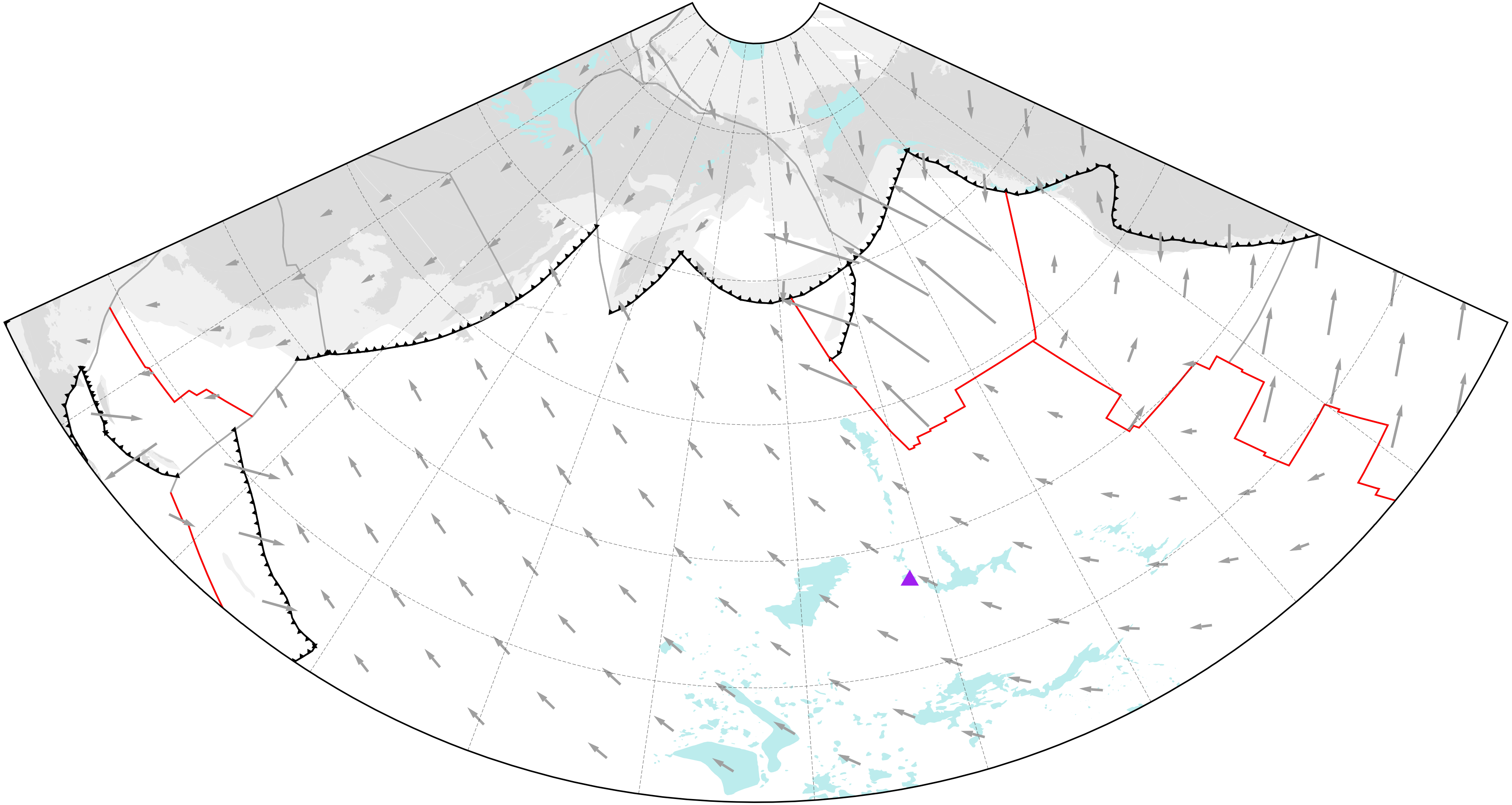

51 Ma

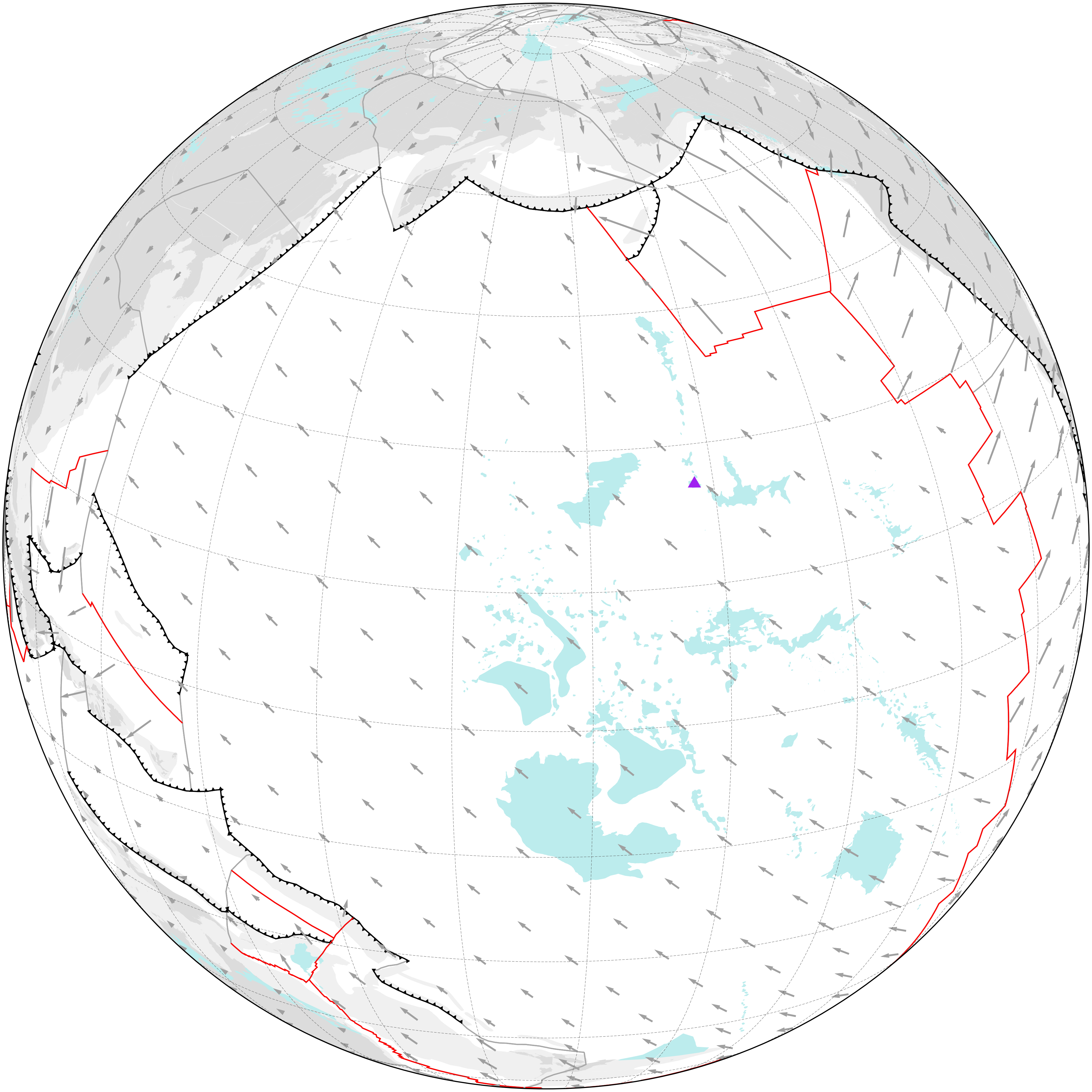

52 Ma

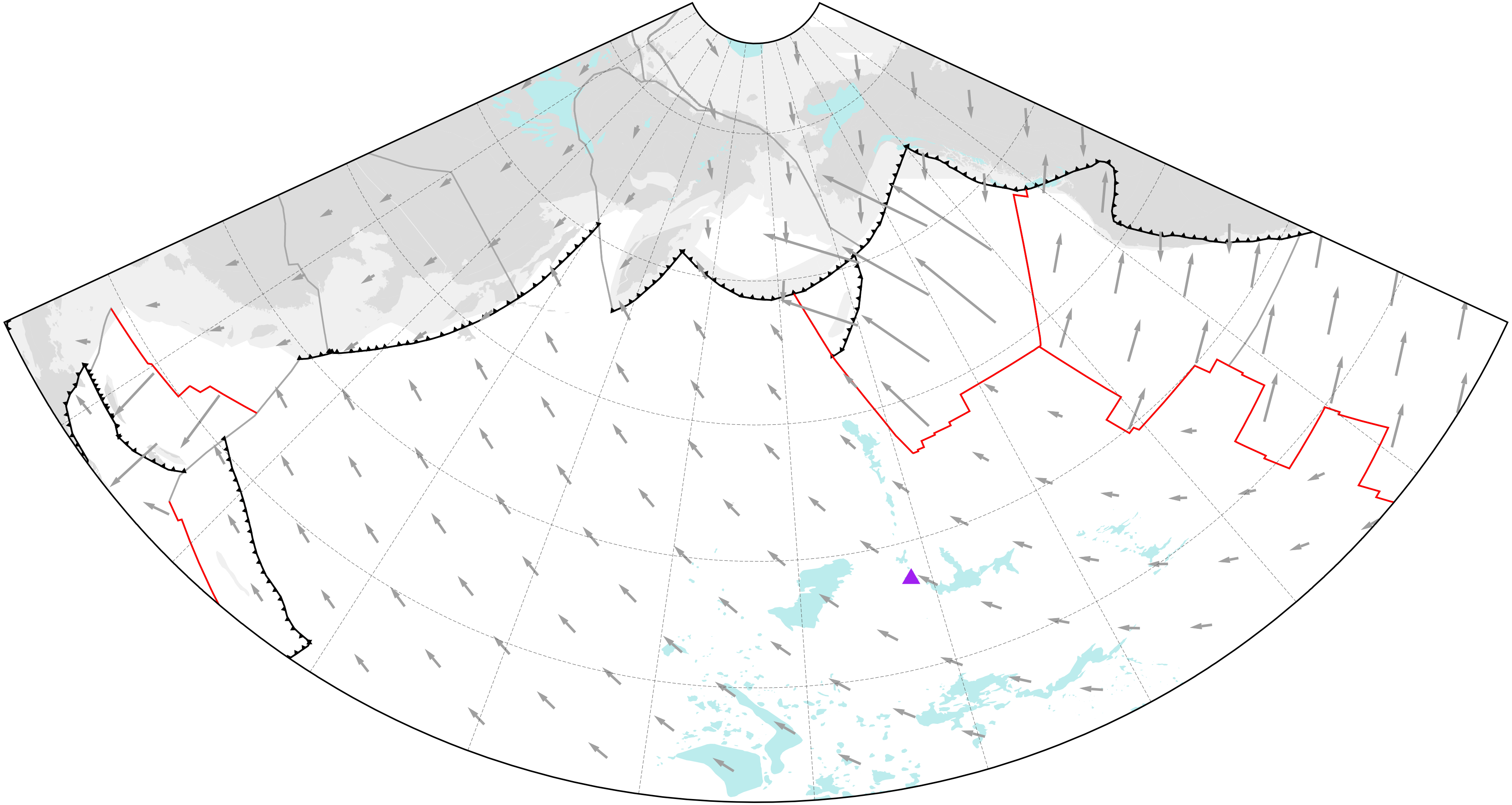

52 Ma

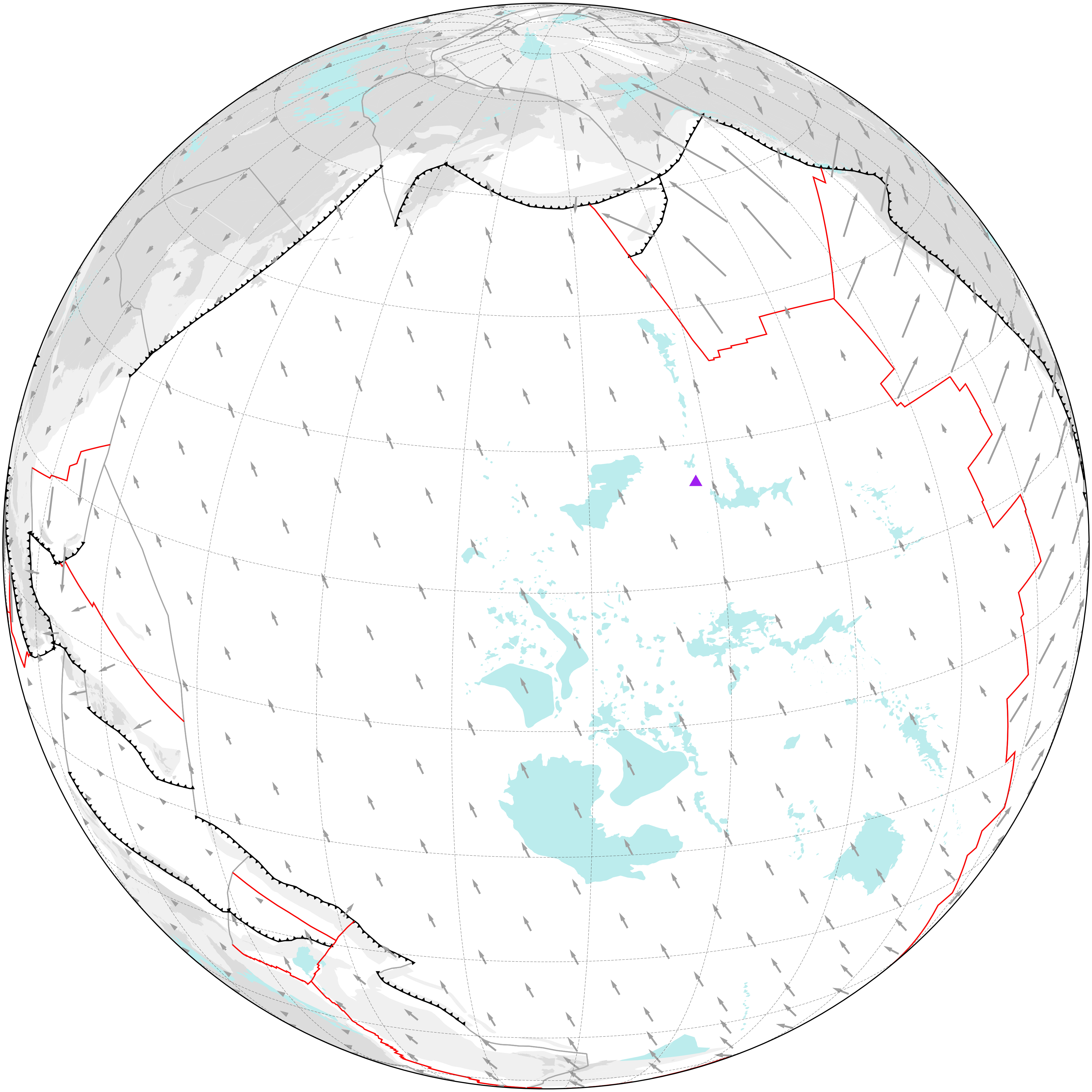

53 Ma

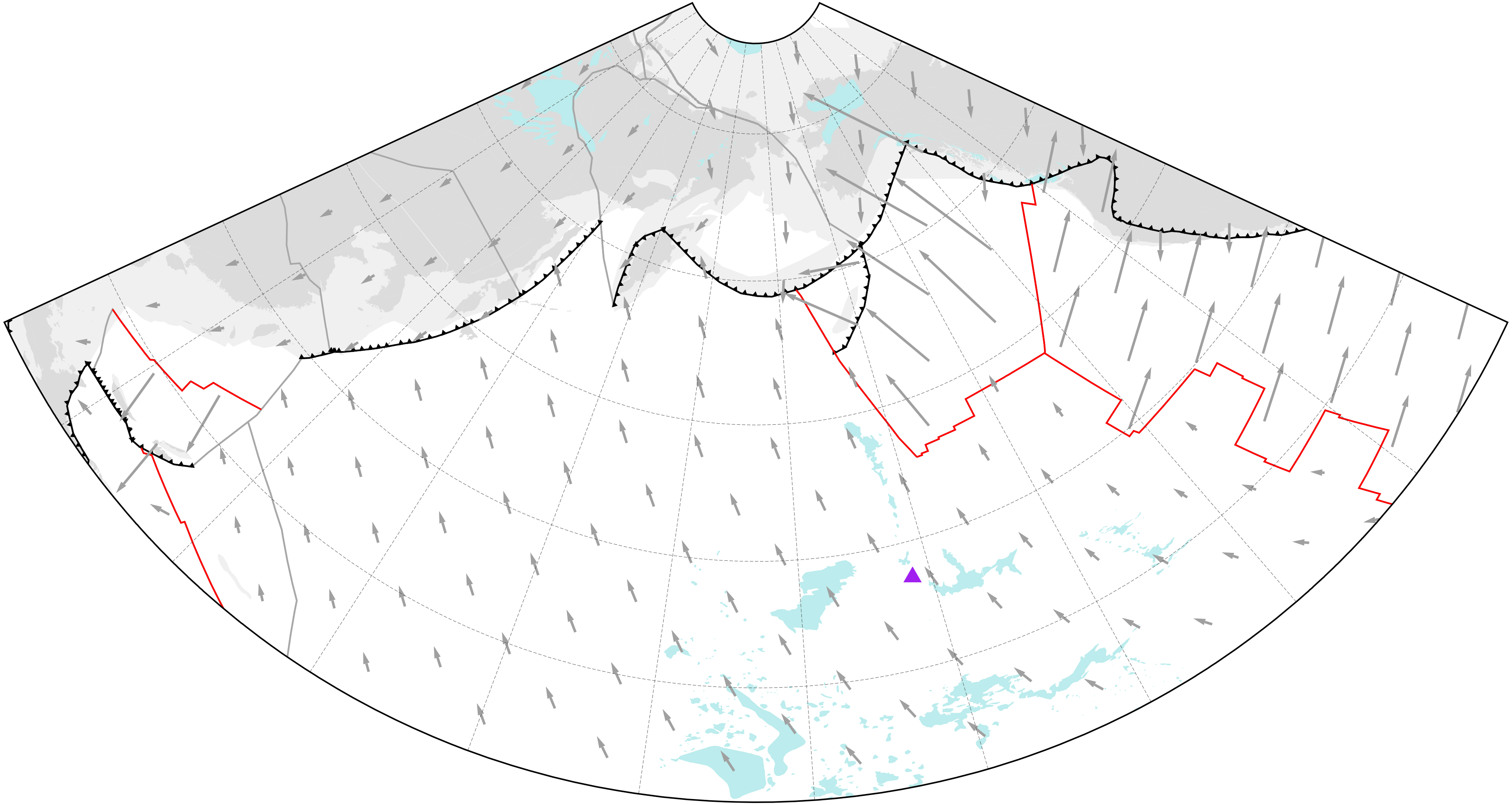

53 Ma

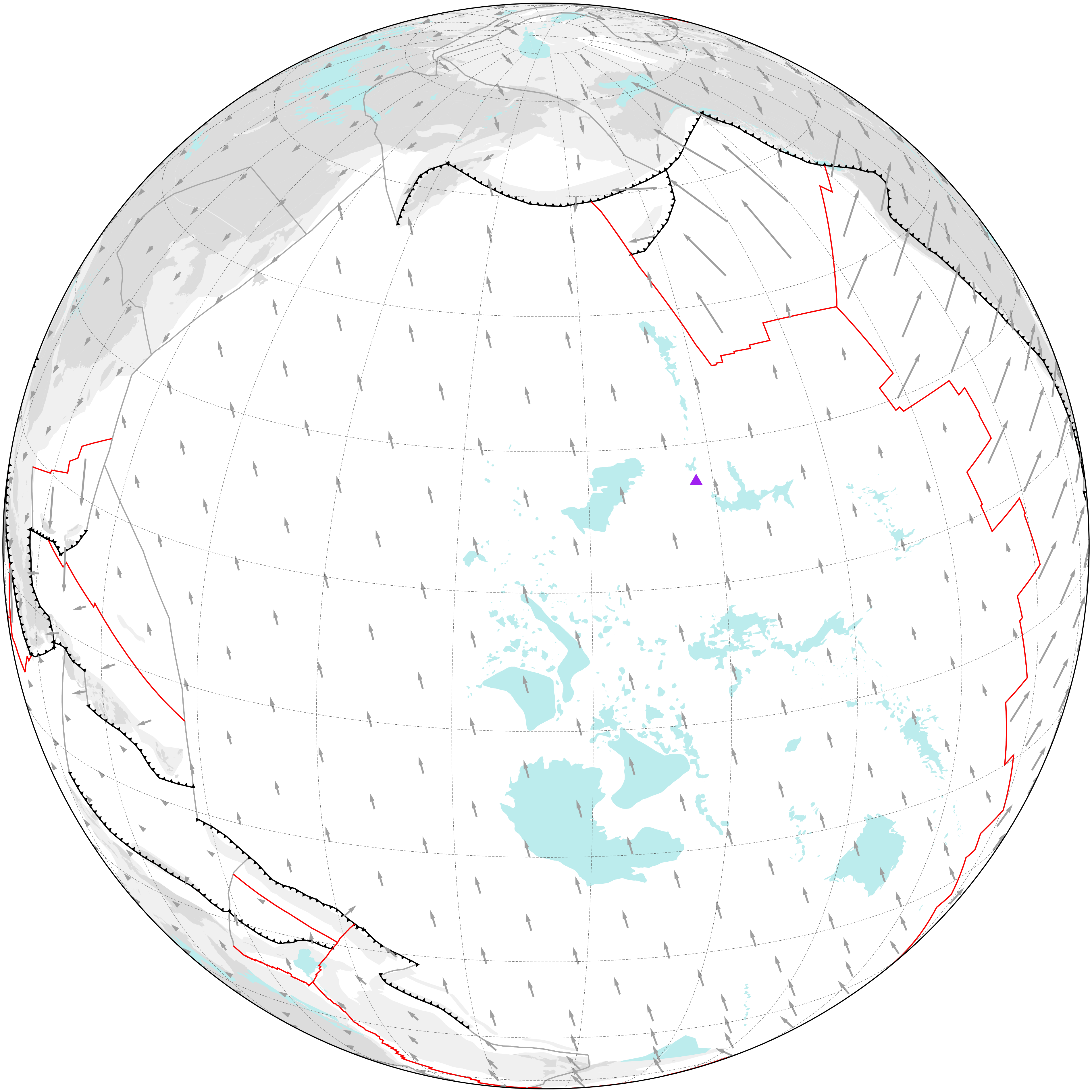

54 Ma

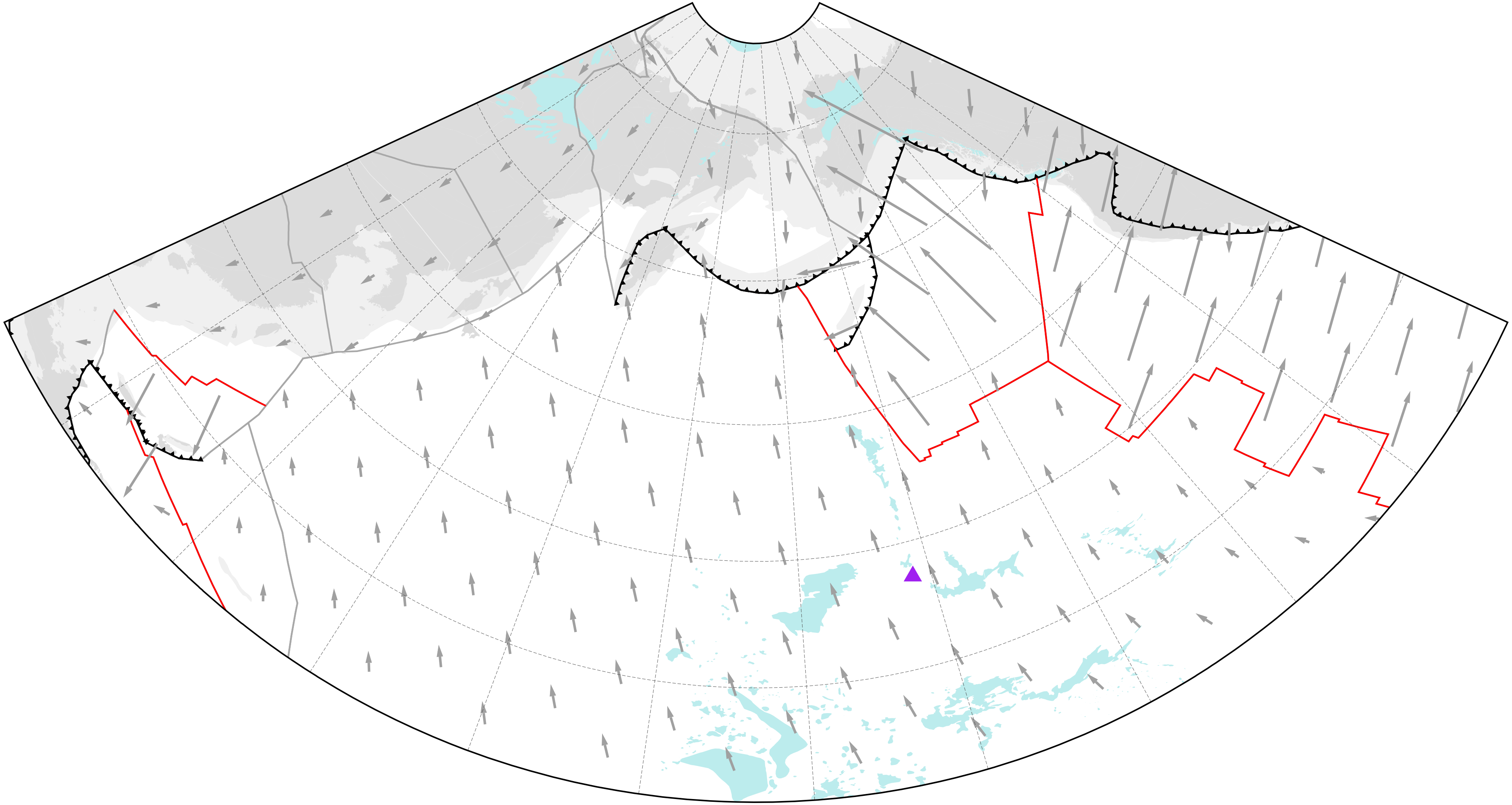

54 Ma

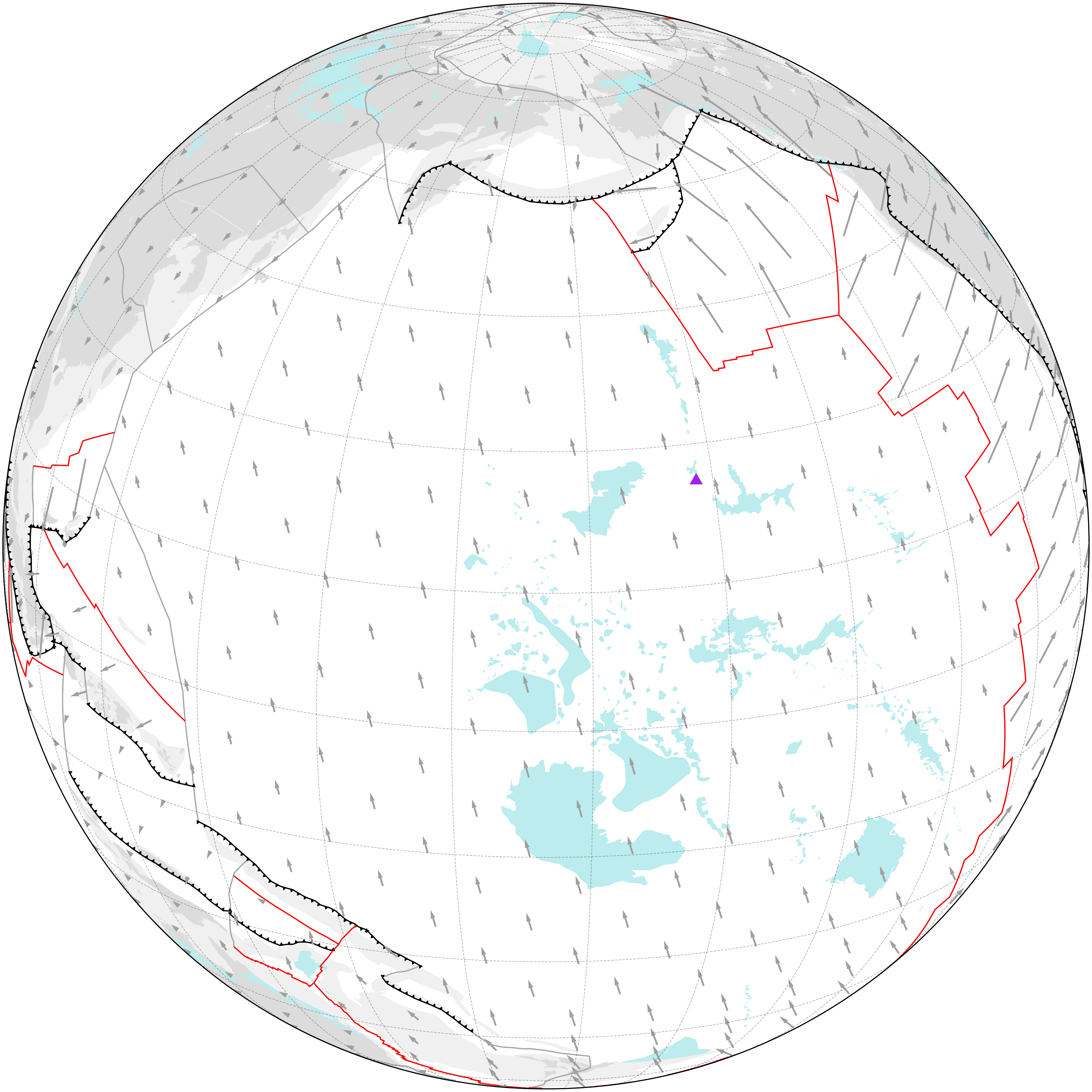

55 Ma

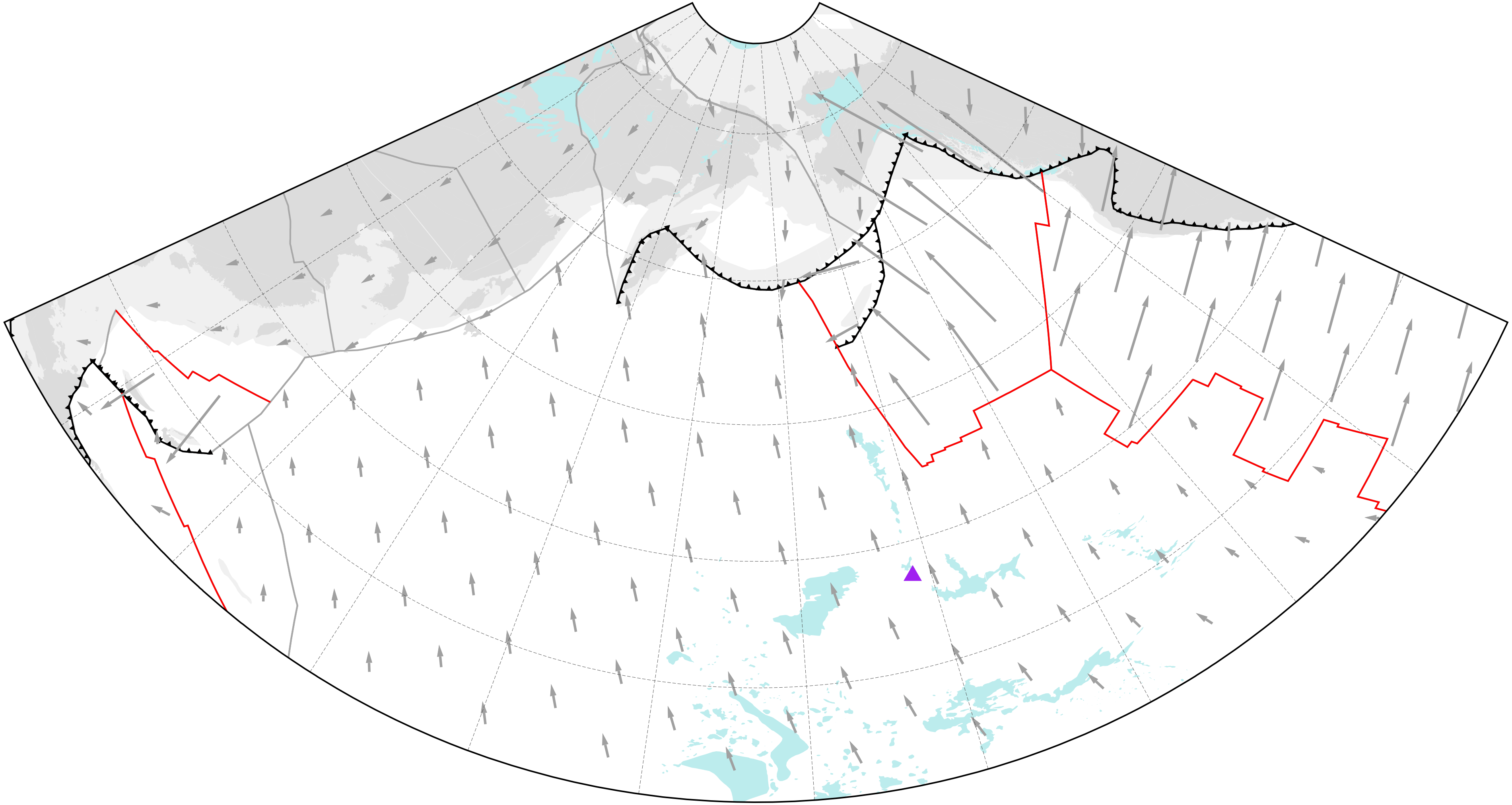

55 Ma

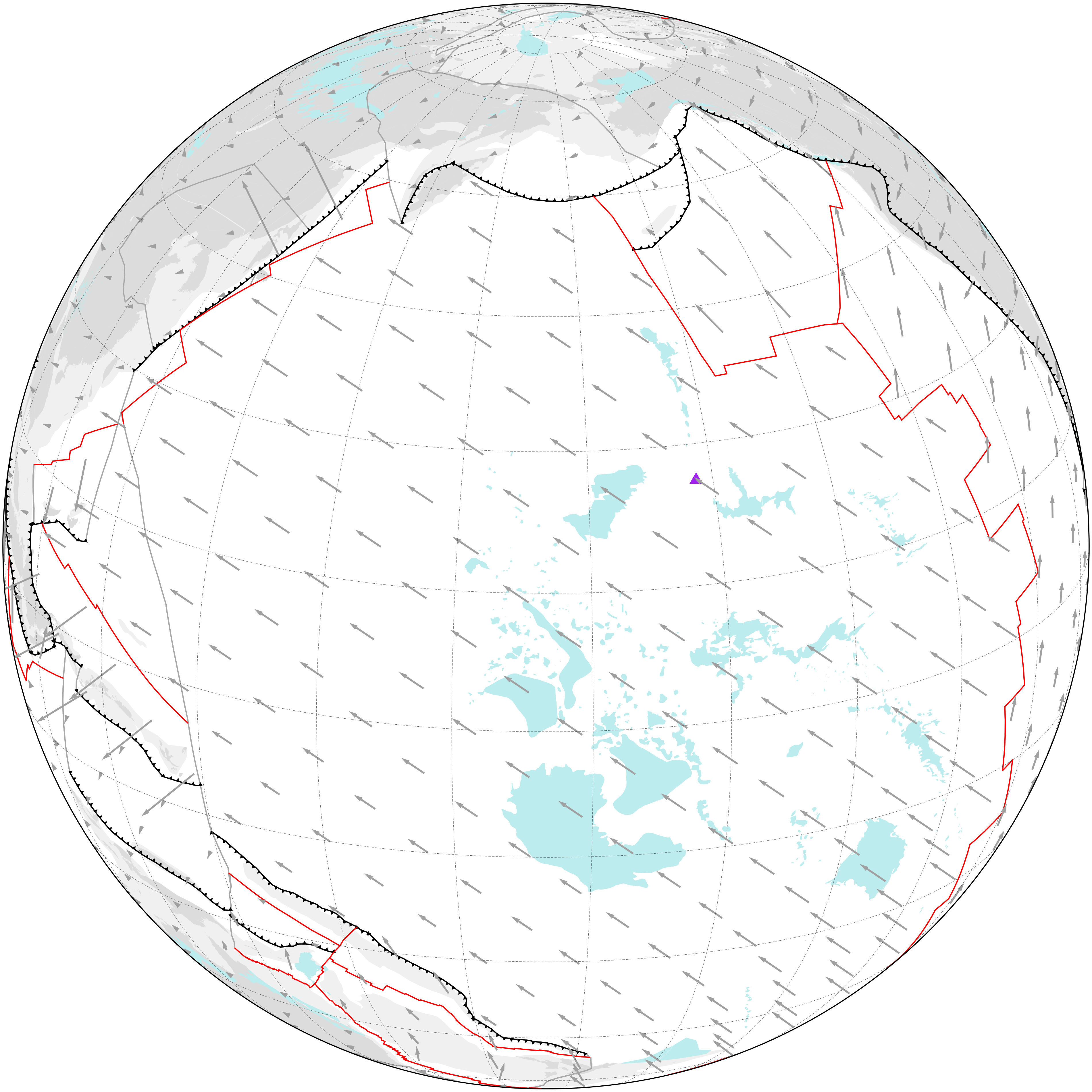

56 Ma

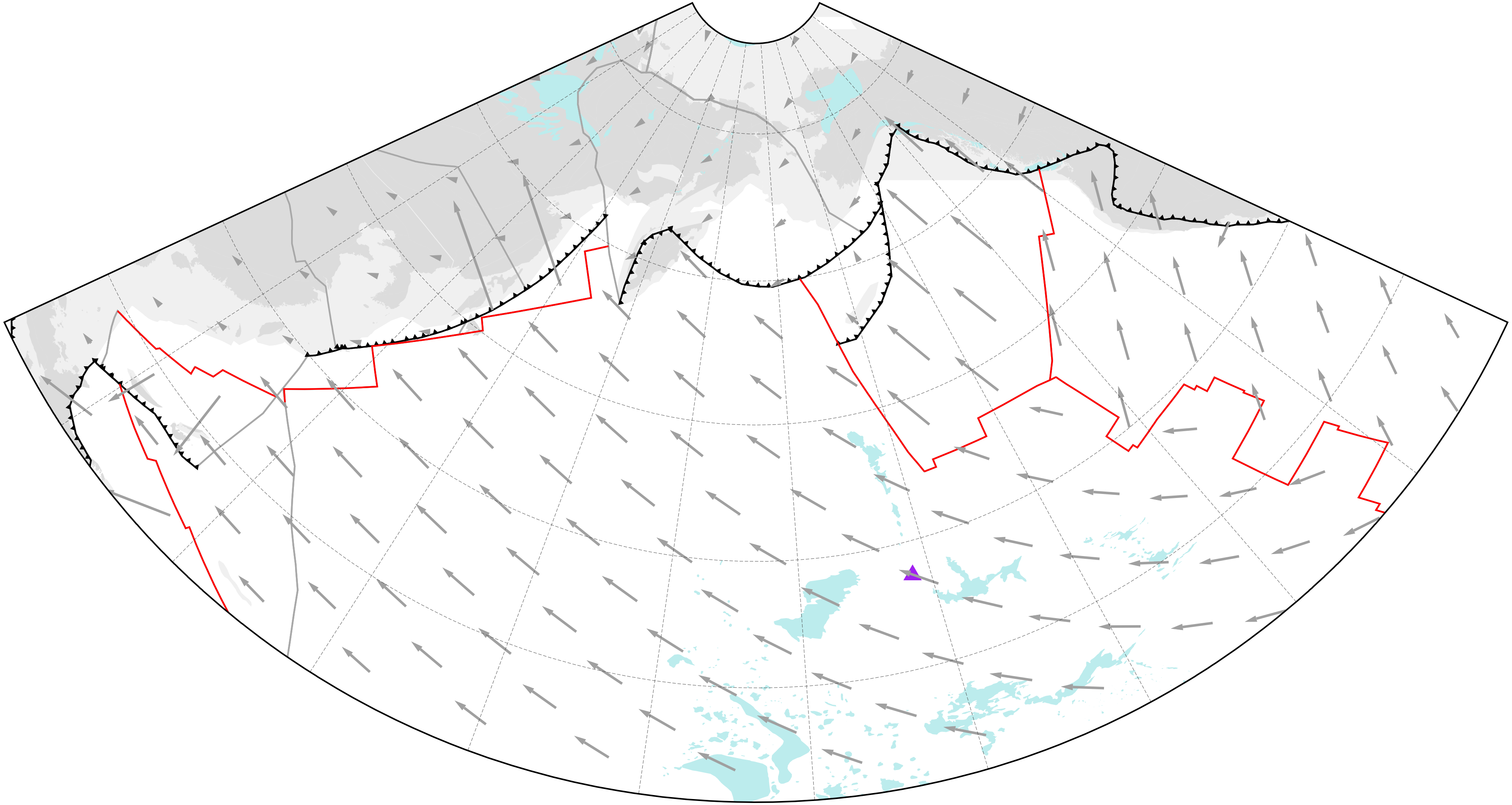

56 Ma

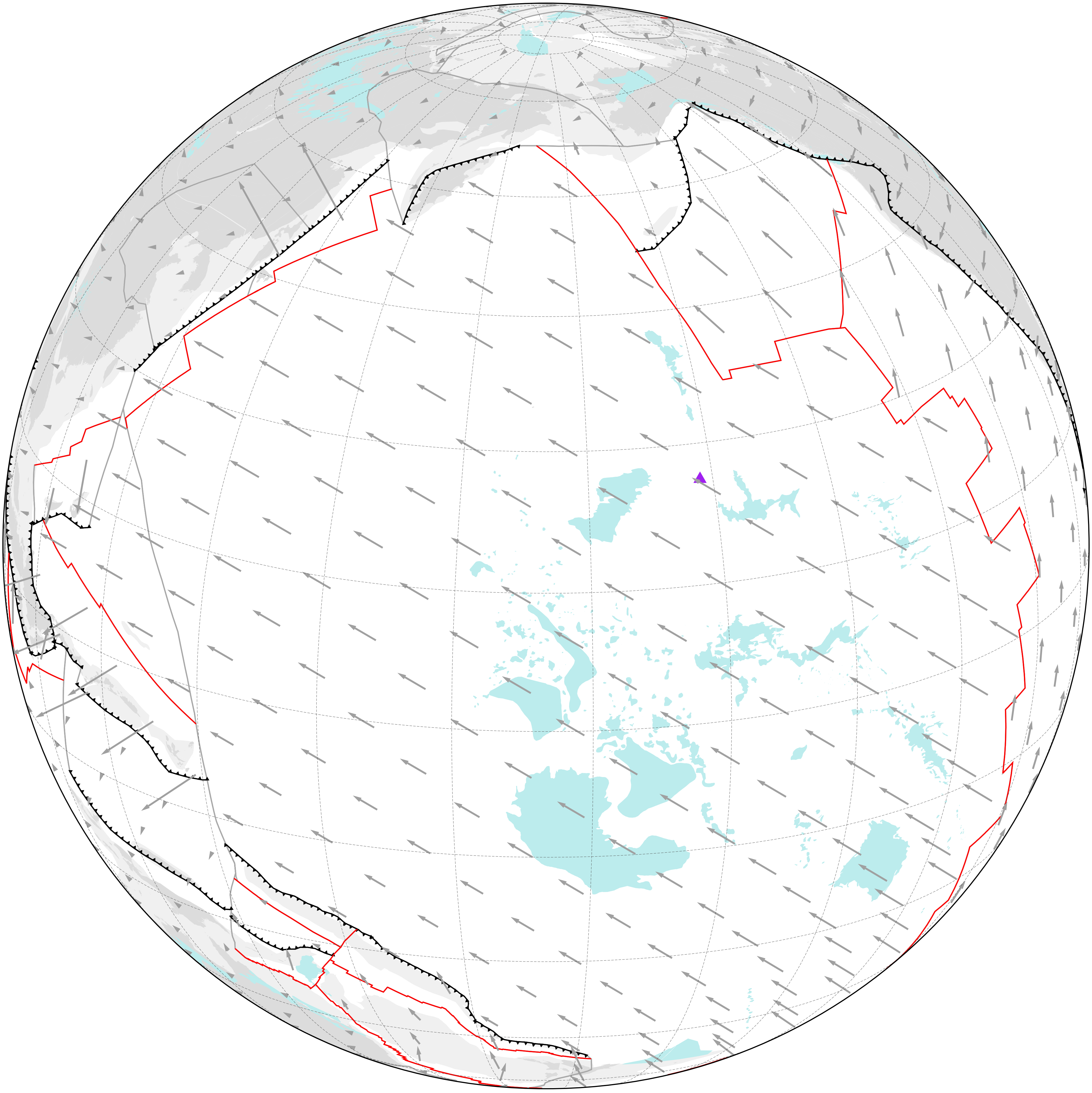

57 Ma

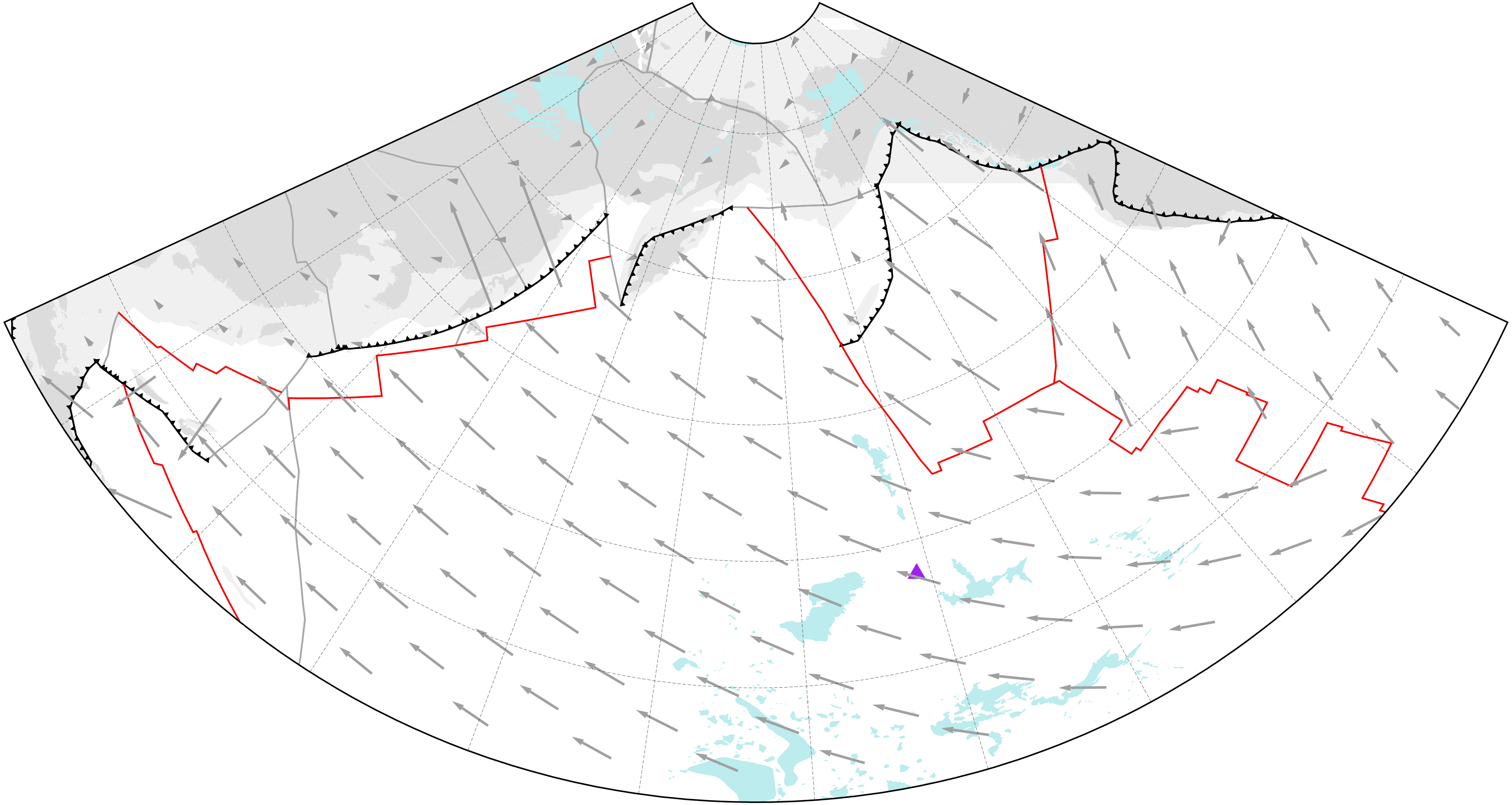

57 Ma

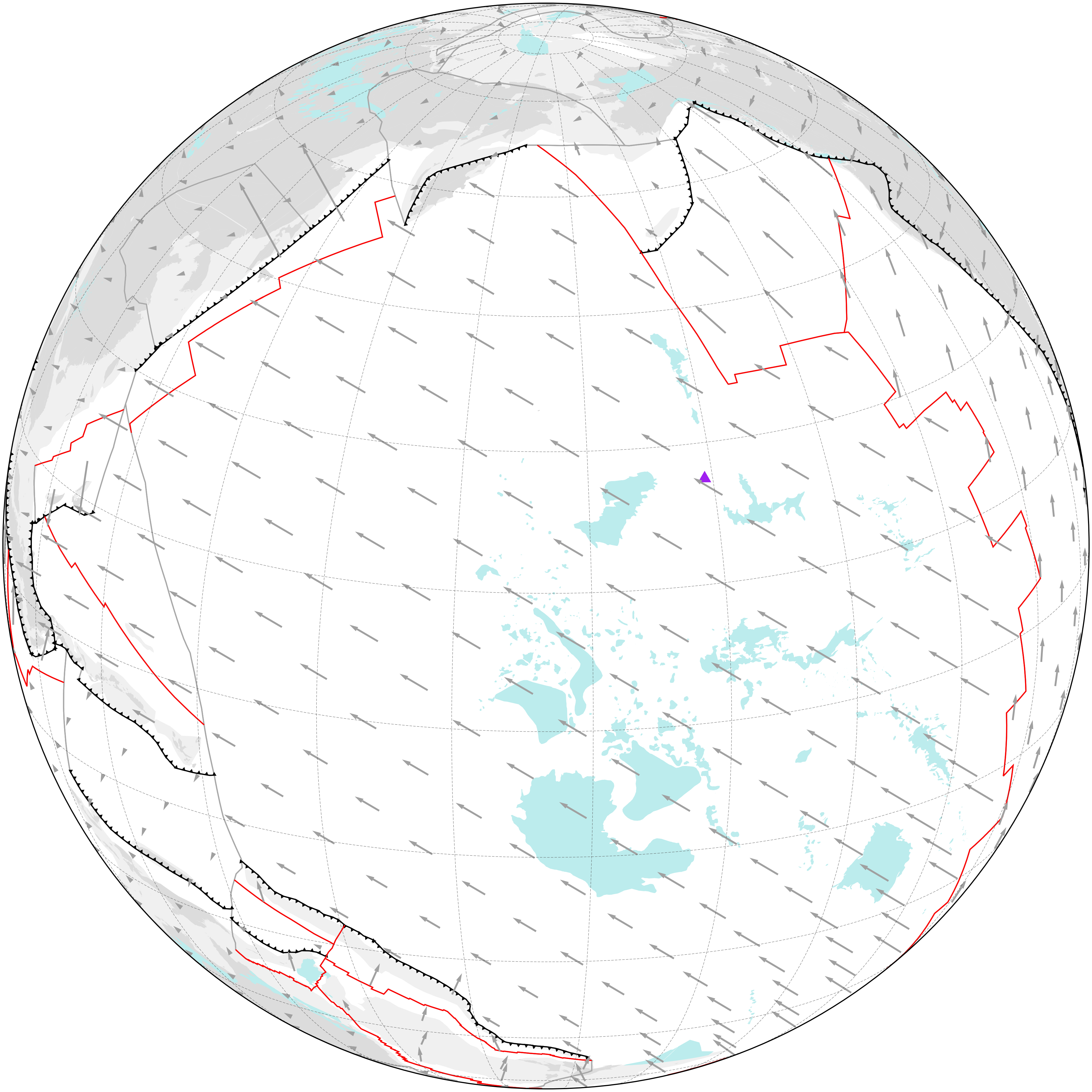

58 Ma

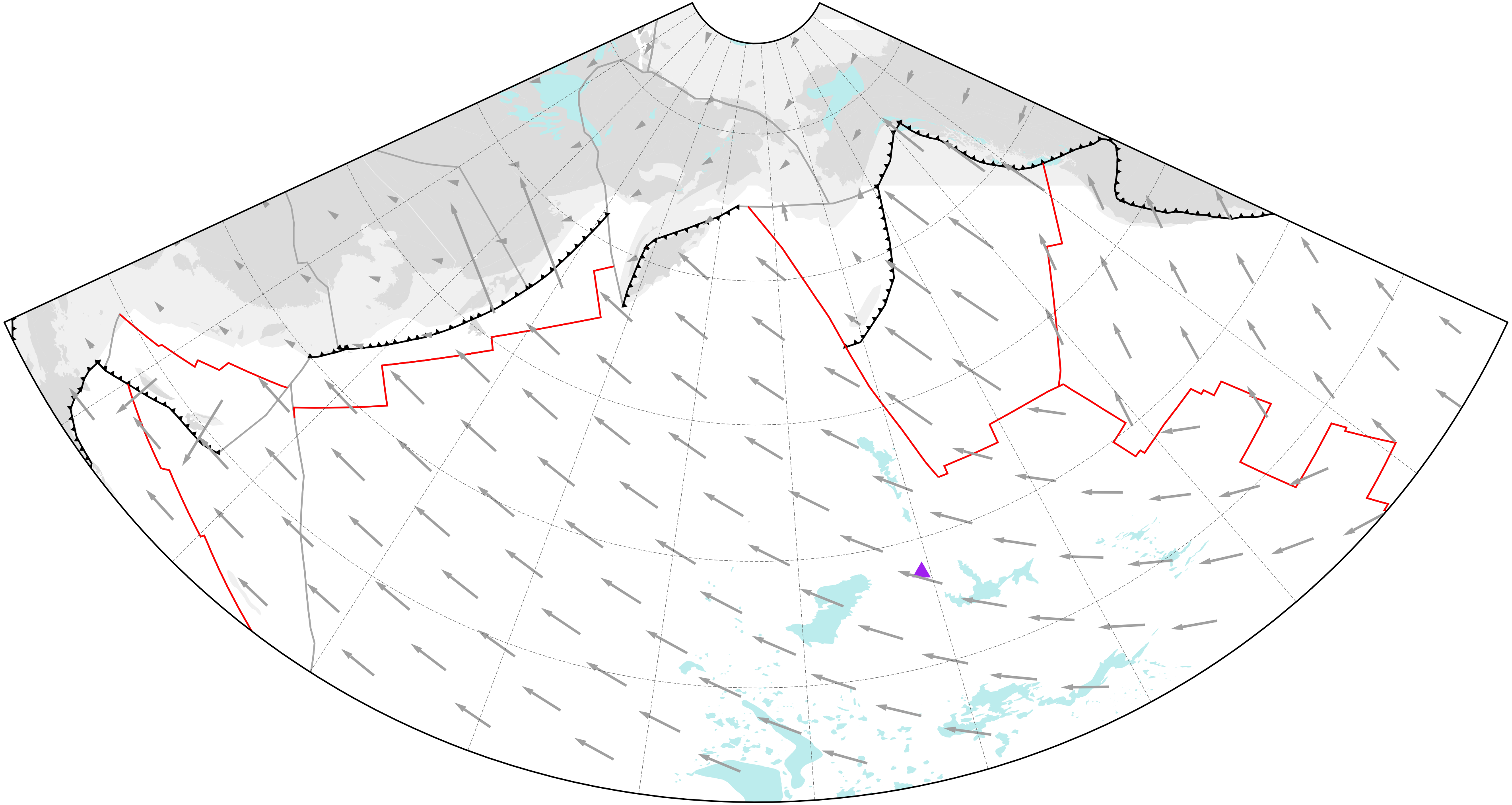

58 Ma

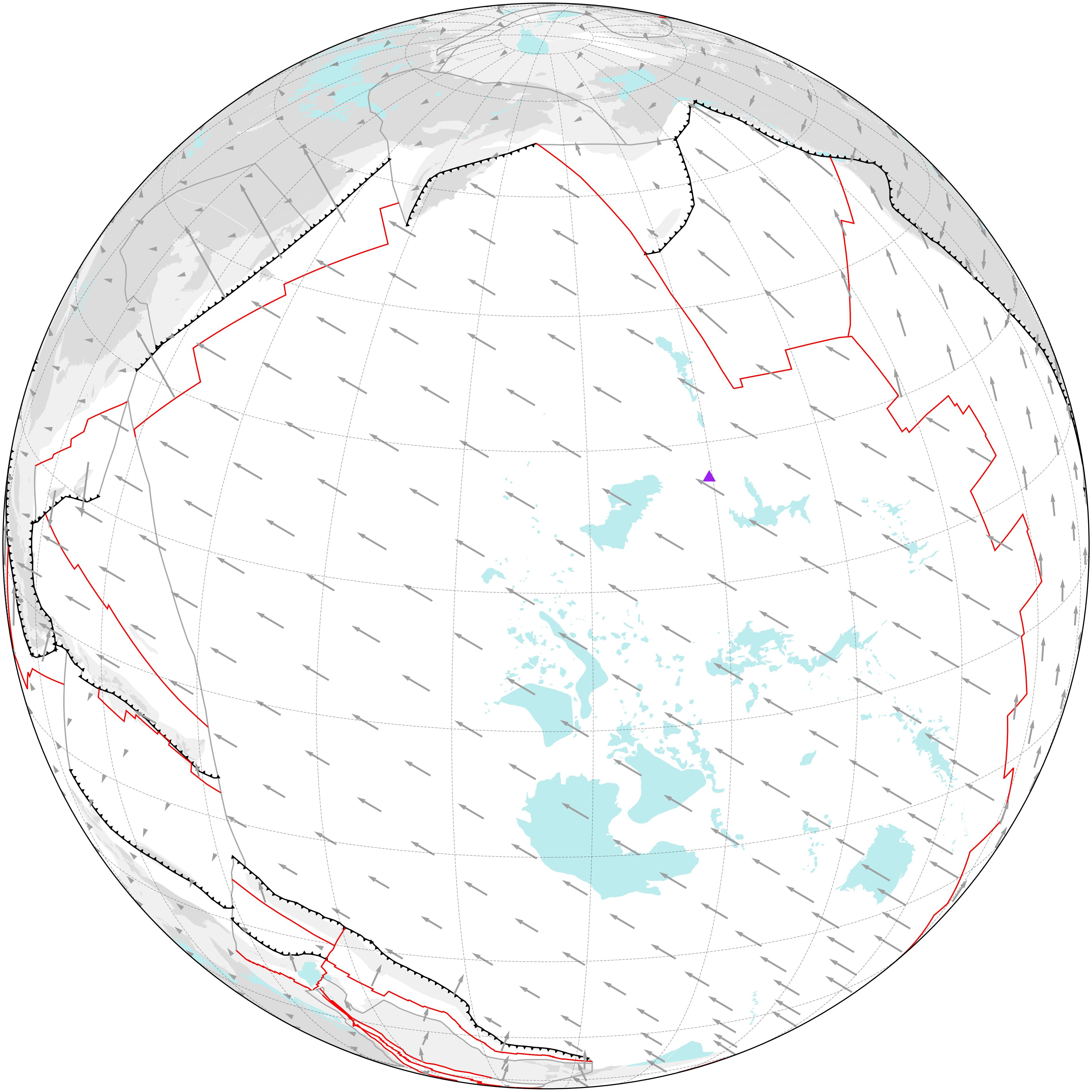

59 Ma

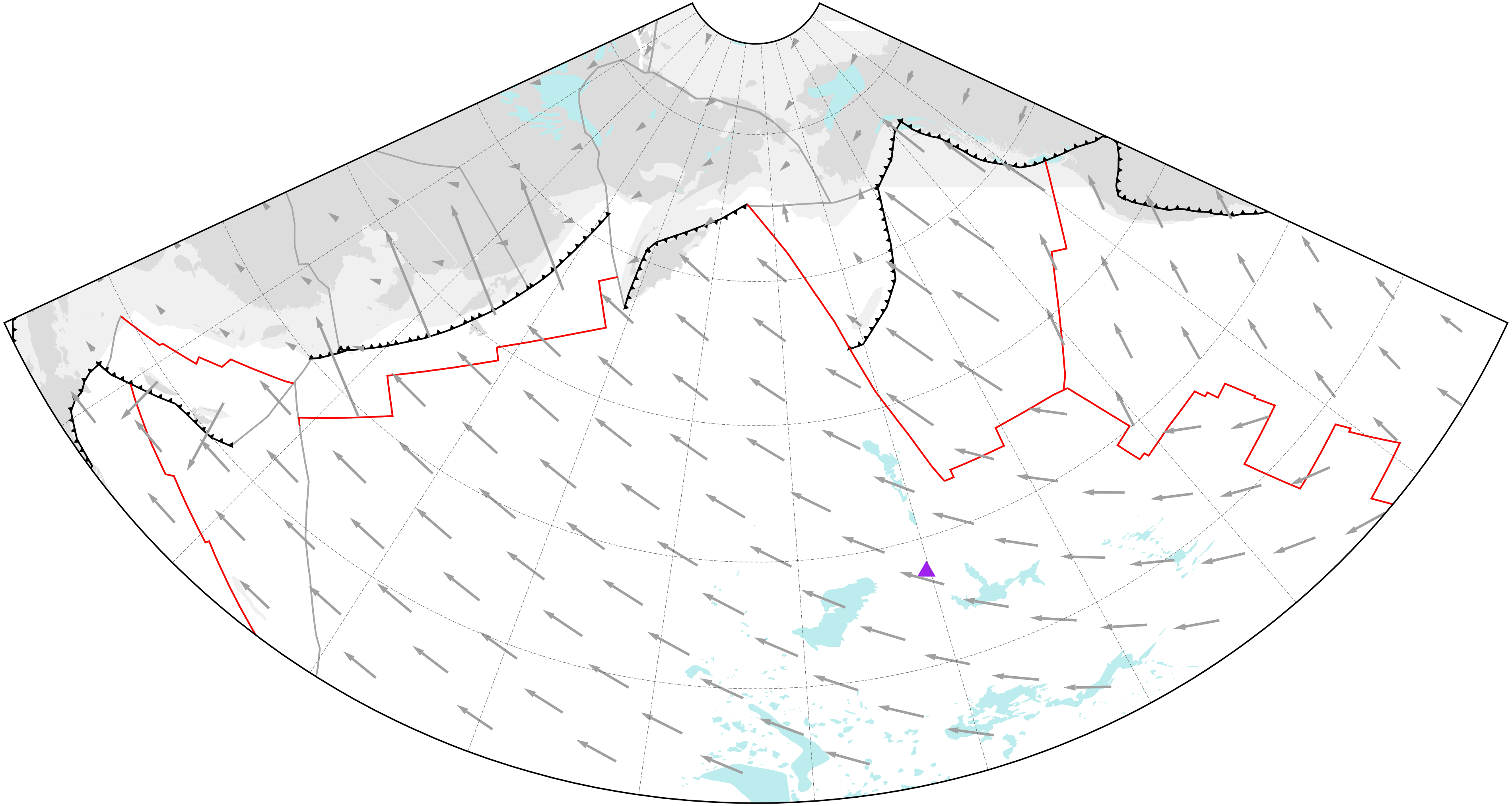

59 Ma

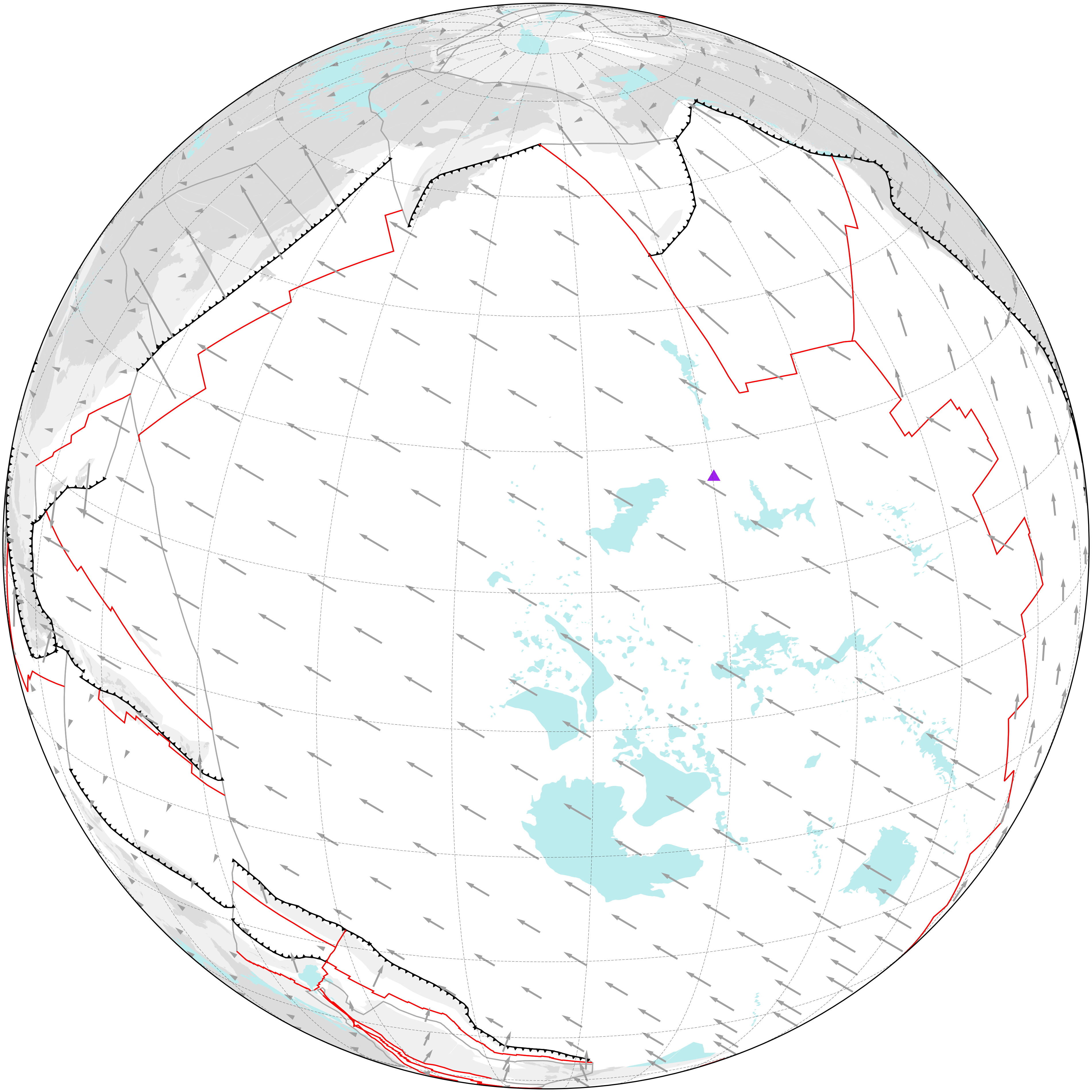

60 Ma

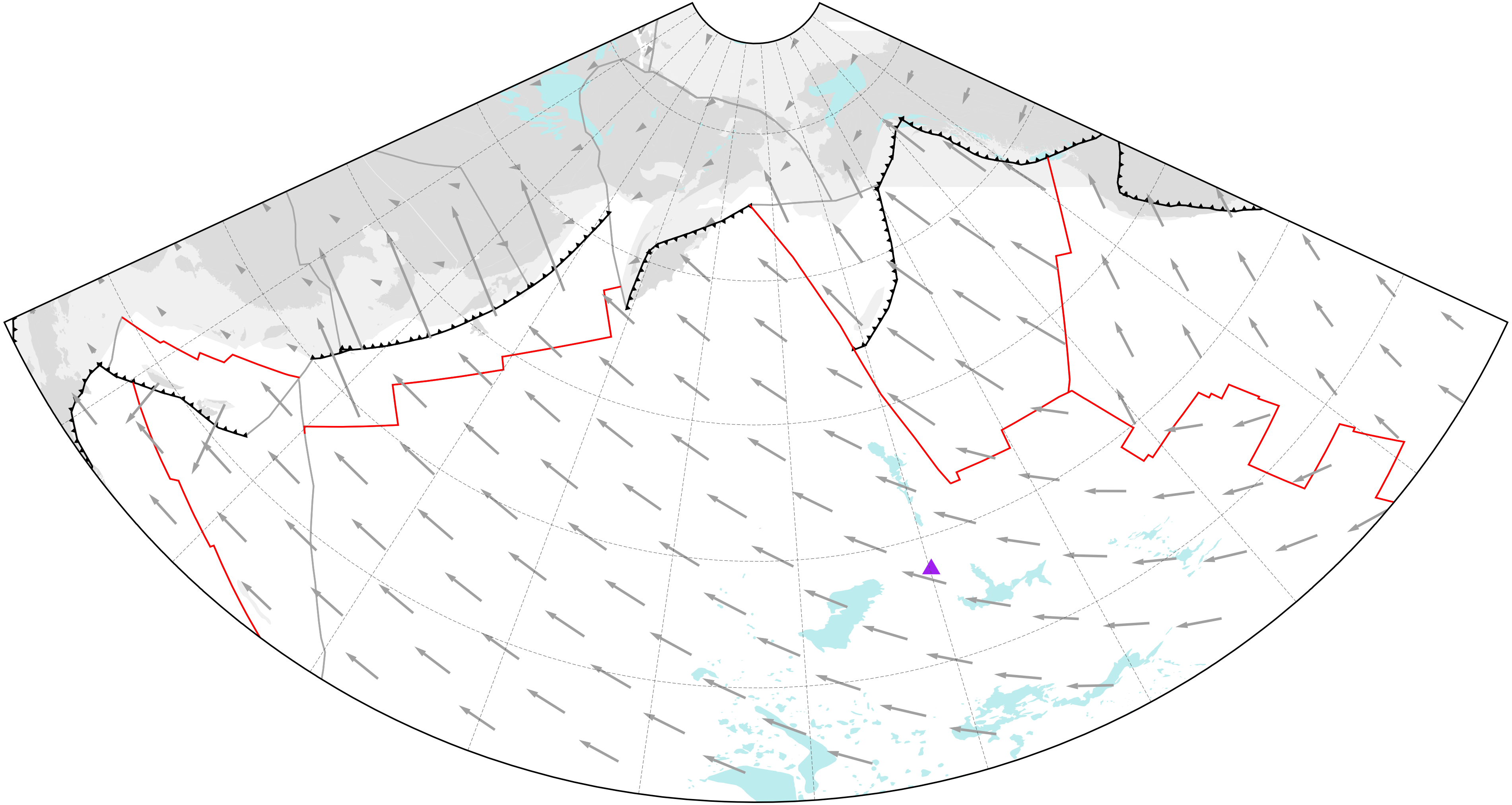

60 Ma

Supplementary Table 1: LA-ICP-MS U-(Th-)Pb Metadata

**Laboratory and Sample Preparation**	
Laboratory name	Dept of Earth Sciences, ETH Zurich
Sample type/mineral	magmatic zircons
Sample preparation	Conventional mineral separation, 1 inch resin mount, 1 μm polish to finish
**Laser ablation system**	
Make, Model and type	Applied Spectra (ASI, Resonetics) Resolution S155
Ablation cell and volume	Laurin Technic, 2 volume cell, effective volume *ca* . 1 cm^3
Laser wavelength (nm)	193 nm
Pulse width (ns)	25 ns
Fluence (J cm^{-2})	~2 J cm^{-2}
Repetition rate (Hz)	4 Hz
Ablation duration (s)	30 s
Ablation pit depth / ablation rate	~10 μm pit depth, equivalent to 0.08 μm/pulse
Spot diameter (μm) nominal/actual	19 μm / 19 μm
Sampling mode / pattern	Static spot ablation
Carrier gas	100% He in the cell, Ar make-up gas combined in cell above ablation in funnel.
Cell carrier gas flow (l min^{-1})	0.25 l min^{-1}
**ICP-MS Instrument**	
Make, Model and type	Thermo Element XR, Sector-field single collector ICP-MS
Sample introduction	direct
RF power (W)	1400 W - 1600W (optimized daily)
Make-up gas flow (l min^{-1})	0.90 - 1.05 l min^{-1} Ar (optimized daily)
Detection system	triple (pulse counting, analog, Faraday) cross calibrated daily, Si29, Zr91, Hf178, U238, Hf measured in analog only
Masses measured (amu)	27,29,31,49,89,91,93,137,139,140,141,146,147,153,157,159,163,165,166,169,172,175,178,202,204,206,207,208,232,235,238
Integration time per peak/dwell times (ms)	11 ms (masses 27, 29, 31, 89, 91, 93,137, 139, 140, 141, 146, 147, 153, 157, 159, 163, 165, 166, 172, 175, 178, 181, 202, 204, 208, 232, 235, 238), 25 ms (49), 50 ms (206, 207)
Total integration time per output data point (s)	0.648 s
Dead time (ns)	13
Typical oxide rate (ThO/Th)	0.18%
Typical doubly charged rate (Ba^{++}/Ba^{+})	3.50%
**Data Processing**	
Gas blank	20 s
Calibration strategy	GJ-1 used as primary reference material for dating, NIST 610 as primary RM for trace elements, 91500 Primary reference material for Ti conc. Plešovice, 91500, AUSZ7-1 and AUSZ7-5, GHR-1 used as secondaries for validation
Reference Material info	GJ-1 (Jackson *et al* . 2004), 600.5 Ma<br>Plešovice (Slama *et al* . 2008) 337 Ma<br>GHR-1 (Eddy *et al.* 2018), 48.1 Ma<br>91500 (Wiedenbeck et al. 1995), 1065 Ma<br>AUSZ7-1 (similar to Kennedy 2014), 38.9 Ma<br>AUSZ7-5 (VonQuadt et al. 2016), 2.4 Ma<br>NIST 610 (Jochum et al. 2016)
Data processing package used / Correction for LIEF	Iolite 4.8. LIEF correction assumes reference material and samples behave identically.
Mass discrimination	normalised to reference material
Common-Pb correction, composition and uncertainty	No common-Pb correction applied to the data.
Th disequilibrium	not corrected
Uncertainty level and propagation	Ages are quoted at 2*s* absolute, propagation is by quadratic addition. Reproducibility and age uncertainty of reference material are propagated where appropriate.
Quality control / Validation	Plešovice – Wtd ave $^{206}Pb/^{238}U$ age = 339.49 ± 5.44 (2s, MSWD = 0.5, n = 12) (0.57% Wtd ave uncert. (internal), 1.5% Total external uncert.)
	GHR-1 - Wtd ave 206Pb/238U age = 48.09 ± 1.08 (2s, MSWD = 4.6, n = 13) (0.71% Wtd ave uncert. (internal), 1.5% total external uncert.) with overdisperion
	91500 – Wtd ave $^{206}Pb/^{238}U$ age = 1066 ± 16 (2*s* , MSWD = 1.4, n = 16) (0.51% Wtd ave uncert. (internal), 1.5% Total external uncert.)
	AUSZ7-1 – Wtd ave $^{206}Pb/^{238}U$ age = 38.5 ± 0.6 (2*s* , MSWD = 1.4, n = 18) (0.73% Wtd ave uncert. (internal), 1.5% Total external uncert.)
	AUSZ7-5 – Wtd ave $^{206}Pb/^{238}U$ age = 2.41 ± 0.055 (2*s* , MSWD = 1.5, n = 18) (2.37 % Wtd ave uncert. (internal), 1.5% Total external uncert.)
	Systematic uncertainty for propagation is 1.5% (2*s*).

This reporting is based on a template available for download on www.Plasmage.org

Supplementary Table 1. U-Pb LA-ICP-MS data on samples and standards

	Date	Duration (s)	Spot Size (µm)	Final Pb206/U238_mean	Final Pb206/U238_2SE(prop)	Final Pb206/U238 age_mean	Final Pb206/U238 age_2SE(prop)	Final Pb207/U235_mean	Final Pb207/U235_2SE(prop)	Final Pb207/U235 age_mean	Final Pb207/U235 age_2SE(prop)	Final Pb208/Th232_mean	Final Pb208/Th232_2SE(prop)	Final Pb208/Th232 age_mean	Final Pb208/Th232 age_2SE(prop)	Final Pb207/Pb206_mean	Final Pb207/Pb206_2SE(prop)	Final Pb207/Pb206 age_mean	Final Pb207/Pb206 age_2SE(prop)	Final U238/Pb206_mean	Final U238/Pb206_2SE(prop)	Final U/Th_mean	Final Pb207/U235_mean	Final Pb207/U235_2SE(prop)	Final Pb206/U238_mean	Final Pb206/U238_2SE(prop)	rho 206Pb/238U v 207Pb/235U	rho 207Pb/206Pb v 238U/206Pb
Z_91500	6/8/2023																											
91500 - 1		27.01	19	0.177452	0.003923	1052.901	21.47558	1.824193	0.053796	1053.21	19.33126	0.05073	0.00342	1000.014	65.65228	0.074467	0.001686	1047.586	45.34736	5.640299	0.125006	2.372873	1.824193	0.053796	0.177452	0.003923	0.38755	0.269452
91500 - 2		27.00	19	0.180856	0.00397	1071.514	21.68391	1.859369	0.054951	1065.782	19.49472	0.052075	0.00359	1025.816	68.92515	0.074693	0.001716	1053.593	46.2221	5.53672	0.12281	2.399772	1.859369	0.054951	0.180856	0.00397	0.349985	0.286039
91500 - 4		9.79	19	0.182724	0.00478	1081.737	26.06442	1.896794	0.062603	1079.456	21.85854	0.054954	0.00374	1081.207	71.62311	0.075339	0.002013	1073.819	53.24963	5.474399	0.144406	2.456252	1.896794	0.062603	0.182724	0.00478	0.438261	0.366069
91500 - 5		28.00	19	0.183574	0.004541	1086.211	24.78874	1.907392	0.059149	1082.388	20.71969	0.054061	0.00379	1063.845	72.63199	0.075174	0.001567	1067.295	42.53958	5.457347	0.139942	2.426349	1.907392	0.059149	0.183574	0.004541	0.612486	0.167953
91500 - 6		28.33	19	0.182931	0.003991	1082.823	21.77839	1.891805	0.062554	1076.649	21.81844	0.0513	0.003835	1010.694	73.71572	0.075015	0.001879	1060.309	50.9849	5.46882	0.122031	2.358607	1.891805	0.062554	0.182931	0.003991	0.443357	0.086742
91500 - 7		27.00	19	0.177536	0.003964	1053.352	21.65547	1.845634	0.057947	1060.626	20.68649	0.051899	0.0038	1022.26	72.95369	0.07539	0.001638	1072.785	44.25309	5.6375	0.122949	2.438359	1.845634	0.057947	0.177536	0.003964	0.57975	0.023813
91500 - 8		27.00	19	0.177234	0.003888	1051.712	21.25207	1.814509	0.051551	1049.866	18.50933	0.054745	0.003644	1077.09	69.77662	0.0743	0.001429	1044.937	38.70293	5.648619	0.121374	2.43887	1.814509	0.051551	0.177234	0.003888	0.547346	0.147122
91500 - 9		26.74	19	0.180184	0.004059	1067.824	22.15224	1.880654	0.069756	1072.186	24.41121	0.053646	0.003861	1055.824	74.03582	0.075788	0.002026	1080.165	53.97523	5.562596	0.12422	2.430215	1.880654	0.069756	0.180184	0.004059	0.595229	-0.16176
91500 - 11		17.85	19	0.181801	0.004323	1076.674	23.57981	1.903265	0.076917	1080.381	27.56317	0.05088	0.004423	1002.487	85.0592	0.075184	0.00277	1060.49	78.4752	5.504983	0.131025	2.95487	1.903265	0.076917	0.181801	0.004323	0.321452	0.351966
91500 - 12		28.12	19	0.177879	0.003915	1055.238	21.44374	1.817223	0.053274	1050.708	19.11012	0.052485	0.003921	1033.447	75.38537	0.074181	0.001711	1039.342	46.67148	5.626987	0.125651	2.41118	1.817223	0.053274	0.177879	0.003915	0.320877	0.338224
91500 - 13		27.04	19	0.17873	0.003749	1059.927	20.48685	1.819188	0.05115	1051.578	18.48368	0.049692	0.003365	980.0229	64.82159	0.073758	0.001555	1029.126	42.67869	5.597502	0.11662	2.381212	1.819188	0.05115	0.17873	0.003749	0.342866	0.267611
91500 - 14		24.73	19	0.179375	0.003671	1063.481	20.04875	1.840899	0.058309	1059.021	20.88813	0.053406	0.003811	1060.762	64.19854	0.074551	0.001826	1049.142	50.29669	5.575858	0.112965	2.413523	1.840899	0.058309	0.179375	0.003671	0.34314	0.101988
91500 - 15		14.25	19	0.182329	0.004603	1079.551	25.11882	1.873885	0.062194	1071.129	21.9442	0.052334	0.003918	1030.783	75.29631	0.074891	0.001912	1060.826	51.01572	5.489265	0.140566	2.372989	1.873885	0.062194	0.182329	0.004603	0.459297	0.240376
91500 - 16		27.01	19	0.17942	0.003863	1063.685	21.12642	1.841965	0.056033	1059.444	20.1011	0.053653	0.003734	1056.052	71.57735	0.074551	0.001716	1049.534	46.70014	5.576763	0.121458	2.366549	1.841965	0.056033	0.17942	0.003863	0.412851	0.159556
91500 - 17		15.01	19	0.183668	0.004346	1086.872	23.62245	1.88886	0.077469	1075.673	27.20715	0.055614	0.004214	1093.629	80.67886	0.074756	0.002305	1054.582	64.12631	5.445592	0.126048	2.331975	1.88886	0.077469	0.183668	0.004346	0.561102	-0.12394
91500 - 18		25.42	19	0.17678	0.003654	1049.275	20.00868	1.806472	0.058833	1046.523	21.19454	0.054538	0.004066	1072.866	77.8169	0.074117	0.001917	1036.422	52.59406	5.657211	0.116674	2.66758	1.806472	0.058833	0.17678	0.003654	0.583366	0.159557
					sumary stats																							
					IsoplotR	1066.21	5.41	0.51%	40,457.5	3794.51%																		
Z_AUSZ7-1																												
AUSZ7-1 - 1		26.83	19	0.005929	0.000209	38.10602	1.337754	0.038412	0.004348	38.18638	4.233651	0.001949	0.000312	39.34146	6.28642	0.046138	0.005045	-24.1777	244.8554	170.0791	5.985265	2.706065	0.038412	0.004348	0.005929	0.000209	-0.08634	0.370754
AUSZ7-1 - 2		24.13	19	0.005993	0.000197	38.5169	1.263064	0.040572	0.003128	40.34407	3.051835	0.001787	0.000321	36.08183	6.479506	0.049597	0.003999	69.19499	204.836	166.5982	5.145786	2.747549	0.040572	0.003128	0.005993	0.000197	-0.02653	0.400047
AUSZ7-1 - 3		27.00	19	0.006067	0.000187	38.99302	1.195976	0.037193	0.005218	36.95225	5.073548	0.002214	0.000354	44.68214	7.139344	0.044799	0.006321	-492.701	422.4681	165.8201	5.258825	2.873917	0.037193	0.005218	0.006067	0.000187	0.033166	0.127636
AUSZ7-1 - 4		27.00	19	0.006232	0.000162	40.04616	1.037322	0.040628	0.004163	40.35704	4.057052	0.002177	0.000345	43.93423	6.95252	0.047539	0.004827	-71.6793	259.5397	160.8975	4.091001	2.6351	0.040628	0.004163	0.006232	0.000162	0.092216	0.101663
AUSZ7-1 - 5		27.00	19	0.00612	0.000172	39.33075	1.102794	0.042486	0.005457	42.11065	5.27577	0.001779	0.000329	35.91209	6.64185	0.048306	0.005842	-99.3706	318.7536	163.9569	4.652787	2.763259	0.042486	0.005457	0.00612	0.000172	0.016133	0.194786
AUSZ7-1 - 6		27.00	19	0.005909	0.000164	37.98174	1.050529	0.037274	0.004386	37.06939	4.272408	0.001704	0.000301	34.39909	6.067997	0.045859	0.005273	-127.257	329.1964	169.796	4.777818	2.677157	0.037274	0.004386	0.005909	0.000164	0.037498	0.062372
AUSZ7-1 - 7		27.01	19	0.005923	0.00019	38.06842	1.214682	0.040961	0.004712	40.65967	4.573934	0.002051	0.000317	41.4061	6.393492	0.050466	0.005826	-3.80257	266.843	169.9883	5.831732	2.763507	0.040961	0.004712	0.005923	0.00019	0.060253	0.212801
AUSZ7-1 - 8		27.01	19	0.005905	0.000194	37.95616	1.246385	0.043898	0.004765	43.52095	4.628621	0.002002	0.000376	40.40547	7.586922	0.054372	0.006198	167.1313	278.1815	170.6213	5.902686	2.730616	0.043898	0.004765	0.005905	0.000194	0.007726	0.257283
AUSZ7-1 - 9		27.00	19	0.006152	0.000198	39.53451	1.267368	0.042651	0.004698	42.30955	4.563145	0.002036	0.000398	41.09857	8.021179	0.051027	0.005949	76.34164	270.5851	163.819	5.432812	2.875564	0.042651	0.004698	0.006152	0.000198	-0.16364	0.409585
AUSZ7-1 - 10		27.01	19	0.005975	0.000184	38.4045	1.180141	0.041194	0.005196	40.86331	5.040383	0.001774	0.000266	35.82237	5.361176	0.048681	0.005722	-82.7812	326.9578	168.4573	5.33936	2.711395	0.041194	0.005196	0.005975	0.000184	-0.16345	0.364152
AUSZ7-1 - 11		27.01	19	0.005951	0.000209	38.24964	1.338036	0.034756	0.00421	34.60958	4.123007	0.001725	0.000256	34.82322	5.170164	0.043053	0.00554	-448.622	369.4502	169.386	5.78651	2.689079	0.034756	0.00421	0.005951	0.000209	-0.15527	0.435754
AUSZ7-1 - 12		27.00	19	0.006067	0.000195	38.9918	1.248616	0.037068	0.004755	36.85091	4.655744	0.002161	0.000363	43.6289	7.31016	0.044677	0.005965	-65.539	249.5281	165.8853	5.302965	2.775547	0.037068	0.004755	0.006067	0.000195	-0.00791	0.236358
AUSZ7-1 - 13		21.03	19	0.005949	0.000186	38.23346	1.194797	0.04453	0.005439	44.13036	5.269582	0.002062	0.000446	41.62533	8.998703	0.054638	0.006709	173.8202	326.6786	168.9061	5.568406	2.642714	0.04453	0.005439	0.005949	0.000186	-0.01228	0.313579
AUSZ7-1 - 14		27.00	19	0.005905	0.000185	37.95059	1.18482	0.042809	0.005086	42.44591	4.94117	0.001819	0.000261	36.7291	5.266685	0.052788	0.006206	162.6746	270.1414	170.3744	5.406683	2.470551	0.042809	0.005086	0.005905	0.000185	0.1335	0.071094
AUSZ7-1 - 15		27.01	19	0.005916	0.000188	38.02316	1.201802	0.038277	0.005139	38.01659	5.010689	0.002008	0.000405	40.5392	8.160431	0.047271	0.006287	-241.92	377.8367	170.0896	5.368717	2.95172	0.038277	0.005139	0.005916	0.000188	0.053651	0.14495
AUSZ7-1 - 16		27.00	19	0.00601	0.000218	38.62294	1.39825	0.043245	0.003762	42.92339	3.660738	0.001942	0.000321	39.20819	6.482018	0.053536	0.004755	267.2497	199.3704	167.9746	6.161342	2.647205	0.043245	0.003762	0.00601	0.000218	0.282424	0.053044
AUSZ7-1 - 17		27.01	19	0.005827	0.000194	37.45515	1.246487	0.037575	0.003814	37.38641	3.721259	0.002265	0.000377	45.70712	7.601134	0.046104	0.004497	-159.385	288.6678	172.8381	5.80783	2.673468	0.037575	0.003814	0.005827	0.000194	-0.08604	0.320925
AUSZ7-1 - 18		27.02	19	0.005901	0.000198	37.92972	1.270934	0.039862	0.004352	39.60127	4.243019	0.001875	0.000357	37.84718	7.19457	0.049317	0.005462	-23.7009	290.1025	170.6492	5.503422	2.851034	0.039862	0.004352	0.005901	0.000198	0.019923	0.281603
					sumary stats																							
					IsoplotR	38.5	0.28	0.73%	1,460.89	3794.51%																		
Z_AUSZ7-5																												
AUSZ7-5 - 1		26.70	19	0.000333	4.11E-05	2.148954	0.265085	0.00449	0.001909	4.531058	1.924267	0.000153	6.69E-05	3.097307	1.352321	0.126383	0.058407	2154.494	663.3678	3585.496	531.8801	1.364195	0.00449	0.001909	0.000333	4.11E-05	0.079545	0.247134
AUSZ7-5 - 2		27.01	19	0.000364	3.07E-05	2.348062	0.198024	0.007318	0.001897	7.385517	1.909206	0.0002	5.6E-05	4.050971	1.131642	0.137331	0.036663	2166.839	456.2643	2880.406	235.0612	1.222122	0.007318	0.001897	0.000364	3.07E-05	0.281739	-0.02339
AUSZ7-5 - 3		27.00	19	0.000355	3.57E-05	2.285849	0.230315	0.006133	0.002198	6.183915	2.212478	7.94E-05	5.24E-05	1.603935	1.058839	0.131312	0.047473	1905.813	835.0789	3094.797	304.1005	1.380874	0.006133	0.002198	0.000355	3.57E-05	0.145164	0.150369
AUSZ7-5 - 4		27.03	19	0.0004	3.83E-05	2.57755	0.246695	0.004279	0.001815	4.319124	1.830631	0.000138	5.19E-05	2.781008	1.048873	0.069592	0.036207	1692.401	629.5516	2655.906	252.4864	1.258162	0.004279	0.001815	0.0004	3.83E-05	0.021993	-0.10245
AUSZ7-5 - 5		27.00	19	0.000392	4.37E-05	2.525155	0.281703	0.006748	0.002456	6.799157	2.468343	0.000248	0.000104	5.011663	2.109535	0.155961	0.065786	1577.905	833.1956	2767.792	354.3856	1.471223	0.006748	0.002456	0.000392	4.37E-05	0.001934	0.168937
AUSZ7-5 - 6		27.00	19	0.000387	3.1E-05	2.496445	0.199884	0.004528	0.001387	4.577396	1.401151	0.000208	6.19E-05	4.200801	1.251287	0.095401	0.031962	2100.512	343.8438	2698.87	227.3097	1.357436	0.004528	0.001387	0.000387	3.1E-05	0.200879	-0.10751
AUSZ7-5 - 7		27.01	19	0.000383	3.9E-05	2.46829	0.251016	0.002907	0.0017	2.933103	1.717612	0.000141	7.61E-05	2.857857	1.538082	0.054929	0.033545	2145.241	542.1517	2897.347	312.2352	1.437448	0.002907	0.0017	0.000383	3.9E-05	0.065295	-0.05065
AUSZ7-5 - 8		27.00	19	0.000369	3.75E-05	2.379014	0.241545	0.005243	0.002092	5.286913	2.104853	0.00022	9.53E-05	4.437363	1.926188	0.097274	0.039745	1367.829	797.9438	3046.502	355.9614	1.392715	0.005243	0.002092	0.000369	3.75E-05	0.140982	0.092272
AUSZ7-5 - 9		27.00	19	0.000377	4.14E-05	2.429669	0.266543	0.004042	0.001589	4.082914	1.604298	0.000135	6.32E-05	2.726125	1.276259	0.084002	0.036218	1024.504	858.56	2953.097	355.6012	1.443628	0.004042	0.001589	0.000377	4.14E-05	-0.17599	0.447518
AUSZ7-5 - 10		27.00	19	0.000406	3.72E-05	2.61618	0.239987	0.002264	0.00133	2.286606	1.344891	0.000171	7.26E-05	3.459425	1.467894	0.040345	0.023518	383.7779	938.6117	2686.915	261.7715	1.367509	0.002264	0.00133	0.000406	3.72E-05	0.109751	-0.06156
AUSZ7-5 - 11		27.00	19	0.000397	4.08E-05	2.55791	0.263014	0.005014	0.002124	5.055673	2.135599	0.000155	6.95E-05	3.127441	1.404951	0.074571	0.034733	1077.505	716.3918	2748.962	309.6851	1.366957	0.005014	0.002124	0.000397	4.08E-05	-0.15187	0.124416
AUSZ7-5 - 12		27.00	19	0.000375	4.05E-05	2.41802	0.260769	0.002914	0.00134	2.945458	1.354742	0.000214	8.42E-05	4.3174	1.701982	0.052265	0.027317	417.0037	860.6175	2968.311	378.6421	1.43546	0.002914	0.00134	0.000375	4.05E-05	0.166296	0.146196
AUSZ7-5 - 13		27.00	19	0.000353	3.79E-05	2.27652	0.244001	0.003017	0.00148	3.047381	1.494876	0.000167	6.02E-05	3.369466	1.216481	0.070668	0.033912	1255.867	778.6248	3120.801	371.1999	1.426969	0.003017	0.00148	0.000353	3.79E-05	-0.06486	0.294162
AUSZ7-5 - 14		27.00	19	0.000382	5.16E-05	2.463074	0.332198	0.005954	0.001852	6.009734	1.868106	0.000182	0.000105	3.684919	2.118527	0.135709	0.055373	2063.635	558.1869	3251.035	643.5916	1.504256	0.005954	0.001852	0.000382	5.16E-05	0.051808	0.071145
AUSZ7-5 - 15		27.00	19	0.000332	3.14E-05	2.138688	0.202307	0.003757	0.001354	3.798317	1.36772	0.000172	6.02E-05	3.478012	1.215748	0.089325	0.0337	944.0944	757.8871	3201.479	271.4897	1.232888	0.003757	0.001354	0.000332	3.14E-05	-0.01709	0.204515
AUSZ7-5 - 16		27.01	19	0.000392	4.91E-05	2.52738	0.316276	0.002894	0.001355	2.925071	1.370497	0.000188	7.87E-05	3.796727	1.591092	0.058316	0.031302	387.2717	890.2581	2907.373	385.4793	1.380068	0.002894	0.001355	0.000392	4.91E-05	0.089043	0.025762
AUSZ7-5 - 17		27.00	19	0.00036	4E-05	2.318141	0.258025	0.003964	0.001616	4.00399	1.630131	0.000157	7.44E-05	3.172892	1.504159	0.093449	0.043101	1085.656	790.4176	2955.063	284.4113	1.423138	0.003964	0.001616	0.00036	4E-05	-0.18313	0.229789
AUSZ7-5 - 18		27.01	19	0.000406	3.91E-05	2.618892	0.252193	0.003567	0.001497	3.603873	1.510985	0.000155	6.68E-05	3.140682	1.349865	0.06838	0.029835	889.968	650.2385	2624.513	239.2122	1.385552	0.003567	0.001497	0.000406	3.91E-05	-0.03277	0.04278
					sumary stats																							
					IsoplotR	2.41	0.057	2.37%	91.448	3794.51%																		

	Duration (s)	Spot Size (µm)	Final Pb206/U238_mean	Final Pb206/U238_2SE(prop)	Final Pb206/U238 age_mean	Final Pb206/U238 age_2SE(prop)	Final Pb207/U235_mean	Final Pb207/U235_2SE(prop)	Final Pb207/U235 age_mean	Final Pb207/U235 age_2SE(prop)	Final Pb208/Th232_mean	Final Pb208/Th232_2SE(prop)	Final Pb208/Th232 age_mean	Final Pb208/Th232 age_2SE(prop)	Final Pb207/Pb206_mean	Final Pb207/Pb206_2SE(prop)	Final Pb207/Pb206 age_mean	Final Pb207/Pb206 age_2SE(prop)	Final U238/Pb206_mean	Final U238/Pb206_2SE(prop)	Final U/Th_mean	Final Pb207/U235_mean	Final Pb207/U235_2SE(prop)	Final Pb206/U238_mean	Final Pb206/U238_2SE(prop)	rho 206Pb/238U v 207Pb/235U	rho 207Pb/206Pb v 238U/206Pb
**DR-40-1**																											
DR40-1 - 1	27.00	19	0.007456	0.000219	**47.88511**	**1.404141**	0.055059	0.007483	54.16744	7.160129	0.002348	0.000307	47.38564	6.181315	0.054031	0.007449	218.9398	293.7516	134.8028	4.106335	2.028686	0.055059	0.007483	0.007456	0.000219	-0.14085	0.28373
DR40-1 - 2	27.01	19	0.007552	0.00031	**48.49851**	**1.982903**	0.060075	0.008591	58.90361	8.202538	0.002212	0.000396	44.65482	7.975366	0.056942	0.007454	330.3699	297.6639	134.2731	5.783949	1.824235	0.060075	0.008591	0.007552	0.00031	0.475189	-0.24789
DR40-1 - 3	27.00	19	0.007662	0.000251	**49.20477**	**1.608192**	0.054176	0.005487	53.43911	5.294101	0.002331	0.000329	47.054	6.642576	0.051444	0.005231	123.3513	263.2431	131.4087	4.304178	2.221134	0.054176	0.005487	0.007662	0.000251	0.11989	0.138166
DR40-1 - 4	27.00	19	0.007219	0.000344	**46.36364**	**2.199656**	0.04927	0.008523	48.50242	8.138851	0.002071	0.000512	41.79034	10.31288	0.048511	0.008054	-100.347	461.9776	137.511	5.374134	2.102107	0.04927	0.008523	0.007219	0.000344	-0.15501	0.444936
DR40-1 - 5	27.01	19	0.00739	0.000221	**47.46179**	**1.413609**	0.052748	0.00637	52.01571	6.11144	0.002615	0.000458	52.77095	9.224104	0.052269	0.006566	10.91457	330.2922	136.0283	4.149405	2.393523	0.052748	0.00637	0.00739	0.000221	-0.14254	0.35777
DR40-1 - 6	27.00	19	0.007428	0.000205	**47.70931**	**1.310584**	0.049755	0.005005	49.19083	4.814727	0.002324	0.000255	46.90425	5.144505	0.048347	0.004451	-26.0262	216.432	135.1511	3.778032	1.263175	0.049755	0.005005	0.007428	0.000205	0.401346	-0.20274
DR40-1 - 7	15.29	19	0.00749	0.000269	**48.10204**	**1.721529**	0.049203	0.005558	48.69091	5.346107	0.002373	0.000499	47.88997	10.06374	0.048002	0.005663	-25.1702	263.0598	134.2195	5.134241	2.388038	0.049203	0.005558	0.00749	0.000269	-0.02704	0.31041
DR40-1 - 8	22.28	19	0.007628	0.000234	**48.98447**	**1.495131**	0.048445	0.00466	47.95459	4.511995	0.00239	0.000298	48.24	6.005221	0.046161	0.004381	-80.1951	230.2776	131.7026	3.989889	1.614208	0.048445	0.00466	0.007628	0.000234	0.09321	0.150586
DR40-1 - 9	27.01	19	0.007557	0.000223	**48.53231**	**1.423438**	0.057879	0.00731	56.89587	7.010874	0.002628	0.000417	53.03463	8.400564	0.055485	0.006915	193.9513	343.0333	132.9614	3.848158	2.10506	0.057879	0.00731	0.007557	0.000223	0.195397	-0.03447
DR40-1 - 10	27.01	19	0.007406	0.000273	**47.56719**	**1.749965**	0.048587	0.004969	48.06381	4.814528	0.002243	0.000245	45.28065	4.950123	0.049409	0.004811	-3.62746	250.4201	136.3889	5.152461	1.46435	0.048587	0.004969	0.007406	0.000273	0.298946	0.052504
DR40-1 - 11	9.71	19	0.007633	0.000384	**49.01431**	**2.458709**	0.080461	0.014402	78.273	13.33896	0.00285	0.000765	57.5007	15.41778	0.076745	0.013548	978.2459	348.0257	131.9884	6.857785	1.806185	0.080461	0.014402	0.007633	0.000384	0.040146	0.182026
DR40-1 - 12	12.14	19	0.007646	0.000413	**49.1016**	**2.644534**	0.0494	0.00972	48.76675	9.379107	0.002697	0.000641	54.41288	12.91757	0.047335	0.009221	-333.715	617.8657	132.3883	7.883689	1.901534	0.0494	0.00972	0.007646	0.000413	0.133679	0.240825
DR40-1 - 13	8.84	19	0.007578	0.000323	**48.6663**	**2.067669**	0.054575	0.00913	53.84413	8.711956	0.002327	0.0006	46.96173	12.09808	0.052492	0.008668	184.8473	359.7726	133.3256	6.093756	1.748309	0.054575	0.00913	0.007578	0.000323	0.110423	0.152421
DR40-1 - 14	27.00	19	0.007438	0.000239	**47.76813**	**1.528942**	0.052756	0.006351	52.02178	6.146594	0.002918	0.000493	58.86807	9.936491	0.051721	0.006321	222.6201	274.6022	135.3083	4.193197	1.84929	0.052756	0.006351	0.007438	0.000239	0.020909	0.118923
DR40-1 - 15	17.88	19	0.007269	0.000331	**46.69049**	**2.117542**	0.053152	0.005983	52.47762	5.747698	0.002266	0.000333	45.74052	6.717676	0.053399	0.006218	202.3537	270.809	139.2919	6.983414	1.445154	0.053152	0.005983	0.007269	0.000331	0.218116	0.299911
DR40-1 - 16	19.06	19	0.00765	0.000276	**49.1236**	**1.768706**	0.053976	0.007119	53.22044	6.832573	0.002535	0.000416	51.15815	8.379607	0.051771	0.007382	189.505	261.9463	131.5912	4.885789	1.588305	0.053976	0.007119	0.00765	0.000276	-0.09653	0.362367
DR40-1 - 17	27.00	19	0.007609	0.000333	**48.86055**	**2.129502**	0.050606	0.007991	49.84337	7.605439	0.002214	0.000368	44.68537	7.414971	0.048588	0.006895	26.65826	372.5802	133.4683	5.825072	1.609493	0.050606	0.007991	0.007609	0.000333	0.088434	0.017226
DR40-1 - 18	22.93	19	0.007668	0.000225	**49.24477**	**1.441596**	0.055468	0.005424	54.70316	5.196286	0.002534	0.000401	51.14003	8.080435	0.052654	0.005184	182.7935	222.6389	130.1147	3.320373	2.066628	0.055468	0.005424	0.007668	0.000225	0.112545	0.133593
DR40-1 - 19	27.01	19	0.007537	0.000297	**48.39985**	**1.901246**	0.048882	0.004972	48.3472	4.8143	0.002519	0.000377	50.8418	7.59837	0.047526	0.004929	-25.0104	230.1177	134.3223	5.29157	1.898301	0.048882	0.004972	0.007537	0.000297	0.061195	0.24864
DR40-1 - 20	14.33	19	0.007394	0.000359	**47.48444**	**2.297013**	0.052604	0.0065	51.95659	6.280571	0.002266	0.000473	45.74805	9.5395	0.052407	0.007081	90.41073	415.5949	136.6937	6.834816	2.141414	0.052604	0.0065	0.007394	0.000359	-0.19955	0.478848
DR40-1 - 21	27.01	19	0.007499	0.000213	**48.15957**	**1.361711**	0.05134	0.005179	50.71446	5.007751	0.002555	0.000373	51.56364	7.514494	0.049934	0.004991	97.32238	285.0128	133.2519	3.546677	2.243754	0.05134	0.005179	0.007499	0.000213	0.078416	0.137359
DR40-1 - 22	22.54	19	0.007831	0.000299	**50.28554**	**1.914381**	0.050587	0.008339	49.84292	8.014179	0.002867	0.00058	57.84579	11.6857	0.04707	0.007537	-179.927	406.9014	128.8815	4.919385	1.953573	0.050587	0.008339	0.007831	0.000299	0.111739	0.087825
DR40-1 - 23	8.38	19	0.007815	0.000337	**50.18315**	**2.158003**	0.049377	0.008228	48.84597	7.923492	0.002544	0.000363	51.3507	7.317421	0.04635	0.008419	-155.794	449.6347	128.5052	5.628807	1.517405	0.049377	0.008228	0.007815	0.000337	-0.15846	0.405344
DR40-1 - 24	26.48	19	0.007648	0.000237	**49.11022**	**1.514819**	0.056763	0.005273	55.94003	5.056597	0.002859	0.00035	57.69164	7.052761	0.054422	0.005228	290.9063	215.0955	131.5794	4.143246	2.222651	0.056763	0.005273	0.007648	0.000237	-0.0921	0.369562
DR40-1 - 25	14.11	19	0.007479	0.000313	**48.0319**	**1.99989**	0.057815	0.010152	56.83081	9.659425	0.002885	0.000474	58.22293	9.552533	0.056746	0.010012	353.2802	352.5961	134.6761	5.68125	2.298145	0.057815	0.010152	0.007479	0.000313	-0.04019	0.220104
DR40-1 - 26	9.32	19	0.007786	0.000474	**49.9948**	**3.028801**	0.058801	0.010626	57.85108	10.2376	0.003067	0.001223	61.85118	24.58775	0.055568	0.010528	186.5193	551.8827	129.7932	7.813046	2.314891	0.058801	0.010626	0.007786	0.000474	-0.0519	0.240994
DR40-1 - 27	19.09	19	0.0074	0.000281	**47.52939**	**1.79969**	0.050155	0.007253	49.52055	6.961915	0.002253	0.00044	45.4686	8.873363	0.049811	0.007441	-105.046	388.3358	136.233	5.37353	1.841999	0.050155	0.007253	0.0074	0.000281	-0.05135	0.29316
DR40-1 - 28	27.00	19	0.007356	0.000215	**47.24502**	**1.377772**	0.051196	0.00536	50.5674	5.153802	0.002312	0.000391	46.66748	7.879613	0.050935	0.00544	96.44653	244.2332	136.6661	4.041143	2.242356	0.051196	0.00536	0.007356	0.000215	-0.03701	0.278674
DR40-1 - 29	27.02	19	0.007747	0.000207	**49.75**	**1.320889**	0.048709	0.003688	48.2332	3.566319	0.002294	0.000288	46.30947	5.797302	0.045876	0.003853	-76.0775	185.8912	129.5365	3.39947	1.656279	0.048709	0.003688	0.007747	0.000207	-0.36334	0.56878
DR40-1 - 30	27.00	19	0.007514	0.000211	**48.25726**	**1.352433**	0.04849	0.004504	47.98851	4.351983	0.002279	0.000273	45.99667	5.496777	0.046745	0.004339	-129.982	242.6094	133.6788	3.713714	1.500223	0.04849	0.004504	0.007514	0.000211	0.096846	0.220608
DR40-1 - 31	16.40	19	0.007568	0.000187	**48.60481**	**1.195612**	0.049853	0.002598	49.38235	2.510859	0.002559	0.000316	51.66221	6.380272	0.047989	0.00236	73.40714	114.5308	132.3151	3.170473	8.677632	0.049853	0.002598	0.007568	0.000187	0.167021	0.15737
DR40-1 - 32	20.74	19	0.00698	0.000203	**44.84168**	**1.297458**	0.047768	0.002772	47.35552	2.680687	0.003029	0.000465	61.12519	9.378302	0.048965	0.002139	150.4104	115.6481	143.7976	4.21918	17.67232	0.047768	0.002772	0.00698	0.000203	0.358507	0.060524
DR40-1 - 33	27.00	19	0.007689	0.000288	**49.37343**	**1.844251**	0.051756	0.00703	51.01126	6.755874	0.002309	0.000407	46.61027	8.206012	0.048611	0.006086	-119.53	326.7013	131.5032	5.042291	2.122648	0.051756	0.00703	0.007689	0.000288	0.370647	-0.18007
DR40-1 - 34	21.42	19	0.007269	0.000336	**46.68529**	**2.151861**	0.054781	0.009311	53.84363	8.917122	0.003072	0.000529	61.97523	10.65938	0.052889	0.008677	229.1191	396.824	139.5171	6.420672	1.975562	0.054781	0.009311	0.007269	0.000336	-0.00702	0.368524
DR40-1 - 35	27.01	19	0.007118	0.000169	**45.72535**	**1.084037**	0.053465	0.003241	52.84314	3.103066	0.002237	0.000167	45.17015	3.37517	0.055407	0.003697	369.0939	131.6626	140.2499	3.693942	0.765348	0.053465	0.003241	0.007118	0.000169	0.011796	0.257142
DR40-1 - 36	27.00	19	0.007062	0.000262	**45.36202**	**1.677684**	0.052023	0.005954	52.81483	6.423657	0.002134	0.000331	43.08352	6.668489	0.053637	0.005928	223.2643	298.699	141.97	4.840937	1.873274	0.052023	0.005954	0.007062	0.000262	0.231286	0.067585
DR40-1 - 37	23.89	19	0.007562	0.000338	**48.56464**	**2.163675**	0.051573	0.008778	50.7458	8.493629	0.001858	0.000601	37.49155	12.10699	0.050167	0.008709	-231.117	560.0293	134.1067	5.753423	2.412925	0.051573	0.008778	0.007562	0.000338	0.067479	0.202846
DR40-1 - 38	27.00	19	0.007695	0.000259	**49.41651**	**1.653673**	0.054134	0.006104	53.36237	5.876669	0.002773	0.000422	55.96143	8.509494	0.051569	0.005957	86.06235	296.2891	130.9946	4.452309	1.750435	0.054134	0.006104	0.007695	0.000259	0.012912	0.237935
DR40-1 - 39	24.61	19	0.008012	0.000308	**51.44246**	**1.972645**	0.052266	0.005101	51.62491	4.898759	0.002817	0.000477	56.83731	9.604348	0.047165	0.004033	-14.9809	238.3781	126.1032	4.847108	2.027571	0.052266	0.005101	0.008012	0.000308	0.279119	0.147633
DR40-1 - 40	27.01	19	0.007524	0.000215	**48.31821**	**1.37783**	0.048003	0.004593	47.5151	4.44598	0.002522	0.000285	50.90267	5.743164	0.046196	0.00451	-79.3148	271.7987	133.5243	3.804049	1.405784	0.048003	0.004593	0.007524	0.000215	-0.13453	0.31436
DR40-1 - 41	27.00	19	0.007575	0.00019	**48.64491**	**1.214554**	0.052376	0.002712	51.80694	2.619289	0.003494	0.000529	70.48416	10.65167	0.050474	0.002637	168.1583	128.4068	132.3887	3.3509	12.69416	0.052376	0.002712	0.007575	0.00019	-0.00715	0.386654
DR40-1 - 42	27.00	19	0.007652	0.000273	**49.1388**	**1.744024**	0.051154	0.007672	50.39366	7.344829	0.002424	0.000518	48.91667	10.44341	0.049022	0.007254	-197.276	410.4033	131.9403	4.957704	2.381193	0.051154	0.007672	0.007652	0.000273	0.048553	0.178646
DR40-1 - 43	20.89	19	0.0077	0.000264	**49.44342**	**1.690997**	0.048857	0.008058	48.20801	7.770585	0.002221	0.00044	44.82566	8.860592	0.045791	0.006911	55.67162	338.6916	130.6624	4.381115	2.106267	0.048857	0.008058	0.0077	0.000264	0.40164	-0.28869
DR40-1 - 44	27.01	19	0.007566	0.000225	**48.59101**	**1.440806**	0.048181	0.00413	47.70506	3.969247	0.002302	0.000242	46.46131	4.878968	0.046628	0.004066	-91.31	195.8457	132.8643	3.890417	1.415889	0.048181	0.00413	0.007566	0.000225	-0.04156	0.300698
DR40-1 - 45	27.00	19	0.0076	0.000245	**48.80839**	**1.568091**	0.051274	0.006507	50.57909	6.256999	0.002823	0.000442	56.95275	8.897518	0.04778	0.005838	-46.3764	298.5277	132.4624	4.170378	2.430412	0.051274	0.006507	0.0076	0.000245	0.05972	0.166662
DR40-1 - 46	22.14	19	0.007469	0.000291	**47.96478**	**1.862034**	0.051998	0.004011	51.41424	3.868061	0.00241	0.000267	48.65447	5.374301	0.051245	0.00402	192.9325	222.2753	133.7539	4.472285	1.610688	0.051998	0.004011	0.007469	0.000291	0.103641	0.285837
DR40-1 - 47	27.01	19	0.007685	0.00024	**49.35319**	**1.533644**	0.052834	0.004954	52.17064	4.76643	0.0026	0.000397	52.46265	8.010865	0.050039	0.004501	57.05102	220.7524	130.0106	3.67076	2.193131	0.052834	0.004954	0.007685	0.00024	0.267931	0.024083
DR40-1 - 48	17.34	19	0.00773	0.000446	**49.63356**	**2.84968**	0.069595	0.015489	67.66427	14.55804	0.003179	0.000733	64.12332	14.75957	0.066209	0.014648	617.6587	456.9848	131.709	7.528572	1.902503	0.069595	0.015489	0.00773	0.000446	0.031733	0.097143
DR40-1 - 49	27.00	19	0.007511	0.00025	**48.23424**	**1.60257**	0.050387	0.006228	49.73688	6.016144	0.002626	0.00043	52.99753	8.658194	0.049235	0.006414	-213.902	390.7311	134.191	4.593035	2.412099	0.050387	0.006228	0.007511	0.00025	0.000327	0.234189
DR40-1 - 50	27.00	19	0.007712	0.000323	**49.51886**	**2.067034**	0.050891	0.006222	50.22981	5.995867	0.002102	0.000515	42.42343	10.38397	0.048757	0.005993	-1.26592	332.8686	131.5933	5.874781	2.349561	0.050891	0.006222	0.007712	0.000323	0.173989	0.113727
DR40-1 - 51	27.00	19	0.007782	0.000394	**49.96692**	**2.519246**	0.052918	0.008767	52.00211	8.471416	0.002845	0.000762	57.36142	15.34909	0.051277	0.009299	-21.1628	536.7638	131.424	6.688917	2.322997	0.052918	0.008767	0.007782	0.000394	-0.07317	0.29601
DR40-1 - 52	27.01	19	0.007554	0.000248	**48.51127**	**1.585663**	0.048065	0.005931	47.51099	5.720305	0.002571	0.000335	51.88523	6.749469	0.045273	0.005179	-134.864	329.7221	133.3016	4.313174	1.683321	0.048065	0.005931	0.007554	0.000248	-0.08459	0.223733
DR40-1 - 53	17.95	19	0.007404	0.000249	**47.5538**	**1.590103**	0.050789	0.004533	50.24534	4.371785	0.002447	0.000243	49.39917	4.90417	0.049883	0.004196	113.9177	195.927	135.6475	4.291889	1.324581	0.050789	0.004533	0.007404	0.000249	0.307253	-0.02007
DR40-1 - 54	27.00	19	0.007648	0.000259	**49.11361**	**1.654743**	0.049621	0.003295	49.12738	3.186605	0.002419	0.000221	48.83823	4.458602	0.04748	0.003175	-9.93638	169.2913	131.7621	4.351321	1.050579	0.049621	0.003295	0.007648	0.000259	0.124086	0.288848
DR40-1 - 55	7.23	19	0.007664	0.000474	**49.2182**	**3.033011**	0.053966	0.014857	53.11747	14.31754	0.003005	0.000907	60.63295	18.28121	0.051011	0.013422	-218.703	951.908	131.5014	7.911979	1.398146	0.053966	0.014857	0.007664	0.000474	0.377965	-0.17316
				sumary stats IsoplotR	**48.51**	**0.23**	0.47%	**1,840.72**	3794.51%																		
**DR-40-3**																											
DR40-3 - 1	27.00	19	0.007545	0.000247	**48.45292**	**1.581517**	0.052338	0.006134	51.62845	5.88911	0.002517	0.000335	50.7953	6.750793	0.050537	0.005672	55.36569	259.9296	133.4741	4.281911	2.055201	0.052338	0.006134	0.007545	0.000247	0.194175	0.043354
DR40-3 - 2	25.79	19	0.007425	0.000245	**47.68693**	**1.569988**	0.052087	0.005116	51.44482	4.928713	0.002317	0.000305	46.76802	6.147122	0.049869	0.004427	76.47101	260.1719	135.601	4.395676	1.670424	0.052087	0.005116	0.007425	0.000245	0.161809	0.166939
DR40-3 - 3	23.61	19	0.008156	0.000352	**52.3587**	**2.250993**	0.056406	0.009801	55.33854	9.444342	0.003547	0.000776	71.52448	15.62038	0.051621	0.009635	106.2337	457.7758	124.4111	6.038003	2.356618	0.056406	0.009801	0.008156	0.000352	-0.15185	0.410862
DR40-3 - 4	26.26	19	0.007479	0.000239	**48.03469**	**1.528006**	0.046809	0.004903	46.34277	4.75514	0.002396	0.000371	48.36566	7.470929	0.045848	0.005033	-148.062	321.0353	134.5715	4.323998	2.009151	0.046809	0.004903	0.007479	0.000239	-0.14427	0.385359

	Duration (s)	Spot Size (µm)	Final Pb206/U238_mean	Final Pb206/U238_2SE(prop)	Final Pb206/U238 age_mean	Final Pb206/U238 age_2SE(prop)	Final Pb207/U235_mean	Final Pb207/U235_2SE(prop)	Final Pb207/U235 age_mean	Final Pb207/U235 age_2SE(prop)	Final Pb208/Th232_mean	Final Pb208/Th232_2SE(prop)	Final Pb208/Th232 age_mean	Final Pb208/Th232 age_2SE(prop)	Final Pb207/Pb206_mean	Final Pb207/Pb206_2SE(prop)	Final Pb207/Pb206 age_mean	Final Pb207/Pb206 age_2SE(prop)	Final U238/Pb206_mean	Final U238/Pb206_2SE(prop)	Final U/Th_mean	Final Pb207/U235_mean	Final Pb207/U235_2SE(prop)	Final Pb206/U238_mean	Final Pb206/U238_2SE(prop)	rho 206Pb/238U v 207Pb/235U	rho 207Pb/206Pb v 238U/206Pb
DR40-3 - 6	12.16	19	0.00788	0.00045	50.5972	2.877928	0.055703	0.010982	54.79989	10.43274	0.002428	0.00056	49.0043	11.28045	0.051444	0.009101	-16.1356	497.0443	128.4358	7.019426	1.292837	0.055703	0.010982	0.00788	0.00045	0.264175	0.08795
DR40-3 - 7	21.81	19	0.007714	0.000314	49.53379	2.010059	0.052687	0.010816	51.70654	10.28092	0.002529	0.000371	51.04556	7.476486	0.04742	0.00908	-340.943	556.2525	130.9905	5.287535	1.804915	0.052687	0.010816	0.007714	0.000314	-0.04758	0.245314
DR40-3 - 9	27.02	19	0.007415	0.000279	47.62095	1.785096	0.052072	0.006476	51.35133	6.232579	0.002481	0.000453	50.06563	9.13594	0.051697	0.006749	23.95799	337.007	135.2223	4.846831	2.603408	0.052072	0.006476	0.007415	0.000279	0.062802	0.337494
DR40-3 - 10	27.00	19	0.0077	0.000262	49.44812	1.678562	0.055386	0.006451	54.55278	6.174168	0.002737	0.00041	55.24131	8.259801	0.052447	0.00591	78.45145	289.0099	130.8941	4.48827	2.50163	0.055386	0.006451	0.0077	0.000262	0.182996	0.085655
DR40-3 - 11	25.72	19	0.00728	0.000215	46.75955	1.37656	0.059755	0.00563	58.79967	5.341765	0.002544	0.0003	51.34986	6.040028	0.059676	0.005481	523.5086	208.4229	138.0214	4.054331	1.890652	0.059755	0.00563	0.00728	0.000215	-0.07379	0.32173
DR40-3 - 12	27.00	19	0.00772	0.000232	49.57336	1.487132	0.05388	0.005925	53.12642	5.715671	0.00271	0.00041	54.68226	8.25977	0.052392	0.006293	181.2295	273.3397	130.2622	4.01097	2.179007	0.05388	0.005925	0.00772	0.000232	0.214608	0.073239
DR40-3 - 14	27.00	19	0.007427	0.00023	47.69739	1.472674	0.050732	0.006029	50.08201	5.832048	0.0028	0.000514	56.48248	10.34851	0.049983	0.006167	-81.2936	376.4021	133.8089	4.083797	2.456043	0.050732	0.006029	0.007427	0.00023	-0.03074	0.080322
DR40-3 - 15	18.95	19	0.007611	0.000385	48.87586	2.464237	0.057653	0.007014	56.76364	6.713496	0.002688	0.00057	54.2406	11.47708	0.055578	0.006336	419.9696	281.6362	133.3398	6.640092	2.633296	0.057653	0.007014	0.007611	0.000385	0.290122	0.037241
DR40-3 - 16	27.00	19	0.007535	0.000233	48.38828	1.492764	0.052465	0.005269	51.79631	5.082355	0.00227	0.000323	45.8277	6.506717	0.050552	0.004853	111.4733	231.7199	133.5076	4.093135	2.30131	0.052465	0.005269	0.007535	0.000233	0.282734	-0.04608
DR40-3 - 17	17.67	19	0.007806	0.000463	50.11815	2.962527	0.062523	0.01021	61.27997	9.746516	0.003603	0.000812	72.64453	16.34592	0.058905	0.009091	549.2669	296.6844	127.8604	6.250993	2.063961	0.062523	0.01021	0.007806	0.000463	0.144046	0.217791
DR40-3 - 18	23.28	19	0.007657	0.000293	49.17089	1.873286	0.059967	0.007885	58.89445	7.547063	0.00298	0.000581	60.12046	11.70502	0.057481	0.007962	363.2588	347.3305	131.8686	5.169328	2.205286	0.059967	0.007885	0.007657	0.000293	-0.21381	0.408967
DR40-3 - 19	27.01	19	0.007415	0.000256	47.62407	1.640141	0.052731	0.008073	51.88621	7.768191	0.00218	0.000382	43.99547	7.697754	0.052118	0.008205	-275.709	508.8868	135.9211	4.536049	2.225748	0.052731	0.008073	0.007415	0.000256	0.035188	0.216276
DR40-3 - 20	27.00	19	0.007499	0.000226	48.16045	1.445799	0.056489	0.00557	55.6621	5.321924	0.002646	0.000347	53.39145	6.986232	0.055019	0.004676	288.9516	211.7462	134.0513	3.99118	1.649318	0.056489	0.00557	0.007499	0.000226	-0.07556	0.121661
DR40-3 - 21	26.62	19	0.0073	0.000314	46.88241	2.010445	0.051925	0.007158	51.1716	6.883838	0.002469	0.000654	49.80486	13.16628	0.052169	0.007201	120.9702	327.3741	138.9259	5.702033	2.310841	0.051925	0.007158	0.0073	0.000314	-0.218	0.280562
DR40-3 - 22	26.73	19	0.007194	0.000208	46.20679	1.328172	0.053187	0.004631	52.5253	4.458682	0.002638	0.000385	53.24493	7.753867	0.0538	0.004412	306.6232	171.7231	138.7736	4.399546	3.007749	0.053187	0.004631	0.007194	0.000208	0.268737	0.035141
DR40-3 - 23	17.92	19	0.007443	0.000436	47.79843	2.788668	0.064324	0.014747	62.65264	13.96094	0.003236	0.001182	65.20565	23.78668	0.06462	0.015089	262.0827	713.1057	137.169	8.182451	2.501586	0.064324	0.014747	0.007443	0.000436	-0.09714	0.328967
DR40-3 - 24	24.18	19	0.007691	0.000316	49.38489	2.02452	0.049753	0.008128	49.02647	7.830631	0.003222	0.000694	64.97166	13.98084	0.047989	0.008221	-44.1841	356.4873	131.6264	5.465468	2.503315	0.049753	0.008128	0.007691	0.000316	-0.16213	0.355337
DR40-3 - 25	27.00	19	0.007597	0.000271	48.78769	1.732636	0.059597	0.006391	58.60241	6.094702	0.002555	0.000455	51.55348	9.173067	0.057379	0.006254	321.6082	250.7548	132.7883	4.667868	2.092218	0.059597	0.006391	0.007597	0.000271	0.056305	0.213057
DR40-3 - 26	27.00	19	0.007406	0.000256	47.5626	1.638737	0.057013	0.005822	56.15271	5.580073	0.002933	0.00037	59.19332	7.459875	0.056554	0.005994	283.2644	265.6947	136.1544	4.763016	1.961227	0.057013	0.005822	0.007406	0.000256	0.144738	-0.11508
DR40-3 - 27	26.58	19	0.007437	0.000341	47.76087	2.184511	0.055881	0.008582	54.88433	8.225842	0.002177	0.000429	43.93916	8.652017	0.055426	0.008649	144.2569	405.4658	135.3727	5.776993	1.888738	0.055881	0.008582	0.007437	0.000341	0.087181	0.28808
DR40-3 - 28	18.45	19	0.007728	0.000379	49.62496	2.422733	0.055679	0.006659	54.88548	6.381608	0.002941	0.00094	59.30514	18.92665	0.053387	0.007526	148.5612	309.9795	131.1153	6.335935	2.33272	0.055679	0.006659	0.007728	0.000379	-0.08506	0.509516
DR40-3 - 29	9.67	19	0.007944	0.0003	51.00474	1.916242	0.059632	0.013987	58.50696	13.33985	0.002824	0.000638	56.98533	12.85965	0.053998	0.011467	288.4754	514.8831	126.2744	4.620709	2.185282	0.059632	0.013987	0.007944	0.0003	0.431905	-0.24525
DR40-3 - 30	18.73	19	0.00718	0.00033	46.11823	2.112544	0.056439	0.00786	55.55805	7.487928	0.002175	0.000258	43.91955	5.199518	0.056886	0.006922	305.6255	313.0738	140.8976	6.320044	1.219804	0.056439	0.00786	0.00718	0.00033	0.3226	-0.09333
DR40-3 - 31	25.11	19	0.007828	0.000269	50.26602	1.724006	0.058962	0.00499	58.06974	4.78068	0.002726	0.000394	55.01175	7.945706	0.056322	0.005214	392.9634	194.9909	127.5834	3.927896	2.035941	0.058962	0.00499	0.007828	0.000269	0.135553	0.184583
DR40-3 - 32	27.02	19	0.007565	0.000209	48.5834	1.338563	0.050907	0.003972	50.34848	3.826283	0.002579	0.000327	52.05311	6.596948	0.048876	0.003537	44.5022	183.4067	132.7091	3.639222	1.995293	0.050907	0.003972	0.007565	0.000209	0.273699	0.081462
DR40-3 - 33	20.49	19	0.00804	0.000328	51.61921	2.097887	0.058742	0.008207	57.73742	7.83122	0.003516	0.000716	70.91605	14.40608	0.052033	0.00723	118.4143	323.1564	125.5677	5.155974	2.173927	0.058742	0.008207	0.00804	0.000328	0.182537	0.091172
DR40-3 - 34	18.63	19	0.007199	0.000298	46.24165	1.907201	0.054268	0.010746	53.30847	10.33165	0.002266	0.00066	45.72707	13.29604	0.055309	0.011158	508.1709	346.6932	138.4151	4.828926	2.193969	0.054268	0.010746	0.007199	0.000298	-0.03007	0.193872
DR40-3 - 35	10.90	19	0.007872	0.000578	50.54608	3.695245	0.059703	0.010867	58.68263	10.43128	0.003106	0.000753	62.665	15.17683	0.055131	0.009426	152.8659	573.1594	129.37	9.448144	1.895301	0.059703	0.010867	0.007872	0.000578	0.393347	-0.00228
DR40-3 - 36	27.01	19	0.007311	0.000187	46.95646	1.19941	0.053305	0.004381	52.64966	4.215115	0.002603	0.000264	52.53882	5.330299	0.053042	0.004326	218.7253	196.0124	137.1751	3.583185	1.630725	0.053305	0.004381	0.007311	0.000187	0.034528	0.184016
DR40-3 - 37	27.01	19	0.00724	0.000244	46.50361	1.561726	0.054336	0.005826	55.01136	6.252774	0.00216	0.000349	43.60349	7.037947	0.056695	0.006769	298.9605	281.475	138.0856	4.1766	1.834249	0.054336	0.005826	0.00724	0.000244	-0.12343	0.34827
DR40-3 - 38	27.00	19	0.007496	0.000193	48.13909	1.236764	0.048504	0.004951	47.98204	4.774186	0.002369	0.000215	47.82644	4.33629	0.04698	0.00478	-140.534	257.1117	133.794	3.390096	1.28803	0.048504	0.004951	0.007496	0.000193	0.109851	0.03518
DR40-3 - 39	27.00	19	0.007391	0.000243	47.46934	1.552289	0.050511	0.007688	49.761	7.435949	0.00244	0.000462	49.23348	9.306493	0.049872	0.007832	-253.669	518.9657	136.306	4.646864	2.104035	0.050511	0.007688	0.007391	0.000243	0.066984	0.168087
DR40-3 - 40	24.43	19	0.007403	0.000237	47.5439	1.516127	0.048011	0.004473	47.53335	4.341171	0.002625	0.000288	52.98406	5.804609	0.046916	0.004092	-26.6844	208.9514	135.8834	4.359229	1.534511	0.048011	0.004473	0.007403	0.000237	0.385709	-0.08092
DR40-3 - 41	16.55	19	0.007462	0.000286	47.92162	1.830113	0.045796	0.007847	45.29898	7.59132	0.002774	0.000671	55.97171	13.52844	0.04512	0.008179	-329.487	496.2657	134.8476	5.157402	2.204004	0.045796	0.007847	0.007462	0.000286	-0.1989	0.372977
DR40-3 - 42	27.00	19	0.007813	0.000223	50.16831	1.429649	0.061485	0.005597	62.34498	6.530039	0.002755	0.000351	55.59005	7.083951	0.059294	0.006174	506.8451	240.8507	128.6036	3.884115	2.229781	0.061485	0.005597	0.007813	0.000223	0.154495	0.035803
DR40-3 - 43	26.08	19	0.007846	0.000233	50.38073	1.488462	0.0578	0.006221	56.88836	5.930796	0.002996	0.000423	60.44506	8.518801	0.05363	0.005825	174.5277	246.0049	128.0833	3.802295	2.054978	0.0578	0.006221	0.007846	0.000233	0.039329	0.178337
DR40-3 - 44	27.01	19	0.007535	0.000299	48.3875	1.910509	0.056296	0.008019	55.32105	7.632824	0.00268	0.000525	54.06343	10.57897	0.054954	0.008406	111.2736	341.9619	134.2691	5.106797	2.165383	0.056296	0.008019	0.007535	0.000299	-0.05736	0.261363
DR40-3 - 45	26.58	19	0.008179	0.000302	52.50859	1.931408	0.053644	0.005008	52.94998	4.815955	0.002827	0.000422	57.04921	8.505306	0.047813	0.00433	-76.8689	251.0913	123.4253	4.429742	2.00406	0.053644	0.005008	0.008179	0.000302	0.15329	0.219299
DR40-3 - 46	20.92	19	0.007717	0.000332	49.5506	2.124189	0.057418	0.009417	59.21022	10.57664	0.002716	0.000545	54.80069	10.9873	0.053753	0.008833	125.4087	508.8121	131.0685	5.737214	1.863692	0.057418	0.009417	0.007717	0.000332	-0.09519	0.304527
DR40-3 - 47	27.01	19	0.007629	0.000221	48.99405	1.412829	0.053364	0.007709	52.51647	7.400524	0.002933	0.000469	59.17909	9.447846	0.050167	0.006956	53.46344	326.5261	130.9528	4.319037	2.583883	0.053364	0.007709	0.007629	0.000221	0.280467	-0.05441
DR40-3 - 48	22.21	19	0.007671	0.000346	49.26005	2.211275	0.052527	0.006839	51.80424	6.582934	0.003273	0.000556	66.01742	11.19669	0.050418	0.007116	31.97166	331.509	132.1827	6.020869	2.245568	0.052527	0.006839	0.007671	0.000346	-0.02385	0.361503
DR40-3 - 49	22.96	19	0.007582	0.000212	48.69158	1.355921	0.057155	0.006728	56.26615	6.469925	0.002674	0.00038	53.96876	7.650478	0.055062	0.006677	245.6726	315.2227	132.3219	3.769565	1.930704	0.057155	0.006728	0.007582	0.000212	-0.21268	0.379867
DR40-3 - 50	27.01	19	0.007657	0.00028	49.17314	1.792952	0.053698	0.006473	54.51906	6.966827	0.002365	0.000414	47.7259	8.344242	0.050824	0.005879	-49.8678	388.0604	131.8507	4.779354	1.680405	0.053698	0.006473	0.007657	0.00028	0.23463	0.036856
DR40-3 - 51	14.10	19	0.007952	0.00045	51.05779	2.877079	0.057737	0.012895	56.6079	12.27103	0.002164	0.000762	43.65443	15.36798	0.058035	0.011585	476.959	545.8588	127.4934	6.967933	2.994143	0.057737	0.012895	0.007952	0.00045	0.111294	0.140479
DR40-3 - 52	6.71	19	0.007673	0.000457	49.27123	2.920972	0.061697	0.011423	60.66063	10.83555	0.002803	0.000653	56.55922	13.1572	0.058523	0.011445	437.856	380.6917	131.149	7.693034	1.641518	0.061697	0.011423	0.007673	0.000457	-0.02613	0.278523
DR40-3 - 53	23.85	19	0.007712	0.000333	49.52027	2.132916	0.063093	0.009213	61.79905	8.754654	0.003047	0.000522	61.47463	10.51139	0.060675	0.009839	358.0806	351.9174	131.3733	5.837767	1.780615	0.063093	0.009213	0.007712	0.000333	-0.16912	0.435946
DR40-3 - 54	24.72	19	0.007412	0.000314	47.60411	2.007923	0.050652	0.007783	49.92042	7.460273	0.002709	0.000659	54.64013	13.27949	0.04785	0.006645	96.55435	294.158	136.6796	5.871183	2.122129	0.050652	0.007783	0.007412	0.000314	0.070445	0.12503
DR40-3 - 55	21.35	19	0.007486	0.000299	48.07741	1.910425	0.055351	0.007684	54.492	7.383848	0.002895	0.00058	58.4066	11.68491	0.054449	0.008074	164.7558	368.987	134.8098	5.30307	2.37667	0.055351	0.007684	0.007486	0.000299	-0.18956	0.427727
				sumary stats IsoplotR	48.49	0.39	0.80%	1,839.96	3794.51%																		
**DR-40-6**																											
DR40-6 - 1	27.00	19	0.007445	0.000232	47.81736	1.48314	0.051972	0.004332	51.36333	4.18167	0.0024	0.000263	48.44008	5.299234	0.050848	0.004282	163.0707	187.5881	135.1062	4.241994	1.965071	0.051972	0.004332	0.007445	0.000232	0.038524	0.285364
DR40-6 - 2	27.01	19	0.007321	0.000309	47.0175	1.975483	0.053161	0.006503	52.40528	6.258159	0.002161	0.00028	43.61792	5.651708	0.053702	0.007439	92.44548	342.5433	138.4624	5.781224	1.815278	0.053161	0.006503	0.007321	0.000309	-0.13792	0.424808
DR40-6 - 3	24.90	19	0.007391	0.000188	47.4666	1.203327	0.048067	0.003549	47.61965	3.438381	0.002338	0.000208	47.19013	4.196039	0.04713	0.003396	12.19018	166.1623	135.6099	3.488145	1.027036	0.048067	0.003549	0.007391	0.000188	0.133311	0.119104
DR40-6 - 4	27.00	19	0.007623	0.000204	48.95447	1.304773	0.052965	0.003839	52.34218	3.697381	0.002562	0.000256	51.70905	5.165793	0.050134	0.003689	155.9965	159.0183	131.5726	3.404269	1.227458	0.052965	0.003839	0.007623	0.000204	-0.03677	0.275519
DR40-6 - 5	24.86	19	0.007538	0.000296	48.40921	1.895579	0.055268	0.007548	54.38352	7.21482	0.002746	0.000473	55.41762	9.520807	0.053336	0.007169	2.716192	387.4857	132.8375	4.800845	1.9729	0.055268	0.007548	0.007538	0.000296	0.188742	0.170378
DR40-6 - 6	27.00	19	0.007221	0.000214	46.38235	1.372443	0.048362	0.003656	47.89792	3.547993	0.002171	0.000205	43.82277	4.126032	0.048674	0.003668	87.4662	172.7547	139.1214	4.10372	1.111811	0.048362	0.003656	0.007221	0.000214	0.037357	0.258951
DR40-6 - 7	13.76	19	0.008263	0.000412	53.04601	2.63736	0.056312	0.013839	55.17865	13.16204	0.002822	0.000474	56.95792	9.553789	0.04979	0.011967	54.92862	492.3844	122.3208	6.292784	2.079644	0.056312	0.013839	0.008263	0.000412	0.048098	0.145691
DR40-6 - 8	14.05	19	0.007363	0.000296	47.28999	1.892257	0.052579	0.006382	51.9412	6.148841	0.002659	0.000417	53.67431	8.408761	0.052133	0.006532	141.0755	337.6621	136.5476	5.495637	2.013928	0.052579	0.006382	0.007363	0.000296	-0.07266	0.307611
DR40-6 - 9	19.17	19	0.007259	0.000227	46.62158	1.449962	0.050356	0.003771	49.84153	3.64109	0.002403	0.000258	48.51182	5.205949	0.050656	0.003839	155.5716	179.5102	138.2637	4.211402	1.487857	0.050356	0.003771	0.007259	0.000227	0.390604	-0.26318
DR40-6 - 10	10.64	19	0.007791	0.000547	50.02736	3.498674	0.050894	0.010766	50.19232	10.49471	0.002514	0.000916	50.72257	18.45469	0.048276	0.010666	351.4791	516.6196	130.3433	8.245962	2.253723	0.050894	0.010766	0.007791	0.000547	-0.21352	0.29594
DR40-6 - 11	26.11	19	0.007618	0.000286	48.92012	1.827736	0.054031	0.007332	53.1988	7.030612	0.002345	0.000378	47.32873	7.626088	0.051898	0.007361	94.24888	324.8864	132.6019	5.242239	1.440158	0.054031	0.007332	0.007618	0.000286	0.014649	0.284852

	Duration (s)	Spot Size (µm)	Final Pb206/U238_mean	Final Pb206/U238_2SE(prop)	Final Pb206/U238 age_mean	Final Pb206/U238 age_2SE(prop)	Final Pb207/U235_mean	Final Pb207/U235_2SE(prop)	Final Pb207/U235 age_mean	Final Pb207/U235 age_2SE(prop)	Final Pb208/Th232_mean	Final Pb208/Th232_2SE(prop)	Final Pb208/Th232 age_mean	Final Pb208/Th232 age_2SE(prop)	Final Pb207/Pb206_mean	Final Pb207/Pb206_2SE(prop)	Final Pb207/Pb206 age_mean	Final Pb207/Pb206 age_2SE(prop)	Final U238/Pb206_mean	Final U238/Pb206_2SE(prop)	Final U/Th_mean	Final Pb207/U235_mean	Final Pb207/U235_2SE(prop)	Final Pb206/U238_mean	Final Pb206/U238_2SE(prop)	rho 206Pb/238U v 207Pb/235U	rho 207Pb/206Pb v 238U/206Pb
DR40-6 - 13	24.11	19	0.007378	0.000218	**47.38863**	**1.391915**	0.049309	0.003109	48.8361	3.00565	0.002357	0.000198	47.58641	3.987914	0.048474	0.002989	61.63759	152.7048	136.0545	4.006806	1.171572	0.049309	0.003109	0.007378	0.000218	0.121449	0.278983
DR40-6 - 14	18.66	19	0.00784	0.000373	**50.33726**	**2.384274**	0.054851	0.009594	53.93307	9.183389	0.002879	0.000789	58.06631	15.89387	0.050921	0.00935	135.7046	333.2679	129.2967	6.635923	2.862336	0.054851	0.009594	0.00784	0.000373	0.132681	0.145525
DR40-6 - 15	6.59	19	0.007655	0.000659	**49.15514**	**4.215761**	0.055908	0.013192	55.04426	12.56369	0.002645	0.001184	53.35176	23.85642	0.053681	0.013293	108.2444	602.6559	132.6519	10.58885	1.712191	0.055908	0.013192	0.007655	0.000659	-0.08804	0.333344
DR40-6 - 16	5.95	19	0.007428	0.000512	**47.70366**	**3.278999**	0.056088	0.013609	55.24434	13.03444	0.001781	0.000742	35.95486	14.95318	0.055377	0.014383	204.7663	622.8296	135.6724	9.382246	1.835029	0.056088	0.013609	0.007428	0.000512	-0.12939	0.40477
DR40-6 - 17	18.99	19	0.008056	0.000379	**51.72204**	**2.422774**	0.055194	0.010788	54.18473	10.35519	0.002313	0.00066	46.67598	13.30577	0.050347	0.010241	-29.143	491.0169	123.9277	4.937464	2.508772	0.055194	0.010788	0.008056	0.000379	-0.03485	0.323511
DR40-6 - 18	27.01	19	0.007735	0.000275	**49.66881**	**1.761819**	0.047741	0.006784	48.84614	7.282511	0.002286	0.000609	46.11576	12.27526	0.046872	0.007299	-308.147	388.2745	129.352	4.306431	2.717537	0.047741	0.006784	0.007735	0.000275	-0.05203	0.22457
DR40-6 - 19	26.04	19	0.0077	0.000287	**49.44616**	**1.833175**	0.05947	0.007142	58.44098	6.79849	0.002763	0.00045	55.74944	9.062715	0.056188	0.006876	242.6164	272.2293	131.062	4.84599	2.736218	0.05947	0.007142	0.0077	0.000287	0.103909	0.167695
DR40-6 - 20	18.12	19	0.007365	0.000319	**47.29968**	**2.041175**	0.054593	0.009142	53.71882	8.79512	0.002618	0.000548	52.83469	11.03855	0.05491	0.010346	-162.403	655.1816	137.0628	5.990603	2.24079	0.054593	0.009142	0.007365	0.000319	-0.30202	0.540958
DR40-6 - 21	27.00	19	0.007508	0.000212	**48.21863**	**1.354783**	0.050787	0.003821	50.23801	3.68682	0.002347	0.000232	47.37994	4.676955	0.049747	0.003688	88.03406	179.8906	133.7022	3.855402	1.301811	0.050787	0.003821	0.007508	0.000212	0.403129	-0.15278
DR40-6 - 22	8.74	19	0.007953	0.000426	**51.06289**	**2.721834**	0.063346	0.016272	61.98408	15.47055	0.002801	0.0007	56.51751	14.11996	0.058304	0.015924	109.7954	710.5478	126.5955	6.589354	2.159689	0.063346	0.016272	0.007953	0.000426	-0.17548	0.322634
DR40-6 - 23	27.00	19	0.007907	0.00023	**50.77155**	**1.4711**	0.054953	0.005278	54.19833	5.069569	0.002538	0.000309	51.23361	6.234509	0.050533	0.004858	121.9789	221.3164	127.0049	3.804462	1.730244	0.054953	0.005278	0.007907	0.00023	-0.00885	0.268804
DR40-6 - 24	27.00	19	0.007835	0.00027	**50.30996**	**1.729946**	0.057699	0.007419	56.70978	7.099694	0.002337	0.000457	47.15661	9.201295	0.053121	0.00697	-10.7681	370.3101	127.7161	5.035805	2.116001	0.057699	0.007419	0.007835	0.00027	0.068678	0.156963
DR40-6 - 25	27.00	19	0.007531	0.00021	**48.36802**	**1.344448**	0.053341	0.005212	52.6448	5.028066	0.002416	0.000313	48.75969	6.307855	0.051187	0.004803	137.6469	239.7645	133.2563	3.807447	1.721728	0.053341	0.005212	0.007531	0.00021	0.226037	-0.01386
DR40-6 - 27	27.00	19	0.007461	0.000297	**47.91622**	**1.89805**	0.052104	0.009003	51.21222	8.591993	0.00267	0.000458	53.86732	9.22399	0.050988	0.008696	-164.104	485.6551	135.5877	5.486731	1.556403	0.052104	0.009003	0.007461	0.000297	0.165016	-0.02588
DR40-6 - 28	27.00	19	0.007553	0.000277	**48.50351**	**1.774094**	0.053032	0.007418	52.22424	7.113273	0.002405	0.000507	48.52652	10.22046	0.051475	0.00735	-2.71657	339.0516	133.5582	4.747949	2.055766	0.053032	0.007418	0.007553	0.000277	-0.10436	0.29815
DR40-6 - 29	16.16	19	0.007257	0.000351	**46.60791**	**2.246596**	0.052297	0.011705	51.3931	11.12755	0.002564	0.000522	51.74087	10.5256	0.052535	0.011415	-57.5112	542.6186	139.3498	6.930529	1.888172	0.052297	0.011705	0.007257	0.000351	0.073423	0.208244
DR40-6 - 30	25.76	19	0.007959	0.000318	**51.0997**	**2.032578**	0.076706	0.011578	74.49057	10.79175	0.004461	0.000859	89.89451	17.28057	0.070133	0.010118	674.1285	335.4459	127.0873	5.185747	2.460449	0.076706	0.011578	0.007959	0.000318	0.119126	0.156298
DR40-6 - 31	11.29	19	0.007747	0.000466	**49.74483**	**2.979699**	0.064002	0.012758	62.70779	12.01787	0.002753	0.000979	55.53907	19.72148	0.061098	0.014601	393.612	414.6086	130.6041	7.810889	2.602704	0.064002	0.012758	0.007747	0.000466	-0.22247	0.464373
DR40-6 - 32	27.01	19	0.00741	0.000249	**47.59259**	**1.593605**	0.049975	0.006523	49.32492	6.313565	0.002359	0.00041	47.6145	8.260387	0.048908	0.006408	-50.2869	352.4376	135.8518	4.519839	2.432204	0.049975	0.006523	0.00741	0.000249	0.107117	0.070637
DR40-6 - 33	11.61	19	0.00741	0.000393	**47.59103**	**2.512833**	0.049672	0.011848	48.95156	11.43431	0.003672	0.00121	74.02229	24.32893	0.048828	0.011606	-233.482	744.2186	136.2411	7.469562	2.342599	0.049672	0.011848	0.00741	0.000393	0.012158	0.159595
DR40-6 - 35	14.08	19	0.007848	0.000397	**50.39327**	**2.538385**	0.06725	0.011869	65.77983	11.2196	0.003287	0.000501	66.32468	10.09414	0.061342	0.009719	454.9797	370.2901	128.6581	6.460069	2.181836	0.06725	0.011869	0.007848	0.000397	0.531559	-0.29673
DR40-6 - 36	27.01	19	0.007882	0.000375	**50.60898**	**2.398394**	0.060579	0.010702	59.20501	10.12303	0.002691	0.000731	54.26013	14.71526	0.057298	0.010664	97.87121	451.2514	129.3787	6.487802	2.669769	0.060579	0.010702	0.007882	0.000375	-0.09787	0.391941
DR40-6 - 37	11.85	19	0.007635	0.000246	**49.03001**	**1.572941**	0.053347	0.007136	52.67522	6.894193	0.002342	0.00041	47.27205	8.268139	0.050728	0.00672	61.85462	399.956	131.1957	4.300572	2.127872	0.053347	0.007136	0.007635	0.000246	0.042259	0.176877
DR40-6 - 38	15.16	19	0.00765	0.00022	**49.12804**	**1.407544**	0.056674	0.00648	55.87346	6.208214	0.002525	0.0003	50.95864	6.043801	0.053525	0.005805	248.3674	247.5758	130.8827	3.653541	1.341496	0.056674	0.00648	0.00765	0.00022	0.321854	-0.11487
DR40-6 - 39	13.61	19	0.007262	0.000381	**46.64545**	**2.439497**	0.061193	0.011767	60.00056	11.20893	0.003704	0.000752	74.69501	15.14548	0.060552	0.011031	531.4119	353.2591	139.0142	6.624384	3.175469	0.061193	0.011767	0.007262	0.000381	0.349315	-0.08364
DR40-6 - 40	27.01	19	0.007269	0.000273	**46.68484**	**1.747073**	0.048814	0.007621	48.12315	7.368584	0.002198	0.000471	44.348	9.485445	0.048766	0.007853	-39.6199	386.7196	137.9283	5.611317	2.225959	0.048814	0.007621	0.007269	0.000273	-0.03198	0.215767
DR40-6 - 41	8.35	19	0.007631	0.000562	**49.00304**	**3.593806**	0.047762	0.010007	47.23677	9.674248	0.002631	0.000614	53.09427	12.37443	0.045733	0.009415	-318.119	643.7028	133.0447	10.69233	1.457858	0.047762	0.010007	0.007631	0.000562	0.090739	0.312375
DR40-6 - 42	27.00	19	0.007714	0.000239	**49.53574**	**1.529574**	0.050697	0.005326	50.08896	5.144568	0.002227	0.000326	44.95499	6.579126	0.048024	0.005322	-154.593	317.625	129.4516	3.679341	1.811752	0.050697	0.005326	0.007714	0.000239	-0.1415	0.388931
DR40-6 - 43	20.78	19	0.007931	0.000301	**50.92525**	**1.922837**	0.053409	0.007088	52.65779	6.831781	0.002189	0.000382	44.18317	7.7006	0.049358	0.006975	-40.7315	352.7023	127.0695	5.133549	1.485034	0.053409	0.007088	0.007931	0.000301	-0.0825	0.362821
DR40-6 - 44	26.19	19	0.007186	0.00025	**46.15988**	**1.601359**	0.054113	0.005713	53.36637	5.516225	0.002459	0.000312	49.63633	6.295848	0.055112	0.00629	153.4222	326.8651	139.1603	4.181712	0.974126	0.054113	0.005713	0.007186	0.00025	-0.11315	0.393637
DR40-6 - 45	25.93	19	0.007902	0.000289	**50.73986**	**1.847112**	0.056986	0.008064	55.99337	7.721749	0.002366	0.000471	47.74833	9.499761	0.052064	0.007185	178.8305	275.2369	127.6585	4.816144	2.545608	0.056986	0.008064	0.007902	0.000289	0.276055	-0.01357
DR40-6 - 46	27.01	19	0.007487	0.000246	**48.08187**	**1.576735**	0.058836	0.006301	57.87236	6.033937	0.002651	0.000447	53.49611	9.010509	0.05614	0.00559	317.5369	275.4873	133.5045	4.944018	2.25223	0.058836	0.006301	0.007487	0.000246	0.21997	-0.01534
DR40-6 - 47	27.01	19	0.007236	0.000207	**46.47709**	**1.322143**	0.04761	0.004091	47.15171	3.966336	0.002322	0.000274	46.87196	5.534094	0.047677	0.004028	7.208897	199.71	138.6861	3.860319	2.145378	0.04761	0.004091	0.007236	0.000207	0.14339	0.103488
DR40-6 - 48	20.92	19	0.007742	0.00033	**49.71356**	**2.111015**	0.04775	0.009649	47.03566	9.278466	0.002723	0.00039	54.96227	7.853436	0.042253	0.007696	-54.6032	404.2046	128.9688	4.83116	1.780116	0.04775	0.009649	0.007742	0.00033	0.135554	0.06567
DR40-6 - 49	17.44	19	0.007372	0.000335	**47.34904**	**2.142912**	0.051673	0.007933	50.97996	7.659981	0.002588	0.000422	52.23928	8.514587	0.051509	0.008454	-90.3899	476.6447	136.9685	6.413243	2.206343	0.051673	0.007933	0.007372	0.000335	-0.06814	0.370519
DR40-6 - 50	27.00	19	0.007413	0.000242	**47.60854**	**1.550206**	0.056905	0.008795	55.84367	8.428815	0.002559	0.000491	51.6365	9.888388	0.055469	0.008411	206.5798	363.4001	135.7652	4.546127	2.16216	0.056905	0.008795	0.007413	0.000242	0.201896	-0.02273
DR40-6 - 51	16.73	19	0.007473	0.000371	**47.99505**	**2.374088**	0.054418	0.009488	53.55239	9.082171	0.002192	0.000438	44.2412	8.835201	0.052173	0.008114	-16.2602	442.6157	135.3663	6.566908	1.599897	0.054418	0.009488	0.007473	0.000371	0.441572	-0.16928
DR40-6 - 52	12.97	19	0.007697	0.000311	**49.42564**	**1.989811**	0.055104	0.008155	54.32522	7.759103	0.002601	0.000377	52.49241	7.58831	0.052037	0.007798	137.834	310.3136	130.5244	5.232833	1.847667	0.055104	0.008155	0.007697	0.000311	0.005975	0.26004
DR40-6 - 54	27.01	19	0.008321	0.000281	**53.41789**	**1.797085**	0.05348	0.005326	52.77363	5.12062	0.002977	0.000366	60.07392	7.380148	0.045653	0.00431	-90.3538	242.4774	120.135	3.638734	2.479317	0.05348	0.005326	0.008321	0.000281	0.065063	0.223552
DR40-6 - 55	27.00	19	0.00775	0.000246	**49.76354**	**1.572576**	0.062886	0.007241	63.90339	8.08547	0.002419	0.000392	48.82072	7.904351	0.060249	0.006886	452.0182	272.6402	128.9589	4.232825	1.541318	0.062886	0.007241	0.00775	0.000246	0.46574	-0.23626
				sumary																							
				stats																							
				IsoplotR	**48.44**	**0.41**	0.85%	**1,838.06**	3794.51%																		
**DR-40-8**																											
DR40-8 - 1	27.00	19	0.007692	0.000322	**49.39262**	**2.058315**	0.055404	0.00459	54.66087	4.424156	0.002445	0.000461	49.33499	9.290045	0.054151	0.005596	313.0401	224.3025	131.8121	5.772907	2.224686	0.055404	0.00459	0.007692	0.000322	-0.13267	0.448153
DR40-8 - 2	14.76	19	0.00645	0.000266	**41.44286**	**1.704112**	0.048866	0.005175	48.37785	5.000182	0.002465	0.000277	49.75812	5.589257	0.055272	0.006287	305.6602	270.2622	155.9572	6.307665	1.800601	0.048866	0.005175	0.00645	0.000266	-0.11141	0.406952
DR40-8 - 3	19.49	19	0.007622	0.000284	**48.94536**	**1.817057**	0.055089	0.006098	54.33181	5.862719	0.002622	0.000462	52.90596	9.313521	0.052181	0.00543	160.4329	250.0826	132.0307	4.983184	1.871655	0.055089	0.006098	0.007622	0.000284	0.390109	-0.02114
DR40-8 - 4	27.00	19	0.007822	0.000346	**50.22623**	**2.210041**	0.057066	0.00746	56.09918	7.130696	0.002222	0.00042	44.85535	8.455448	0.053094	0.007131	90.28216	320.8648	129.8315	5.819613	2.04198	0.057066	0.00746	0.007822	0.000346	0.197779	0.082459
DR40-8 - 5	14.47	19	0.007288	0.000311	**46.81213**	**1.993197**	0.048141	0.007243	47.61703	6.997769	0.002346	0.000358	47.34908	7.223163	0.047673	0.006601	-144.703	427.6995	138.091	6.008393	1.686816	0.048141	0.007243	0.007288	0.000311	0.287056	-0.06037
DR40-8 - 6	27.00	19	0.007738	0.000223	**49.69157**	**1.429435**	0.051804	0.004479	51.19434	4.318362	0.002278	0.000263	45.99008	5.294035	0.048441	0.003903	51.28025	187.8028	129.7119	3.723757	1.403172	0.051804	0.004479	0.007738	0.000223	0.29188	-0.00609
DR40-8 - 7	17.91	19	0.007639	0.000265	**49.05458**	**1.698166**	0.054493	0.005107	53.80003	4.900058	0.002302	0.000316	46.46483	6.378079	0.052069	0.005472	175.6915	235.8876	131.4776	4.484482	1.351632	0.054493	0.005107	0.007639	0.000265	-0.1668	0.446146
DR40-8 - 8	10.61	19	0.007477	0.000324	**48.02154**	**2.072858**	0.053581	0.014127	52.6374	13.55306	0.002481	0.00051	50.06731	10.29091	0.052672	0.014335	78.89744	827.46	134.3273	5.593653	1.44151	0.053581	0.014127	0.007477	0.000324	-0.28222	0.406996
DR40-8 - 9	26.15	19	0.007783	0.000246	**49.97691**	**1.571326**	0.048861	0.004738	48.34071	4.565417	0.002381	0.00026	48.06257	5.237778	0.045548	0.004249	-189.315	244.0338	129.1504	4.026073	1.541931	0.048861	0.004738	0.007783	0.000246	0.174417	0.072867
DR40-8 - 10	19.88	19	0.007469	0.000273	**47.96635**	**1.746985**	0.056827	0.00536	56.02834	5.147688	0.002526	0.000316	50.98471	6.37771	0.055411	0.005255	309.6384	236.8407	133.2299	4.156206	1.495772	0.056827	0.00536	0.007469	0.000273	0.059861	0.281126
DR40-8 - 11	22.21	19	0.007659	0.000234	**49.18311**	**1.49918**	0.058505	0.005362	57.63041	5.139412	0.002531	0.000302	51.0838	6.078664	0.055426	0.00494	314.5705	224.1041	131.0517	4.118755	1.360838	0.058505	0.005362	0.007659	0.000234	0.119445	0.12001
DR40-8 - 12	24.57	19	0.00756	0.000205	**48.55017**	**1.309065**	0.052971	0.003873	52.35184	3.723427	0.002363	0.000242	47.69522	4.877511	0.050793	0.003444	193.2401	175.207	132.5813	3.577683	1.35294	0.052971	0.003873	0.00756	0.000205	0.198227	0.060858
DR40-8 - 13	19.35	19	0.007817	0.000255	**50.19436**	**1.629871**	0.053634	0.005491	52.95329	5.290499	0.002612	0.000399	52.70922	8.036581	0.050235	0.005524	114.4879	275.9051	128.3979	4.060963	2.24436	0.053634	0.005491	0.007817	0.000255	-0.28391	0.467448
DR40-8 - 14	27.00	19	0.007904	0.000306	**50.75236**	**1.957658**	0.055458	0.005762	54.6558	5.538962	0.002518	0.000296	50.82735	5.976081	0.051233	0.005446	96.50682	264.3006	127.8133	4.844225	2.018954	0.055458	0.005762	0.007904	0.000306	0.09479	0.227274
DR40-8 - 15	16.87	19	0.007597	0.000206	**48.78905**	**1.315762**	0.053562	0.006089	52.87764	5.849409	0.002083	0.000314	42.04978	6.326762	0.051071	0.00566	113.9463	268.7698	131.7728	3.667581	1.533376	0.053562	0.006089	0.007597	0.000206	0.262225	-0.0502
DR40-8 - 16	23.86	19	0.00766	0.000281	**49.18663**	**1.795341**	0.054232	0.004842	53.53382	4.665929	0.002514	0.000293	50.74708	5.89833	0.050601	0.004414	161.7786	193.9455	131.5806	4.894309	1.606097	0.054232	0.004842	0.00766	0.000281	0.287248	0.195634
DR40-8 - 17	27.02	19	0.007391	0.000208	**47.47208**	**1.32871**	0.050156	0.004061	49.62032	3.918854	0.002419	0.00029	48.82563	5.848001	0.048888	0.003773	81.76649	179.3449	133.9993	3.624047	2.116986	0.050156	0.004061	0.007391	0.000208	0.237482	0.066211
DR40-8 - 18	27.00	19	0.007595	0.000255	**48.77684**	**1.632975**	0.050884	0.005331	50.26703	5.122267	0.002346	0.00029	47.3478	5.847297	0.048346	0.004619	-39.8397	226.7777	132.5766	4.533298	1.759497	0.050884	0.005331	0.007595	0.000255	0.397906	-0.13616
DR40-8 - 19	16.08	19	0.007381	0.000263	**47.4048**	**1.681091**	0.051065	0.004632	50.51616	4.477699	0.002123	0.000258	42.85529	5.213346	0.050175	0.004398	194.9031	179.0948	136.011	4.835269	1.166476	0.051065	0.004632	0.007381	0.000263	0.193627	0.168441

	Duration (s)	Spot Size (µm)	Final Pb206/U238_mean	Final Pb206/U238_2SE(prop)	Final Pb206/U238 age_mean	Final Pb206/U238 age_2SE(prop)	Final Pb207/U235_mean	Final Pb207/U235_2SE(prop)	Final Pb207/U235 age_mean	Final Pb207/U235 age_2SE(prop)	Final Pb208/Th232_mean	Final Pb208/Th232_2SE(prop)	Final Pb208/Th232 age_mean	Final Pb208/Th232 age_2SE(prop)	Final Pb207/Pb206_mean	Final Pb207/Pb206_2SE(prop)	Final Pb207/Pb206 age_mean	Final Pb207/Pb206 age_2SE(prop)	Final U238/Pb206_mean	Final U238/Pb206_2SE(prop)	Final U/Th_mean	Final Pb207/U235_mean	Final Pb207/U235_2SE(prop)	Final Pb206/U238_mean	Final Pb206/U238_2SE(prop)	rho 206Pb/238U v 207Pb/235U	rho 207Pb/206Pb v 238U/206Pb
DR40-8 - 21	13.65	19	0.007648	0.000289	**49.1119**	**1.850327**	0.055091	0.006716	54.35202	6.453698	0.002429	0.000448	49.03569	9.030017	0.052511	0.006396	288.0266	249.4183	131.2651	4.850368	1.79052	0.055091	0.006716	0.007648	0.000289	-0.15713	0.344929
DR40-8 - 22	18.35	19	0.00734	0.000241	**47.14036**	**1.545196**	0.061949	0.008545	60.81282	8.18398	0.00232	0.000449	46.82309	9.060439	0.06144	0.00875	513.7016	326.4752	136.7222	4.365941	1.777161	0.061949	0.008545	0.00734	0.000241	-0.01984	0.219669
DR40-8 - 23	16.51	19	0.007828	0.000265	**50.26744**	**1.692006**	0.053054	0.006248	52.38117	6.02907	0.002536	0.00037	51.18586	7.458297	0.048821	0.005279	35.65974	400.2541	128.1768	4.168973	1.567574	0.053054	0.006248	0.007828	0.000265	0.134026	0.092949
DR40-8 - 24	27.00	19	0.007708	0.000356	**49.49511**	**2.279475**	0.050323	0.005917	49.692	5.7376	0.002437	0.000452	49.18838	9.107983	0.048091	0.005582	58.01078	300.994	131.9724	6.111764	1.943862	0.050323	0.005917	0.007708	0.000356	-0.02683	0.26315
DR40-8 - 25	20.13	19	0.007385	0.000205	**47.43282**	**1.314303**	0.057014	0.008092	56.08463	7.708384	0.002374	0.000245	47.92055	4.93499	0.055862	0.007775	230.4099	308.949	135.6634	3.766722	1.156997	0.057014	0.008092	0.007385	0.000205	0.178999	-0.03163
DR40-8 - 26	8.46	19	0.007933	0.000346	**50.93411**	**2.215818**	0.054487	0.006966	53.80582	6.699985	0.002608	0.000435	52.64077	8.765155	0.050166	0.00688	108.544	321.2014	126.48	5.634912	2.390971	0.054487	0.006966	0.007933	0.000346	-0.06958	0.356328
DR40-8 - 27	25.72	19	0.007827	0.000286	**50.25741**	**1.827496**	0.05696	0.006588	56.06805	6.302356	0.002712	0.000388	54.72403	7.821365	0.053596	0.006657	175.7993	284.3756	128.8103	4.71264	2.217933	0.05696	0.006588	0.007827	0.000286	-0.19098	0.422691
DR40-8 - 28	27.00	19	0.007625	0.000273	**48.96336**	**1.746245**	0.052682	0.004889	52.02387	4.729021	0.002264	0.000313	45.70257	6.312268	0.051805	0.005648	207.9976	221.8117	132.2613	4.739409	1.908	0.052682	0.004889	0.007625	0.000273	0.097446	0.231564
DR40-8 - 29	25.50	19	0.007865	0.000273	**50.50335**	**1.745402**	0.051669	0.005661	51.0192	5.455269	0.002824	0.00038	56.99532	7.653573	0.048158	0.005575	-67.8194	288.7661	127.9842	4.2672	2.154063	0.051669	0.005661	0.007865	0.000273	-0.11552	0.354028
DR40-8 - 30	17.27	19	0.00756	0.000275	**48.54776**	**1.762121**	0.051515	0.006067	50.89828	5.846458	0.002555	0.00035	51.57111	7.048241	0.049665	0.005881	-3.89066	331.9191	132.9299	4.709392	1.31654	0.051515	0.006067	0.00756	0.000275	0.036596	0.245434
DR40-8 - 31	22.07	19	0.00737	0.000294	**47.33452**	**1.882361**	0.048876	0.006782	48.28177	6.564575	0.002198	0.00035	44.36567	7.059832	0.047952	0.006199	-305.158	503.5624	136.9008	5.400018	1.73128	0.048876	0.006782	0.00737	0.000294	0.328966	-0.06454
DR40-8 - 32	3.12	19	0.007754	0.000247	**49.79382**	**1.577098**	0.04784	0.004439	47.44132	4.302751	0.003276	0.000782	66.09897	15.75982	0.044911	0.004476	-79.3268	244.6171	128.8523	4.120828	13.58573	0.04784	0.004439	0.007754	0.000247	-0.18584	0.432886
2nd intebration	8.96	19	0.010565	0.000397	**67.74959**	**2.534107**	0.071953	0.006486	70.49413	6.124676	0.003367	0.000704	67.93115	14.17924	0.049704	0.004947	126.1156	237.3554	94.85189	3.6573	2.625073	0.071953	0.006486	0.010565	0.000397	-0.23591	0.49677
3rd integration	7.95	19	0.00767	0.000378	**49.25644**	**2.420278**	0.05074	0.008477	50.16646	8.178809	0.002837	0.000555	57.25821	11.18407	0.048177	0.00845	-44.8556	439.8232	130.9234	6.371863	1.947486	0.05074	0.008477	0.00767	0.000378	-0.06274	0.255695
DR40-8 - 33	11.50	19	0.007503	0.000315	**48.18623**	**2.018239**	0.055403	0.009409	54.58554	8.970467	0.002419	0.00091	48.79837	18.32801	0.053706	0.009167	168.1834	391.0286	133.9677	6.091907	2.330213	0.055403	0.009409	0.007503	0.000315	0.019491	0.166173
DR40-8 - 34	22.75	19	0.007837	0.000201	**50.32273**	**1.286015**	0.050542	0.004671	49.98383	4.516102	0.002157	0.0003	43.53729	6.049389	0.046731	0.004179	-108.725	259.5224	127.7803	3.214025	1.715337	0.050542	0.004671	0.007837	0.000201	0.209129	-0.0041
DR40-8 - 35	10.93	19	0.007771	0.000408	**49.90253**	**2.609237**	0.053011	0.012778	52.15375	12.24298	0.002277	0.000617	45.95139	12.44273	0.050105	0.01292	28.71885	508.1635	129.6401	6.300723	2.081397	0.053011	0.012778	0.007771	0.000408	0.00266	0.284708
DR40-8 - 36	12.61	19	0.007515	0.000257	**48.26094**	**1.644807**	0.046563	0.004755	46.16327	4.6194	0.002357	0.000331	47.57221	6.681807	0.045433	0.005202	-167.856	335.8205	133.398	4.409363	1.601331	0.046563	0.004755	0.007515	0.000257	-0.39204	0.612778
DR40-8 - 37	12.86	19	0.007264	0.000244	**46.65665**	**1.558912**	0.06942	0.00728	68.04184	6.875397	0.003076	0.000606	62.06948	12.21101	0.069305	0.007205	841.3048	207.8646	137.9848	4.558643	2.394281	0.06942	0.00728	0.007264	0.000244	0.107453	0.121263
DR40-8 - 38	27.01	19	0.007321	0.000179	**47.0243**	**1.143298**	0.048445	0.003624	47.97995	3.501838	0.002269	0.000203	45.81421	4.090345	0.048129	0.003637	8.386168	181.9943	136.7672	3.256901	1.839084	0.048445	0.003624	0.007321	0.000179	-0.01102	0.273012
DR40-8 - 39	14.48	19	0.006853	0.00028	**44.02266**	**1.791971**	0.048625	0.004961	48.1504	4.791201	0.002329	0.000251	47.01233	5.057435	0.051322	0.004773	182.3217	205.2305	144.9008	5.003392	1.930313	0.048625	0.004961	0.006853	0.00028	0.434088	-0.03904
DR40-8 - 40	27.01	19	0.00782	0.000218	**50.21304**	**1.396**	0.051033	0.005817	50.38933	5.587747	0.002492	0.000403	50.299	8.126881	0.047991	0.005365	-139.141	297.4561	128.2896	3.544904	2.771233	0.051033	0.005817	0.00782	0.000218	0.036582	0.235409
DR40-8 - 41	16.73	19	0.007479	0.000352	**48.03082**	**2.253067**	0.056759	0.008698	55.85321	8.368842	0.002846	0.000711	57.41756	14.33121	0.055805	0.008912	87.93962	517.5623	135.1335	6.742291	2.591711	0.056759	0.008698	0.007479	0.000352	-0.06764	0.324974
DR40-8 - 42	22.71	19	0.007542	0.000219	**48.43712**	**1.402408**	0.049514	0.005618	48.95169	5.419275	0.002483	0.000317	50.12133	6.382576	0.04609	0.004998	-69.884	285.7117	132.0946	4.341339	1.721036	0.049514	0.005618	0.007542	0.000219	-0.13194	0.359407
DR40-8 - 43	27.00	19	0.007482	0.00022	**48.04959**	**1.409975**	0.057222	0.006074	56.33589	5.820079	0.002425	0.000274	48.95951	5.525318	0.055685	0.00581	376.8348	214.7831	134.2463	3.978314	1.707927	0.057222	0.006074	0.007482	0.00022	0.082943	0.155231
DR40-8 - 44	6.34	19	0.00783	0.000548	**50.27757**	**3.506184**	0.056567	0.008967	55.78957	8.654463	0.003615	0.000785	72.91563	15.8125	0.053092	0.009577	190.083	481.9477	128.8507	8.990388	1.640457	0.056567	0.008967	0.00783	0.000548	-0.12114	0.485249
DR40-8 - 45	27.01	19	0.007753	0.000227	**49.78306**	**1.453099**	0.056101	0.006023	55.2646	5.780452	0.002624	0.000401	52.96082	8.074958	0.05235	0.005247	159.84	262.4382	129.556	3.967309	2.339029	0.056101	0.006023	0.007753	0.000227	0.339586	-0.09574
DR40-8 - 46	27.00	19	0.007801	0.000238	**50.0897**	**1.521394**	0.058073	0.004847	57.2154	4.643915	0.002433	0.000268	49.10646	5.405138	0.054054	0.004288	261.6401	196.893	128.8336	3.896095	1.516317	0.058073	0.004847	0.007801	0.000238	0.242727	0.033942
DR40-8 - 47	12.28	19	0.007748	0.00026	**49.75598**	**1.660753**	0.050526	0.003919	50.01974	3.786187	0.002311	0.000241	46.65544	4.856053	0.047564	0.00382	25.26237	198.4836	129.3819	4.57981	1.268311	0.050526	0.003919	0.007748	0.00026	0.004397	0.359745
DR40-8 - 48	12.61	19	0.008206	0.000321	**52.68335**	**2.053744**	0.059368	0.012628	58.23625	12.16296	0.003147	0.000897	63.47543	18.05021	0.053248	0.011639	28.58996	693.8577	122.3673	4.79598	2.43507	0.059368	0.012628	0.008206	0.000321	-0.34267	0.42572
DR40-8 - 49	17.84	19	0.007526	0.000288	**48.33455**	**1.842788**	0.05356	0.008254	52.78156	7.863236	0.002403	0.000388	48.51287	7.820706	0.052412	0.008855	50.87064	351.9105	133.7606	5.484062	1.996361	0.05356	0.008254	0.007526	0.000288	-0.16609	0.369593
DR40-8 - 50	27.01	19	0.007628	0.000249	**48.98721**	**1.595629**	0.053185	0.003547	52.56424	3.418244	0.002416	0.000208	48.76778	4.201595	0.050809	0.003384	157.0645	161.0513	131.918	4.310358	1.172093	0.053185	0.003547	0.007628	0.000249	0.158533	0.229294
DR40-8 - 51	26.44	19	0.007191	0.000219	**46.18765**	**1.398727**	0.052382	0.003428	51.79288	3.312034	0.002499	0.000257	50.43576	5.175016	0.053184	0.003671	254.5627	169.4166	139.7834	4.44799	1.605069	0.052382	0.003428	0.007191	0.000219	-0.00926	0.380597
DR40-8 - 52	11.39	19	0.007213	0.000274	**46.32729**	**1.753845**	0.048145	0.005139	47.69822	4.963496	0.002693	0.000551	54.34635	11.10156	0.048435	0.004996	44.56103	254.4878	139.0999	5.327704	1.544923	0.048145	0.005139	0.007213	0.000274	0.176307	0.174198
DR40-8 - 53	14.62	19	0.007846	0.000413	**50.37606**	**2.645419**	0.060796	0.007401	59.80195	7.0663	0.002903	0.000657	58.56707	13.25059	0.057057	0.007206	330.0867	351.613	129.1753	7.707316	2.335686	0.060796	0.007401	0.007846	0.000413	-0.01208	0.420838
DR40-8 - 54	23.75	19	0.007433	0.000284	**47.73495**	**1.81871**	0.052252	0.005344	51.60631	5.156237	0.002424	0.000295	48.9348	5.944233	0.051274	0.005276	151.8027	238.3508	135.7955	5.432915	1.595202	0.052252	0.005344	0.007433	0.000284	0.158044	0.188095
DR40-8 - 55	8.13	19	0.007303	0.000465	**46.90753**	**2.974938**	0.044196	0.008369	43.82412	8.139924	0.00248	0.00054	50.05588	10.88929	0.044827	0.009576	-353.327	668.2485	138.2394	9.266384	1.804446	0.044196	0.008369	0.007303	0.000465	-0.24187	0.510163
					sumary stats IsoplotR	**48.81**	**0.36**	0.74%	**1,852.10**	3794.51%																	
**GHR-1**																											
GHR-1 - 1	23.00	19	0.00723	0.000157	**46.4426**	**1.00659**	0.047216	0.001631	46.8382	1.580879	0.002208	0.000143	44.57063	2.893322	0.047334	0.001376	53.69679	69.02469	138.3735	2.993459	1.017472	0.047216	0.001631	0.00723	0.000157	0.271861	0.212306
GHR-1 - 2	27.00	19	0.007528	0.000228	**48.34436**	**1.457625**	0.054357	0.002957	53.71023	2.843248	0.002495	0.000207	50.35535	4.175397	0.052403	0.002224	272.5033	100.8296	133.6777	4.331498	2.048125	0.054357	0.002957	0.007528	0.000228	0.54206	-0.03461
GHR-1 - 3	27.00	19	0.007219	0.000174	**46.36746**	**1.112716**	0.047805	0.002326	47.39454	2.254111	0.002407	0.000201	48.58127	4.044148	0.048198	0.002145	73.53302	109.2369	138.8322	3.436865	2.130679	0.047805	0.002326	0.007219	0.000174	0.223946	0.161594
GHR-1 - 4	25.57	19	0.007721	0.000209	**49.58283**	**1.335669**	0.049361	0.002547	48.89823	2.459442	0.002666	0.000216	53.82028	4.350366	0.046312	0.002132	-20.917	111.0186	129.2017	3.09373	1.142228	0.049361	0.002547	0.007721	0.000209	0.300452	0.148181
GHR-1 - 6	12.07	19	0.007389	0.000222	**47.45504**	**1.419091**	0.051685	0.003538	51.14538	3.417275	0.002475	0.000289	49.95495	5.836253	0.050762	0.003495	192.3155	167.8897	135.5286	4.311261	3.013321	0.051685	0.003538	0.007389	0.000222	0.050269	0.336122
GHR-1 - 7	5.30	19	0.007692	0.000356	**49.39331**	**2.275106**	0.050698	0.005464	50.1892	5.278842	0.002162	0.00027	43.65843	5.455003	0.048004	0.005015	61.13471	249.1714	130.3673	6.291969	1.646287	0.050698	0.005464	0.007692	0.000356	0.260573	0.173492
GHR-1 - 8	27.00	19	0.007278	0.000161	**46.74849**	**1.028042**	0.047164	0.001658	46.7859	1.607552	0.002325	0.000166	46.93263	3.337429	0.0471	0.001534	35.35748	80.01957	137.5657	3.011885	1.643229	0.047164	0.001658	0.007278	0.000161	0.013527	0.400497
GHR-1 - 9	10.21	19	0.007667	0.00025	**49.2361**	**1.600234**	0.04938	0.003155	48.92525	3.052738	0.002421	0.000271	48.87402	5.459866	0.047051	0.003256	21.65514	159.9285	130.8261	4.212426	2.628818	0.04938	0.003155	0.007667	0.00025	0.006972	0.406811
GHR-1 - 10	23.35	19	0.007544	0.000172	**48.45111**	**1.101847**	0.051628	0.00282	51.08662	2.72176	0.002355	0.000178	47.53473	3.595896	0.049751	0.00255	143.9167	123.8662	132.7806	3.098078	2.288699	0.051628	0.00282	0.007544	0.000172	0.166039	0.132901
GHR-1 - 12	27.01	19	0.007665	0.000245	**49.21883**	**1.564687**	0.050107	0.002686	49.61555	2.597849	0.002436	0.000233	49.18106	4.690795	0.04743	0.002092	37.33706	106.8553	131.3578	4.561716	3.019135	0.050107	0.002686	0.007665	0.000245	0.507564	0.008474
GHR-1 - 15	18.99	19	0.007814	0.000179	**50.17308**	**1.145943**	0.052292	0.002444	51.73828	2.360755	0.002677	0.000223	54.04055	4.491263	0.048663	0.002022	109.2955	102.8135	128.0744	2.992729	2.089113	0.052292	0.002444	0.007814	0.000179	0.145034	0.156297
GHR-1 - 16	27.01	19	0.00764	0.000183	**49.06377**	**1.169696**	0.050966	0.002345	50.45372	2.266324	0.002552	0.000197	51.51067	3.975646	0.048843	0.002029	109.5318	101.7976	131.1192	3.238071	2.199206	0.050966	0.002345	0.00764	0.000183	0.3165	0.253335
GHR-1 - 18	22.60	19	0.00746	0.000191	**47.91284**	**1.223243**	0.048075	0.002954	47.6466	2.854715	0.002486	0.000258	50.18581	5.204166	0.046784	0.002769	-12.9205	141.5529	134.3125	3.530173	2.627713	0.048075	0.002954	0.00746	0.000191	0.108385	0.231159
					**48.09**	**0.81**	1.68%	1824.781	3794.51%																		
**Z_GJ-1**																											
GJ-1 - 1	20.47	19	0.096937	0.002041	**596.4076**	**11.99631**	0.803667	0.021116	598.6806	11.88281	0.02894	0.003138	576.1372	61.49627	0.060036	0.000986	601.7896	35.98582	10.31735	0.217456	29.35603	0.803667	0.021116	0.096937	0.002041	0.603419	0.185392
GJ-1 - 2	23.88	19	0.098556	0.002073	**605.9062**	**12.16155**	0.819199	0.021842	607.3306	12.11288	0.030777	0.003274	612.0814	64.12267	0.060251	0.001035	608.8923	37.06775	10.14969	0.212593	29.88825	0.819199	0.021842	0.098556	0.002073	0.545543	0.168415
GJ-1 - 3	18.68	19	0.096297	0.00214	**592.6399**	**12.58066**	0.795866	0.021791	594.2611	12.32833	0.030359	0.003011	604.1207	59.0517	0.059888	0.001329	594.3899	47.9695	10.38877	0.230032	30.0639	0.795866	0.021791	0.096297	0.00214	0.244383	0.459728
GJ-1 - 4	20.61	19	0.099776	0.002148	**613.0651**	**12.5787**	0.822102	0.022419	608.9568	12.53006	0.031343	0.002922	623.4208	57.25914	0.059929	0.001181	596.5322	43.51627	10.03033	0.211776	29.55738	0.822102	0.022419	0.099776	0.002148	0.403971	0.317137
GJ-1 - 5	27.01	19	0.098274	0.002082	**604.2472**	**12.2268**	0.817904	0.020182	606.68	11.29544	0.029076	0.003034	578.7225	59.61525	0.060497	0.001041	617.352	37.36845	10.18633	0.217978	30.02216	0.817904	0.020182	0.098274	0.002082	0.405793	0.458687
GJ-1 - 6	27.65	19	0.098942	0.002077	**608.1682**	**12.18056**	0.824236	0.022041	610.0853	12.22188	0.02987	0.002676	594.5191	52.4709	0.060519	0.001055	617.8565	37.88468	10.11728	0.212323	29.38121	0.824236	0.022041	0.098942	0.002077	0.532443	0.189454
GJ-1 - 7	27.00	19	0.097725	0.001909	**601.0415**	**11.21151**	0.811033	0.021091	602.7626	11.80562	0.029918	0.003026	595.2674	59.37697	0.060399	0.001106	613.2039	39.42244	10.23464	0.200213	29.74679	0.811033	0.021091	0.097725	0.001909	0.326154	0.237169

	Duration (s)	Spot Size (µm)	Final Pb206/U238_mean	Final Pb206/U238_2SE(prop)	Final Pb206/U238 age_mean	Final Pb206/U238 age_2SE(prop)	Final Pb207/U235_mean	Final Pb207/U235_2SE(prop)	Final Pb207/U235 age_mean	Final Pb207/U235 age_2SE(prop)	Final Pb208/Th232_mean	Final Pb208/Th232_2SE(prop)	Final Pb208/Th232 age_mean	Final Pb208/Th232 age_2SE(prop)	Final Pb207/Pb206_mean	Final Pb207/Pb206_2SE(prop)	Final Pb207/Pb206 age_mean	Final Pb207/Pb206 age_2SE(prop)	Final U238/Pb206_mean	Final U238/Pb206_2SE(prop)	Final U/Th_mean	Final Pb207/U235_mean	Final Pb207/U235_2SE(prop)	Final Pb206/U238_mean	Final Pb206/U238_2SE(prop)	rho 206Pb/238U v 207Pb/235U	rho 207Pb/206Pb v 238U/206Pb
GJ-1 - 9	22.71	19	0.097672	0.002141	**600.7106**	**12.56648**	0.80012	0.022469	596.567	12.74099	0.031249	0.003276	621.3595	64.05249	0.059602	0.001143	584.5439	41.84829	10.24751	0.222386	29.8224	0.80012	0.022469	0.097672	0.002141	0.541485	0.163708
GJ-1 - 10	23.83	19	0.098281	0.002006	**604.3035**	**11.7767**	0.804517	0.022413	599.0467	12.60627	0.030645	0.002968	609.6457	58.06319	0.059391	0.001066	577.2982	39.37316	10.17756	0.208662	29.75266	0.804517	0.022413	0.098281	0.002006	0.590324	-0.00266
GJ-1 - 11	20.96	19	0.096551	0.002008	**594.1395**	**11.79535**	0.796544	0.021658	594.619	12.23689	0.030387	0.003512	604.3684	68.74152	0.059799	0.001083	592.4619	39.54494	10.35988	0.213269	29.71962	0.796544	0.021658	0.096551	0.002008	0.515841	0.123064
GJ-1 - 12	27.54	19	0.09895	0.001957	**608.2294**	**11.48106**	0.829088	0.021226	612.8426	11.82548	0.029037	0.002515	578.2222	49.33095	0.060766	0.000961	627.4941	34.11708	10.10768	0.200218	29.68145	0.829088	0.021226	0.09895	0.001957	0.573724	0.03919
GJ-1 - 14	18.87	19	0.097973	0.002178	**602.4855**	**12.78654**	0.820114	0.023402	607.824	13.08642	0.027709	0.003243	551.9376	63.60429	0.060581	0.001223	620.1076	43.98044	10.20599	0.226882	29.42529	0.820114	0.023402	0.097973	0.002178	0.487477	0.229598
GJ-1 - 15	16.41	19	0.096624	0.002427	**594.5474**	**14.27053**	0.806099	0.021224	600.0942	11.97019	0.03233	0.002971	642.846	58.04787	0.060332	0.001115	612.3018	39.91624	10.35509	0.263317	30.09042	0.806099	0.021224	0.096624	0.002427	0.56845	0.513057
GJ-1 - 16	27.01	19	0.096866	0.002158	**595.9661**	**12.6869**	0.802157	0.020942	597.7777	11.78876	0.02874	0.002699	572.2992	52.94808	0.06002	0.000971	600.7722	34.88284	10.32672	0.232244	29.60952	0.802157	0.020942	0.096866	0.002158	0.633708	0.278979
GJ-1 - 17	27.01	19	0.097759	0.002278	**601.1995**	**13.36192**	0.808848	0.023597	601.3669	13.22749	0.032717	0.003051	650.1904	59.70348	0.059946	0.001043	597.4638	37.74064	10.23656	0.234461	29.72163	0.808848	0.023597	0.097759	0.002278	0.719438	0.034612
GJ-1 - 18	27.01	19	0.098252	0.002046	**604.1242**	**12.01388**	0.818345	0.021434	606.848	11.93777	0.029394	0.002646	585.1972	51.89322	0.06037	0.001078	612.5517	38.20489	10.17462	0.213561	29.77213	0.818345	0.021434	0.098252	0.002046	0.457336	0.290977
GJ-1 - 19	23.34	19	0.097831	0.002092	**601.6479**	**12.27902**	0.805836	0.024239	599.6846	13.58849	0.029414	0.002713	585.6082	53.20931	0.059718	0.001217	588.1783	44.10231	10.223	0.217773	29.65342	0.805836	0.024239	0.097831	0.002092	0.574275	-0.01662
GJ-1 - 21	27.01	19	0.098594	0.002094	**606.1246**	**12.28294**	0.821679	0.021963	608.679	12.16718	0.031594	0.003359	627.9946	65.57082	0.060494	0.001108	616.692	39.43041	10.14532	0.214892	29.98842	0.821679	0.021963	0.098594	0.002094	0.466678	0.292869
GJ-1 - 22	22.37	19	0.097524	0.0021	**599.8491**	**12.33002**	0.803355	0.020682	598.5144	11.63519	0.030317	0.002968	603.2524	58.35397	0.059752	0.001056	590.9282	37.97156	10.25759	0.22023	29.53853	0.803355	0.020682	0.097524	0.0021	0.468738	0.35973
GJ-1 - 24	27.01	19	0.098133	0.001943	**603.4319**	**11.41013**	0.81958	0.019959	607.6259	11.19504	0.029771	0.002996	592.393	58.81662	0.060557	0.000871	620.7602	31.24695	10.19007	0.203546	29.66573	0.81958	0.019959	0.098133	0.001943	0.592758	0.125872
GJ-1 - 25	27.02	19	0.09775	0.001988	**601.1779**	**11.67396**	0.803494	0.021968	598.454	12.40972	0.029894	0.002881	594.8884	56.50962	0.059712	0.001109	588.2575	40.6635	10.24015	0.20876	29.3235	0.803494	0.021968	0.09775	0.001988	0.495042	0.095309
GJ-1 - 26	27.01	19	0.097911	0.002093	**602.1161**	**12.29284**	0.811482	0.019296	603.1287	10.83936	0.029714	0.003076	591.2307	60.28961	0.059988	0.000937	599.782	34.19898	10.22816	0.222011	29.94663	0.811482	0.019296	0.097911	0.002093	0.576041	0.287314
GJ-1 - 27	27.01	19	0.097948	0.001999	**602.3434**	**11.74163**	0.815447	0.02162	605.2118	12.08203	0.030387	0.002983	604.5225	58.44559	0.060488	0.001053	616.9665	37.53356	10.19325	0.187485	29.99738	0.815447	0.02162	0.097948	0.001999	0.523089	0.128595
GJ-1 - 28	27.02	19	0.097105	0.002081	**597.3832**	**12.22963**	0.80602	0.022634	599.833	12.74239	0.029673	0.002605	590.694	51.04802	0.060105	0.001039	603.1876	37.50844	10.28515	0.246675	29.71904	0.80602	0.022634	0.097105	0.002081	0.676228	0.108757
GJ-1 - 29	18.82	19	0.097843	0.002351	**601.7038**	**13.80879**	0.811094	0.026276	602.6124	14.82026	0.030692	0.003397	610.483	66.49336	0.06018	0.001312	604.9675	48.22432	10.24258	0.248045	30.20955	0.811094	0.026276	0.097843	0.002351	0.631662	0.015146
GJ-1 - 30	19.77	19	0.098231	0.002192	**603.9962**	**12.86323**	0.814336	0.023713	604.5628	13.28258	0.031294	0.00317	622.3856	61.96717	0.060322	0.001352	609.7741	48.29463	10.19659	0.22581	29.57976	0.814336	0.023713	0.098231	0.002192	0.37646	0.303618
GJ-1 - 31	24.37	19	0.098036	0.002117	**602.8482**	**12.42605**	0.816232	0.022177	605.6455	12.43649	0.030279	0.003348	602.2566	65.7115	0.060415	0.001054	614.6102	38.12067	10.20817	0.221134	29.29576	0.816232	0.022177	0.098036	0.002117	0.594435	0.157155
GJ-1 - 32	23.00	19	0.097226	0.001978	**598.1065**	**11.61697**	0.803122	0.02146	598.3268	12.05216	0.028413	0.003058	565.7568	60.05709	0.059938	0.001168	596.5922	42.28476	10.28783	0.208944	29.62386	0.803122	0.02146	0.097226	0.001978	0.30806	0.297098
GJ-1 - 33	27.00	19	0.098208	0.001885	**603.8796**	**11.0648**	0.816092	0.019908	605.6774	11.13563	0.030638	0.002956	609.4521	57.89536	0.060308	0.000929	611.3769	33.78952	10.18183	0.195687	30.02766	0.816092	0.019908	0.098208	0.001885	0.444757	0.18784
GJ-1 - 34	21.64	19	0.097725	0.00199	**601.0409**	**11.68437**	0.81456	0.02181	604.7617	12.23317	0.030545	0.002769	607.8213	54.26738	0.060463	0.001181	615.7578	42.28894	10.23148	0.207809	29.12267	0.81456	0.02181	0.097725	0.00199	0.306889	0.295241
GJ-1 - 35	17.78	19	0.0994	0.002114	**610.8655**	**12.40202**	0.813185	0.02135	604.0493	12.03438	0.029213	0.003315	581.505	65.04278	0.059366	0.001233	576.1301	45.31139	10.06036	0.215999	29.97209	0.813185	0.02135	0.0994	0.002114	0.222235	0.46723
GJ-1 - 36	27.00	19	0.097479	0.002044	**599.5848**	**12.02421**	0.81168	0.021269	603.1126	11.94926	0.030652	0.003198	609.5818	62.59072	0.060258	0.001022	608.8211	37.05661	10.26301	0.223034	29.86996	0.81168	0.021269	0.097479	0.002044	0.79397	-0.24487
GJ-1 - 37	27.00	19	0.09706	0.001948	**597.1306**	**11.4411**	0.802238	0.019221	597.9279	10.80208	0.028138	0.002898	560.3456	57.02719	0.059935	0.000845	598.6077	30.24978	10.30325	0.205285	29.33158	0.802238	0.019221	0.09706	0.001948	0.534423	0.187146
GJ-1 - 38	27.01	19	0.097223	0.002061	**598.0764**	**12.10569**	0.809081	0.023928	601.4563	13.53219	0.031047	0.003172	617.3634	62.05889	0.060301	0.001216	608.4699	44.62577	10.29088	0.216787	30.20415	0.809081	0.023928	0.097223	0.002061	0.562971	-0.02333
GJ-1 - 39	27.00	19	0.09847	0.002025	**605.4079**	**11.8853**	0.820606	0.020442	608.1748	11.42694	0.031424	0.003146	624.7877	61.58972	0.060676	0.00108	623.3962	38.74756	10.1576	0.209484	29.64563	0.820606	0.020442	0.09847	0.002025	0.447934	0.263642
GJ-1 - 40	13.90	19	0.097841	0.002431	**601.706**	**14.27159**	0.808861	0.024267	601.5705	13.55944	0.032078	0.004125	637.5511	80.82253	0.060057	0.001337	601.6978	47.71517	10.22749	0.254961	29.37702	0.808861	0.024267	0.097841	0.002431	0.497475	0.324992
GJ-1 - 41	27.01	19	0.097773	0.002009	**601.3152**	**11.79911**	0.816278	0.022663	605.5973	12.66351	0.031262	0.003342	621.474	65.37352	0.060486	0.001023	617.1293	35.92191	10.23109	0.211826	29.97422	0.816278	0.022663	0.097773	0.002009	0.666192	-0.12585
GJ-1 - 42	13.68	19	0.098859	0.002003	**607.7033**	**11.74673**	0.813121	0.022553	604.0216	12.69902	0.028253	0.003234	562.7767	63.58928	0.059587	0.00115	585.5342	41.95041	10.11173	0.203299	29.85804	0.813121	0.022553	0.098859	0.002003	0.444105	0.110639
GJ-1 - 43	27.00	19	0.098131	0.002054	**603.4139**	**12.05126**	0.806858	0.022771	600.2911	12.90043	0.029632	0.002473	589.9502	48.61582	0.0598	0.001079	591.7633	39.30716	10.19537	0.212225	29.58271	0.806858	0.022771	0.098131	0.002054	0.630337	-0.03675
GJ-1 - 44	27.00	19	0.099206	0.002119	**609.7146**	**12.42752**	0.819533	0.022172	607.4628	12.36962	0.029662	0.002893	590.3273	56.76214	0.060108	0.001044	603.3676	37.36859	10.08657	0.216243	29.6411	0.819533	0.022172	0.099206	0.002119	0.57507	0.135723
GJ-1 - 45	27.00	19	0.096278	0.001938	**592.5332**	**11.38567**	0.807428	0.023114	600.5907	12.99897	0.030891	0.00304	614.3773	59.57119	0.061012	0.001254	633.6513	44.50017	10.38807	0.206136	29.68444	0.807428	0.023114	0.096278	0.001938	0.421212	0.088126
GJ-1 - 46	27.00	19	0.097681	0.001947	**600.7804**	**11.44057**	0.809831	0.021386	602.0675	11.95767	0.030921	0.003	614.9764	58.74353	0.060186	0.001072	605.8757	38.2007	10.23691	0.206288	29.76579	0.809831	0.021386	0.097681	0.001947	0.433642	0.154826
GJ-1 - 47	22.86	19	0.098841	0.002014	**607.5912**	**11.80855**	0.81906	0.023334	609.6038	11.36731	0.028979	0.003313	576.763	64.95925	0.059996	0.001236	598.0401	46.40527	10.08576	0.232509	29.94822	0.81906	0.023334	0.098841	0.002014	0.434439	0.190272
GJ-1 - 48	17.65	19	0.097412	0.002504	**599.1666**	**14.68897**	0.812277	0.024178	603.4182	13.57955	0.03072	0.003544	611.0102	69.48633	0.060637	0.001288	621.9654	45.56064	10.27839	0.258266	29.52308	0.812277	0.024178	0.097412	0.002504	0.579854	0.351094
GJ-1 - 49	27.01	19	0.097472	0.002038	**599.5421**	**11.96862**	0.797788	0.022374	595.1998	12.65274	0.030876	0.003277	613.9395	64.23412	0.05945	0.001031	579.3869	37.88347	10.26194	0.214859	29.58438	0.797788	0.022374	0.097472	0.002038	0.577101	-0.08818
GJ-1 - 51	27.00	19	0.097646	0.002044	**600.5649**	**12.00291**	0.815716	0.021036	605.3927	11.80777	0.030141	0.002867	599.7353	56.21435	0.060711	0.001086	624.5577	38.69418	10.24367	0.214112	29.84557	0.815716	0.021036	0.097646	0.002044	0.422853	0.327921
GJ-1 - 52	27.00	19	0.098895	0.002038	**607.9003**	**11.96274**	0.825368	0.022506	610.6875	12.59913	0.028327	0.002535	564.2574	49.60481	0.060532	0.0011	618.032	39.15033	10.1122	0.212943	29.7409	0.825368	0.022506	0.098895	0.002038	0.523978	0.085249
GJ-1 - 53	27.02	19	0.096197	0.002073	**592.0397**	**12.18627**	0.801423	0.021678	597.3059	12.22845	0.030085	0.002866	598.6441	56.26265	0.060455	0.001187	614.5298	41.76552	10.39981	0.222998	29.65744	0.801423	0.021678	0.096197	0.002073	0.418126	0.319501
GJ-1 - 54	27.00	19	0.098523	0.002086	**605.7095**	**12.23577**	0.812633	0.021032	603.6642	11.79986	0.032668	0.003514	648.9289	68.53862	0.05979	0.001009	592.0303	36.40625	10.15253	0.21321	29.81449	0.812633	0.021032	0.098523	0.002086	0.302871	0.701919
GJ-1 - 55	15.55	19	0.097443	0.002286	**599.3727**	**13.41676**	0.812814	0.021983	603.8478	12.25037	0.029088	0.003916	578.884	76.5199	0.060491	0.001247	617.2781	45.41153	10.26716	0.237256	29.34	0.812814	0.021983	0.097443	0.002286	0.399889	0.489162
GJ-1 - 56	27.00	19	0.097767	0.001884	**601.2919**	**11.06038**	0.799283	0.020059	596.2098	11.33701	0.029327	0.003003	583.6786	58.9111	0.059308	0.001023	574.2501	38.11819	10.22589	0.195613	29.89178	0.799283	0.020059	0.097767	0.001884	0.352345	0.31167
GJ-1 - 57	21.22	19	0.098366	0.002065	**604.7977**	**12.11774**	0.827214	0.021649	611.842	12.00854	0.032318	0.003269	642.3601	64.06133	0.060889	0.001062	631.7745	37.93866	10.16801	0.213407	29.70061	0.827214	0.021649	0.098366	0.002065	0.462321	0.255655
**DR40-Z**																											
DR40-Z_001	27.004	19	0.00783	0.000136	**50.28072**	**0.867498**	0.050822	0.004944	50.22419	4.779214	0.002366	0.000313	47.75513	6.306514	0.04658	0.004186	127.1916	208.8182	127.534	2.376551	1.297021	0.050822	0.004944	0.00783	0.000136	0.219127	-0.07227
DR40-Z_002	27.011	19	0.007748	0.000248	**49.74988**	**1.586324**	0.047551	0.006128	47.00033	5.92196	0.002708	0.00054	54.64352	10.88466	0.044762	0.005617	-160.937	357.0895	130.1621	4.184653	2.419574	0.047551	0.006128	0.007748	0.000248	0.220916	0.002698
DR40-Z_003	27.003	19	0.007523	0.00021	**48.31124**	**1.346789**	0.047636	0.006343	48.44813	6.625042	0.002377	0.000433	47.97623	8.722684	0.045771	0.006069	19.36386	355.7297	132.5204	3.713931	2.160975	0.047636	0.006343	0.007523	0.00021	-0.12831	0.342279
DR40-Z_004	15.87181	19	0.007873	0.00022	**50.55078**	**1.405221**	0.058595	0.006044	57.72727	5.790023	0.002274	0.000366	45.89743	7.380543	0.055552	0.006111	443.1274	212.651	126.6232	3.926873	1.7907	0.058595	0.006044	0.007873	0.00022	0.136293	0.264949
DR40-Z_005	27.01	19	0.007478	0.000337	**48.02045**	**2.156205**	0.045589	0.009149	46.5772	8.34858	0.002187	0.000479	44.13518	9.660101	0.043655	0.00908	77.97631	351.1618	136.0938	5.855406	2.404729	0.045589	0.009149	0.007478	0.000337	-0.06286	0.323498
DR40-Z_006	27.004	19	0.00745	0.000327	**47.84537**	**2.095119**	0.048728	0.007528	48.05299	7.289533	0.001836	0.000611	37.04944	12.31443	0.051092	0.008491	130.4006	361.237	133.1033	5.226763	2.491562	0.048728	0.007528	0.00745	0.000327	0.038437	0.132505
DR40-Z_007	27.006	19	0.007726	0.000278	**49.6134**	**1.780561**	0.053536	0.005673	52.81248	5.459499	0.002425	0.000557	48.92496	11.22517	0.047411	0.0064	184.4372	296.6536	130.036	4.369297	2.503503	0.053536	0.005673	0.007726	0.000278	-0.24528	0.517113
DR40-Z_008	27.005	19	0.007502	0.000189	**48.17737**	**1.206406**	0.049702	0.006069	49.08277	5.875456	0.002465	0.000516	49.73174	10.39476	0.047748	0.005444	53.36046	300.0585	133.2986	3.530614	2.283367	0.049702	0.006069	0.007502	0.000189	0.254629	0.003242
DR40-Z_009	27.004	19	0.007601	0.000226	**48.81422**	**1.442936**	0.047607	0.004724	47.12481	4.570029	0.002302	0.000367	46.45996	7.403829	0.045858	0.004531	-49.838	234.0037	132.4863	3.993182	1.921412	0.047607	0.004724	0.007601	0.000226	0.031192	0.188745
DR40-Z_010	21.65457	19	0.007497	0.000244	**48.14796**	**1.562996**	0.041432	0.008933	40.92475	8.718358	0.002266	0.000581	45.72494	11.72391	0.041254	0.009376	-88.4389	509.4755	134.3151	4.383845	2.098258	0.041432	0.008933	0.007497	0.000244	-0.07235	0.324943
DR40-Z_011	27.008	19	0.007368	0.000272	**47.32169**	**1.738572**	0.053578	0.007911	52.71383	7.614937	0.002293	0.000482	46.28232	9.714287	0.052564	0.007575	404.7592	263.036	134.7979	5.296762	1.906883	0.053578	0.007911	0.007368	0.000272	0.153068	0.054801
DR40-Z_012	27.004	19	0.007769	0.000234	**49.88408**	**1.497682**	0.048033	0.004546	47.54506	4.406969	0.002567	0.000387	51.80286	7.807659	0.044819	0.00428	-59.3461	241.5301	128.4924	3.970554	1.569836	0.048033	0.004546	0.007769	0.000234	0.181945	0.086731
DR40-Z_013	27.006	19	0.007795	0.000226	**50.05409**	**1.444027**	0.054937	0.005255	54.18281	5.04187	0.002588	0.000322	52.23257	6.492611	0.049816	0.00504	153.0967	239.3728	127.4404	3.803392	1.376857	0.054937	0.005255	0.007795	0.000226	0.006266	0.285904
DR40-Z_014	27.004	19	0.007319	0.00025	**47.009**	**1.598818**	0.057975	0.008509	56.90499	8.093549	0.002163	0.000384	43.66088	7.736823	0.059216	0.009321	390.8411	356.4415	135.6405	4.403326	1.648308	0.057975	0.008509	0.007319	0.00025	-0.05349	0.292544
DR40-Z_015	27.004	19	0.007589	0.000232	**48.73354**	**1.486924**	0.051191	0.007269	50.45449	7.00659	0.00264	0.000448	53.27122	9.036461	0.0498	0.006714	-41.5348	352.7654	131.9663	3.846611	1.617363	0.051191	0.007269	0.007589	0.000232	0.536931	-0.28515
DR40-Z_016	27.007	19	0.007612	0.00034	**48.8812**	**2.17764**	0.051987	0.007999	51.17158	7.72083	0.002567	0.0007	51.77419	14.10901	0.048692	0.007997	309.8735	350.0388	131.5083	5.502702	2.934968	0.051987	0.007999	0.007612	0.00034	-0.19151	0.47056

	Duration (s)	Spot Size (µm)	Final Pb206/U238_mean	Final Pb206/U238_2SE(prop)	Final Pb206/U238 age_mean	Final Pb206/U238 age_2SE(prop)	Final Pb207/U235_mean	Final Pb207/U235_2SE(prop)	Final Pb207/U235 age_mean	Final Pb207/U235 age_2SE(prop)	Final Pb208/Th232_mean	Final Pb208/Th232_2SE(prop)	Final Pb208/Th232 age_mean	Final Pb208/Th232 age_2SE(prop)	Final Pb207/Pb206_mean	Final Pb207/Pb206_2SE(prop)	Final Pb207/Pb206 age_mean	Final Pb207/Pb206 age_2SE(prop)	Final U238/Pb206_mean	Final U238/Pb206_2SE(prop)	Final U/Th_mean	Final Pb207/U235_mean	Final Pb207/U235_2SE(prop)	Final Pb206/U238_mean	Final Pb206/U238_2SE(prop)	rho 206Pb/238U v 207Pb/235U	rho 207Pb/206Pb v 238U/206Pb
DR40-Z_017	27.006	19	0.007458	0.00031	47.89468	1.983607	0.034074	0.00794	35.83379	8.652233	0.002859	0.000646	57.66033	13.007	0.033088	0.007523	-254.227	510.2497	136.0685	5.412583	1.921228	0.034074	0.00794	0.007458	0.00031	-0.19994	0.294845
DR40-Z_018	27.004	19	0.007678	0.00025	49.30196	1.597451	0.048067	0.006937	47.45153	6.712528	0.002378	0.000541	47.98116	10.89822	0.044906	0.006544	-130.986	382.5874	130.6332	4.17848	2.425331	0.048067	0.006937	0.007678	0.00025	0.200859	0.007183
DR40-Z_019	27.012	19	0.007709	0.000315	49.5002	2.014256	0.054968	0.009101	53.96154	8.758693	0.00211	0.000586	42.56462	11.82557	0.05073	0.007994	322.066	378.0656	129.9034	5.316591	2.15975	0.054968	0.009101	0.007709	0.000315	0.06339	0.14393
DR40-Z_020	18.9939	19	0.008003	0.000284	51.3856	1.817809	0.048921	0.008217	48.28173	7.944578	0.002813	0.000639	56.7471	12.88391	0.044848	0.007562	-30.3877	360.0689	125.8631	4.436054	2.208573	0.048921	0.008217	0.008003	0.000284	-0.13416	0.328203
DR40-Z_021	27.004	19	0.007217	0.000245	46.35696	1.566485	0.053334	0.004807	52.65839	4.631767	0.002688	0.000477	54.24495	9.619069	0.054169	0.005699	344.0458	251.5087	139.8892	4.765112	2.27353	0.053334	0.004807	0.007217	0.000245	0.121743	0.210579
DR40-Z_022	27.004	19	0.007721	0.000254	49.57799	1.624388	0.051918	0.00725	52.28303	7.286384	0.002435	0.00059	49.12781	11.89348	0.04885	0.006678	53.87533	359.2754	130.0472	4.117514	1.993632	0.051918	0.00725	0.007721	0.000254	0.071444	0.108803
DR40-Z_023	27.005	19	0.007485	0.000193	48.06817	1.23353	0.055285	0.003717	54.58041	3.574624	0.002366	0.000304	48.54555	6.438196	0.052551	0.003953	297.1891	185.562	133.7545	3.586754	1.630126	0.055285	0.003717	0.007485	0.000193	-0.05743	0.332582
DR40-Z_024	27.007	19	0.007783	0.000274	49.97408	1.750998	0.043332	0.006411	44.20817	6.668528	0.002546	0.000478	51.37921	9.634059	0.04374	0.007116	-184.604	378.7646	129.8769	4.725995	1.608828	0.043332	0.006411	0.007783	0.000274	0.030778	0.178802
DR40-Z_025	27.004	19	0.007662	0.000269	49.20524	1.721448	0.046203	0.007572	45.60065	7.334281	0.002621	0.000615	52.88421	12.38631	0.043439	0.0069	-2.585	372.9301	131.8313	4.539938	2.250892	0.046203	0.007572	0.007662	0.000269	-0.10596	0.309684
DR40-Z_026	27.004	19	0.007901	0.000177	50.73361	1.133377	0.055037	0.006722	54.19901	6.470795	0.002695	0.000405	54.39507	8.163712	0.050446	0.006161	251.0347	283.5891	125.3767	3.051582	1.703327	0.055037	0.006722	0.007901	0.000177	0.074978	0.109731
DR40-Z_027	27.006	19	0.007658	0.000335	49.17378	2.144025	0.04463	0.007447	44.08045	7.229211	0.00232	0.000572	46.80899	11.52524	0.040063	0.007011	-307.831	444.5295	133.7702	4.397929	2.667114	0.04463	0.007447	0.007658	0.000335	0.152779	0.330613
DR40-Z_028	27.006	19	0.00741	0.000195	47.59131	1.249618	0.058817	0.007947	57.75192	7.607724	0.002819	0.000519	56.87244	10.45231	0.056741	0.007433	473.9325	293.9907	134.9446	3.914035	1.910125	0.058817	0.007947	0.00741	0.000195	0.173848	0.059718
DR40-Z_030	27.006	19	0.007845	0.000247	50.37137	1.577695	0.043939	0.006521	43.46891	6.312245	0.002745	0.000564	55.3779	11.35753	0.042319	0.006806	-226.939	401.4547	127.8046	4.208299	2.447315	0.043939	0.006521	0.007845	0.000247	-0.2804	0.493202
DR40-Z_031	27.004	19	0.007723	0.000206	49.59556	1.317532	0.054973	0.005493	55.21399	5.592059	0.00258	0.000431	52.06909	8.693722	0.052441	0.005438	236.0762	236.7482	130.1465	3.564536	1.785672	0.054973	0.005493	0.007723	0.000206	0.04329	0.162836
DR40-Z_032	27.004	19	0.007391	0.00025	47.46862	1.602168	0.049421	0.006397	48.79782	6.151564	0.002259	0.000406	45.59384	8.182726	0.04846	0.006184	-125.739	324.984	135.7739	4.353535	2.329247	0.049421	0.006397	0.007391	0.00025	0.170082	-0.16212
DR40-Z_033	27.006	19	0.007718	0.000365	49.55731	2.334909	0.048612	0.007671	49.11635	7.106486	0.002365	0.000478	47.73564	9.631901	0.045557	0.00765	-164.224	404.762	131.0616	6.039545	1.806617	0.048612	0.007671	0.007718	0.000365	-0.12676	0.412445
DR40-Z_034	27.006	19	0.007592	0.000225	48.75622	1.440418	0.057117	0.004872	56.29716	4.680361	0.002382	0.000375	48.07694	7.564267	0.055348	0.004397	355.9541	207.971	132.5699	3.858334	1.234906	0.057117	0.004872	0.007592	0.000225	-0.02155	0.297211
DR40-Z_035	27.012	19	0.00721	0.000259	46.31128	1.657229	0.057653	0.007624	56.65545	7.327669	0.002364	0.000399	47.7131	8.055429	0.056256	0.007271	315.3979	342.2918	139.0824	4.657508	1.570704	0.057653	0.007624	0.00721	0.000259	0.265993	0.206477
DR40-Z_036	27.005	19	0.007862	0.000265	50.48015	1.692677	0.052483	0.009453	51.53699	9.069382	0.002058	0.000377	41.54174	7.593684	0.051998	0.009999	181.8648	369.1743	128.3895	4.436721	1.768588	0.052483	0.009453	0.007862	0.000265	-0.1479	0.411541
DR40-Z_037	11.46933	19	0.007802	0.000366	50.0978	2.338931	0.050415	0.012364	49.64269	12.03178	0.002848	0.000894	57.44382	18.02291	0.047886	0.011751	491.5057	387.0499	129.1935	5.974073	1.78128	0.050415	0.012364	0.007802	0.000366	0.257407	-0.08856
DR40-Z_038	22.94936	19	0.007511	0.000271	48.23471	1.736327	0.060427	0.008015	59.33412	7.647427	0.002662	0.000464	53.71221	9.358406	0.053636	0.007728	251.3033	370.9945	131.7202	4.515084	2.126384	0.060427	0.008015	0.007511	0.000271	-0.18819	0.419996
DR40-Z_039	14.20449	19	0.007912	0.000471	50.79721	3.011169	0.047053	0.019532	45.78218	18.65504	0.003309	0.000986	66.72146	19.85141	0.045556	0.019395	114.0782	1072.669	128.8025	8.435999	1.754399	0.047053	0.019532	0.007912	0.000471	-0.031	0.115802
DR40-Z_040	27.005	19	0.007311	0.000275	46.9558	1.757071	0.041976	0.008375	41.43055	8.08618	0.002248	0.00041	45.36497	8.264057	0.041266	0.007794	-312.438	406.544	137.6007	5.127781	2.183164	0.041976	0.008375	0.007311	0.000275	0.039376	-0.00667
DR40-Z_041	27.006	19	0.007783	0.000202	49.98007	1.294612	0.054411	0.005127	53.68241	4.93232	0.002273	0.000306	45.89398	6.167443	0.051298	0.00513	74.75446	252.8895	129.0791	3.497473	1.366882	0.054411	0.005127	0.007783	0.000202	0.237743	-0.12955
DR40-Z_042	11.91467	19	0.007703	0.000418	49.46391	2.67675	0.053506	0.017089	52.33408	16.35487	0.002181	0.001062	43.99515	21.40686	0.048905	0.015376	130.9049	737.1483	129.6391	7.616641	2.421889	0.053506	0.017089	0.007703	0.000418	0.104738	-0.05342
DR40-Z_043	9.103217	19	0.007689	0.000284	49.37401	1.816247	0.050501	0.009815	49.88319	9.470363	0.002032	0.000376	41.02818	7.584996	0.047958	0.00922	55.7414	398.0772	130.4792	4.782173	1.195789	0.050501	0.009815	0.007689	0.000284	0.107495	0.086407
DR40-Z_044	29.45658	19	0.007921	0.00022	50.86195	1.407775	0.053292	0.004702	52.61579	4.515728	0.002775	0.000353	56.00295	7.112436	0.048332	0.00388	110.0154	199.5937	127.0033	3.635873	1.867068	0.053292	0.004702	0.007921	0.00022	0.000899	0.285057
DR40-Z_045	27.01	19	0.007391	0.000298	47.46822	1.909839	0.042279	0.00553	41.90721	5.377493	0.00267	0.000726	53.84517	14.6204	0.041627	0.005554	-335.283	382.6988	136.2363	5.382185	2.595357	0.042279	0.00553	0.007391	0.000298	0.038257	0.220038
DR40-Z_046	27.007	19	0.007504	0.000429	48.18444	2.747985	0.054698	0.013133	53.28358	12.51945	0.002013	0.000686	40.5979	13.82438	0.056895	0.013123	722.4228	444.7786	136.148	7.886484	2.358861	0.054698	0.013133	0.007504	0.000429	0.258674	-0.00058
DR40-Z_047	27.006	19	0.007434	0.000247	47.74364	1.580739	0.04872	0.006109	48.13365	5.926139	0.002051	0.000404	41.39596	8.154029	0.047434	0.00567	51.84246	288.5063	133.3808	4.640139	1.930595	0.04872	0.006109	0.007434	0.000247	0.163237	0.124438
DR40-Z_048	8.27786	19	0.007696	0.000561	49.41788	3.591008	0.054006	0.011934	53.23202	11.46451	0.002846	0.000762	57.43184	15.34528	0.055591	0.013337	157.002	604.3845	128.3786	8.413785	1.107374	0.054006	0.011934	0.007696	0.000561	0.301387	-0.05687
DR40-Z_049	27.004	19	0.007501	0.000216	48.17527	1.379845	0.044067	0.006502	43.59338	6.324865	0.002378	0.000482	47.99174	9.721568	0.043605	0.006616	-183.226	466.3285	133.4452	3.997402	2.042965	0.044067	0.006502	0.007501	0.000216	-0.04897	0.196936
DR40-Z_050	24.4655	19	0.007354	0.000268	47.2319	1.714466	0.054299	0.009108	53.35505	8.72401	0.002728	0.000575	55.03924	11.59154	0.055294	0.009995	207.1192	408.7932	134.9549	5.034478	2.369281	0.054299	0.009108	0.007354	0.000268	-0.39587	0.581712
DR40-Z_051	22.17464	19	0.007234	0.000261	46.46692	1.671369	0.054955	0.015449	53.45708	14.50141	0.002115	0.000459	42.68097	9.266319	0.054818	0.014642	370.8065	519.9245	138.4701	5.214582	1.612757	0.054955	0.015449	0.007234	0.000261	0.397121	-0.30324
				summary stats IsoplotR	48.93	0.37	0.76%	0.4	0.76%																		
**Rak-17**																											
Rak-17_001	12.23509	19	0.045329	0.001795	285.7409	11.07467	0.334361	0.023524	292.233	17.88708	0.013898	0.001589	278.9416	31.6673	0.054532	0.004175	350.7378	174.9923	21.96745	1.006725	1.328791	0.334361	0.023524	0.045329	0.001795	0.349918	0.229876
Rak-17_002	27.004	19	0.047702	0.000835	300.39	5.138972	0.333241	0.012221	291.7225	9.31194	0.015251	0.001499	305.9077	29.85479	0.050909	0.001694	218.3241	79.81877	20.9892	0.369903	0.92892	0.333241	0.012221	0.047702	0.000835	0.065634	0.295388
Rak-17_003	27.004	19	0.045471	0.000796	286.65	4.909647	0.320322	0.01869	281.2377	14.38417	0.014058	0.001541	282.1125	30.7207	0.051281	0.003037	268.0188	130.3001	22.01182	0.390843	2.349251	0.320322	0.01869	0.045471	0.000796	-0.06338	0.268636
Rak-17_004	20.76371	19	0.046627	0.001044	293.765	6.428335	0.335598	0.018465	293.1657	14.05471	0.014772	0.001549	296.3624	30.8491	0.05158	0.002644	233.2103	120.4319	21.4183	0.497175	0.948269	0.335598	0.018465	0.046627	0.001044	0.298068	0.013197
Rak-17_006	27.012	19	0.046862	0.000983	295.2071	6.051863	0.334973	0.020614	292.2694	15.55305	0.014514	0.001653	291.1616	32.91799	0.050768	0.002871	247.8546	151.5827	21.39633	0.447856	1.471353	0.334973	0.020614	0.046862	0.000983	-0.05376	0.340714
Rak-17_007	27.006	19	0.046133	0.000869	290.7245	5.352173	0.330533	0.015695	291.1708	12.44557	0.014114	0.001506	283.2228	29.99727	0.051969	0.002639	278.0531	117.8253	21.55554	0.465345	2.077343	0.330533	0.015695	0.046133	0.000869	-0.16846	0.483695
Rak-17_008	27.99909	19	0.045832	0.0012	288.8497	7.393807	0.330012	0.024493	287.9587	18.42882	0.013902	0.00154	278.9829	30.69904	0.052829	0.003933	295.0481	191.0425	21.82628	0.58613	0.813694	0.330012	0.024493	0.045832	0.0012	0.125682	0.124199
Rak-17_009	5.883008	19	0.046056	0.002791	290.2207	17.18588	0.327047	0.046995	286.0876	35.30354	0.016698	0.004265	334.4005	84.91994	0.051793	0.005968	235.4249	250.8208	21.85365	1.281142	7.945018	0.327047	0.046995	0.046056	0.002791	0.553972	-0.27003
Rak-17_010	27.006	19	0.045398	0.000882	286.1923	5.437519	0.320029	0.014629	281.4064	11.26855	0.014914	0.001643	299.1467	32.71644	0.051308	0.002148	247.2043	91.25534	22.07459	0.436471	2.140589	0.320029	0.014629	0.045398	0.000882	0.273115	0.008679
Rak-17_011	27.01	19	0.047059	0.000916	296.4262	5.636886	0.354388	0.018588	309.3315	13.34893	0.014331	0.001619	287.5212	32.26248	0.053574	0.002455	375.0253	106.8758	21.29014	0.41211	2.065707	0.354388	0.018588	0.047059	0.000916	0.214384	0.078041
Rak-17_012	27.011	19	0.046566	0.001076	293.3791	6.624126	0.3486	0.022776	302.3334	17.21255	0.014646	0.001659	293.8027	33.06055	0.054146	0.003248	346.8978	136.1784	21.47337	0.518808	1.60241	0.3486	0.022776	0.046566	0.001076	0.289514	0.011656
				sumary stats IsoplotR	292.16	3.39	1.16%	3.4	1.16%																		
**Z_91500**																											
91500_001	18.31847	19	0.178143	0.003047	1056.758	16.6763	1.865756	0.057033	1068.181	20.26002	0.053598	0.005527	1054.986	105.9868	0.076515	0.002141	1101.558	56.71613	5.614582	0.096361	2.499716	1.865756	0.057033	0.178143	0.003047	0.295256	0.148107
91500_002	16.85681	19	0.180728	0.003613	1070.841	19.74856	1.87688	0.058346	1072.137	20.51163	0.053402	0.006099	1050.851	117.0586	0.07487	0.001941	1059.413	51.96649	5.54053	0.112807	2.527208	1.87688	0.058346	0.180728	0.003613	0.381472	0.237556
91500_003	21.82749	19	0.176175	0.003391	1045.926	18.5823	1.833072	0.057775	1056.284	20.78461	0.052249	0.005656	1028.832	108.5955	0.075395	0.001781	1081.77	44.69534	5.682576	0.109425	2.416312	1.833072	0.057775	0.176175	0.003391	0.509611	0.23604
91500_004	27.004	19	0.183356	0.002816	1085.229	15.34131	1.891166	0.04668	1077.433	16.36636	0.054388	0.005537	1070.038	106.0314	0.074716	0.001512	1055.813	40.80381	5.454632	0.084096	2.439895	1.891166	0.04668	0.183356	0.002816	0.285532	0.142292
91500_005	27.007	19	0.183083	0.003271	1083.68	17.80853	1.96521	0.057912	1102.511	19.90768	0.053906	0.00587	1060.422	112.4645	0.078228	0.002072	1144.087	52.17105	5.470448	0.096911	3.197318	1.96521	0.057912	0.183083	0.003271	0.228484	0.283035
91500_006	27.006	19	0.180506	0.003498	1069.579	19.11122	1.899731	0.052838	1080.065	18.66689	0.05439	0.005472	1070.15	104.866	0.07588	0.001758	1085.221	46.6198	5.538253	0.113155	2.47423	1.899731	0.052838	0.180506	0.003498	0.361175	0.319707
91500_007	27.004	19	0.179346	0.003062	1063.305	16.73785	1.863406	0.049649	1067.424	17.61132	0.051045	0.005168	1005.925	99.29378	0.07551	0.001437	1077.769	37.68011	5.576496	0.095362	2.477287	1.863406	0.049649	0.179346	0.003062	0.364426	0.111216
91500_008	19.52352	19	0.176947	0.003009	1050.208	16.48306	1.85465	0.074627	1067.57	24.97206	0.053553	0.00558	1054.06	107.0634	0.076364	0.002384	1122.095	60.14893	5.648909	0.096397	2.437685	1.85465	0.074627	0.176947	0.003009	0.378862	-0.00028
91500_009	15.05007	19	0.179792	0.004236	1065.676	23.12081	1.828023	0.099222	1059.961	33.29093	0.057739	0.006569	1133.911	125.3064	0.074253	0.003792	1028.743	105.3708	5.547729	0.119337	3.239538	1.828023	0.099222	0.179792	0.004236	0.239064	0.131809
91500_010	19.9399	19	0.176328	0.004133	1046.676	22.65444	1.832227	0.05687	1059.179	19.31201	0.051092	0.005726	1006.5	109.9713	0.075672	0.002144	1078.441	57.77372	5.66698	0.141294	2.5686	1.832227	0.05687	0.176328	0.004133	0.148352	0.536212
91500_011	27.006	19	0.179891	0.002951	1066.302	16.12857	1.876268	0.052147	1071.842	18.32486	0.052228	0.005373	1028.567	103.1263	0.075046	0.001583	1071.072	44.37808	5.554081	0.095459	2.407432	1.876268	0.052147	0.179891	0.002951	0.330205	0.116836

	Duration (s)	Spot Size (µm)	Final Pb206/U238_mean	Final Pb206/U238_2SE(prop)	Final Pb206/U238 age_mean	Final Pb206/U238 age_2SE(prop)	Final Pb207/U235_mean	Final Pb207/U235_2SE(prop)	Final Pb207/U235 age_mean	Final Pb207/U235 age_2SE(prop)	Final Pb208/Th232_mean	Final Pb208/Th232_2SE(prop)	Final Pb208/Th232 age_mean	Final Pb208/Th232 age_2SE(prop)	Final Pb207/Pb206_mean	Final Pb207/Pb206_2SE(prop)	Final Pb207/Pb206 age_mean	Final Pb207/Pb206 age_2SE(prop)	Final U238/Pb206_mean	Final U238/Pb206_2SE(prop)	Final U/Th_mean	Final Pb207/U235_mean	Final Pb207/U235_2SE(prop)	Final Pb206/U238_mean	Final Pb206/U238_2SE(prop)	rho 206Pb/238U v 207Pb/235U	rho 207Pb/206Pb v 238U/206Pb
91500_012	28.27034	19	0.181018	0.003605	**1072.35**	**19.65086**	1.875191	0.053233	1071.327	18.92279	0.052986	0.005335	1043.216	102.4474	0.075542	0.001847	1082.105	50.42871	5.53499	0.108647	2.473635	1.875191	0.053233	0.181018	0.003605	0.393967	0.268492
				sumary stats IsoplotR	**1065.56**	**8.4**	0.79%	**8.4**	0.79%																		
**Z_AUSZ7-1**																											
AUSZ7-1_001	27.69412	19	0.005738	0.000185	**36.88411**	**1.186093**	0.039506	0.004919	39.22883	4.788162	0.00195	0.000402	39.36814	8.111248	0.050455	0.006485	270.2096	285.4365	175.7499	5.749844	2.834131	0.039506	0.004919	0.005738	0.000185	0.049742	0.127585
AUSZ7-1_002	24.19441	19	0.006124	0.000175	**39.35662**	**1.119738**	0.042762	0.005206	42.40617	5.073167	0.001975	0.000375	39.86169	7.556737	0.051343	0.006358	202.3815	276.1748	164.2374	4.776017	2.818934	0.042762	0.005206	0.006124	0.000175	0.01637	0.275985
AUSZ7-1_003	28.63737	19	0.005904	0.000177	**37.94512**	**1.133688**	0.040901	0.005192	40.57047	5.056007	0.001635	0.000313	33.01302	6.305044	0.050748	0.00638	214.1538	249.4565	169.9312	5.28597	2.770763	0.040901	0.005192	0.005904	0.000177	0.22385	-0.11102
AUSZ7-1_004	23.70912	19	0.005925	0.000188	**38.08398**	**1.205319**	0.044735	0.005731	44.30551	5.565752	0.001842	0.000356	37.18428	7.176627	0.053552	0.006589	178.0678	317.3489	165.9355	5.483018	2.841098	0.044735	0.005731	0.005925	0.000188	-0.0587	0.10262
AUSZ7-1_005	27.004	19	0.006146	0.00017	**39.49785**	**1.088204**	0.038681	0.003928	38.46504	3.841016	0.002169	0.000421	43.78416	8.48595	0.044677	0.004296	-42.6681	248.6309	163.6771	4.467747	2.844771	0.038681	0.003928	0.006146	0.00017	0.035734	0.222606
AUSZ7-1_006	27.004	19	0.005879	0.000176	**37.78959**	**1.126291**	0.039063	0.004161	38.82984	4.060271	0.001662	0.000364	33.56233	7.350564	0.047792	0.005105	31.2086	251.4087	169.579	5.046966	2.89511	0.039063	0.004161	0.005879	0.000176	-0.0943	0.321395
AUSZ7-1_007	27.007	19	0.005869	0.000173	**37.7198**	**1.107328**	0.040381	0.004767	40.09256	4.655953	0.001682	0.000386	33.95405	7.795409	0.048314	0.005245	195.3204	245.0513	170.7278	5.31244	2.902901	0.040381	0.004767	0.005869	0.000173	0.294264	-0.09211
AUSZ7-1_008	27.004	19	0.005913	0.000151	**38.00797**	**0.967864**	0.035865	0.004975	35.66388	4.881983	0.00176	0.000331	35.52899	6.667906	0.044902	0.006338	127.5571	287.258	169.7185	4.422853	2.816099	0.035865	0.004975	0.005913	0.000151	-0.23402	0.415648
AUSZ7-1_009	27.006	19	0.005739	0.000127	**36.8906**	**0.816423**	0.038473	0.005312	38.20159	5.186147	0.001774	0.000363	35.81981	7.314915	0.047123	0.006267	55.29247	310.7257	173.6445	4.183673	2.781999	0.038473	0.005312	0.005739	0.000127	0.245652	-0.1366
AUSZ7-1_010	27.01	19	0.005911	0.000133	**37.99043**	**0.852825**	0.03485	0.004928	34.66784	4.828826	0.002142	0.00047	43.22352	9.47836	0.04269	0.006173	-447.331	447.9193	169.7919	3.926999	2.797959	0.03485	0.004928	0.005911	0.000133	0.09373	0.033377
AUSZ7-1_011	27.004	19	0.005745	0.000161	**36.92544**	**1.030999**	0.035722	0.005333	35.50648	5.225874	0.002014	0.000429	40.64186	8.642584	0.045395	0.006088	-112.916	325.8791	174.2305	4.708707	2.786618	0.035722	0.005333	0.005745	0.000161	0.214991	0.009126
AUSZ7-1_012	27.004	19	0.005987	0.000173	**38.48036**	**1.110452**	0.037682	0.004502	37.46532	4.403837	0.001735	0.000371	35.02991	7.481879	0.045585	0.005911	-71.8403	319.6821	168.0936	4.978183	2.840186	0.037682	0.004502	0.005987	0.000173	0.019758	0.181749
				sumary stats IsoplotR	**37.9**	**0.54**	1.42%	**0.5**	1.42%																		
**Z_GJ-1**																											
GJ-1_001	23.45	19	0.09776	0.001704	**601.2388**	**10.00198**	0.829138	0.019897	612.9283	11.04707	0.028701	0.003702	571.4013	72.6413	0.061502	0.001051	653.3164	36.86419	10.23301	0.177465	30.01985	0.829138	0.019897	0.09776	0.001704	0.477825	0.333072
GJ-1_002	27.004	19	0.097233	0.00151	**598.1534**	**8.869911**	0.790636	0.022769	591.0663	12.77239	0.031253	0.003978	621.3337	77.92729	0.059242	0.001229	570.0642	44.82726	10.28371	0.158592	29.01735	0.790636	0.022769	0.097233	0.00151	0.452484	-0.1457
GJ-1_003	27.75068	19	0.097892	0.001933	**601.9854**	**11.33015**	0.791103	0.021504	591.3987	12.10193	0.0309	0.003786	614.511	74.30317	0.05893	0.001395	573.5141	40.77675	10.23004	0.197297	30.29345	0.791103	0.021504	0.097892	0.001933	0.137782	0.079312
GJ-1_004	27.004	19	0.098907	0.001762	**607.9598**	**10.33871**	0.813002	0.020133	603.8683	11.30203	0.028939	0.003552	576.1238	69.8023	0.059759	0.001115	590.0994	40.6035	10.0995	0.173899	29.87706	0.813002	0.020133	0.098907	0.001762	0.352974	0.227455
GJ-1_005	27.004	19	0.098203	0.001813	**603.8228**	**10.63985**	0.817341	0.019862	606.319	11.06791	0.029293	0.003554	583.0938	69.70033	0.060193	0.001027	606.6671	36.70312	10.19508	0.188392	29.80667	0.817341	0.019862	0.098203	0.001813	0.483581	0.424156
GJ-1_006	27.006	19	0.098438	0.001612	**605.225**	**9.463386**	0.816871	0.020475	606.0183	11.4069	0.032263	0.004309	640.9164	84.25688	0.06013	0.001153	610.4023	38.93707	10.16364	0.168837	30.25501	0.816871	0.020475	0.098438	0.001612	0.264896	0.314318
GJ-1_007	27.004	19	0.098802	0.001575	**607.3601**	**9.236406**	0.810391	0.01885	602.4798	10.59128	0.029962	0.003849	596.0506	75.5092	0.060111	0.001126	602.869	39.90251	10.12883	0.16098	29.50841	0.810391	0.01885	0.098802	0.001575	0.387624	0.35797
GJ-1_008	24.73334	19	0.096933	0.001713	**596.3755**	**10.07005**	0.816025	0.025392	605.2434	14.19253	0.031201	0.004322	620.1232	84.7715	0.060764	0.001396	631.5646	52.27626	10.30651	0.19361	30.38068	0.816025	0.025392	0.096933	0.001713	0.491826	0.100629
GJ-1_009	27.008	19	0.097565	0.001538	**600.1048**	**9.032799**	0.806598	0.020096	601.6887	10.652	0.03034	0.003688	603.5916	72.314	0.060014	0.001043	600.0178	37.75549	10.25682	0.162314	28.45351	0.806598	0.020096	0.097565	0.001538	0.498173	0.000149
GJ-1_010	27.004	19	0.097412	0.001627	**599.1936**	**9.559213**	0.80007	0.021816	596.4753	12.29921	0.027202	0.003374	552.6239	72.47435	0.060007	0.001201	598.3721	42.89987	10.25456	0.165202	30.08749	0.80007	0.021816	0.097412	0.001627	0.427915	0.096112
GJ-1_011	27.006	19	0.098254	0.001646	**604.1417**	**9.660314**	0.818292	0.020453	606.8129	11.44997	0.031085	0.003755	618.1984	73.5825	0.060243	0.001089	607.916	38.87654	10.18533	0.17081	29.70612	0.818292	0.020453	0.098254	0.001646	0.387542	0.284859
GJ-1_012	22.49619	19	0.096884	0.001773	**596.0876**	**10.42027**	0.816194	0.021535	605.6294	12.09106	0.03363	0.004386	667.8413	85.80633	0.060434	0.001049	622.1147	40.22199	10.33193	0.190048	29.7862	0.816194	0.021535	0.096884	0.001773	0.629853	0.027452
GJ-1_013	17.17079	19	0.09733	0.00175	**598.7157**	**10.27599**	0.81019	0.018963	602.436	10.6239	0.029259	0.0037	582.5471	72.59142	0.060161	0.00128	605.1128	45.85006	10.27893	0.184149	28.33379	0.81019	0.018963	0.09733	0.00175	0.039019	0.782055
GJ-1_014	24.04484	19	0.098536	0.001771	**605.7872**	**10.40856**	0.813142	0.026074	606.9177	12.65416	0.029211	0.003958	581.2631	77.81501	0.060063	0.001421	614.4418	42.13722	10.15806	0.190645	30.18737	0.813142	0.026074	0.098536	0.001771	0.357933	0.140124
GJ-1_015	27.007	19	0.098501	0.001845	**605.57**	**10.82913**	0.812075	0.020927	603.2888	11.78818	0.031716	0.004002	630.3941	78.51758	0.06011	0.001304	601.0477	45.89611	10.13948	0.179268	30.05888	0.812075	0.020927	0.098501	0.001845	0.228868	0.381489
GJ-1_016	27.004	19	0.098273	0.001669	**604.249**	**9.784727**	0.832754	0.02351	614.6752	12.9684	0.029447	0.004328	585.5459	84.80464	0.060723	0.001341	622.6736	47.65057	10.18381	0.169702	29.65593	0.832754	0.02351	0.098273	0.001669	0.482245	0.008781
GJ-1_017	28.71993	19	0.097069	0.001717	**597.1672**	**10.087**	0.806321	0.019981	600.1016	11.24706	0.031843	0.004114	632.7812	80.53303	0.06018	0.001152	604.7557	41.43578	10.31416	0.18165	29.95246	0.806321	0.019981	0.097069	0.001717	0.378582	0.183986
GJ-1_018	27.006	19	0.098187	0.001656	**603.7462**	**9.72534**	0.808671	0.018231	601.5493	10.22328	0.029639	0.004051	589.5403	79.5075	0.05989	0.000988	596.0089	35.42482	10.17148	0.182703	29.86204	0.808671	0.018231	0.098187	0.001656	0.475075	0.301139
GJ-1_019	27.008	19	0.097975	0.001756	**602.4888**	**10.31869**	0.815309	0.020711	605.1264	11.61591	0.032607	0.003881	648.0189	75.87693	0.060214	0.001063	607.0982	38.1511	10.20157	0.197514	31.89456	0.815309	0.020711	0.097975	0.001756	0.606675	-0.00866
GJ-1_020	27.012	19	0.097732	0.001613	**601.0795**	**9.47335**	0.809069	0.021544	601.5677	12.10082	0.029875	0.003476	594.5958	68.20937	0.059906	0.00124	594.2087	45.11876	10.22712	0.178144	28.80666	0.809069	0.021544	0.097732	0.001613	0.3698	0.278833
GJ-1_021	27.004	19	0.097285	0.001771	**598.4351**	**10.40353**	0.807424	0.019032	600.8008	10.71053	0.027204	0.003385	542.0292	66.57523	0.06032	0.001093	618.3931	35.44453	10.27644	0.178841	29.29325	0.807424	0.019032	0.097285	0.001771	0.397593	0.218014
GJ-1_022	27.90507	19	0.098631	0.00186	**606.3298**	**10.91635**	0.815442	0.022161	605.0978	12.35032	0.031345	0.003605	623.4108	70.67419	0.059667	0.001063	587.1871	38.19648	10.15238	0.194665	29.96694	0.815442	0.022161	0.098631	0.00186	0.907073	0.307917
GJ-1_023	16.61583	19	0.098686	0.001994	**606.6648**	**11.70712**	0.810573	0.025849	602.3526	14.49554	0.028383	0.00413	565.1036	81.19258	0.06041	0.001391	623.6946	52.85183	10.14215	0.208884	29.44213	0.810573	0.025849	0.098686	0.001994	0.383814	0.291191
GJ-1_024	25.74753	19	0.09751	0.001751	**599.765**	**10.28821**	0.813544	0.020856	605.4605	11.14196	0.030557	0.003927	607.7282	77.04803	0.060456	0.000927	622.6249	30.75144	10.23916	0.174224	28.8608	0.813544	0.020856	0.09751	0.001751	0.562325	0.197129
GJ-1_025	27.005	19	0.097948	0.001758	**602.334**	**10.32237**	0.81645	0.022847	605.6226	12.78499	0.031551	0.004725	640.4919	97.85349	0.060495	0.00121	615.9153	42.80302	10.21094	0.184926	30.22364	0.81645	0.022847	0.097948	0.001758	0.39479	0.248068
GJ-1_026	27.004	19	0.09755	0.00169	**600.0021**	**9.930304**	0.803485	0.019889	598.53	11.18262	0.028899	0.00392	575.0785	76.88736	0.059703	0.001112	594.0954	42.1275	10.25035	0.179791	29.79841	0.803485	0.019889	0.09755	0.00169	0.431998	0.287661
GJ-1_027	27.016	19	0.09744	0.001737	**599.3528**	**10.20075**	0.815165	0.020008	605.0911	11.18921	0.030396	0.003493	604.8381	68.5406	0.060398	0.001118	620.0869	37.8083	10.26347	0.182493	29.71437	0.815165	0.020008	0.09744	0.001737	0.481923	0.110795
GJ-1_028	16.17202	19	0.097577	0.002181	**600.1442**	**12.82159**	0.820933	0.028017	611.0886	16.90517	0.026954	0.003705	537.1884	72.86187	0.061582	0.001635	665.5285	60.71799	10.25926	0.234685	29.86326	0.820933	0.028017	0.097577	0.002181	0.537061	-0.02964
GJ-1_029	27.004	19	0.097857	0.00156	**601.8179**	**9.156843**	0.810039	0.019471	602.2466	10.89702	0.032589	0.004116	647.4842	80.54472	0.059802	0.001059	597.7681	36.3204	10.21899	0.16259	29.38227	0.810039	0.019471	0.097857	0.00156	0.465943	0.051165
GJ-1_030	24.76138	19	0.09895	0.001796	**608.2132**	**10.53536**	0.813046	0.021417	603.8363	12.05709	0.029795	0.003662	592.9329	71.88404	0.059785	0.00117	590.7884	43.12526	10.11278	0.183569	29.82698	0.813046	0.021417	0.09895	0.001796	0.250093	0.023865
GJ-1_031	23.05939	19	0.097624	0.002009	**600.413**	**11.80716**	0.807429	0.019408	600.8149	10.93914	0.028943	0.004274	575.8268	83.90063	0.060678	0.001095	624.0147	38.52299	10.21574	0.176655	29.6166	0.807429	0.019408	0.097624	0.002009	0.07894	0.786356
GJ-1_032	27.004	19	0.096902	0.001449	**596.2109**	**8.516056**	0.803312	0.020326	598.4026	11.48759	0.030952	0.003585	615.7134	70.31696	0.05978	0.001103	590.9402	40.57534	10.32614	0.155704	30.1526	0.803312	0.020326	0.096902	0.001449	0.210182	0.284565
GJ-1_033	27.004	19	0.099199	0.001551	**609.6927**	**9.093318**	0.826888	0.020744	611.5971	11.45354	0.030699	0.004061	610.3826	79.57745	0.060296	0.001226	608.6007	43.36823	10.08908	0.157333	29.53855	0.826888	0.020744	0.099199	0.001551	0.065049	0.410166
GJ-1_034	27.004	19	0.098333	0.00176	**604.5929**	**10.33707**	0.819485	0.019806	609.0761	10.16355	0.029899	0.003611	594.9753	70.89816	0.060085	0.001257	607.9656	43.20152	10.15558	0.17142	29.30785	0.819485	0.019806	0.098333	0.00176	0.364932	0.461222
GJ-1_035	27.012	19	0.097667	0.001759	**600.6838**	**10.32517**	0.809121	0.01912	601.7505	10.72864	0.030391	0.003773	604.5417	73.88238	0.060282	0.001127	608.9161	40.73578	10.25294	0.183121	30.02683	0.809121	0.01912	0.097667	0.001759	0.253395	0.484225
GJ-1_036	27.012	19	0.097792	0.001714	**601.4196**	**10.06238**	0.804729	0.019687	599.2429	11.09102	0.02973	0.004065	591.3293	79.77079	0.06002	0.001042	600.252	37.46864	10.23814	0.179017	29.7768	0.804729	0.019687	0.097792	0.001714	0.534533	0.19836
GJ-1_037	27.004	19	0.098276	0.001724	**604.2632**	**10.12153**	0.818178	0.020558	606.7415	11.53555	0.029473	0.003463	586.6901	68.0724	0.06019	0.000968	606.9829	34.82396	10.18753	0.179195	29.39165	0.818178	0.020558	0.098276	0.001724	0.59619	0.122199
GJ-1_038	27.006	19	0.097709	0.001516	**600.9496**	**8.908053**	0.807683	0.020121	600.8804	11.30691	0.030082	0.003887	598.3759	76.2046	0.06029	0.001248	608.0957	44.69067	10.22295	0.169802	30.05062	0.807683	0.020121	0.097709	0.001516	0.268657	0.279493
GJ-1_039	27.004	19	0.097238	0.001783	**598.1608**	**10.47322**	0.806909	0.022882	602.0642	13.5494	0.030588	0.003627	617.372	66.20977	0.060113	0.001231	601.9208	43.84238	10.2991	0.189143	29.54336	0.806909	0.022882	0.097238	0.001783	0.554394	0.028237
GJ-1_040	21.38558	19	0.098363	0.001564	**604.7918**	**9.181112**	0.830017	0.023157	613.2658	12.82926	0.030927	0.003736	615.2246	73.24486	0.061151	0.001129	640.6765	39.70618	10.16511	0.161562	29.57934	0.830017	0.023157	0.098363	0.001564	0.499634	0.007485
GJ-1_041	27.01	19	0.097666	0.001529	**600.6954**	**8.978691**	0.808711	0.020625	601.43	11.50434	0.031102	0.003698	618.5726	72.49098	0.059546	0.001094	591.0415	43.49405	10.23854	0.161312	29.8449	0.808711	0.020625	0.097666	0.001529	0.053174	0.355211
GJ-1_042	16.50416	19	0.097513	0.002304	**599.7601**	**13.53873**	0.800823	0.022326	597.036	12.61112	0.027207	0.003995	542.0295	78.62592	0.059369	0.001618	573.8608	59.40709	10.27072	0.245653	30.0747	0.800823	0.022326	0.097513	0.002304	0.264847	0.545098
	**Duration (s)**	**Spot Size (µm)**	**Final Pb206/U238_mean**	**Final Pb206/U238_2SE(prop)**	**Final Pb206/U238 age_mean**	**Final Pb206/U238 age_2SE(prop)**	**Final Pb207/U235_mean**	**Final Pb207/U235_2SE(prop)**	**Final Pb207/U235 age_mean**	**Final Pb207/U235 age_2SE(prop)**	**Final Pb208/Th232_mean**	**Final Pb208/Th232_2SE(prop)**	**Final Pb208/Th232 age_mean**	**Final Pb208/Th232 age_2SE(prop)**	**Final Pb207/Pb206_mean**	**Final Pb207/Pb206_2SE(prop)**	**Final Pb207/Pb206 age_mean**	**Final Pb207/Pb206 age_2SE(prop)**	**Final U238/Pb206_mean**	**Final U238/Pb206_2SE(prop)**	**Final U/Th_mean**	**Final Pb207/U235_mean**	**Final Pb207/U235_2SE(prop)**	**Final Pb206/U238_mean**	**Final Pb206/U238_2SE(prop)**	**rho 206Pb/238U v 207Pb/235U**	**rho 207Pb/206Pb v 238U/206Pb**

				sumary stats IsoplotR	**602.03**	**1.52**	0.25%	**1.5**	0.25%																		
**Z_Plesovice**																											
Plesovice_001	24.36864	19	0.053106	0.000951	**333.5527**	**5.819643**	0.40301	0.011623	343.6484	8.404143	0.019711	0.002194	394.4115	43.46122	0.055081	0.001135	418.2233	43.74859	18.84495	0.339701	10.26651	0.40301	0.011623	0.053106	0.000951	0.514086	-0.05408
Plesovice_002	27.009	19	0.054337	0.000881	**341.0855**	**5.387981**	0.397403	0.009442	339.6751	6.873946	0.0178	0.001841	356.5471	36.5439	0.052972	0.000994	322.4169	42.84195	18.38727	0.285479	9.164078	0.397403	0.009442	0.054337	0.000881	0.257316	0.324513
Plesovice_004	27.004	19	0.053391	0.000874	**335.2987**	**5.350706**	0.391854	0.010383	335.5813	7.578302	0.015898	0.00164	318.7583	32.62053	0.053125	0.001154	327.0638	49.50107	18.73832	0.305697	9.293497	0.391854	0.010383	0.053391	0.000874	0.123207	0.354913
Plesovice_005	16.36146	19	0.053993	0.000952	**338.9826**	**5.823383**	0.395654	0.01306	338.3098	9.553252	0.016552	0.001971	331.7383	39.1896	0.053173	0.001496	348.342	52.0909	18.53842	0.32977	10.53309	0.395654	0.01306	0.053993	0.000952	0.091144	0.173729
Plesovice_007	23.69284	19	0.053579	0.000979	**336.4438**	**5.989604**	0.398827	0.011373	340.6314	8.214515	0.016833	0.001744	337.3533	34.67081	0.053823	0.001023	358.9276	42.96574	18.66974	0.342582	9.290382	0.398827	0.011373	0.053579	0.000979	0.532853	0.057903
Plesovice_008	27.004	19	0.053029	0.000823	**333.0854**	**5.03983**	0.389168	0.009582	333.6599	7.0011	0.017699	0.001897	354.5215	37.66729	0.053132	0.00094	336.0959	37.73188	18.84694	0.292096	10.12584	0.389168	0.009582	0.053029	0.000823	0.369982	0.165685
Plesovice_009	11.754	19	0.053502	0.001117	**335.9764**	**6.831457**	0.386839	0.015123	331.8604	11.0522	0.016488	0.002048	330.4768	40.73191	0.052979	0.001313	323.2918	56.70744	18.70596	0.385985	9.220792	0.386839	0.015123	0.053502	0.001117	0.8589	-0.64159
Plesovice_010	27.005	19	0.052119	0.000937	**327.5062**	**5.737152**	0.38074	0.009284	327.489	6.816823	0.015815	0.001601	317.1133	31.85275	0.052757	0.000956	313.5032	41.72145	19.21644	0.339932	9.299781	0.38074	0.009284	0.052119	0.000937	0.465601	0.413818
Plesovice_011	21.94307	19	0.052723	0.00094	**331.2106**	**5.758454**	0.388637	0.010428	334.4667	8.204529	0.017054	0.001773	341.7572	35.24018	0.053105	0.001143	335.3505	51.09151	18.98577	0.33918	9.310412	0.388637	0.010428	0.052723	0.00094	0.491304	0.222362
Plesovice_012	27.004	19	0.054115	0.000792	**339.7292**	**4.846367**	0.403077	0.009426	344.65	7.255105	0.017579	0.001765	352.1883	35.04963	0.05376	0.000968	356.3428	40.61307	18.4559	0.284951	9.315974	0.403077	0.009426	0.054115	0.000792	0.309497	0.156055
				sumary stats IsoplotR	**335.45**	**3**	0.89%	**3.0**	0.89%																		
**ZirconBlank**																											
Zircon Blank_001	24.46168	19	0.01083	0.014432	**63.44941**	**91.11823**	0.345076	1.776239	1294.807	355.162	-15.1697	11.84252	53288.3	7919.417	0.634843	0.763854	2882.165	870.5616	4.656409	16.82761	-59.1192	0.345076	1.776239	0.01083	0.014432	0.06862	-0.04944
Zircon Blank_002	27.02	19	-0.00279	0.009708	**-21.0575**	**62.75453**	0.003882	1.315964	1430.24	238.1607	-1.89484	1.352428	14513.72	7069.391	0.218267	0.697364	3632.986	881.0811	8.449604	27.46991	0.414315	0.003882	1.315964	-0.00279	0.009708	-0.13206	-0.23911
Zircon Blank_003	27.004	19	-0.00568	0.011425	**-41.0323**	**74.77783**	-0.7213	1.159725	874.2733	416.6463	-4.116	14.45329	60713.36	7960.023	-0.23738	0.62873	3877.754	702.3465	8.985263	24.24976	78.3496	-0.7213	1.159725	-0.00568	0.011425	-0.09585	-0.21598
Zircon Blank_004	27.005	19	-0.00125	0.01649	**-16.9228**	**107.0737**	-1.9051	1.699797	739.0291	429.7834	-1.0925	4.476518	34549.28	7583.908	-0.11029	0.425691	3393.851	724.1248	-8.39385	18.56432	41.16125	-1.9051	1.699797	-0.00125	0.01649	0.289472	-0.04581
Zircon Blank_005	27.004	19	0.003435	0.010865	**18.28708**	**70.46341**	2.147015	1.209402	1459.431	239.0333	-1.443	3.066877	36796.64	7575.402	0.326834	0.662581	3472.092	596.5397	16.72151	19.43096	-31.4993	2.147015	1.209402	0.003435	0.010865	0.301538	0.049009
Zircon Blank_006	27.004	19	0.00896	0.009128	**54.85825**	**58.53366**	-0.53843	0.965966	730.9935	287.8344	-0.48743	2.172922	17041.28	12766.47	0.500422	1.297187	3972.545	520.2053	10.52355	15.18723	-10.9973	-0.53843	0.965966	0.00896	0.009128	0.144159	-0.29109
Zircon Blank_007	27.004	19	5.11E-05	0.007803	**-1.63545**	**50.39555**	-0.2019	1.058358	269.172	706.8159	-1.83441	1.649444	-953.008	12153.06	0.703588	0.600638	4061.965	467.69	-18.2939	27.25161	9.582811	-0.2019	1.058358	5.11E-05	0.007803	0.324603	-0.04225
Zircon Blank_008	27.004	19	-0.00563	0.015141	**-44.0055**	**99.67979**	1.000791	1.528889	1149.651	304.4667	-0.21364	0.672772	8475.095	5756.097	0.224122	0.588476	3368.472	963.4813	13.45585	16.04161	-7.94359	1.000791	1.528889	-0.00563	0.015141	0.035038	0.530168
Zircon Blank_009	27.006	19	0.005334	0.005999	**40.33756**	**40.76917**	-0.58218	1.02183	1263.2	273.6659	0.334835	2.219746	30900.03	3179.145	13.68611	37.64811	4356.831	169.3885	-1625.04	3713.007	49.19647	-0.58218	1.02183	0.005334	0.005999	0.124839	0.229216

**Systematic uncertainty :**	**1.50%**				
all	**48.66**	**0.1**	0.21%	**0.10**	0.21%

Supplementary Table 2. Complete 40Ar/39Ar data

Sample: K8-9 **Identifier:** UW150:C80
Material: Groundmass

N	Power %	Age (Ma)	±1σ (Ma)	K/Ca	±1σ	Cum. %^{39}Ar (%)	%^{40}Ar (%)	^{40}Ar*/^{39}Ar$_K$	±1σ	^{39}Ar/^{40}Ar	±1σ	^{36}Ar/^{40}Ar	±1σ	Corrected[1] ^{40}Ar (cps)	±1σ	^{39}Ar (cps)	±1σ	^{38}Ar (cps)	±1σ	^{37}Ar (cps)	±1σ	^{36}Ar (cps)	±1σ	J	±1σ	LambdaK	MonitorAge	Monitor	Material	(^{40}Ar/^{39}39)$_K$	±1σ	(^{38}Ar/^{39}39)$_K$	±1σ	(^{37}Ar/^{39}39)$_K$	±1σ	(^{39}Ar/^{37}37)$_{Ca}$	±1σ	(^{38}Ar/^{37}37)$_{Ca}$	±1σ	(^{36}Ar/^{37}37)$_{Ca}$	±1σ	(^{36}Ar/^{38}38)$_{Cl}$	Ca/K
01A	0.35	62.220	11.641	0.0511	0.0016	0.254	6.632	1.5888	0.3023	0.04175	0.00014	0.003127	0.000042	43032.01	40.34	1804.00	5.90	70.56	2.79	189.90	6.05	138.65	1.80	0.021765	0.000008	5.464E-10	28.201	FC-2	sanidine	0.00054	0.00014	0.01210	0.00002	0.000224	0.000016	0.000702	0.000012	0.000020	0.000001	0.0002702	0.0000004	263.00	2.32
01B	0.37	49.706	8.674	0.0485	0.0009	0.681	6.935	1.2649	0.2237	0.05484	0.00014	0.003117	0.000041	54975.49	47.69	3027.93	7.24	82.07	1.99	335.55	6.41	178.56	2.24	0.021765	0.000008	5.464E-10	28.201	FC-2	sanidine	0.00054	0.00014	0.01210	0.00002	0.000224	0.000016	0.000702	0.000012	0.000020	0.000001	0.0002702	0.0000004	263.00	2.32
01C	0.39	54.189	8.995	0.0389	0.0007	1.040	8.380	1.3807	0.2326	0.06071	0.00017	0.003069	0.000047	41805.76	40.21	2553.09	6.53	72.75	2.97	352.08	6.46	135.85	1.96	0.021765	0.000008	5.464E-10	28.201	FC-2	sanidine	0.00054	0.00014	0.01210	0.00002	0.000224	0.000016	0.000702	0.000012	0.000020	0.000001	0.0002702	0.0000004	263.00	2.32
01D	0.43	43.728	5.201	0.0413	0.0005	1.842	8.824	1.1110	0.1337	0.07946	0.00016	0.003054	0.000035	71350.78	58.53	5699.64	10.61	121.15	2.50	739.44	8.80	233.78	2.51	0.021765	0.000008	5.464E-10	28.201	FC-2	sanidine	0.00054	0.00014	0.01210	0.00002	0.000224	0.000016	0.000702	0.000012	0.000020	0.000001	0.0002702	0.0000004	263.00	2.32
01E	0.53	54.763	2.769	0.0339	0.0003	4.234	14.540	1.3956	0.0716	0.10424	0.00012	0.002862	0.000025	162190.73	104.50	17021.89	16.24	317.92	3.27	2681.33	23.37	521.93	3.98	0.021765	0.000008	5.464E-10	28.201	FC-2	sanidine	0.00054	0.00014	0.01210	0.00002	0.000224	0.000016	0.000702	0.000012	0.000020	0.000001	0.0002702	0.0000004	263.00	2.32
01F	0.65	52.871	1.628	0.0260	0.0002	7.345	20.039	1.3467	0.0421	0.14893	0.00013	0.002678	0.000021	147736.55	82.96	22204.85	13.64	326.40	5.96	4545.71	37.57	493.62	2.94	0.021765	0.000008	5.464E-10	28.201	FC-2	sanidine	0.00054	0.00014	0.01210	0.00002	0.000224	0.000016	0.000702	0.000012	0.000020	0.000001	0.0002702	0.0000004	263.00	2.32
01G	0.8	54.278	1.194	0.0259	0.0002	12.407	25.368	1.3830	0.0309	0.18361	0.00014	0.002499	0.000019	194969.11	105.66	36123.63	16.91	534.40	6.87	7435.99	60.73	647.68	3.38	0.021765	0.000008	5.464E-10	28.201	FC-2	sanidine	0.00054	0.00014	0.01210	0.00002	0.000224	0.000016	0.000702	0.000012	0.000020	0.000001	0.0002702	0.0000004	263.00	2.32
01H	1	51.740	1.145	0.0229	0.0002	19.597	24.997	1.3174	0.0296	0.18993	0.00015	0.002511	0.000019	267736.35	156.38	51390.39	24.15	760.70	8.94	11930.01	96.91	930.10	4.46	0.021765	0.000008	5.464E-10	28.201	FC-2	sanidine	0.00054	0.00014	0.01210	0.00002	0.000224	0.000016	0.000702	0.000012	0.000020	0.000001	0.0002702	0.0000004	263.00	2.32
01I	1.2	50.441	1.026	0.0210	0.0002	26.426	31.278	1.2839	0.0265	0.24393	0.00022	0.002300	0.000021	198038.25	129.04	48863.17	27.01	674.55	10.67	12352.59	100.36	722.66	3.59	0.021765	0.000008	5.464E-10	28.201	FC-2	sanidine	0.00054	0.00014	0.01210	0.00002	0.000224	0.000016	0.000702	0.000012	0.000020	0.000001	0.0002702	0.0000004	263.00	2.32
01J	1.45	51.234	1.121	0.0182	0.0002	33.423	31.333	1.3044	0.0290	0.24052	0.00024	0.002299	0.000023	205800.08	149.14	50178.34	28.82	715.68	8.31	14581.71	118.39	788.65	3.94	0.021765	0.000008	5.464E-10	28.201	FC-2	sanidine	0.00054	0.00014	0.01210	0.00002	0.000224	0.000016	0.000702	0.000012	0.000020	0.000001	0.0002702	0.0000004	263.00	2.32
01K	1.75	50.322	1.158	0.0156	0.0001	41.278	33.170	1.2808	0.0299	0.25933	0.00020	0.002237	0.000026	214300.38	120.58	56481.88	20.80	831.75	9.87	19034.70	154.30	891.85	4.31	0.021765	0.000008	5.464E-10	28.201	FC-2	sanidine	0.00054	0.00014	0.01210	0.00002	0.000224	0.000016	0.000702	0.000012	0.000020	0.000001	0.0002702	0.0000004	263.00	2.32
01L	2.1	50.666	1.301	0.0123	0.0001	48.960	38.595	1.2897	0.0336	0.29973	0.00025	0.002055	0.000033	181358.89	103.48	55523.30	21.45	827.12	11.37	23745.62	192.39	887.65	4.30	0.021765	0.000008	5.464E-10	28.201	FC-2	sanidine	0.00054	0.00014	0.01210	0.00002	0.000224	0.000016	0.000702	0.000012	0.000020	0.000001	0.0002702	0.0000004	263.00	2.32
01M	2.5	50.780	1.344	0.0120	0.0001	54.647	39.958	1.2927	0.0347	0.30963	0.00028	0.002009	0.000036	129999.74	78.36	41129.77	18.61	590.10	6.52	17888.77	145.04	649.39	3.34	0.021765	0.000008	5.464E-10	28.201	FC-2	sanidine	0.00054	0.00014	0.01210	0.00002	0.000224	0.000016	0.000702	0.000012	0.000020	0.000001	0.0002702	0.0000004	263.00	2.32
01N	3	53.911	1.520	0.0098	0.0001	62.934	40.643	1.3735	0.0393	0.29637	0.00027	0.001986	0.000039	197851.22	114.21	60254.49	22.79	897.31	9.93	31965.89	258.05	1087.45	5.07	0.021765	0.000008	5.464E-10	28.201	FC-2	sanidine	0.00054	0.00014	0.01210	0.00002	0.000224	0.000016	0.000702	0.000012	0.000020	0.000001	0.0002702	0.0000004	263.00	2.32
01O	3.6	52.682	1.706	0.0091	0.0001	68.868	36.845	1.3418	0.0441	0.27501	0.00027	0.002113	0.000040	152690.91	91.27	43256.63	18.84	641.06	10.61	24694.63	199.60	859.45	4.26	0.021765	0.000008	5.464E-10	28.201	FC-2	sanidine	0.00054	0.00014	0.01210	0.00002	0.000224	0.000016	0.000702	0.000012	0.000020	0.000001	0.0002702	0.0000004	263.00	2.32
01P	4.3	54.060	1.796	0.0078	0.0001	83.238	37.519	1.3774	0.0464	0.27279	0.00026	0.002091	0.000042	372722.08	206.79	105296.45	31.00	1601.74	14.08	69606.07	561.10	2292.95	9.50	0.021765	0.000008	5.464E-10	28.201	FC-2	sanidine	0.00054	0.00014	0.01210	0.00002	0.000224	0.000016	0.000702	0.000012	0.000020	0.000001	0.0002702	0.0000004	263.00	2.32
01Q	5.1	50.021	2.361	0.0075	0.0001	84.988	37.606	1.2731	0.0609	0.29587	0.00041	0.002088	0.000060	41839.37	32.81	12838.18	10.41	169.48	4.63	8819.42	70.65	279.27	1.96	0.021765	0.000008	5.464E-10	28.201	FC-2	sanidine	0.00054	0.00014	0.01210	0.00002	0.000224	0.000016	0.000702	0.000012	0.000020	0.000001	0.0002702	0.0000004	263.00	2.32
01R	6	53.701	2.018	0.0072	0.0001	90.086	36.547	1.3681	0.0522	0.26752	0.00029	0.002124	0.000046	134835.50	78.98	37471.16	17.19	558.50	6.95	26692.09	212.27	867.42	4.10	0.021765	0.000008	5.464E-10	28.201	FC-2	sanidine	0.00054	0.00014	0.01210	0.00002	0.000224	0.000016	0.000702	0.000012	0.000020	0.000001	0.0002702	0.0000004	263.00	2.32
01S	7	54.050	2.223	0.0075	0.0001	92.639	38.323	1.3771	0.0575	0.27870	0.00035	0.002064	0.000053	64815.31	43.98	18738.31	13.32	269.39	6.04	12909.87	103.07	414.91	2.58	0.021765	0.000008	5.464E-10	28.201	FC-2	sanidine	0.00054	0.00014	0.01210	0.00002	0.000224	0.000016	0.000702	0.000012	0.000020	0.000001	0.0002702	0.0000004	263.00	2.32
01T	8.2	49.986	2.330	0.0073	0.0001	95.002	32.124	1.2722	0.0601	0.25286	0.00032	0.002272	0.000051	66121.39	46.14	17360.25	12.01	251.21	5.49	12199.75	97.46	416.09	2.54	0.021765	0.000008	5.464E-10	28.201	FC-2	sanidine	0.00054	0.00014	0.01210	0.00002	0.000224	0.000016	0.000702	0.000012	0.000020	0.000001	0.0002702	0.0000004	263.00	2.32
01U	9.5	50.699	2.616	0.0072	0.0001	98.256	29.554	1.2906	0.0675	0.22929	0.00031	0.002358	0.000052	100388.68	71.32	23923.51	18.74	387.76	3.64	17148.96	137.36	610.60	4.17	0.021765	0.000008	5.464E-10	28.201	FC-2	sanidine	0.00054	0.00014	0.01210	0.00002	0.000224	0.000016	0.000702	0.000012	0.000020	0.000001	0.0002702	0.0000004	263.00	2.32
01V	11	51.778	3.319	0.0074	0.0001	100.000	29.092	1.3184	0.0857	0.22093	0.00037	0.002374	0.000063	55846.47	48.62	12804.04	15.04	215.04	3.23	8833.01	70.67	325.27	3.15	0.021765	0.000008	5.464E-10	28.201	FC-2	sanidine	0.00054	0.00014	0.01210	0.00002	0.000224	0.000016	0.000702	0.000012	0.000020	0.000001	0.0002702	0.0000004	263.00	2.32

Integrated K/Ca ±2σ **0.0115** **0.0001**
Plateau Age ±2σ **51.827** **0.720** **n=22/22** **MSWD=1.011**
Total Integrated Age ±2σ **52.190** **0.847**
Isochron Age ±2σ **51.139** **1.568** **MSWD=1.030**
(^{40}Ar/^{36}Ar)$_{trapped}$ ±2σ **300.279** **3.506**

Sample: K8-15 **Identifier:** UW150:C82
Material: Groundmass

N	Power %	Age (Ma)	±1σ (Ma)	K/Ca	±1σ	Cum. %^{39}Ar (%)	%^{40}Ar (%)	^{40}Ar*/^{39}Ar$_K$	±1σ	Corrected[1] ^{39}Ar/^{40}Ar	±1σ	^{36}Ar/^{40}Ar	±1σ	^{40}Ar (cps)	±1σ	^{39}Ar (cps)	±1σ	^{38}Ar (cps)	±1σ	^{37}Ar (cps)	±1σ	^{36}Ar (cps)	±1σ	J	±1σ	LambdaK	MonitorAge	onitorNa	nitorMate	(^{40}Ar/^{39}39)$_K$	±1σ	(^{38}Ar/^{39}39)$_K$	±1σ	(^{37}Ar/^{39}39)$_K$	±1σ	(^{39}Ar/^{37}37)$_{Ca}$	±1σ	(^{38}Ar/^{37}37)$_{Ca}$	±1σ	(^{36}Ar/^{37}37)$_{Ca}$	±1σ	(^{36}Ar/^{38}38)$_{Cl}$	Ca/K
X 01A	0.35	10.647	16.120	0.0324	0.0006	0.291	0.568	0.2681	0.4070	0.02117	0.00006	0.003330	0.000029	114033.82	83.78	2432.88	6.52	127.69	3.32	388.35	6.88	388.41	3.25	0.021765	0.000008	5.464E-10	28.201	FC-2	sanidine	0.00054	0.00014	0.01210	0.00002	0.000224	0.000016	0.000702	0.000012	0.000020	0.000001	0.0002702	0.0000004	263.00	2.32
X 01B	0.37	8.121	10.917	0.0323	0.0005	0.664	0.641	0.2043	0.2753	0.03139	0.00008	0.003328	0.000029	98923.20	71.32	3129.17	7.81	113.45	2.92	501.16	7.46	340.35	2.83	0.021765	0.000008	5.464E-10	28.201	FC-2	sanidine	0.00054	0.00014	0.01210	0.00002	0.000224	0.000016	0.000702	0.000012	0.000020	0.000001	0.0002702	0.0000004	263.00	2.32
X 01C	0.41	50.712	7.924	0.0280	0.0003	1.150	5.323	1.2909	0.2045	0.04124	0.00010	0.003171	0.000028	97829.75	73.99	4070.90	8.88	118.20	2.41	750.03	8.93	326.92	2.73	0.021765	0.000008	5.464E-10	28.201	FC-2	sanidine	0.00054	0.00014	0.01210	0.00002	0.000224	0.000016	0.000702	0.000012	0.000020	0.000001	0.0002702	0.0000004	263.00	2.32
X 01D	0.49	41.013	5.206	0.0296	0.0003	2.342	6.001	1.0412	0.1337	0.05765	0.00009	0.003148	0.000026	171776.85	119.29	9986.03	12.97	236.36	3.38	1742.58	15.62	579.61	4.36	0.021765	0.000008	5.464E-10	28.201	FC-2	sanidine	0.00054	0.00014	0.01210	0.00002	0.000224	0.000016	0.000702	0.000012	0.000020	0.000001	0.0002702	0.0000004	263.00	2.32
X 01E	0.59	47.350	2.377	0.0283	0.0002	4.812	10.470	1.2042	0.0612	0.08699	0.00008	0.002999	0.000018	236028.47	132.83	20709.27	13.67	348.61	6.57	3775.46	29.70	791.88	4.06	0.021765	0.000008	5.464E-10	28.201	FC-2	sanidine	0.00054	0.00014	0.01210	0.00002	0.000224	0.000016	0.000702	0.000012	0.000020	0.000001	0.0002702	0.0000004	263.00	2.32
01F	0.71	55.091	1.882	0.0257	0.0002	9.269	18.431	1.4040	0.0487	0.13137	0.00012	0.002732	0.000021	282029.04	219.65	37398.96	17.73	569.68	7.47	7484.10	57.70	937.38	5.79	0.021765	0.000008	5.464E-10	28.201	FC-2	sanidine	0.00054	0.00014	0.01210	0.00002	0.000224	0.000016	0.000702	0.000012	0.000020	0.000001	0.0002702	0.0000004	263.00	2.32
01G	0.83	54.100	1.783	0.0227	0.0003	14.319	20.313	1.3784	0.0461	0.14748	0.00014	0.002668	0.000023	284711.88	220.23	42448.22	19.29	634.95	10.24	9594.96	104.62	974.10	5.92	0.021765	0.000008	5.464E-10	28.201	FC-2	sanidine	0.00054	0.00014	0.01210	0.00002	0.000224	0.000016	0.000702	0.000012	0.000020	0.000001	0.0002702	0.0000004	263.00	2.32
01H	0.96	52.584	1.873	0.0212	0.0002	19.920	18.541	1.3392	0.0484	0.13855	0.00013	0.002728	0.000022	336091.88	259.20	47118.80	20.55	708.17	8.59	11405.07	124.28	1171.78	6.86	0.021765	0.000008	5.464E-10	28.201	FC-2	sanidine	0.00054	0.00014	0.01210	0.00002	0.000224	0.000016	0.000702	0.000012	0.000020	0.000001	0.0002702	0.0000004	263.00	2.32
01I	1.08	53.748	1.333	0.0198	0.0002	25.372	21.663	1.3693	0.0345	0.15834	0.00012	0.002623	0.000018	286256.51	156.77	45904.02	20.26	682.72	9.15	11847.28	92.81	1016.05	4.67	0.021765	0.000008	5.464E-10	28.201	FC-2	sanidine	0.00054	0.00014	0.01210	0.00002	0.000224	0.000016	0.000702	0.000012	0.000020	0.000001	0.0002702	0.0000004	263.00	2.32
01J	1.2	55.222	1.291	0.0195	0.0002	29.960	26.194	1.4074	0.0334	0.18630	0.00015	0.002471	0.000021	204800.32	112.40	38644.77	17.86	547.10	8.80	10131.68	79.47	732.98	3.79	0.021765	0.000008	5.464E-10	28.201	FC-2	sanidine	0.00054	0.00014	0.01210	0.00002	0.000224	0.000016	0.000702	0.000012	0.000020	0.000001	0.0002702	0.0000004	263.00	2.32
01K	1.34	55.395	1.505	0.0190	0.0002	34.329	22.008	1.4119	0.0390	0.15600	0.00012	0.002612	0.000020	232887.35	125.73	36819.16	17.68	542.43	8.65	9889.23	76.54	829.84	4.33	0.021765	0.000008	5.464E-10	28.201	FC-2	sanidine	0.00054	0.00014	0.01210	0.00002	0.000224	0.000016	0.000702	0.000012	0.000020	0.000001	0.0002702	0.0000004	263.00	2.32
01L	1.5	57.344	1.300	0.0176	0.0001	38.918	26.337	1.4624	0.0337	0.18027	0.00014	0.002466	0.000020	211654.99	116.95	38713.13	17.87	562.71	7.99	11243.37	86.85	774.20	3.74	0.021765	0.000008	5.464E-10	28.201	FC-2	sanidine	0.00054	0.00014	0.01210	0.00002	0.000224	0.000016	0.000702	0.000012	0.000020	0.000001	0.0002702	0.0000004	263.00	2.32
01M	1.67	57.409	1.470	0.0147	0.0001	43.567	25.959	1.4641	0.0381	0.17748	0.00015	0.002479	0.000022	217813.66	122.74	39353.44	18.80	582.01	7.76	13627.06	105.19	845.98	4.22	0.021765	0.000008	5.464E-10	28.201	FC-2	sanidine	0.00054	0.00014	0.01210	0.00002	0.000224	0.000016	0.000702	0.000012	0.000020	0.000001	0.0002702	0.0000004	263.00	2.32
01N	1.87	58.162	1.494	0.0125	0.0001	48.732	28.746	1.4836	0.0387	0.19397	0.00016	0.002386	0.000025	221470.84	122.84	43880.80	18.49	650.90	8.27	17747.50	136.62	927.15	4.52	0.021765	0.000008	5.464E-10	28.201	FC-2	sanidine	0.00054	0.00014	0.01210	0.00002	0.000224	0.000016	0.000702	0.000012	0.000020	0.000001	0.0002702	0.0000004	263.00	2.32
01O	2.1	54.167	1.530	0.0129	0.0001	52.805	26.956	1.3802	0.0396	0.19552	0.00017	0.002446	0.000026	173215.50	99.64	34572.04	16.92	483.69	7.71	13606.15	103.33	729.58	3.74	0.021765	0.000008	5.464E-10	28.201	FC-2	sanidine	0.00054	0.00014	0.01210	0.00002	0.000224	0.000016	0.000702	0.000012	0.000020	0.000001	0.0002702	0.0000004	263.00	2.32
01P	2.35	53.797	1.538	0.0135	0.0001	57.154	24.951	1.3706	0.0398	0.18223	0.00016	0.002513	0.000024	198471.99	112.18	36884.49	18.12	527.99	9.27	13870.92	105.38	810.75	4.09	0.021765	0.000008	5.464E-10	28.201	FC-2	sanidine	0.00054	0.00014	0.01210	0.00002	0.000224	0.000016	0.000702	0.000012	0.000020	0.000001	0.0002702	0.0000004	263.00	2.32
01Q	2.6	57.565	1.603	0.0114	0.0001	61.795	27.864	1.4681	0.0415	0.18999	0.00017	0.002415	0.000026	203131.93	115.59	39513.47	19.02	565.48	8.58	17459.21	132.37	883.61	4.34	0.021765	0.000008	5.464E-10	28.201	FC-2	sanidine	0.00054	0.00014	0.01210	0.00002	0.000224	0.000016	0.000702	0.000012	0.000020	0.000001	0.0002702	0.0000004	263.00	2.32
01R	2.9	55.195	1.636	0.0123	0.0001	65.482	25.533	1.4067	0.0423	0.18168	0.00017	0.002493	0.000025	168753.22	98.53	31331.60	16.31	436.12	8.02	12841.34	97.54	710.01	3.66	0.021765	0.000008	5.464E-10	28.201	FC-2	sanidine	0.00054	0.00014	0.01210	0.00002	0.000224	0.000016	0.000702	0.000012	0.000020	0.000001	0.0002702	0.0000004	263.00	2.32
01S	3.3	54.339	1.561	0.0135	0.0001	69.143	26.453	1.3846	0.0404	0.19125	0.00017	0.002462	0.000026	159200.33	92.40	31049.61	16.50	444.05	8.95	11658.32	88.71	654.72	3.52	0.021765	0.000008	5.464E-10	28.201	FC-2	sanidine	0.00054	0.00014	0.01210	0.00002	0.000224	0.000016	0.000702	0.000012	0.000020	0.000001	0.0002702	0.0000004	263.00	2.32
01T	3.9	55.402	1.766	0.0121	0.0001	78.003	30.603	1.4121	0.0457	0.21698	0.00020	0.002323	0.000033	339627.64	198.71	75325.96	36.50	1122.81	4.83	31373.53	238.05	1496.37	9.75	0.021765	0.000008	5.464E-10	28.201	FC-2	sanidine	0.00054	0.00014	0.01210	0.00002	0.000224	0.000016	0.000702	0.000012	0.000020	0.000001	0.0002702	0.0000004	263.00	2.32
01U	4.4	54.426	1.818	0.0107	0.0001	81.958	33.218	1.3868	0.0470	0.23983	0.00030	0.002235	0.000037	137156.34	113.05	33724.39	25.74	510.91	3.66	15780.99	123.26	662.74	4.28	0.021765	0.000008	5.464E-10	28.201	FC-2	sanidine	0.00054	0.00014	0.01210	0.00002	0.000224	0.000016	0.000702	0.000012	0.000020	0.000001	0.0002702	0.0000004	263.00	2.32
01V	5	52.048	1.848	0.0105	0.0001	86.125	33.402	1.3254	0.0477	0.25236	0.00031	0.002229	0.000040	137371.93	118.50	35563.58	22.65	537.15	3.95	17042.18	133.03	690.97	4.56	0.021765	0.000008	5.464E-10	28.201	FC-2	sanidine	0.00054	0.00014	0.01210	0.00002	0.000224	0.000016	0.000702	0.000012	0.000020	0.000001	0.0002702	0.0000004	263.00	2.32
01W	5.7	53.559	1.853	0.0110	0.0001	90.043	30.617	1.3644	0.0479	0.22466	0.00028	0.002323	0.000036	145045.32	123.03	33392.20	23.69	521.44	3.69	15301.05	119.83	682.43	4.38	0.021765	0.000008	5.464E-10	28.201	FC-2	sanidine	0.00054	0.00014	0.01210	0.00002	0.000224	0.000016	0.000702	0.000012	0.000020	0.000001	0.0002702	0.0000004	263.00	2.32
01X	6.5	54.403	1.717	0.0107	0.0001	96.911	29.578	1.3863	0.0444	0.21361	0.00024	0.002358	0.000031	267404.37	215.24	58573.37	34.11	901.59	4.55	27427.15	219.75	1250.21	6.70	0.021765	0.000008	5.464E-10	28.201	FC-2	sanidine	0.00054	0.00014	0.01210	0.00002	0.000224	0.000016	0.000702	0.000012	0.000020	0.000001	0.0002702	0.0000004	263.00	2.32
01Y	7.5	55.919	2.327	0.0103	0.0001	99.152	28.658	1.4255	0.0602	0.20126	0.00033	0.002389	0.000040	92618.30	104.08	19138.16	19.65	308.28	2.70	9332.48	75.74	432.18	3.29	0.021765	0.000008	5.464E-10	28.201	FC-2	sanidine	0.00054	0.00014	0.01210	0.00002	0.000224	0.000016	0.000702	0.000012	0.000020	0.000001	0.0002702	0.0000004	263.00	2.32
01Z	9	53.243	3.583	0.0095	0.0001	100.000	25.043	1.3563	0.0926	0.18483	0.00051	0.002510	0.000057	38153.56	79.43	7256.92	11.95	125.22	2.79	3799.65	32.04	181.68	2.03	0.021765	0.000008	5.464E-10	28.201	FC-2	sanidine	0.00054	0.00014	0.01210	0.00002	0.000224	0.000016	0.000702	0.000012	0.000020	0.000001	0.0002702	0.0000004	263.00	2.32

Integrated K/Ca ±2σ **0.0140** **0.0001**
Plateau Age ±2σ **55.129** **0.727** **n=21/26** **MSWD=1.034**
Total Integrated Age ±2σ **54.304** **0.762**
Isochron Age ±2σ **55.325** **3.424** **MSWD=1.106**
(^{40}Ar/^{36}Ar)$_{trapped}$ ±2σ **298.191** **6.439**

Sample: K8-18 **Identifier:** UW144:C40
Material: Groundmass

N	Power %	Age (Ma)	±1σ (Ma)	K/Ca	±1σ	Cum. %^{39}Ar (%)	%^{40}Ar (%)	^{40}Ar*/^{39}Ar$_K$	±1σ	Corrected[1] ^{39}Ar/^{40}Ar	±1σ	^{36}Ar/^{40}Ar	±1σ	^{40}Ar (cps)	±1σ	^{39}Ar (cps)	±1σ	^{38}Ar (cps)	±1σ	^{37}Ar (cps)	±1σ	^{36}Ar (cps)	±1σ	J	±1σ	LambdaK	MonitorAge	onitorNa	nitorMate	(^{40}Ar/^{39}39)$_K$	±1σ	(^{38}Ar/^{39}39)$_K$	±1σ	(^{37}Ar/^{39}39)$_K$	±1σ	(^{39}Ar/^{37}37)$_{Ca}$	±1σ	(^{38}Ar/^{37}37)$_{Ca}$	±1σ	(^{36}Ar/^{37}37)$_{Ca}$	±1σ	(^{36}Ar/^{38}38)$_{Cl}$	Ca/K
X 01A	0.34	22.617	11.639	0.1396	0.0235	1.393	4.350	0.7313	0.3787	0.05951	0.00051	0.003204	0.000075	215487.05	1795.22	12829.54	25.21	481.83	3.68	1026.63	172.48	700.88	14.98	0.017003	0.000015	5.464E-10	28.201	FC-2	sanidine	0.00054	0.00014	0.01210	0.00005	0.000224	0.000016	0.000702	0.000012	0.000020	0.000001	0.0002702	0.0000004	263.00	2.32
X 01B	0.4	54.625	3.845	0.0646	0.0005	2.647	15.991	1.7818	0.1273	0.08979	0.00021	0.002814	0.000038	128587.42	132.54	11578.81	23.68	207.00	2.89	1996.29	14.57	382.43	4.88	0.017003	0.000015	5.464E-10	28.201	FC-2	sanidine	0.00054	0.00014	0.01210	0.00005	0.000224	0.000016	0.000702	0.000012	0.000020	0.000001	0.0002702	0.0000004	263.00	2.32
X 01C	0.44	59.296	2.870	0.0611	0.0004	4.918	16.885	1.9367	0.0953	0.08723	0.00015	0.002784	0.000028	239668.17	216.66	20971.48	31.70	392.79	3.51	3816.32	24.27	706.66	6.58	0.017003	0.000015	5.464E-10	28.201	FC-2	sanidine	0.00054	0.00014	0.01210	0.00005	0.000224	0.000016	0.000702	0.000012	0.000020	0.000001	0.0002702	0.0000004	263.00	2.32
X 01D	0.49	58.178	1.876	0.0558	0.0004	7.902	23.367	1.8996	0.0622	0.12309	0.00019	0.002566	0.000025	223164.52	211.21	27564.82	34.44	437.69	3.95	5490.51	34.84	629.65	5.63	0.017003	0.000015	5.464E-10	28.201	FC-2	sanidine	0.00054	0.00014	0.01210	0.00005	0.000224	0.000016	0.000702	0.000012	0.000020	0.000001	0.0002702	0.0000004	263.00	2.32
X 01E	0.54	58.420	1.572	0.0493	0.0003	11.749	28.349	1.9076	0.0522	0.14873	0.00021	0.002399	0.000026	238154.57	210.63	35563.26	38.53	535.79	3.67	8006.66	47.52	654.56	6.09	0.017003	0.000015	5.464E-10	28.201	FC-2	sanidine	0.00054	0.00014	0.01210	0.00005	0.000224	0.000016	0.000702	0.000012	0.000020	0.000001	0.0002702	0.0000004	263.00	2.32
X 01F	0.59	55.716	1.182	0.0430	0.0003	15.570	34.606	1.8180	0.0392	0.19055	0.00028	0.002189	0.000025	184727.18	176.51	35364.80	38.22	496.45	3.97	9106.52	55.18	499.18	4.51	0.017003	0.000015	5.464E-10	28.201	FC-2	sanidine	0.00054	0.00014	0.01210	0.00005	0.000224	0.000016	0.000702	0.000012	0.000020	0.000001	0.0002702	0.0000004	263.00	2.32
01G	0.65	53.611	1.132	0.0364	0.0002	20.457	32.807	1.7482	0.0375	0.18785	0.00034	0.002249	0.000023	239607.60	371.44	45278.58	42.48	643.61	4.24	13763.09	79.70	682.39	5.40	0.017003	0.000015	5.464E-10	28.201	FC-2	sanidine	0.00054	0.00014	0.01210	0.00005	0.000224	0.000016	0.000702	0.000012	0.000020	0.000001	0.0002702	0.0000004	263.00	2.32
01H	0.71	53.552	1.163	0.0303	0.0002	24.703	36.444	1.7463	0.0385	0.20893	0.00041	0.002127	0.000026	187189.40	318.84	39403.16	38.66	566.34	3.77	14331.58	82.42	547.76	4.81	0.017003	0.000015	5.464E-10	28.201	FC-2	sanidine	0.00054	0.00014	0.01210	0.00005	0.000224	0.000016	0.000702	0.000012	0.000020	0.000001	0.0002702	0.0000004	263.00	2.32
01I	0.77	51.413	1.420	0.0269	0.0002	28.908	33.364	1.6756	0.0469	0.19933	0.00028	0.002231	0.000031	194305.45	177.86	39072.88	39.06	564.25	4.31	15980.69	92.20	600.30	5.95	0.017003	0.000015	5.464E-10	28.201	FC-2	sanidine	0.00054	0.00014	0.01210	0.00005	0.000224	0.000016	0.000702	0.000012	0.000020	0.000001	0.0002702	0.0000004	263.00	2.32
01J	0.84	51.869	1.287	0.0238	0.0001	33.781	31.970	1.6907	0.0425	0.18929	0.00027	0.002278	0.000027	237115.15	215.44	45348.44	46.73	678.35	4.48	20963.97	119.83	759.12	6.18	0.017003	0.000015	5.464E-10	28.201	FC-2	sanidine	0.00054	0.00014	0.01210	0.00005	0.000224	0.000016	0.000702	0.000012	0.000020	0.000001	0.0002702	0.0000004	263.00	2.32
01K	0.91	52.561	1.340	0.0224	0.0001	37.515	36.113	1.7135	0.0443	0.21099	0.00030	0.002138	0.000031	163026.47	153.14	34775.51	34.68	493.51	3.82	17006.98	97.62	526.48	4.94	0.017003	0.000015	5.464E-10	28.201	FC-2	sanidine	0.00054	0.00014	0.01210	0.00005	0.000224	0.000016	0.000702	0.000012	0.000020	0.000001	0.0002702	0.0000004	263.00	2.32
01L	0.99	53.583	1.470	0.0227	0.0001	42.169	33.340	1.7473	0.0486	0.19100	0.00026	0.002232	0.000031	224410.80	207.73	43331.67	41.76	630.85	5.15	20905.75	118.26	719.52	6.79	0.017003	0.000015	5.464E-10	28.201	FC-2	sanidine	0.00054	0.00014	0.01210	0.00005	0.000224	0.000016	0.000702	0.000012	0.000020	0.000001	0.0002702	0.0000004	263.00	2.32
01M	1.07	54.689	1.262	0.0239	0.0001	46.153	40.351	1.7839	0.0418	0.22647	0.00032	0.001996	0.000031	162061.25	150.46	37073.04	38.72	515.21	3.74	17049.84	97.32	502.04	4.98	0.017003	0.000015	5.464E-10	28.201	FC-2	sanidine	0.00054	0.00014	0.01210	0.00005	0.000224	0.000016	0.000702	0.000012	0.000020	0.000001	0.0002702	0.0000004	263.00	2.32
01N	1.15	52.561	1.243	0.0258	0.0002	49.904	40.760	1.7135	0.0411	0.23817	0.00036	0.001982	0.000033	145115.24	141.15	34877.09	38.99	481.76	3.94	14830.67	85.67	443.00	4.62	0.017003	0.000015	5.464E-10	28.201	FC-2	sanidine	0.00054	0.00014	0.01210	0.00005	0.000224	0.000016	0.000702	0.000012	0.000020	0.000001	0.0002702	0.0000004	263.00	2.32
01O	1.23	54.242	1.116	0.0286	0.0002	53.263	43.155	1.7691	0.0369	0.24425	0.00038	0.001902	0.000030	126680.17	129.81	31187.73	35.04	431.43	3.82	11997.51	72.32	366.65	3.70	0.017003	0.000015	5.464E-10	28.201	FC-2	sanidine	0.00054	0.00014	0.01210	0.00005	0.000224	0.000016	0.000702	0.000012	0.000020	0.000001	0.0002702	0.0000004	263.00	2.32
01P	1.31	51.014	1.207	0.0317	0.0002	56.380	39.926	1.6624	0.0399	0.24048	0.00037	0.002010	0.000032	119420.11	123.01	28917.10	32.28	404.43	4.09	10023.84	59.90	345.09	3.74	0.017003	0.000015	5.464E-10	28.201	FC-2	sanidine	0.00054	0.00014	0.01210	0.00005	0.000224	0.000016	0.000702	0.000012	0.000020	0.000001	0.0002702	0.0000004	263.00	2.32
01Q	1.4	52.133	1.178	0.0344	0.0002	59.532	39.368	1.6994	0.0390	0.23195	0.00039	0.002029	0.000030	125177.20	129.48	29214.86	38.15	418.00	3.42	9325.79	55.55	351.74	3.69	0.017003	0.000015	5.464E-10	28.201	FC-2	sanidine	0.00054	0.00014	0.01210	0.00005	0.000224	0.000016	0.000702	0.000012	0.000020	0.000001	0.0002702	0.0000004	263.00	2.32
01R	1.5	53.929	1.362	0.0369	0.0002	62.959	40.994	1.7588	0.0451	0.23338	0.00038	0.001975	0.000035	135296.55	135.66	31752.21	39.47	447.51	3.35	9469.86	56.11	366.42	4.69	0.017003	0.000015	5.464E-10	28.201	FC-2	sanidine	0.00054	0.00014	0.01210	0.00005	0.000224	0.000016	0.000702	0.000012	0.000020	0.000001	0.0002702	0.0000004	263.00	2.32
01S	1.65	52.039	1.165	0.0344	0.0002	67.643	38.950	1.6963	0.0385	0.22991	0.00031	0.002043	0.000029	187696.90	176.18	43422.29	40.53	626.16	4.26	13884.68	79.57	529.14	5.44	0.017003	0.000015	5.464E-10	28.201	FC-2	sanidine	0.00054	0.00014	0.01210	0.00005	0.000224	0.000016	0.000702	0.000012	0.000020	0.000001	0.0002702	0.0000004	263.00	2.32
01T	1.85	51.376	1.004	0.0306	0.0002	73.115	39.061	1.6744	0.0332	0.23359	0.00032	0.002039	0.000026	215796.79	191.90	50774.76	50.54	734.60	5.03	18202.20	102.17	631.17	5.41	0.017003	0.000015	5.464E-10	28.201	FC-2	sanidine	0.00054	0.00014	0.01210	0.00005	0.000224	0.000016	0.000702	0.000012	0.000020	0.000001	0.0002702	0.0000004	263.00	2.32
01U	2.1	49.660	1.339	0.0272	0.0002	78.956	37.264	1.6176	0.0442	0.23065	0.00030	0.002100	0.000034	233328.66	217.86	54275.25	48.78	807.65	4.60	21822.40	124.24	719.17	7.79	0.017003	0.000015	5.464E-10	28.201	FC-2	sanidine	0.00054	0.00014	0.01210	0.00005	0.000224	0.000016	0.000702	0.000012	0.000020	0.000001	0.0002702	0.0000004	263.00	2.32
01V	2.4	50.253	1.064	0.0224	0.0001	84.656	36.975	1.6372	0.0352	0.22611	0.00030	0.002109	0.000026	232220.12	220.09	53083.32	45.20	809.95	5.00	25932.32	148.83	762.47	5.88	0.017003	0.000015	5.464E-10	28.201	FC-2	sanidine	0.00054	0.00014	0.01210	0.00005	0.000224	0.000016	0.000702	0.000012	0.000020	0.000001	0.0002702	0.0000004	263.00	2.32
01W	2.7	50.950	1.467	0.0199	0.0001	90.541	37.338	1.6603	0.0485	0.22517	0.00029	0.002097	0.000036	240777.42	220.21	54900.92	47.02	840.84	5.48	30100.61	169.52	821.66	8.54	0.017003	0.000015	5.464E-10	28.201	FC-2	sanidine	0.00054	0.00014	0.01210	0.00005	0.000224	0.000016	0.000702	0.000012	0.000020	0.000001	0.0002702	0.0000004	263.00	2.32
X 01X	3.05	46.053	1.233	0.0202	0.0001	96.400	31.840	1.4987	0.0406	0.21270	0.00027	0.002282	0.000029	253728.32	218.46	54642.75	49.56	880.28	5.48	29533.92	166.09	889.72	7.03	0.017003	0.000015	5.464E-10	28.201	FC-2	sanidine	0.00054	0.00014	0.01210	0.00005	0.000224	0.000016	0.000702	0.000012	0.000020	0.000001	0.0002702	0.0000004	263.00	2.32
X 01Y	3.45	38.196	2.729	0.0247	0.0002	97.740	23.171	1.2403	0.0896	0.18700	0.00045	0.002573	0.000056	66019.20	83.49	12468.17	25.00	212.35	2.97	5513.12	34.24	227.84	3.66	0.017003	0.000015	5.464E-10	28.201	FC-2	sanidine	0.00054	0.00014	0.01210	0.00005	0.000224	0.000016	0.000702	0.000012	0.000020	0.000001	0.0002702	0.0000004	263.00	2.32
X 01Z	4	42.020	1.912	0.0306	0.0002	100.000	28.424	1.3659	0.0629	0.20833	0.00040	0.002396	0.000044	99920.82	111.45	20970.92	32.79	342.07	3.42	7507.37	46.00	318.42	4.32	0.017003	0.000015	5.464E-10	28.201	FC-2	sanidine	0.00054	0.00014	0.01210	0.00005	0.000224	0.000016	0.000702	0.000012	0.000020	0.000001	0.0002702	0.0000004	263.00	2.32

Integrated K/Ca ±2σ **0.0268** **0.0001**
Plateau Age ±2σ **52.307** **0.704** **n=17/26** **MSWD=1.398**
Total Integrated Age ±2σ **51.731** **0.645**
Isochron Age ±2σ **50.482** **4.261** **MSWD=1.454**
(^{40}Ar/^{36}Ar)$_{trapped}$ ±2σ **304.901** **15.425**

Sample: K8-18 **Identifier:** UW144:C40
Material: Groundmass

N	Power %	Age (Ma)	±1σ (Ma)	K/Ca	±1σ	Cum. %^{39}Ar (%)	%^{40}Ar (%)	^{40}Ar*/^{39}Ar$_K$	±1σ	Corrected[1] ^{39}Ar/^{40}Ar	±1σ	^{36}Ar/^{40}Ar	±1σ	^{40}Ar (cps)	±1σ	^{39}Ar (cps)	±1σ	^{38}Ar (cps)	±1σ	^{37}Ar (cps)	±1σ	^{36}Ar (cps)	±1σ	J	±1σ	LambdaK	MonitorAge	onitorNa	nitorMate	(^{40}Ar/^{39}39)$_K$	±1σ	(^{38}Ar/^{39}39)$_K$	±1σ	(^{37}Ar/^{39}39)$_K$	±1σ	(^{39}Ar/^{37}37)$_{Ca}$	±1σ	(^{38}Ar/^{37}37)$_{Ca}$	±1σ	(^{36}Ar/^{37}37)$_{Ca}$	±1σ	(^{36}Ar/^{38}38)$_{Cl}$	Ca/K
01AG	0.3	47.889	3.527	0.0675	0.0008	3.195	13.142	1.5592	0.1163	0.08432	0.00016	0.002909	0.000033	129869.51	129.54	10976.22	17.80	228.99	2.38	557.60	6.58	396.51	4.22	0.017003	0.000015	5.464E-10	28.201	FC-2	sanidine	0.00054	0.00014	0.01210	0.00005	0.000224	0.000016	0.000702	0.000012	0.000020	0.000001	0.0002702	0.0000004	263.00	2.32
01AH	0.35	56.201	2.403	0.0565	0.0005	10.328	24.931	1.8340	0.0796	0.13604	0.00021	0.002514	0.000036	179805.93	177.38	24530.77	28.22	382.52	2.96	1487.48	11.82	502.04	6.46	0.017003	0.000015	5.464E-10	28.201	FC-2	sanidine	0.00054	0.00014	0.01210	0.00005	0.000224	0.000016	0.000702	0.000012	0.000020	0.000001	0.0002702	0.0000004	263.00	2.32
01AI	0.41	50.843	3.480	0.0480	0.0004	13.928	35.452	1.6567	0.1150	0.21424	0.00043	0.002161	0.000082	57654.73	66.15	12393.82	20.45	166.88	2.36	883.27	7.35	154.36	4.74	0.017003	0.000015	5.464E-10	28.201	FC-2	sanidine	0.00054	0.00014	0.01210	0.00005	0.000224	0.000016	0.000702	0.000012	0.000020	0.000001	0.0002702	0.0000004	263.00	2.32
01AJ	0.52	53.628	1.468	0.0377	0.0002	27.655	44.182	1.7488	0.0486	0.25299	0.00033	0.001868	0.000041	186175.67	174.97	47330.83	40.81	617.09	3.30	4281.29	27.49	492.40	7.56	0.017003	0.000015	5.464E-10	28.201	FC-2	sanidine	0.00054	0.00014	0.01210	0.00005	0.000224	0.000016	0.000702	0.000012	0.000020	0.000001	0.0002702	0.0000004	263.00	2.32
01AK	0.66	51.189	1.359	0.0267	0.0002	44.605	37.812	1.6682	0.0449	0.22695	0.00026	0.002081	0.000034	256236.13	224.43	58637.28	42.07	816.76	4.22	7468.57	45.57	786.08	8.53	0.017003	0.000015	5.464E-10	28.201	FC-2	sanidine	0.00054	0.00014	0.01210	0.00005	0.000224	0.000016	0.000702	0.000012	0.000020	0.000001	0.0002702	0.0000004	263.00	2.32
01AL	0.82	52.882	1.817	0.0229	0.0001	58.786	46.119	1.7241	0.0601	0.26787	0.00035	0.001802	0.000054	181656.40	176.55	49146.35	41.47	668.15	3.89	7265.26	43.45	573.61	9.63	0.017003	0.000015	5.464E-10	28.201	FC-2	sanidine	0.00054	0.00014	0.01210	0.00005	0.000224	0.000016	0.000702	0.000012	0.000020	0.000001	0.0002702	0.0000004	263.00	2.32

01AN	1.3	51.926	1.018	0.0280	0.0002	74.396	43.927	1.6925	0.0336	0.25990	0.00034	0.001876	0.000029	206084.86	183.96	53970.33	49.08	757.88	4.73	6538.36	41.41	608.36	5.79	0.017003	0.000015	5.464E-10	28.201	FC-2	sanidine	0.00054	0.00014	0.01210	0.00005	0.000224	0.000016	0.000702	0.000012	0.000020	0.000001	0.0002702	0.0000004	263.00	2.32
01AO	1.6	50.924	1.325	0.0231	0.0001	86.673	40.356	1.6594	0.0438	0.24351	0.00031	0.001996	0.000036	172986.80	166.13	42547.65	34.18	614.61	4.36	6249.63	38.23	557.38	5.98	0.017003	0.000015	5.464E-10	28.201	FC-2	sanidine	0.00054	0.00014	0.01210	0.00005	0.000224	0.000016	0.000702	0.000012	0.000020	0.000001	0.0002702	0.0000004	263.00	2.32
01AQ	2	50.083	1.924	0.0165	0.0001	96.576	35.975	1.6316	0.0635	0.22075	0.00035	0.002143	0.000047	153906.58	179.31	34497.68	34.88	542.39	3.67	7048.52	43.43	569.42	7.02	0.017003	0.000015	5.464E-10	28.201	FC-2	sanidine	0.00054	0.00014	0.01210	0.00005	0.000224	0.000016	0.000702	0.000012	0.000020	0.000001	0.0002702	0.0000004	263.00	2.32
X 01AR	2.2	42.398	2.382	0.0191	0.0001	100.006	29.138	1.3784	0.0783	0.21164	0.00046	0.002372	0.000055	55593.24	77.57	11918.16	19.45	196.23	2.58	2107.28	14.80	203.53	3.02	0.017003	0.000015	5.464E-10	28.201	FC-2	sanidine	0.00054	0.00014	0.01210	0.00005	0.000224	0.000016	0.000702	0.000012	0.000020	0.000001	0.0002702	0.0000004	263.00	2.32
X 01AS	3.2	315.686	535.888	-0.0822	0.3562	100.000	4.460	11.0720	20.4626	0.00403	0.00068	0.003200	0.000275	-5195.37	25.93	-20.82	3.50	-3.85	0.67	0.87	3.78	-16.60	1.42	0.017003	0.000015	5.464E-10	28.201	FC-2	sanidine	0.00054	0.00014	0.01210	0.00005	0.000224	0.000016	0.000702	0.000012	0.000020	0.000001	0.0002702	0.0000004	263.00	2.32

Integrated K/Ca ±2σ **0.0272** **0.0001**
Plateau Age ±2σ **51.908** **1.077 n=9/11** **MSWD=0.997**
Total Integrated Age ±2σ **51.659** **1.121**
Isochron Age ±2σ **52.205** **2.351** **MSWD=1.133**
(^{40}Ar/^{36}Ar)$_{trapped}$ ±2σ **297.477** **7.616**

Sample: K8-8 **Identifier: UW144:C47**
Material: Groundmass

N	Power %	Age (Ma)	±1σ (Ma)	K/Ca	±1σ	Cum. %^{39}Ar (%)	%^{40}Ar (%)	^{40}Ar*/^{39}Ar$_K$	±1σ	^{39}Ar/^{40}Ar	±1σ	^{36}Ar/^{40}Ar	±1σ	Corrected[1] ^{40}Ar (cps)	±1σ	^{39}Ar (cps)	±1σ	^{38}Ar (cps)	±1σ	^{37}Ar (cps)	±1σ	^{36}Ar (cps)	±1σ	J	±1σ	LambdaK	MonitorAge	onitorNar	nitorMate	(^{40}Ar/^{39}39)$_K$	±1σ	(^{38}Ar/^{39}39)$_K$	±1σ	(^{37}Ar/^{39}39)$_K$	±1σ	(^{39}Ar/^{37}37)$_{Ca}$	±1σ	(^{38}Ar/^{37}37)$_{Ca}$	±1σ	(^{36}Ar/^{37}37)$_{Ca}$	±1σ	(^{36}Ar/^{38}38)$_{Cl}$	Ca/K
X 01A	0.26	105.994	9.640	0.0625	0.0009	0.533	17.872	3.5067	0.3283	0.05098	0.00021	0.002751	0.000056	44944.24	56.38	2298.55	9.17	53.90	1.33	391.89	5.51	127.78	2.50	0.01700	0.00002	5.464E-10	28.201	FC-2	sanidine	0.00540	0.00014	0.01210	0.00005	0.000000	0.000000	0.000695	0.000018	0.000020	0.000002	0.000265	0.000004	263.00	2.32
01B	0.28	50.602	9.966	0.0434	0.0006	1.054	8.682	1.6488	0.3292	0.05267	0.00024	0.003059	0.000058	42527.24	62.71	2251.77	9.79	51.63	1.29	551.24	7.21	135.93	2.45	0.01700	0.00002	5.464E-10	28.201	FC-2	sanidine	0.00540	0.00014	0.01210	0.00005	0.000000	0.000000	0.000695	0.000018	0.000020	0.000002	0.000265	0.000004	263.00	2.32
01C	0.3	49.719	8.320	0.0380	0.0004	1.835	10.308	1.6196	0.2747	0.06367	0.00024	0.003004	0.000058	52782.01	66.58	3381.23	12.13	75.98	1.58	943.37	8.58	168.60	3.07	0.01700	0.00002	5.464E-10	28.201	FC-2	sanidine	0.00540	0.00014	0.01210	0.00005	0.000000	0.000000	0.000695	0.000018	0.000020	0.000002	0.000265	0.000004	263.00	2.32
01D	0.32	44.208	6.271	0.0330	0.0003	2.838	10.580	1.4379	0.2065	0.07361	0.00025	0.002995	0.000051	58605.53	74.69	4345.45	13.72	90.05	1.65	1395.60	11.58	190.39	2.95	0.01700	0.00002	5.464E-10	28.201	FC-2	sanidine	0.00540	0.00014	0.01210	0.00005	0.000000	0.000000	0.000695	0.000018	0.000020	0.000002	0.000265	0.000004	263.00	2.32
01E	0.34	42.858	14.362	0.0293	0.0002	3.945	11.994	1.3935	0.4725	0.08611	0.00029	0.002948	0.000136	55327.51	81.86	4804.32	14.09	93.27	1.72	1734.72	13.42	181.58	7.52	0.01700	0.00002	5.464E-10	28.201	FC-2	sanidine	0.00540	0.00014	0.01210	0.00005	0.000000	0.000000	0.000695	0.000018	0.000020	0.000002	0.000265	0.000004	263.00	2.32
01F	0.37	43.860	4.708	0.0270	0.0002	5.434	14.614	1.4265	0.1550	0.10251	0.00032	0.002860	0.000053	62487.88	88.08	6464.39	17.91	119.48	1.95	2527.58	17.65	205.68	3.27	0.01700	0.00002	5.464E-10	28.201	FC-2	sanidine	0.00540	0.00014	0.01210	0.00005	0.000000	0.000000	0.000695	0.000018	0.000020	0.000002	0.000265	0.000004	263.00	2.32
01G	0.46	41.510	3.342	0.0264	0.0002	9.024	14.714	1.3492	0.1099	0.10912	0.00022	0.002856	0.000040	141554.41	143.89	15592.37	26.40	283.06	2.87	6235.96	40.71	470.95	5.53	0.01700	0.00002	5.464E-10	28.201	FC-2	sanidine	0.00540	0.00014	0.01210	0.00005	0.000000	0.000000	0.000695	0.000018	0.000020	0.000002	0.000265	0.000004	263.00	2.32
01H	0.57	45.717	1.663	0.0228	0.0001	17.550	27.006	1.4876	0.0548	0.18171	0.00026	0.002444	0.000033	201936.05	177.80	37091.85	40.40	558.67	4.04	17143.50	96.04	677.07	5.98	0.01700	0.00002	5.464E-10	28.201	FC-2	sanidine	0.00540	0.00014	0.01210	0.00005	0.000000	0.000000	0.000695	0.000018	0.000020	0.000002	0.000265	0.000004	263.00	2.32
01I	0.72	45.916	1.549	0.0202	0.0001	30.050	30.091	1.4942	0.0510	0.20161	0.00028	0.002340	0.000034	266872.18	241.43	54469.02	50.65	799.40	4.88	28269.44	156.74	927.52	7.70	0.01700	0.00002	5.464E-10	28.201	FC-2	sanidine	0.00540	0.00014	0.01210	0.00005	0.000000	0.000000	0.000695	0.000018	0.000020	0.000002	0.000265	0.000004	263.00	2.32
01J	0.9	44.956	1.996	0.0182	0.0001	43.863	36.772	1.4625	0.0657	0.25177	0.00035	0.002116	0.000055	236219.81	212.72	60286.82	55.50	846.57	5.50	34600.48	204.92	871.02	11.56	0.01700	0.00002	5.464E-10	28.201	FC-2	sanidine	0.00540	0.00014	0.01210	0.00005	0.000000	0.000000	0.000695	0.000018	0.000020	0.000002	0.000265	0.000004	263.00	2.32
01K	1.15	47.449	1.792	0.0166	0.0001	55.088	24.358	1.5447	0.0591	0.15782	0.00022	0.002533	0.000031	306082.83	258.44	49070.08	46.10	790.01	5.30	30813.16	170.11	1105.94	7.83	0.01700	0.00002	5.464E-10	28.201	FC-2	sanidine	0.00540	0.00014	0.01210	0.00005	0.000000	0.000000	0.000695	0.000018	0.000020	0.000002	0.000265	0.000004	263.00	2.32
01L	1.45	45.837	1.925	0.0137	0.0001	62.852	42.065	1.4916	0.0634	0.28245	0.00047	0.001938	0.000060	118369.31	115.84	34065.88	39.71	505.40	3.57	25822.11	141.61	506.94	5.48	0.01700	0.00002	5.464E-10	28.201	FC-2	sanidine	0.00540	0.00014	0.01210	0.00005	0.000000	0.000000	0.000695	0.000018	0.000020	0.000002	0.000265	0.000004	263.00	2.32
01M	1.8	42.303	3.232	0.0074	0.0000	67.281	39.402	1.3752	0.1063	0.28696	0.00066	0.002028	0.000102	66470.58	85.11	19786.87	30.45	318.74	2.84	27221.19	157.73	427.64	4.83	0.01700	0.00002	5.464E-10	28.201	FC-2	sanidine	0.00540	0.00014	0.01210	0.00005	0.000000	0.000000	0.000695	0.000018	0.000020	0.000002	0.000265	0.000004	263.00	2.32
01N	2.2	43.960	3.399	0.0059	0.0000	75.932	37.848	1.4297	0.1119	0.26510	0.00054	0.002080	0.000099	140509.73	146.84	39026.39	40.75	637.52	5.07	66644.27	360.76	1009.86	7.80	0.01700	0.00002	5.464E-10	28.201	FC-2	sanidine	0.00540	0.00014	0.01210	0.00005	0.000000	0.000000	0.000695	0.000018	0.000020	0.000002	0.000265	0.000004	263.00	2.32
01Q	4	49.449	3.440	0.0070	0.0000	80.705	39.461	1.6107	0.1136	0.24532	0.00053	0.002026	0.000093	83772.75	97.35	21370.02	29.35	319.52	3.11	30905.18	170.21	503.04	5.65	0.01700	0.00002	5.464E-10	28.201	FC-2	sanidine	0.00540	0.00014	0.01210	0.00005	0.000000	0.000000	0.000695	0.000018	0.000020	0.000002	0.000265	0.000004	263.00	2.32
01R	4.8	52.417	3.734	0.0065	0.0000	83.747	35.308	1.7088	0.1235	0.20686	0.00054	0.002165	0.000085	63304.48	84.66	13662.76	24.59	213.39	2.58	21196.82	121.17	365.85	3.93	0.01700	0.00002	5.464E-10	28.201	FC-2	sanidine	0.00540	0.00014	0.01210	0.00005	0.000000	0.000000	0.000695	0.000018	0.000020	0.000002	0.000265	0.000004	263.00	2.32
01S	5.7	50.053	2.945	0.0067	0.0000	93.497	34.405	1.6306	0.0973	0.21123	0.00038	0.002196	0.000069	198679.21	178.99	43739.25	42.82	686.57	4.89	66281.19	374.55	1151.89	7.22	0.01700	0.00002	5.464E-10	28.201	FC-2	sanidine	0.00540	0.00014	0.01210	0.00005	0.000000	0.000000	0.000695	0.000018	0.000020	0.000002	0.000265	0.000004	263.00	2.32
01T	6.7	49.703	3.891	0.0067	0.0000	99.225	33.689	1.6191	0.1285	0.20831	0.00064	0.002220	0.000089	118371.56	126.00	25692.45	64.81	406.58	3.67	38668.23	216.10	680.50	8.15	0.01700	0.00002	5.464E-10	28.201	FC-2	sanidine	0.00540	0.00014	0.01210	0.00005	0.000000	0.000000	0.000695	0.000018	0.000020	0.000002	0.000265	0.000004	263.00	2.32
01U	9	38.899	7.994	0.0063	0.0000	100.000	21.365	1.2634	0.2624	0.16927	0.00067	0.002633	0.000149	19706.76	48.05	3487.16	9.55	59.22	1.37	5610.06	34.98	112.52	2.75	0.01700	0.00002	5.464E-10	28.201	FC-2	sanidine	0.00540	0.00014	0.01210	0.00005	0.000000	0.000000	0.000695	0.000018	0.000020	0.000002	0.000265	0.000004	263.00	2.32

Integrated K/Ca ±2σ **0.0112** **0.0000**
Plateau Age ±2σ **46.159** **1.309 n=18/19** **MSWD=0.746**
Total Integrated Age ±2σ **46.677** **1.466**
Isochron Age ±2σ **46.345** **2.695** **MSWD=0.793**
(^{40}Ar/^{36}Ar)$_{trapped}$ ±2σ **298.068** **6.187**

Sample: K8-10 **Identifier: UW144:C45**
Material: Groundmass

N	Power %	Age (Ma)	±1σ (Ma)	K/Ca	±1σ	Cum. %^{39}Ar (%)	%^{40}Ar (%)	^{40}Ar*/^{39}Ar$_K$	±1σ	^{39}Ar/^{40}Ar	±1σ	^{36}Ar/^{40}Ar	±1σ	Corrected[1] ^{40}Ar (cps)	±1σ	^{39}Ar (cps)	±1σ	^{38}Ar (cps)	±1σ	^{37}Ar (cps)	±1σ	^{36}Ar (cps)	±1σ	J	±1σ	LambdaK	MonitorAge	onitorNar	nitorMate	(^{40}Ar/^{39}39)$_K$	±1σ	(^{38}Ar/^{39}39)$_K$	±1σ	(^{37}Ar/^{39}39)$_K$	±1σ	(^{39}Ar/^{37}37)$_{Ca}$	±1σ	(^{38}Ar/^{37}37)$_{Ca}$	±1σ	(^{36}Ar/^{37}37)$_{Ca}$	±1σ	(^{36}Ar/^{38}38)$_{Cl}$	Ca/K
X 01E	0.34	48.763	12.577	0.1938	0.0050	0.407	3.929	1.5881	0.4151	0.02474	0.00008	0.003218	0.000034	170420.49	162.93	4217.50	12.56	156.43	2.40	250.36	6.35	550.79	5.81	0.017003	0.000015	5.464E-10	28.201	FC-2	sanidine	0.00540	0.00014	0.01210	0.00005	0.000000	0.000000	0.000695	0.000018	0.000020	0.000002	0.000265	0.000004	263.00	2.32
X 01F	0.36	40.796	5.160	0.1846	0.0016	2.327	4.713	1.3257	0.1696	0.03556	0.00006	0.003192	0.000020	560113.90	455.95	19919.88	28.54	597.77	4.36	1240.24	10.68	1799.60	11.05	0.017003	0.000015	5.464E-10	28.201	FC-2	sanidine	0.00540	0.00014	0.01210	0.00005	0.000000	0.000000	0.000695	0.000018	0.000020	0.000002	0.000265	0.000004	263.00	2.32
X 01G	0.38	42.141	5.220	0.1763	0.0015	4.092	5.576	1.3699	0.1716	0.04071	0.00007	0.003163	0.000023	449858.42	396.96	18318.31	27.54	497.93	3.72	1193.56	9.90	1434.28	10.33	0.017003	0.000015	5.464E-10	28.201	FC-2	sanidine	0.00540	0.00014	0.01210	0.00005	0.000000	0.000000	0.000695	0.000018	0.000020	0.000002	0.000265	0.000004	263.00	2.32
X 01H	0.4	51.501	3.856	0.1659	0.0013	6.447	8.700	1.6785	0.1275	0.05185	0.00008	0.003058	0.000022	471042.28	406.98	24428.20	32.11	573.76	4.47	1690.09	12.62	1456.82	10.22	0.017003	0.000015	5.464E-10	28.201	FC-2	sanidine	0.00540	0.00014	0.01210	0.00005	0.000000	0.000000	0.000695	0.000018	0.000020	0.000002	0.000265	0.000004	263.00	2.32
X 01I	0.42	56.992	2.958	0.1539	0.0012	9.023	11.782	1.8603	0.0981	0.06336	0.00010	0.002955	0.000021	421852.78	380.72	26736.39	34.63	559.91	4.58	1992.39	15.22	1265.82	8.58	0.017003	0.000015	5.464E-10	28.201	FC-2	sanidine	0.00540	0.00014	0.01210	0.00005	0.000000	0.000000	0.000695	0.000018	0.000020	0.000002	0.000265	0.000004	263.00	2.32
X 01J	0.44	51.442	3.300	0.1479	0.0011	11.242	11.085	1.6765	0.1091	0.06615	0.00010	0.002978	0.000024	347948.63	319.70	23023.59	28.69	462.35	4.21	1784.07	13.46	1053.58	8.26	0.017003	0.000015	5.464E-10	28.201	FC-2	sanidine	0.00540	0.00014	0.01210	0.00005	0.000000	0.000000	0.000695	0.000018	0.000020	0.000002	0.000265	0.000004	263.00	2.32
X 01K	0.46	52.523	2.645	0.1360	0.0010	13.617	13.222	1.7123	0.0875	0.07725	0.00013	0.002906	0.000022	319042.99	289.00	24658.96	33.44	471.75	3.98	2076.49	15.44	947.55	7.08	0.017003	0.000015	5.464E-10	28.201	FC-2	sanidine	0.00540	0.00014	0.01210	0.00005	0.000000	0.000000	0.000695	0.000018	0.000020	0.000002	0.000265	0.000004	263.00	2.32
X 01L	0.49	59.419	2.405	0.1282	0.0009	16.478	17.108	1.9407	0.0798	0.08819	0.00012	0.002776	0.000023	336589.60	291.51	29700.81	30.98	539.70	3.94	2652.44	18.15	960.42	7.80	0.017003	0.000015	5.464E-10	28.201	FC-2	sanidine	0.00540	0.00014	0.01210	0.00005	0.000000	0.000000	0.000695	0.000018	0.000020	0.000002	0.000265	0.000004	263.00	2.32
X 01M	0.52	54.848	1.892	0.1126	0.0007	19.837	18.670	1.7892	0.0626	0.10441	0.00015	0.002724	0.000022	333788.12	285.67	34877.34	37.98	569.30	4.85	3542.15	22.16	943.96	7.16	0.017003	0.000015	5.464E-10	28.201	FC-2	sanidine	0.00540	0.00014	0.01210	0.00005	0.000000	0.000000	0.000695	0.000018	0.000020	0.000002	0.000265	0.000004	263.00	2.32
X 01N	0.55	56.881	1.714	0.1004	0.0006	23.794	22.154	1.8566	0.0568	0.11940	0.00015	0.002607	0.000022	343947.32	294.11	41111.21	37.41	646.33	4.06	4679.80	29.42	942.77	7.65	0.017003	0.000015	5.464E-10	28.201	FC-2	sanidine	0.00540	0.00014	0.01210	0.00005	0.000000	0.000000	0.000695	0.000018	0.000020	0.000002	0.000265	0.000004	263.00	2.32
X 01O	0.58	56.116	1.661	0.0898	0.0006	27.196	23.572	1.8312	0.0550	0.12881	0.00018	0.002559	0.000023	274041.99	241.86	35347.03	38.87	541.34	4.05	4491.37	30.04	745.73	6.36	0.017003	0.000015	5.464E-10	28.201	FC-2	sanidine	0.00540	0.00014	0.01210	0.00005	0.000000	0.000000	0.000695	0.000018	0.000020	0.000002	0.000265	0.000004	263.00	2.32
X 01P	0.61	56.393	1.661	0.0800	0.0005	30.390	23.465	1.8404	0.0551	0.12759	0.00018	0.002563	0.000023	259773.97	224.39	33201.38	37.57	509.73	3.53	4731.10	30.17	712.60	5.97	0.017003	0.000015	5.464E-10	28.201	FC-2	sanidine	0.00540	0.00014	0.01210	0.00005	0.000000	0.000000	0.000695	0.000018	0.000020	0.000002	0.000265	0.000004	263.00	2.32
X 01Q	0.64	52.013	1.632	0.0692	0.0004	33.576	21.707	1.6954	0.0540	0.12812	0.00019	0.002622	0.000023	258097.15	232.02	33144.19	37.88	520.15	4.16	5452.45	34.04	730.74	5.81	0.017003	0.000015	5.464E-10	28.201	FC-2	sanidine	0.00540	0.00014	0.01210	0.00005	0.000000	0.000000	0.000695	0.000018	0.000020	0.000002	0.000265	0.000004	263.00	2.32
X 01R	0.68	55.865	1.513	0.0623	0.0004	36.800	22.851	1.8229	0.0501	0.12544	0.00019	0.002584	0.000021	266742.29	236.67	33554.24	39.81	536.30	4.08	6128.48	37.91	749.99	5.41	0.017003	0.000015	5.464E-10	28.201	FC-2	sanidine	0.00540	0.00014	0.01210	0.00005	0.000000	0.000000	0.000695	0.000018	0.000020	0.000002	0.000265	0.000004	263.00	2.32
01S	0.72	49.862	1.877	0.0548	0.0004	40.452	18.641	1.6243	0.0620	0.11483	0.00018	0.002725	0.000024	330006.50	412.54	38029.02	34.18	641.40	4.27	7888.18	64.04	977.57	7.56	0.017003	0.000015	5.464E-10	28.201	FC-2	sanidine	0.00540	0.00014	0.01210	0.00005	0.000000	0.000000	0.000695	0.000018	0.000020	0.000002	0.000265	0.000004	263.00	2.32
01T	0.76	49.930	1.890	0.0498	0.0003	43.442	16.824	1.6266	0.0624	0.10349	0.00015	0.002786	0.000021	299728.55	277.23	31148.36	32.79	528.71	4.70	7098.33	42.89	905.56	6.25	0.017003	0.000015	5.464E-10	28.201	FC-2	sanidine	0.00540	0.00014	0.01210	0.00005	0.000000	0.000000	0.000695	0.000018	0.000020	0.000002	0.000265	0.000004	263.00	2.32
01U	0.8	48.820	1.918	0.0464	0.0003	46.797	16.608	1.5899	0.0633	0.10452	0.00016	0.002793	0.000022	333111.60	298.19	34975.08	41.33	608.17	4.25	8536.66	49.79	1015.37	7.11	0.017003	0.000015	5.464E-10	28.201	FC-2	sanidine	0.00540	0.00014	0.01210	0.00005	0.000000	0.000000	0.000695	0.000018	0.000020	0.000002	0.000265	0.000004	263.00	2.32
01V	0.84	49.256	2.301	0.0424	0.0003	49.797	14.780	1.6043	0.0760	0.09217	0.00014	0.002854	0.000023	337771.51	285.62	31296.45	37.75	566.78	4.26	8365.43	48.82	1047.48	7.67	0.017003	0.000015	5.464E-10	28.201	FC-2	sanidine	0.00540	0.00014	0.01210	0.00005	0.000000	0.000000	0.000695	0.000018	0.000020	0.000002	0.000265	0.000004	263.00	2.32
01W	0.88	49.572	2.395	0.0386	0.0002	52.874	12.671	1.6148	0.0791	0.07851	0.00012	0.002925	0.000021	406571.48	351.60	32110.40	37.16	635.72	4.17	9410.29	54.77	1283.10	8.13	0.017003	0.000015	5.464E-10	28.201	FC-2	sanidine	0.00540	0.00014	0.01210	0.00005	0.000000	0.000000	0.000695	0.000018	0.000020	0.000002	0.000265	0.000004	263.00	2.32
01X	0.92	46.283	2.867	0.0351	0.0002	56.002	10.347	1.5063	0.0945	0.06872	0.00010	0.003003	0.000021	472192.86	391.04	32671.68	39.02	693.75	4.95	10522.85	64.21	1523.04	9.92	0.017003	0.000015	5.464E-10	28.201	FC-2	sanidine	0.00540	0.00014	0.01210	0.00005	0.000000	0.000000	0.000695	0.000018	0.000020	0.000002	0.000265	0.000004	263.00	2.32
01Y	0.96	45.104	3.150	0.0330	0.0002	58.560	10.310	1.4674	0.1038	0.07029	0.00011	0.003004	0.000024	377556.99	323.39	26732.94	36.05	563.89	3.87	9145.13	61.92	1225.66	8.95	0.017003	0.000015	5.464E-10	28.201	FC-2	sanidine	0.00540	0.00014	0.01210	0.00005	0.000000	0.000000	0.000695	0.000018	0.000020	0.000002	0.000265	0.000004	263.00	2.32
01Z	1	45.936	2.946	0.0319	0.0002	61.149	10.956	1.4948	0.0971	0.07332	0.00011	0.002982	0.000024	366345.42	324.92	27067.24	33.74	565.56	4.64	9563.32	58.87	1188.32	8.44	0.017003	0.000015	5.464E-10	28.201	FC-2	sanidine	0.00540	0.00014	0.01210	0.00005	0.000000	0.000000	0.000695	0.000018	0.000020	0.000002	0.000265	0.000004	263.00	2.32
01AA	1.05	46.851	3.496	0.0310	0.0002	63.652	11.018	1.5250	0.1153	0.07228	0.00011	0.002980	0.000028	359269.16	310.04	26174.88	32.47	554.98	4.04	9511.18	56.88	1166.01	9.78	0.017003	0.000015	5.464E-10	28.201	FC-2	sanidine	0.00540	0.00014	0.01210	0.00005	0.000000	0.000000	0.000695	0.000018	0.000020	0.000002	0.000265	0.000004	263.00	2.32
01AB	1.1	46.616	2.937	0.0303	0.0002	66.007	11.631	1.5172	0.0968	0.07669	0.00012	0.002960	0.000025	318574.27	283.57	24630.92	30.07	514.25	4.16	9132.27	55.05	1034.47	7.65	0.017003	0.000015	5.464E-10	28.201	FC-2	sanidine	0.00540	0.00014	0.01210	0.00005	0.000000	0.000000	0.000695	0.000018	0.000020	0.000002	0.000265	0.000004	263.00	2.32
01AC	1.2	47.989	2.890	0.0301	0.0002	68.961	11.340	1.5625	0.0953	0.07260	0.00011	0.002969	0.000023	422135.41	356.47	30900.65	36.60	675.72	4.60	11541.38	64.54	1369.32	9.44	0.017003	0.000015	5.464E-10	28.201	FC-2	sanidine	0.00540	0.00014	0.01210	0.00005	0.000000	0.000000	0.000695	0.000018	0.000020	0.000002	0.000265	0.000004	263.00	2.32
01AD	1.25	49.494	2.693	0.0303	0.0002	71.692	12.625	1.6122	0.0889	0.07834	0.00012	0.002926	0.000023	361741.80	300.38	28569.28	33.89	598.44	4.61	10574.62	61.28	1164.78	8.12	0.017003	0.000015	5.464E-10	28.201	FC-2	sanidine	0.00540	0.00014	0.01210	0.00005	0.000000	0.000000	0.000695	0.000018	0.000020	0.000002	0.000265	0.000004	263.00	2.32
01AE	1.3	45.265	2.809	0.0302	0.0002	74.424	12.047	1.4727	0.0925	0.08184	0.00013	0.002946	0.000025	346324.03	300.52	28573.49	35.69	592.25	4.26	10627.68	62.02	1126.95	8.48	0.017003	0.000015	5.464E-10	28.201	FC-2	sanidine	0.00540	0.00014	0.01210	0.00005	0.000000	0.000000	0.000695	0.000018	0.000020	0.000002	0.000265	0.000004	263.00	2.32
01AF	1.35	50.533	2.968	0.0296	0.0002	76.470	14.085	1.6465	0.0980	0.08559	0.00015	0.002877	0.000028	248023.93	213.00	21404.55	31.26	430.67	3.13	8117.59	48.03	795.29	6.76	0.017003	0.000015	5.464E-10	28.201	FC-2	sanidine	0.00540	0.00014	0.01210	0.00005	0.000000	0.000000	0.000695	0.000018	0.000020	0.000002	0.000265	0.000004	263.00	2.32
01AG	1.4	45.707	3.139	0.0292	0.0002	78.394	13.159	1.4873	0.1034	0.08852	0.00016	0.002908	0.000030	225551.23	252.59	20134.29	27.98	404.27	3.57	7733.05	45.81	733.81	6.71	0.017003	0.000015	5.464E-10	28.201	FC-2	sanidine	0.00540	0.00014	0.01210	0.00005	0.000000	0.000000	0.000695	0.000018	0.000020	0.000002	0.000265	0.000004	263.00	2.32
01AH	1.45	50.943	2.729	0.0290	0.0002	80.192	13.666	1.6600	0.0902	0.08236	0.00014	0.002891	0.000025	226461.95	208.76	18810.73	27.32	392.61	3.37	7258.48	42.87	727.89	5.41	0.017003	0.000015	5.464E-10	28.201	FC-2	sanidine	0.00540	0.00014	0.01210	0.00005	0.000000	0.000000	0.000695	0.000018	0.000020	0.000002	0.000265	0.000004	263.00	2.32
01AI	1.5	50.871	2.972	0.0288	0.0002	81.882	15.143	1.6577	0.0982	0.09140	0.00016	0.002842	0.000030	191825.40	180.57	17682.44	25.94	356.05	3.22	6874.11	42.30	614.41	5.59	0.017003	0.000015	5.464E-10	28.201	FC-2	sanidine	0.00540	0.00014	0.01210	0.00005	0.000000	0.000000	0.000695	0.000018	0.000020	0.000002	0.000265	0.000004	263.00	2.32
01AJ	1.57	49.349	3.004	0.0282	0.0002	82.795	10.218	1.6074	0.0992	0.06359	0.00016	0.003007	0.000021	148951.53	135.39	9556.76	22.70	233.79	2.69	3789.23	24.75	486.11	3.01	0.017003	0.000015	5.464E-10	28.201	FC-2	sanidine	0.00540	0.00014	0.01210	0.00005	0.000000	0.000000	0.000695	0.000018	0.000020	0.000002	0.000265	0.000004	263.00	2.32
01AK	1.64	47.502	6.261	0.0245	0.0002	83.893	12.516	1.5464	0.2065	0.08097	0.00018	0.002930	0.000056	140751.49	140.81	11516.38	21.62	249.71	2.53	5253.89	31.38	465.44	7.80	0.017003	0.000015	5.464E-10	28.201	FC-2	sanidine	0.00540	0.00014	0.01210	0.00005	0.000000	0.000000	0.000695	0.000018	0.000020	0.000002	0.000265	0.000004	263.00	2.32
01AL	1.72	44.423	4.016	0.0259	0.0002	84.862	13.469	1.4450	0.1322	0.09326	0.00024	0.002898	0.000041	107812.84	114.89	10152.07	23.00	199.75	3.00	4371.43	27.86	356.59	4.36	0.017003	0.000015	5.464E-10	28.201	FC-2	sanidine	0.00540	0.00014	0.01210	0.00005	0.000000	0.000000	0.000695	0.000018	0.000020	0.000002	0.000265	0.000004	263.00	2.32
01AM	1.85	47.725	2.818	0.0205	0.0001	86.465	15.649	1.5538	0.0929	0.10077	0.00018	0.002825	0.000031	165005.18	160.06	16839.06	24.37	334.90	3.43	9142.16	52.55	558.57	4.90	0.017003	0.000015	5.464E-10	28.201	FC-2	sanidine	0.00540	0.00014	0.01210	0.00005	0.000000	0.000000	0.000695	0.000018	0.000020	0.000002	0.000265	0.000004	263.00	2.32
01AO	2.2	45.640	3.114	0.0135	0.0001	88.591	17.416	1.4851	0.1026	0.11735	0.00022	0.002766	0.000040	188025.10	183.07	22511.21	32.32	419.44	3.37	18408.33	102.30	706.48	6.90	0.017003	0.000015	5.464E-10	28.201	FC-2	sanidine	0.00540	0.00014	0.01210	0.00005	0.000000	0.000000	0.000695	0.000018	0.000020	0.000002	0.000265	0.000004	263.00	2.32
01AQ	3	49.899	3.337	0.0072	0.0000	92.552	18.618	1.6256	0.1102	0.11461	0.00020	0.002725	0.000042	358633.36	309.58	42737.65	42.55	801.00	4.63	64494.19	357.57	1631.82	10.78	0.017003	0.000015	5.464E-10	28.201	FC-2	sanidine	0.00540	0.00014	0.01210	0.00005	0.000000	0.000000	0.000695	0.000018	0.000020	0.000002	0.000265	0.000004	263.00	2.32
01AS	4.4	45.379	3.367	0.0083	0.0000	96.400	13.781	1.4765	0.1109	0.09339	0.00016	0.002888	0.000035	427555.93	363.95	41298.12	43.45	784.98	5.02	54162.63	288.04	1784.53	11.78	0.017003	0.000015	5.464E-10	28.201	FC-2	sanidine	0.00540	0.00014	0.01210	0.00005	0.000000	0.000000	0.000695	0.000018	0.000020	0.000002	0.000265	0.000004	263.00	2.32
01AV	8	44.328	4.740	0.0080	0.0001	97.526	13.975	1.4419	0.1561	0.09697	0.00023	0.002881	0.000051	120460.81	128.96	12095.52	21.74	230.57	2.88	16339.09	93.99	513.20	5.45	0.017003	0.000015	5.464E-10	28.201	FC-2	sanidine	0.00540	0.00014	0.01210	0.00005	0.000000	0.000000	0.000695	0.000018	0.000020	0.000002	0.000265	0.000004	263.00	2.32
01AW	10	46.539	3.619	0.0085	0.0001	100.000	12.645	1.5147	0.1193	0.08352	0.00016	0.002926	0.000033	307369.50	276.16	26528.60	35.16	535.39	3.83	33808.97	184.28	1243.14	8.53	0.017003	0.000015	5.464E-10	28.201	FC-2	sanidine	0.00540	0.00014	0.01210	0.00005	0.000000	0.000000	0.000695	0.000018	0.000020	0.000002	0.000265	0.000004	263.00	2.32

Integrated K/Ca ±2σ **0.0205** **0.0001**
Plateau Age ±2σ **48.225** **1.100 n=26/40** **MSWD=*0.508**
Total Integrated Age ±2σ **49.984** **0.923**
Isochron Age ±2σ **50.878** **5.353** **MSWD=*0.496**
(^{40}Ar/^{36}Ar)$_{trapped}$ ±2σ **295.902** **5.385**

Sample: K8-11 **Identifier: UW144:C44**
Material: Plagioclase **Location: 0.000,0.000**

N	Power %	Age (Ma)	±1σ (Ma)	K/Ca	±1σ	Cum. %^{39}Ar (%)	%^{40}Ar (%)	^{40}Ar*/^{39}Ar$_K$	±1σ	^{39}Ar/^{40}Ar	±1σ	^{36}Ar/^{40}Ar	±1σ	Corrected[1] ^{40}Ar (cps)	±1σ	^{39}Ar (cps)	±1σ	^{38}Ar (cps)	±1σ	^{37}Ar (cps)	±1σ	^{36}Ar (cps)	±1σ	J	±1σ	LambdaK	MonitorAge	onitorNar	nitorMate	(^{40}Ar/^{39}39)$_K$	±1σ	(^{38}Ar/^{39}39)$_K$	±1σ	(^{37}Ar/^{39}39)$_K$	±1σ	(^{39}Ar/^{37}37)$_{Ca}$	±1σ	(^{38}Ar/^{37}37)$_{Ca}$	±1σ	(^{36}Ar/^{37}37)$_{Ca}$	±1σ	(^{36}Ar/^{38}38)$_{Cl}$	Ca/K
X 01C	0.32	28.262	12.798	0.3174	0.0114	0.893	2.220	0.9153	0.4177	0.02426	0.00009	0.003275	0.000034	156505.66	145.62	3794.22	13.01	148.73	1.88	139.58	5.00	513.87	5.26	0.017003	0.000015	5.464E-10	28.201	FC-2	sanidine	0.00540	0.00014	0.01210	0.00005	0.000000	0.000000	0.000695	0.000018	0.000020	0.000002	0.000265	0.000004	263.00	2.32
X 01D	0.35	11.479	10.720	0.2347	0.0033	2.872	0.792	0.3700	0.3467	0.02141	0.00005	0.003323	0.000025	393063.29	349.18	8412.47	19.38	352.40	3.10	418.24	5.73	1310.05	9.60	0.017003	0.000015	5.464E-10	28.201	FC-2	sanidine	0.00540	0.00014	0.01210	0.00005	0.000000	0.000000	0.000695	0.000018	0.000020	0.000002	0.000265	0.000004	263.00	2.32
X 01E	0.38	17.948	9.406	0.1016	0.0010	4.749	1.596	0.5796	0.3052	0.02754	0.00007	0.003296	0.000028	289696.75	261.29	7991.34	18.51	280.02	2.93	916.21	8.71	963.66	8.05	0.017003	0.000015	5.464E-10	28.201	FC-2	sanidine	0.00540	0.00014	0.01210	0.00005	0.000000	0.000000	0.000695	0.000018	0.000020	0.000002	0.000265	0.000004	263.00	2.32
X 01F	0.41	5.108	10.178	0.0642	0.0005	6.605	0.352	0.1644	0.3280	0.02142	0.00005	0.003338	0.000023	368436.72	302.73	7917.56	17.61	333.96	3.34	1433.46	11.67	1243.60	8.51	0.017003	0.000015	5.464E-10	28.201	FC-2	sanidine	0.00540	0.00014	0.01210	0.00005	0.000000	0.000000	0.000695	0.000018	0.000020	0.000002	0.000265	0.000004	263.00	2.32
X 01G	0.44	10.030	8.651	0.0419	0.0003	8.870	0.864	0.3232	0.2795	0.02674	0.00006	0.003320	0.000025	360160.13	297.85	9684.32	20.41	342.14	3.10	2677.30	18.95	1221.97	8.86	0.017003	0.000015	5.464E-10	28.201	FC-2	sanidine	0.00540	0.00014	0.01210	0.00005	0.000000	0.000000	0.000695	0.000018	0.000020	0.000002	0.000265	0.000004	263.00	2.32
X 01H	0.47	15.585	7.161	0.0282	0.0002	11.025	1.529	0.5030	0.2321	0.03041	0.00007	0.003298	0.000023	301304.91	265.07	9246.02	20.33	312.24	3.16	3788.99	25.10	1030.76	6.96	0.017003	0.000015	5.464E-10	28.201	FC-2	sanidine	0.00540	0.00014	0.01210	0.00005	0.000000	0.000000	0.000695	0.000018	0.000020	0.000002	0.000265	0.000004	263.00	2.32
X 01I	0.5	30.841	6.062	0.0191	0.0001	13.358	4.542	0.9995	0.1981	0.04545	0.00010	0.003197	0.000030	218206.73	196.52	10057.26	20.46	260.74	2.85	6031.62	37.54	756.67	6.43	0.017003	0.000015	5.464E-10	28.201	FC-2	sanidine	0.00540	0.00014	0.01210	0.00005	0.000000	0.000000	0.000695	0.000018	0.000020	0.000002	0.000265	0.000004	263.00	2.32
X 01J	0.54	31.246	5.651	0.0125	0.0001	15.573	5.712	1.0127	0.1847	0.05643	0.00014	0.003158	0.000035	166962.87	164.29	9631.82	19.70	221.40	2.62	8784.37	53.93	613.33	5.60	0.017003	0.000015	5.464E-10	28.201	FC-2	sanidine	0.00540	0.00014	0.01210	0.00005	0.000000	0.000000	0.000695	0.000018	0.000020	0.000002	0.000265	0.000004	263.00	2.32
X 01K	0.58	27.754	5.955	0.0096	0.0001	18.222	4.441	0.8987	0.1943	0.04943	0.00012	0.003201	0.000032	227823.61	208.59	11593.62	23.25	291.54	2.89	13579.19	79.93	862.34	6.92	0.017003	0.000015	5.464E-10	28.201	FC-2	sanidine	0.00540	0.00014	0.01210	0.00005	0.000000	0.000000	0.000695	0.000018	0.000020	0.000002	0.000265	0.000004	263.00	2.32
X 01L	0.62	36.665	6.878	0.0067	0.0000	20.773	5.074	1.1901	0.2255	0.04265	0.00011	0.003179	0.000032	254295.79	236.27	11309.44	21.48	300.36	3.65	18689.52	108.68	991.94	7.54	0.017003	0.000015	5.464E-10	28.201	FC-2	sanidine	0.00540	0.00014	0.01210	0.00005	0.000000	0.000000	0.000695	0.000018	0.000020	0.000002	0.000265	0.000004	263.00	2.32
X 01M	0.66	33.791	6.192	0.0056	0.0000	23.321	7.902	1.0960	0.2027	0.07213	0.00020	0.003085	0.000049	150215.76	151.27	11398.80	23.06	236.10	2.59	22591.08	130.06	685.24	6.38	0.017003	0.000015	5.464E-10	28.201	FC-2	sanidine	0.00540	0.00014	0.01210	0.00005	0.000000	0.000000	0.000695	0.000018	0.000020	0.000002	0.000265	0.000004	263.00	2.32
X 01N	0.72	28.139	3.617	0.0209	0.0001	30.233	5.242	0.9112	0.1180	0.05754	0.00009	0.003174	0.000022	510751.26	448.42	29762.54	36.69	681.81	4.38	16299.51	98.63	1780.81	11.09	0.017003	0.000015	5.464E-10	28.201	FC-2	sanidine	0.00540	0.00014	0.01210	0.00005	0.000000	0.000000	0.000695	0.000018	0.000020	0.000002	0.000265	0.000004	263.00	2.32
01O	0.8	48.361	7.125	0.0059	0.0000	33.754	16.808	1.5748	0.2351	0.10679	0.00025	0.002786	0.000084	140220.03	147.82	15704.39	23.96	291.75	2.53	29408.52	184.59	679.82	10.77	0.017003	0.000015	5.464E-10	28.201	FC-2	sanidine	0.00540	0.00014	0.01210	0.00005	0.000000	0.000000	0.000695	0.000018	0.000020	0.000002	0.000265	0.000004	263.00	2.32
01P	0.88	40.209	9.552	0.0034	0.0000	37.111	11.103	1.3064	0.3138	0.08503	0.00027	0.002977	0.000089	167935.05	162.15	15509.62	27.00	313.62	3.28	48714.79	286.70	979.29	12.80	0.017003	0.000015	5.464E-10	28.201	FC-2	sanidine	0.00540	0.00014	0.01210	0.00005	0.000000	0.000000	0.000695	0.000018	0.000020	0.000002	0.000265	0.000004	263.00	2.32
01Q	0.98	48.908	6.265	0.0032	0.0000	40.963	22.983	1.5928	0.2068	0.14440	0.00045	0.002579	0.000100	113474.02	125.05	17883.57	25.29	319.31	2.93	59371.43	319.06	877.15	6.34	0.017003	0.000015	5.464E-10	28.201	FC-2	sanidine	0.00540	0.00014	0.01210	0.00005	0.000000	0.000000	0.000695	0.000018	0.000020	0.000002	0.000265	0.000004	263.00	2.32
01R	1.1	49.150	3.241	0.0077	0.0000	45.947	26.555	1.6008	0.1070	0.16603	0.00036	0.002459	0.000059	127724.42	126.30	21984.15	33.94	374.06	3.58	31929.96	174.45	628.40	5.63	0.017003	0.000015	5.464E-10	28.201	FC-2	sanidine	0.00540	0.00014	0.01210	0.00005	0.000000	0.000000	0.000695	0.000018	0.000020	0.000002	0.000265	0.000004	263.00	2.32
01S	1.25	49.212	3.356	0.0073	0.0000	51.883	22.344	1.6029	0.1108	0.13951	0.00028	0.002600	0.000052	181005.34	169.03	26237.14	34.94	460.51	4.27	40179.56	223.61	866.41	6.80	0.017003	0.000015	5.464E-10	28.201	FC-2	sanidine	0.00540	0.00014	0.01210	0.00005	0.000000	0.000000	0.000695	0.000018	0.000020	0.000002	0.000265	0.000004	263.00	2.32
01T	1.45	46.901	2.001	0.0166	0.0001	60.385	22.596	1.5266	0.0660	0.14813	0.00023	0.002592	0.000033	244168.67	221.94	36743.31	41.37	637.25	4.38	25161.43	142.27	880.51	6.83	0.017003	0.000015	5.464E-10	28.201	FC-2	sanidine	0.00540	0.00014	0.01210	0.00005	0.000000	0.000000	0.000695	0.000018	0.000020	0.000002	0.000265	0.000004	263.00	2.32
01U	1.7	45.541	1.883	0.0179	0.0001	74.565	20.624	1.4818	0.0620	0.13929	0.00018	0.002658	0.000029	433056.68	364.83	61204.72	49.42	1120.74	5.73	39016.73	218.40	1535.24	10.74	0.017003	0.000015	5.464E-10	28.201	FC-2	sanidine	0.00540	0.00014	0.01210	0.00005	0.000000	0.000000	0.000695	0.000018	0.000020	0.000002	0.000265	0.000004	263.00	2.32
01V	2	46.662	2.396	0.0148	0.0001	80.929	22.347	1.5188	0.0790	0.14726	0.00026	0.002600	0.000039	183845.01	172.88	27562.65	37.94	499.51	3.78	21158.85	120.55	686.54	6.26	0.017003	0.000015	5.464E-10	28.201	FC-2	sanidine	0.00540	0.00014	0.01210	0.00005	0.000000	0.000000	0.000695	0.000018	0.000020	0.000002	0.000265	0.000004	263.00	2.32
01W	2.4	49.750	1.923	0.0121	0.0001	91.384	32.041	1.6206	0.0635	0.19792	0.00029	0.002275	0.000042	224804.34	198.89	45488.68	43.20	735.19	4.62	42591.94	237.21	931.53	6.52	0.017003	0.000015	5.464E-10	28.201	FC-2	sanidine	0.00540	0.00014	0.01210	0.00005	0.000000	0.000000	0.000695	0.000018	0.000020	0.000002	0.000265	0.000004	263.00	2.32
01X	3	46.227	2.572	0.0119	0.0001	96.221	27.153	1.5044	0.0848	0.18066	0.00035	0.002439	0.000051	113913.82	122.65	21049.39	29.52	339.18	3.57	19952.59	114.52	474.69	4.86	0.017003	0.000015	5.464E-10	28.201	FC-2	sanidine	0.00540	0.00014	0.01210	0.00005	0.000000	0.000000	0.000695	0.000018	0.000020	0.000002	0.000265	0.000004	263.00	2.32
01Y	3.5	54.151	4.498	0.0119	0.0001	97.807	21.486	1.7661	0.1489	0.12173	0.00034	0.002629	0.000061	55419.20	69.45	6902.51	16.47	121.68	1.96	6538.93	40.17	210.25	3.18	0.017003	0.000015	5.464E-10	28.201	FC-2	sanidine	0.00540	0.00014	0.01210	0.00005	0.000000	0.000000	0.000695	0.000018	0.000020	0.000002	0.000265	0.000004	263.00	2.32

01Z	4.1	44.686	4.759	0.0116	0.0001	98.974	24.799	1.4536	0.1567	0.17076	0.00063	0.002518	0.000089	29081.93	53.63	5082.55	15.57	79.24	1.78	4925.14	32.46	121.86	2.47	0.017003	0.000015	5.464E-10	28.201	FC-2	sanidine	0.00540	0.00014	0.01210	0.00005	0.000000	0.000000	0.000695	0.000018	0.000020	0.000002	0.000265	0.000004	263.00	2.32
01AA	5.2	54.336	6.875	0.0120	0.0001	99.730	27.289	1.7723	0.2276	0.15410	0.00070	0.002435	0.000117	20846.72	51.38	3285.96	12.14	53.75	1.60	3096.32	26.16	81.34	2.38	0.017003	0.000015	5.464E-10	28.201	FC-2	sanidine	0.00540	0.00014	0.01210	0.00005	0.000000	0.000000	0.000695	0.000018	0.000020	0.000002	0.000265	0.000004	263.00	2.32
01AB	7	41.980	16.361	0.0118	0.0001	100.000	20.422	1.3646	0.5380	0.14977	0.00121	0.002665	0.000270	7680.75	37.88	1177.12	7.34	22.28	1.17	1125.51	11.10	31.59	2.06	0.017003	0.000015	5.464E-10	28.201	FC-2	sanidine	0.00540	0.00014	0.01210	0.00005	0.000000	0.000000	0.000695	0.000018	0.000020	0.000002	0.000265	0.000004	263.00	2.32

Integrated K/Ca ±2σ **0.0092** **0.0000**
Plateau Age ±2σ **47.620** **1.641** **n=14/26** **MSWD=0.592**
Total Integrated Age ±2σ **40.465** **1.837**
Isochron Age ±2σ **50.237** **7.468** **MSWD=0.605**
$(^{40}Ar/^{36}Ar)_{trapped}$ ±2σ **293.312** **15.179**

Sample: **K8-11** **Identifier:** **UW144:C44**
Material: **Plagioclase**

N	Power %	Age (Ma)	±1σ (Ma)	K/Ca	±1σ	Cum. %^{39}Ar (%)	%^{40}Ar (%)	$^{40}Ar^*/^{39}Ar_K$	±1σ	$^{39}Ar/^{40}Ar$	±1σ	$^{36}Ar/^{40}Ar$	±1σ	Corrected[1] ^{40}Ar (cps)	±1σ	^{39}Ar (cps)	±1σ	^{38}Ar (cps)	±1σ	^{37}Ar (cps)	±1σ	^{36}Ar (cps)	±1σ	J	±1σ	LambdaK	MonitorAge	onitorNar	nitorMate	$(^{40}Ar/^{39}39)_K$	±1σ	$(^{38}Ar/^{39}39)_K$	±1σ	$(^{37}Ar/^{39}39)_K$	±1σ	$(^{39}Ar/^{37}37)_{Ca}$	±1σ	$(^{38}Ar/^{37}37)_{Ca}$	±1σ	$(^{36}Ar/^{37}37)_{Ca}$	±1σ	$(^{36}Ar/^{38}38)_{Cl}$	Ca/K
X 02A	0.32	31.991	8.954	0.1227	0.0037	1.351	3.212	1.0371	0.2928	0.03097	0.00009	0.003242	0.000030	170656.78	155.16	5288.96	14.03	178.44	2.47	149.60	4.49	558.07	5.12	0.017003	0.000015	5.464E-10	28.201	FC-2	sanidine	0.00540	0.00014	0.01210	0.00005	0.000000	0.000000	0.000695	0.000018	0.000020	0.000002	0.000265	0.000004	263.00	2.32
X 02B	0.35	42.809	4.855	0.0492	0.0005	3.779	7.549	1.3919	0.1597	0.05425	0.00011	0.003097	0.000029	175151.84	167.73	9540.29	17.16	222.41	2.52	670.45	6.92	564.26	5.00	0.017003	0.000015	5.464E-10	28.201	FC-2	sanidine	0.00540	0.00014	0.01210	0.00005	0.000000	0.000000	0.000695	0.000018	0.000020	0.000002	0.000265	0.000004	263.00	2.32
X 02C	0.38	33.820	5.574	0.0285	0.0002	6.712	5.220	1.0969	0.1825	0.04760	0.00010	0.003175	0.000029	241101.92	213.41	11573.53	22.57	290.09	3.32	1398.44	10.38	811.22	6.88	0.017003	0.000015	5.464E-10	28.201	FC-2	sanidine	0.00540	0.00014	0.01210	0.00005	0.000000	0.000000	0.000695	0.000018	0.000020	0.000002	0.000265	0.000004	263.00	2.32
X 02D	0.41	30.976	4.164	0.0175	0.0001	10.242	7.017	1.0039	0.1361	0.06992	0.00013	0.003114	0.000032	197592.81	171.18	14023.57	22.39	289.04	2.90	2738.39	18.26	705.31	6.06	0.017003	0.000015	5.464E-10	28.201	FC-2	sanidine	0.00540	0.00014	0.01210	0.00005	0.000000	0.000000	0.000695	0.000018	0.000020	0.000002	0.000265	0.000004	263.00	2.32
X 02E	0.44	26.670	5.548	0.0096	0.0001	12.218	6.391	0.8633	0.1809	0.07406	0.00020	0.003135	0.000045	104430.37	105.62	7957.08	17.98	161.85	2.44	2780.40	18.96	418.89	4.40	0.017003	0.000015	5.464E-10	28.201	FC-2	sanidine	0.00540	0.00014	0.01210	0.00005	0.000000	0.000000	0.000695	0.000018	0.000020	0.000002	0.000265	0.000004	263.00	2.32
X 02F	0.47	35.058	5.215	0.0059	0.0000	15.159	8.270	1.1374	0.1708	0.07274	0.00019	0.003072	0.000041	158213.48	142.53	12070.28	23.47	247.07	2.92	6795.54	41.00	709.88	5.43	0.017003	0.000015	5.464E-10	28.201	FC-2	sanidine	0.00540	0.00014	0.01210	0.00005	0.000000	0.000000	0.000695	0.000018	0.000020	0.000002	0.000265	0.000004	263.00	2.32
X 02G	0.51	24.093	6.847	0.0038	0.0000	17.837	7.322	0.7793	0.2229	0.09400	0.00030	0.003104	0.000070	111511.04	105.59	11286.70	22.74	212.87	2.27	9592.68	55.82	662.26	5.89	0.017003	0.000015	5.464E-10	28.201	FC-2	sanidine	0.00540	0.00014	0.01210	0.00005	0.000000	0.000000	0.000695	0.000018	0.000020	0.000002	0.000265	0.000004	263.00	2.32
X 02H	0.55	29.001	6.438	0.0061	0.0000	21.103	8.492	0.9394	0.2102	0.09045	0.00021	0.003065	0.000064	141334.45	143.29	13377.42	20.97	256.41	2.86	7209.77	43.74	670.79	8.09	0.017003	0.000015	5.464E-10	28.201	FC-2	sanidine	0.00540	0.00014	0.01210	0.00005	0.000000	0.000000	0.000695	0.000018	0.000020	0.000002	0.000265	0.000004	263.00	2.32
02I	0.59	46.547	8.457	0.0045	0.0000	23.681	16.397	1.5150	0.2788	0.10830	0.00031	0.002800	0.000101	93186.60	84.74	10743.75	20.37	203.30	2.12	7815.64	45.16	518.66	8.43	0.017003	0.000015	5.464E-10	28.201	FC-2	sanidine	0.00540	0.00014	0.01210	0.00005	0.000000	0.000000	0.000695	0.000018	0.000020	0.000002	0.000265	0.000004	263.00	2.32
02J	0.64	48.080	9.382	0.0031	0.0000	26.321	15.796	1.5655	0.3095	0.10096	0.00035	0.002820	0.000105	102361.75	101.68	11303.09	22.63	214.07	2.47	11483.71	66.84	667.61	8.75	0.017003	0.000015	5.464E-10	28.201	FC-2	sanidine	0.00540	0.00014	0.01210	0.00005	0.000000	0.000000	0.000695	0.000018	0.000020	0.000002	0.000265	0.000004	263.00	2.32
02K	0.7	47.016	6.002	0.0060	0.0001	29.483	17.851	1.5304	0.1979	0.11672	0.00052	0.002751	0.000077	106060.23	122.45	12966.17	50.80	236.92	2.70	7130.14	57.65	527.07	7.11	0.017003	0.000015	5.464E-10	28.201	FC-2	sanidine	0.00540	0.00014	0.01210	0.00005	0.000000	0.000000	0.000695	0.000018	0.000020	0.000002	0.000265	0.000004	263.00	2.32
02L	0.77	47.406	5.446	0.0102	0.0001	33.189	19.190	1.5433	0.1796	0.12443	0.00028	0.002706	0.000075	116605.53	117.04	14898.98	26.33	260.64	2.86	4910.02	30.45	477.57	8.29	0.017003	0.000015	5.464E-10	28.201	FC-2	sanidine	0.00540	0.00014	0.01210	0.00005	0.000000	0.000000	0.000695	0.000018	0.000020	0.000002	0.000265	0.000004	263.00	2.32
02M	0.85	44.808	5.918	0.0058	0.0001	36.854	22.102	1.4577	0.1949	0.15175	0.00039	0.002609	0.000099	94547.48	127.63	15055.77	23.63	263.03	2.81	8610.03	73.15	530.98	7.93	0.017003	0.000015	5.464E-10	28.201	FC-2	sanidine	0.00540	0.00014	0.01210	0.00005	0.000000	0.000000	0.000695	0.000018	0.000020	0.000002	0.000265	0.000004	263.00	2.32
02N	0.97	46.754	5.058	0.0042	0.0000	42.296	23.273	1.5218	0.1668	0.15306	0.00039	0.002569	0.000085	139208.51	138.28	22752.15	29.79	408.36	3.48	17322.58	102.98	930.12	7.37	0.017003	0.000015	5.464E-10	28.201	FC-2	sanidine	0.00540	0.00014	0.01210	0.00005	0.000000	0.000000	0.000695	0.000018	0.000020	0.000002	0.000265	0.000004	263.00	2.32
02O	1.15	45.825	3.391	0.0068	0.0000	50.481	23.341	1.4912	0.1117	0.15666	0.00031	0.002567	0.000058	204589.31	188.33	33375.03	41.04	605.79	3.94	16237.34	96.26	1061.81	8.17	0.017003	0.000015	5.464E-10	28.201	FC-2	sanidine	0.00540	0.00014	0.01210	0.00005	0.000000	0.000000	0.000695	0.000018	0.000020	0.000002	0.000265	0.000004	263.00	2.32
02P	1.8	48.601	2.330	0.0152	0.0001	58.876	32.172	1.5827	0.0769	0.20349	0.00033	0.002271	0.000052	161559.83	151.69	33428.96	40.30	579.91	4.02	7450.11	48.99	613.12	7.38	0.017003	0.000015	5.464E-10	28.201	FC-2	sanidine	0.00540	0.00014	0.01210	0.00005	0.000000	0.000000	0.000695	0.000018	0.000020	0.000002	0.000265	0.000004	263.00	2.32
02Q	2.1	47.555	1.815	0.0146	0.0001	76.346	32.947	1.5482	0.0598	0.21305	0.00030	0.002245	0.000043	321169.05	309.70	69625.73	57.92	1155.61	5.67	16107.36	100.63	1253.64	10.47	0.017003	0.000015	5.464E-10	28.201	FC-2	sanidine	0.00540	0.00014	0.01210	0.00005	0.000000	0.000000	0.000695	0.000018	0.000020	0.000002	0.000265	0.000004	263.00	2.32
02R	2.2	49.347	1.841	0.0110	0.0001	93.846	45.892	1.6073	0.0608	0.28595	0.00038	0.001810	0.000058	239778.55	202.67	70196.39	47.62	1036.21	5.46	21382.14	131.52	1141.82	7.71	0.017003	0.000015	5.464E-10	28.201	FC-2	sanidine	0.00540	0.00014	0.01210	0.00005	0.000000	0.000000	0.000695	0.000018	0.000020	0.000002	0.000265	0.000004	263.00	2.32
02S	2.35	48.123	2.598	0.0115	0.0001	97.740	38.145	1.5669	0.0857	0.24376	0.00052	0.002070	0.000070	62579.48	71.74	15604.53	24.94	253.16	2.51	4570.81	31.25	280.92	3.56	0.017003	0.000015	5.464E-10	28.201	FC-2	sanidine	0.00540	0.00014	0.01210	0.00005	0.000000	0.000000	0.000695	0.000018	0.000020	0.000002	0.000265	0.000004	263.00	2.32
02T	2.5	49.759	5.326	0.0123	0.0001	98.754	31.365	1.6209	0.1759	0.19370	0.00077	0.002298	0.000114	20504.93	51.41	4057.36	11.93	66.81	1.50	1111.19	9.98	83.92	2.24	0.017003	0.000015	5.464E-10	28.201	FC-2	sanidine	0.00540	0.00014	0.01210	0.00005	0.000000	0.000000	0.000695	0.000018	0.000020	0.000002	0.000265	0.000004	263.00	2.32
02U	3.5	50.635	5.052	0.0117	0.0001	100.000	34.639	1.6499	0.1669	0.21018	0.00072	0.002188	0.000117	23217.91	45.45	4991.05	13.18	79.52	1.52	1438.62	15.05	98.48	2.57	0.017003	0.000015	5.464E-10	28.201	FC-2	sanidine	0.00540	0.00014	0.01210	0.00005	0.000000	0.000000	0.000695	0.000018	0.000020	0.000002	0.000265	0.000004	263.00	2.32

Integrated K/Ca ±2σ **0.0085** **0.0000** 74.32
Plateau Age ±2σ **48.129** **1.791** **n=13/21** **MSWD=*0.155** 840731.94
Total Integrated Age ±2σ **44.404** **1.827**
Isochron Age ±2σ **49.950** **4.528** **MSWD=*0.103**
$(^{40}Ar/^{36}Ar)_{trapped}$ ±2σ **293.143** **12.447**

Sample: **SO249 DR51-6** **Identifier:** **UW145:B23**
Material: **Groundmass**

N	Power %	Age (Ma)	±1σ (Ma)	K/Ca	±1σ	Cum. %^{39}Ar (%)	%^{40}Ar (%)	$^{40}Ar^*/^{39}Ar_K$	±1σ	Corrected[1] $^{39}Ar/^{40}Ar$	±1σ	$^{36}Ar/^{40}Ar$	±1σ	^{40}Ar (cps)	±1σ	^{39}Ar (cps)	±1σ	^{38}Ar (cps)	±1σ	^{37}Ar (cps)	±1σ	^{36}Ar (cps)	±1σ	J	±1σ	LambdaK	MonitorAge	onitorNar	nitorMate	$(^{40}Ar/^{39}39)_K$	±1σ	$(^{38}Ar/^{39}39)_K$	±1σ	$(^{37}Ar/^{39}39)_K$	±1σ	$(^{39}Ar/^{37}37)_{Ca}$	±1σ	$(^{38}Ar/^{37}37)_{Ca}$	±1σ	$(^{36}Ar/^{37}37)_{Ca}$	±1σ	$(^{36}Ar/^{38}38)_{Cl}$	Ca/K
X 01A	0.35	29.000	10.999	0.0204	0.0002	1.345	3.259	0.9379	0.3586	0.03474	0.00012	0.003240	0.000042	106677.47	108.00	3755.57	12.41	125.59	1.83	1134.22	9.81	366.77	4.42	0.017029	0.000010	5.464E-10	28.201	FC-2	sanidine	0.00054	0.00014	0.01210	0.00002	0.000224	0.000016	0.000702	0.000012	0.000020	0.000001	0.0002702	0.0000004	263.00	2.32
X 01B	0.62	41.289	2.727	0.0130	0.0001	7.249	18.397	1.3398	0.0895	0.13732	0.00025	0.002733	0.000041	118482.28	121.67	16620.73	23.34	273.22	3.12	7787.31	47.26	469.14	4.76	0.017029	0.000010	5.464E-10	28.201	FC-2	sanidine	0.00054	0.00014	0.01210	0.00002	0.000224	0.000016	0.000702	0.000012	0.000020	0.000001	0.0002702	0.0000004	263.00	2.32
01C	0.85	54.356	1.697	0.0091	0.0001	19.580	33.018	1.7702	0.0561	0.18653	0.00030	0.002243	0.000035	182165.81	162.99	35050.92	39.42	523.36	4.03	23237.22	127.10	842.57	5.83	0.017029	0.000010	5.464E-10	28.201	FC-2	sanidine	0.00054	0.00014	0.01210	0.00002	0.000224	0.000016	0.000702	0.000012	0.000020	0.000001	0.0002702	0.0000004	263.00	2.32
01D	1.15	56.768	2.306	0.0086	0.0001	34.296	41.347	1.8500	0.0763	0.22353	0.00034	0.001964	0.000057	181430.00	165.18	41912.88	41.18	608.89	4.58	29393.86	163.54	905.63	9.83	0.017029	0.000010	5.464E-10	28.201	FC-2	sanidine	0.00054	0.00014	0.01210	0.00002	0.000224	0.000016	0.000702	0.000012	0.000020	0.000001	0.0002702	0.0000004	263.00	2.32
01E	1.55	55.069	1.585	0.0092	0.0001	49.129	42.505	1.7938	0.0524	0.23698	0.00037	0.001926	0.000041	172492.33	162.06	42160.13	44.36	602.39	4.48	27824.21	159.20	852.32	6.42	0.017029	0.000010	5.464E-10	28.201	FC-2	sanidine	0.00054	0.00014	0.01210	0.00002	0.000224	0.000016	0.000702	0.000012	0.000020	0.000001	0.0002702	0.0000004	263.00	2.32
01F	2.05	51.251	2.913	0.0043	0.0000	64.314	22.135	1.6677	0.0961	0.13274	0.00023	0.002608	0.000042	315248.42	282.64	44711.84	35.63	794.31	4.21	60421.84	336.07	1952.53	11.67	0.017029	0.000010	5.464E-10	28.201	FC-2	sanidine	0.00054	0.00014	0.01210	0.00002	0.000224	0.000016	0.000702	0.000012	0.000020	0.000001	0.0002702	0.0000004	263.00	2.32
01G	2.6	53.639	5.195	0.0026	0.0000	71.819	21.317	1.7465	0.1716	0.12206	0.00034	0.002635	0.000070	169437.72	161.98	23067.38	30.43	443.91	3.41	49809.12	304.37	1378.82	10.26	0.017029	0.000010	5.464E-10	28.201	FC-2	sanidine	0.00054	0.00014	0.01210	0.00002	0.000224	0.000016	0.000702	0.000012	0.000020	0.000001	0.0002702	0.0000004	263.00	2.32
01H	3.2	53.916	7.434	0.0016	0.0000	77.880	16.026	1.7557	0.2457	0.09128	0.00037	0.002813	0.000075	182963.12	168.23	19834.41	32.79	444.44	3.62	65020.98	360.35	1732.38	11.74	0.017029	0.000010	5.464E-10	28.201	FC-2	sanidine	0.00054	0.00014	0.01210	0.00002	0.000224	0.000016	0.000702	0.000012	0.000020	0.000001	0.0002702	0.0000004	263.00	2.32
01I	3.9	53.387	8.750	0.0014	0.0000	84.804	15.697	1.7382	0.2891	0.09031	0.00038	0.002824	0.000087	211259.77	208.82	23027.16	30.51	525.11	3.80	81835.49	454.07	2129.94	16.14	0.017029	0.000010	5.464E-10	28.201	FC-2	sanidine	0.00054	0.00014	0.01210	0.00002	0.000224	0.000016	0.000702	0.000012	0.000020	0.000001	0.0002702	0.0000004	263.00	2.32
01J	4.7	61.504	7.499	0.0016	0.0000	91.874	18.366	2.0070	0.2488	0.09152	0.00036	0.002734	0.000076	212890.44	192.76	23214.08	31.33	521.75	4.35	77336.13	417.10	2032.07	13.95	0.017029	0.000010	5.464E-10	28.201	FC-2	sanidine	0.00054	0.00014	0.01210	0.00002	0.000224	0.000016	0.000702	0.000012	0.000020	0.000001	0.0002702	0.0000004	263.00	2.32
01K	5.7	65.320	8.278	0.0015	0.0000	95.241	18.827	2.1337	0.2753	0.08824	0.00039	0.002719	0.000081	105132.99	117.06	11117.36	22.34	250.52	2.76	38124.26	210.10	1000.93	7.46	0.017029	0.000010	5.464E-10	28.201	FC-2	sanidine	0.00054	0.00014	0.01210	0.00002	0.000224	0.000016	0.000702	0.000012	0.000020	0.000001	0.0002702	0.0000004	263.00	2.32
01L	7	70.921	18.953	0.0017	0.0000	96.929	21.227	2.3203	0.6322	0.09149	0.00045	0.002638	0.000193	50843.99	80.92	5495.90	15.35	121.05	2.07	17491.43	102.27	462.42	9.63	0.017029	0.000010	5.464E-10	28.201	FC-2	sanidine	0.00054	0.00014	0.01210	0.00002	0.000224	0.000016	0.000702	0.000012	0.000020	0.000001	0.0002702	0.0000004	263.00	2.32
01M	12	59.799	15.364	0.0015	0.0000	99.841	16.856	1.9504	0.5093	0.08642	0.00039	0.002785	0.000147	92849.15	109.60	9596.20	19.40	222.88	2.92	32530.04	180.12	869.32	13.21	0.017029	0.000010	5.464E-10	28.201	FC-2	sanidine	0.00054	0.00014	0.01210	0.00002	0.000224	0.000016	0.000702	0.000012	0.000020	0.000001	0.0002702	0.0000004	263.00	2.32
01N	18	106.861	62.082	0.0017	0.0000	100.000	18.014	3.5308	2.1118	0.05102	0.00073	0.002746	0.000361	8594.24	40.25	515.13	5.71	12.55	0.88	1586.60	13.20	53.40	3.09	0.017029	0.000010	5.464E-10	28.201	FC-2	sanidine	0.00054	0.00014	0.01210	0.00002	0.000224	0.000016	0.000702	0.000012	0.000020	0.000001	0.0002702	0.0000004	263.00	2.32

Integrated K/Ca ±2σ **0.0031** **0.0000**
Plateau Age ±2σ **54.955** **1.858** **n=12/14** **MSWD=0.574**
Total Integrated Age ±2σ **54.492** **2.724**
Isochron Age ±2σ **54.536** **4.174** **MSWD=0.628**
$(^{40}Ar/^{36}Ar)_{trapped}$ ±2σ **299.661** **10.087**

Sample: **SO249 DR51-6** **Identifier:** **UW145:B23**
Material: **Groundmass**

N	Power %	Age (Ma)	±1σ (Ma)	K/Ca	±1σ	Cum. %^{39}Ar (%)	%^{40}Ar (%)	$^{40}Ar^*/^{39}Ar_K$	±1σ	Corrected[1] $^{39}Ar/^{40}Ar$	±1σ	$^{36}Ar/^{40}Ar$	±1σ	^{40}Ar (cps)	±1σ	^{39}Ar (cps)	±1σ	^{38}Ar (cps)	±1σ	^{37}Ar (cps)	±1σ	^{36}Ar (cps)	±1σ	J	±1σ	LambdaK	MonitorAge	onitorNar	nitorMate	$(^{40}Ar/^{39}39)_K$	±1σ	$(^{38}Ar/^{39}39)_K$	±1σ	$(^{37}Ar/^{39}39)_K$	±1σ	$(^{39}Ar/^{37}37)_{Ca}$	±1σ	$(^{38}Ar/^{37}37)_{Ca}$	±1σ	$(^{36}Ar/^{37}37)_{Ca}$	±1σ	$(^{36}Ar/^{38}38)_{Cl}$	Ca/K
X 01Y	0.39	34.208	18.177	0.0190	0.0003	0.511	2.251	1.1079	0.5942	0.02031	0.00009	0.003274	0.000040	104538.21	113.64	2153.45	9.12	113.86	1.88	307.95	4.36	355.26	4.19	0.017029	0.000010	5.464E-10	28.201	FC-2	sanidine	0.00054	0.00014	0.01210	0.00005	0.000224	0.000016	0.000702	0.000012	0.000020	0.000001	0.0002702	0.0000004	263.00	2.32
X 01Z	0.42	16.116	11.327	0.0184	0.0002	1.335	1.640	0.5194	0.3667	0.03157	0.00010	0.003294	0.000039	108553.09	115.43	3477.41	10.47	132.49	1.90	514.00	5.20	379.34	4.17	0.017029	0.000010	5.464E-10	28.201	FC-2	sanidine	0.00054	0.00014	0.01210	0.00005	0.000224	0.000016	0.000702	0.000012	0.000020	0.000001	0.0002702	0.0000004	263.00	2.32
X 01AA	0.45	31.900	9.864	0.0177	0.0002	2.298	4.419	1.0325	0.3221	0.04280	0.00013	0.003201	0.000046	93543.76	101.70	4064.30	11.10	123.58	1.88	623.13	6.01	325.81	4.29	0.017029	0.000010	5.464E-10	28.201	FC-2	sanidine	0.00054	0.00014	0.01210	0.00005	0.000224	0.000016	0.000702	0.000012	0.000020	0.000001	0.0002702	0.0000004	263.00	2.32
X 01AB	0.48	13.962	7.224	0.0164	0.0001	3.545	3.033	0.4497	0.2336	0.06744	0.00018	0.003248	0.000053	76925.43	91.13	5273.57	12.55	120.56	1.94	870.20	7.36	286.63	4.03	0.017029	0.000010	5.464E-10	28.201	FC-2	sanidine	0.00054	0.00014	0.01210	0.00005	0.000224	0.000016	0.000702	0.000012	0.000020	0.000001	0.0002702	0.0000004	263.00	2.32
X 01AC	0.54	41.146	4.680	0.0147	0.0001	5.369	12.641	1.3352	0.1536	0.09468	0.00022	0.002926	0.000049	80101.96	92.62	7726.22	14.90	145.21	1.93	1424.55	10.17	294.65	3.85	0.017029	0.000010	5.464E-10	28.201	FC-2	sanidine	0.00054	0.00014	0.01210	0.00005	0.000224	0.000016	0.000702	0.000012	0.000020	0.000001	0.0002702	0.0000004	263.00	2.32
X 01AD	0.61	37.981	3.955	0.0132	0.0001	7.290	15.824	1.2314	0.1295	0.12851	0.00031	0.002819	0.000056	62156.95	87.73	8155.26	15.35	135.82	2.04	1659.02	11.18	245.48	3.41	0.017029	0.000010	5.464E-10	28.201	FC-2	sanidine	0.00054	0.00014	0.01210	0.00005	0.000224	0.000016	0.000702	0.000012	0.000020	0.000001	0.0002702	0.0000004	263.00	2.32
X 01AE	0.69	35.233	2.950	0.0113	0.0001	10.390	18.696	1.1414	0.0965	0.16380	0.00033	0.002723	0.000053	78693.68	95.30	13209.35	20.22	208.32	2.46	3125.47	19.77	346.73	4.05	0.017029	0.000010	5.464E-10	28.201	FC-2	sanidine	0.00054	0.00014	0.01210	0.00005	0.000224	0.000016	0.000702	0.000012	0.000020	0.000001	0.0002702	0.0000004	263.00	2.32
X 01AF	0.77	46.078	2.843	0.0096	0.0001	13.846	30.368	1.4972	0.0936	0.20285	0.00042	0.002332	0.000063	70868.55	87.89	14799.15	22.20	216.01	2.66	4102.78	25.28	339.24	4.35	0.017029	0.000010	5.464E-10	28.201	FC-2	sanidine	0.00054	0.00014	0.01210	0.00005	0.000224	0.000016	0.000702	0.000012	0.000020	0.000001	0.0002702	0.0000004	263.00	2.32
01AG	0.86	51.639	2.301	0.0088	0.0001	17.774	36.918	1.6805	0.0759	0.21971	0.00045	0.002113	0.000056	74349.24	86.74	16866.97	25.27	244.03	2.37	5115.99	30.52	374.17	3.91	0.017029	0.000010	5.464E-10	28.201	FC-2	sanidine	0.00054	0.00014	0.01210	0.00005	0.000224	0.000016	0.000702	0.000012	0.000020	0.000001	0.0002702	0.0000004	263.00	2.32
01AH	0.96	55.025	2.502	0.0084	0.0001	21.994	42.455	1.7924	0.0827	0.23690	0.00048	0.001927	0.000065	74068.76	93.50	18146.08	25.64	255.04	2.87	5750.75	33.52	386.91	4.61	0.017029	0.000010	5.464E-10	28.201	FC-2	sanidine	0.00054	0.00014	0.01210	0.00005	0.000224	0.000016	0.000702	0.000012	0.000020	0.000001	0.0002702	0.0000004	263.00	2.32
01AI	1.08	55.534	2.424	0.0082	0.0001	26.935	44.468	1.8092	0.0802	0.24582	0.00050	0.001860	0.000066	83603.73	112.63	21270.39	27.39	294.62	3.14	6886.37	53.01	448.08	4.98	0.017029	0.000010	5.464E-10	28.201	FC-2	sanidine	0.00054	0.00014	0.01210	0.00005	0.000224	0.000016	0.000702	0.000012	0.000020	0.000001	0.0002702	0.0000004	263.00	2.32
01AJ	1.22	54.830	2.362	0.0082	0.0001	32.145	45.064	1.7859	0.0781	0.25237	0.00052	0.001840	0.000066	85853.92	124.39	22423.27	27.64	313.45	2.88	7243.37	55.50	465.90	5.09	0.017029	0.000010	5.464E-10	28.201	FC-2	sanidine	0.00054	0.00014	0.01210	0.00005	0.000224	0.000016	0.000702	0.000012	0.000020	0.000001	0.0002702	0.0000004	263.00	2.32
01AK	1.38	57.576	2.222	0.0083	0.0001	37.644	49.196	1.8768	0.0736	0.26217	0.00052	0.001701	0.000064	87231.79	113.66	23651.66	30.37	332.44	2.90	7495.91	59.45	467.34	4.97	0.017029	0.000010	5.464E-10	28.201	FC-2	sanidine	0.00054	0.00014	0.01210	0.00005	0.000224	0.000016	0.000702	0.000012	0.000020	0.000001	0.0002702	0.0000004	263.00	2.32
01AL	1.55	56.911	2.325	0.0085	0.0001	42.980	47.948	1.8547	0.0769	0.25855	0.00052	0.001743	0.000066	85824.10	118.14	22938.52	28.30	329.73	3.17	7173.86	56.71	455.01	5.12	0.017029	0.000010	5.464E-10	28.201	FC-2	sanidine	0.00054	0.00014	0.01210	0.00005	0.000224	0.000016	0.000702	0.000012	0.000020	0.000001	0.0002702	0.0000004	263.00	2.32
01AM	1.85	57.077	2.243	0.0079	0.0001	49.453	45.014	1.8602	0.0742	0.24201	0.00045	0.001842	0.000060	111240.62	133.04	27901.86	32.46	410.71	3.44	9343.88	69.60	602.94	5.92	0.017029	0.000010	5.464E-10	28.201	FC-2	sanidine	0.00054	0.00014	0.01210	0.00005	0.000224	0.000016	0.000702	0.000012	0.000020	0.000001	0.0002702	0.0000004	263.00	2.32
01AN	2.3	53.854	2.936	0.0050	0.0000	59.050	32.609	1.7536	0.0970	0.18597	0.00032	0.002257	0.000060	214603.44	195.05	42229.53	37.00	677.93	4.59	21677.64	162.81	1408.56	10.76	0.017029	0.000010	5.464E-10	28.201	FC-2	sanidine	0.00054	0.00014	0.01210	0.00005	0.000224	0.000016	0.000702	0.000012	0.000020	0.000001	0.0002702	0.0000004	263.00	2.32
01AO	2.9	52.367	3.373	0.0029	0.0000	70.558	24.914	1.7045	0.1114	0.14618	0.00033	0.002515	0.000054	327378.92	286.84	52765.44	45.11	960.42	4.25	45201.97	244.65	2751.34	14.01	0.017029	0.000010	5.464E-10	28.201	FC-2	sanidine	0.00054	0.00014	0.01210	0.00005	0.000224	0.000016	0.000702	0.000012	0.000020	0.000001	0.0002702	0.0000004	263.00	2.32
01AP	3.6	58.401	5.059	0.0022	0.0000	73.865	22.325	1.9041	0.1676	0.11726	0.00038	0.002602	0.000065	117296.99	120.55	15614.77	24.62	315.48	2.86	17045.74	92.18	1032.68	6.49	0.017029	0.000010	5.464E-10	28.201	FC-2	sanidine	0.00054	0.00014	0.01210	0.00005	0.000224	0.000016	0.000702	0.000012	0.000020	0.000001	0.0002702	0.0000004	263.00	2.32
01AQ	4.6	58.656	6.131	0.0020	0.0000	77.405	22.590	1.9125	0.2031	0.11812	0.00039	0.002593	0.000080	124605.17	119.69	16934.60	22.85	344.12	3.09	20260.35	109.91	1188.18	8.69	0.017029	0.000010	5.464E-10	28.201	FC-2	sanidine	0.00054	0.00014	0.01210	0.00005	0.000224	0.000016	0.000702	0.000012	0.000020	0.000001	0.0002702	0.0000004	263.00	2.32
01AR	6.5	46.423	6.384	0.0017	0.0000	83.234	16.107	1.5086	0.2101	0.10678	0.00038	0.002810	0.000075	227015.50	219.27	28492.30	27.71	608.21	3.62	38787.32	206.54	2295.03	14.28	0.017029	0.000010	5.464E-10	28.201	FC-2	sanidine	0.00054	0.00014	0.01210	0.00005	0.000224	0.000016	0.000702	0.000012	0.000020	0.000001	0.0002702	0.0000004	263.00	2.32
01AS	9	50.681	6.427	0.0016	0.0000	89.115	17.738	1.6489	0.2120	0.10758	0.00041	0.002755	0.000076	227337.34	228.06	29127.91	31.25	615.86	3.69	42553.71	226.46	2445.20	14.02	0.017029	0.000010	5.464E-10	28.201	FC-2	sanidine	0.00054	0.00014	0.01210	0.00005	0.000224	0.000016	0.000702	0.000012	0.000020	0.000001	0.0002702	0.0000004	263.00	2.32
01AT	12	47.901	7.268	0.0014	0.0000	92.778	15.721	1.5572	0.2394	0.10096	0.00044	0.002823	0.000081	150875.47	151.39	18544.96	26.12	407.39	3.44	30126.77	140.86	1714.24	10.38	0.017029	0.000010	5.464E-10	28.201	FC-2	sanidine	0.00054	0.00014	0.01210	0.00005	0.000224	0.000016	0.000702	0.000012	0.000020	0.000001	0.0002702	0.0000004	263.00	2.32
01AU	16	49.435	7.121	0.0013	0.0000	98.014	14.792	1.6078	0.2347	0.09201	0.00040	0.002854	0.000072	236626.56	211.56	26699.78	26.57	614.62	4.04	44791.85	206.61	2591.54	14.26	0.017029	0.000010	5.464E-10	28.201	FC-2	sanidine	0.00054	0.00014	0.01210	0.00005	0.000224	0.000016	0.000702	0.000012	0.000020	0.000001	0.0002702	0.0000004	263.00	2.32
01AV	21	46.190	9.129	0.0013	0.0000	99.927	13.169	1.5009	0.3004	0.08774	0.00043	0.002908	0.000088	90692.04	113.49	9789.40	19.15	232.76	2.71	16635.00	75.30	975.83	7.22	0.017029	0.000010	5.464E-10	28.201	FC-2	sanidine	0.00054	0.00014	0.01210	0.00005	0.000224	0.000016	0.000702	0.000012	0.000020	0.000001	0.0002702	0.0000004	263.00	2.32
01AW	27	101.827	54.166	0.0011	0.0000	100.000	1.934	3.3598	1.8374	0.00576	0.00009	0.003285	0.000035	52560.38	77.54	383.55	4.35	41.23	1.31	734.33	6.43	204.09	1.81	0.017029	0.000010	5.464E-10	28.201	FC-2	sanidine	0.00054	0.00014	0.01210	0.00005	0.000224	0.000016	0.000702	0.000012	0.000020	0.000001	0.0002702	0.0000004	263.00	2.32

Integrated K/Ca ±2σ **0.0030** **0.0000**
Plateau Age ±2σ **54.961** **1.555** **n=17/25** **MSWD=0.758**
Total Integrated Age ±2σ **51.009** **1.993**
Isochron Age ±2σ **55.306** **2.240** **MSWD=0.803**
$(^{40}Ar/^{36}Ar)_{trapped}$ ±2σ **297.443** **5.206**

Sample: **SO249 DR51-9** **Identifier:** **UW145:B25**
Material: **Groundmass**

N	Power %	Age (Ma)	±1σ (Ma)	K/Ca	±1σ	Cum. %^{39}Ar (%)	%^{40}Ar (%)	$^{40}Ar^*/^{39}Ar_K$	±1σ	Corrected[1] $^{39}Ar/^{40}Ar$	±1σ	$^{36}Ar/^{40}Ar$	±1σ	^{40}Ar (cps)	±1σ	^{39}Ar (cps)	±1σ	^{38}Ar (cps)	±1σ	^{37}Ar (cps)	±1σ	^{36}Ar (cps)	±1σ	J	±1σ	LambdaK	MonitorAge	onitorNar	nitorMate	$(^{40}Ar/^{39}39)_K$	±1σ	$(^{38}Ar/^{39}39)_K$	±1σ	$(^{37}Ar/^{39}39)_K$	±1σ	$(^{39}Ar/^{37}37)_{Ca}$	±1σ	$(^{38}Ar/^{37}37)_{Ca}$	±1σ	$(^{36}Ar/^{37}37)_{Ca}$	±1σ	$(^{36}Ar/^{38}38)_{Cl}$	Ca/K
X 01A	0.25	6.686	38.207	0.0347	0.0011	0.397	0.224	0.2149	1.2304	0.01040	0.00007	0.003342	0.000043	92904.02	97.28	973.48	6.14	78.62	1.94	191.66	5.84	313.66	3.96	0.017029	0.000010	5.464E-10	28.201	FC-2	sanidine	0.00054	0.00014	0.01210	0.00005	0.000224	0.000016	0.000695	0.000009	0.000020	0.000001	0.0002650	0.0000022	263.00	2.32
X 01B	0.27	23.682	32.043	0.0291	0.0006	0.887	0.856	0.7648	1.0415	0.01120	0.00007	0.003321	0.000039	106501.86	114.19	1202.83	7.00	87.43	1.77	282.07	5.62	358.35	4.12	0.017029	0.000010	5.464E-10	28.201	FC-2	sanidine	0.00054	0.00014	0.01210	0.00005	0.000224	0.000016	0.000695	0.000009	0.000020	0.000001	0.0002650	0.0000022	263.00	2.32
X 01C	0.31	-56.837	21.271	0.0298	0.0004	1.888	-2.761	-1.7957	0.6616	0.01538	0.00006	0.003442	0.000034	158349.83	136.07	2455.87	9.85	137.36	2.35	561.60	6.63	554.36	5.34	0.017029	0.000010	5.464E-10	28.201	FC-2	sanidine	0.00054	0.00014	0.01210	0.00005	0.000224	0.000016	0.000695	0.000009	0.000020	0.000001	0.0002650	0.0000022	263.00	2.32
X 01D	0.38	-31.613	12.499	0.0252	0.0002	3.949	-2.135	-1.0056	0.3942	0.02123	0.00006	0.003421	0.000028	236271.18	202.41	5067.64	13.58	221.37	2.81	1367.44	11.58	831.01	6.53	0.017029	0.000010	5.464E-10	28.201	FC-2	sanidine	0.00054	0.00014	0.01210	0.00005	0.000224	0.000016	0.000695	0.000009	0.000020	0.000001	0.0002650	0.0000022	263.00	2.32
X 01E	0.46	14.120	8.078	0.0173	0.0001	7.490	1.402	0.4548	0.2612	0.03082	0.00007	0.003302	0.000027	279461.51	244.19	8750.34	18.98	293.86	3.13	3419.66	23.15	979.82	7.40	0.017029	0.000010	5.464E-10	28.201	FC-2	sanidine	0.00054	0.00014	0.01210	0.00005	0.000224	0.000016	0.000695	0.000009	0.000020	0.000001	0.0002650	0.0000022	263.00	2.32
X 01F	0.55	30.997	5.631	0.0113	0.0001	11.755	4.759	1.0031	0.1838	0.04745	0.00010	0.003190	0.000029	218749.91	201.01	10637.49	18.59	270.65	3.09	6289.02	40.22	802.56	6.21	0.017029	0.000010	5.464E-10	28.201	FC-2	sanidine	0.00054	0.00014	0.01210	0.00005	0.000224	0.000016	0.000695	0.000009	0.000020	0.000001	0.0002650	0.0000022	263.00	2.32
X 01G	0.69	34.118	4.020	0.0086	0.0001	18.885	7.758	1.1050	0.1314	0.07022	0.00013	0.003090	0.000031	247072.93	229.33	17926.89	26.96	385.11	3.69	13858.50	82.46	994.28	7.17	0.017029	0.000010	5.464E-10	28.201	FC-2	sanidine	0.00054	0.00014	0.01210	0.00005	0.000224	0.000016	0.000695	0.000009	0.000020	0.000001	0.0002650	0.0000022	263.00	2.32
01H	0.85	49.049	3.730	0.0073	0.0000	27.595	14.178	1.5951	0.1229	0.08889	0.00016	0.002875	0.000036	238407.62	201.23	22024.92	32.23	429.90	4.04	19810.66	119.50	1015.69	7.97	0.017029	0.000010	5.464E-10	28.201	FC-2	sanidine	0.00054	0.00014	0.01210	0.00005	0.000224	0.000016	0.000695	0.000009	0.000020	0.000001	0.0002650	0.0000022	263.00	2.32
01I	1.05	53.876	3.096	0.0065	0.0000	38.628	15.373	1.7544	0.1023	0.08763	0.00015	0.002834	0.000030	306354.74	278.53	28037.50	33.95	594.73	5.22	28189.19	167.44	1338.76	7.72	0.017029	0.000010	5.464E-10	28.201	FC-2	sanidine	0.00054	0.00014	0.01210	0.00005	0.000224	0.000016	0.000695	0.000009	0.000020	0.000001	0.0002650	0.0000022	263.00	2.32
01J	1.3	56.852	3.724	0.0062	0.0000	48.957	16.665	1.8528	0.1232	0.08995	0.00015	0.002791	0.000037	279410.07	241.71	26299.68	32.85	583.33	4.34	27547.06	163.60	1239.92	9.16	0.017029	0.000010	5.464E-10	28.201	FC-2	sanidine	0.00054	0.00014	0.01210	0.00005	0.000224	0.000016	0.000695	0.000009	0.000020	0.000001	0.0002650	0.0000022	263.00	2.32
01K	1.6	53.040	3.642	0.0060	0.0000	57.132	15.881	1.7267	0.1203	0.09198	0.00019	0.002817	0.000037	216265.11	203.33	20859.47	33.74	492.63	4.49	22815.71	135.50	990.57	6.93	0.017029	0.000010	5.464E-10	28.201	FC-2	sanidine	0.00054	0.00014	0.01210	0.00005	0.000224	0.000016	0.000695	0.000009	0.000020	0.000001	0.0002650	0.0000022	263.00	2.32

01L	2	56.072	4.626	0.0042	0.0000	66.157	13.164	1.8270	0.1530	0.07206	0.00014	0.002908	0.000037	304760.79	262.42	23491.08	30.70	611.05	4.32	35735.40	208.51	1483.96	9.37	0.017029	0.000010	5.464E-10	28.201	FC-2	sanidine	0.00054	0.00014	0.01210	0.00005	0.000224	0.000016	0.000695	0.000009	0.000020	0.000001	0.0002650	0.0000022	263.00	2.32
01M	2.5	51.362	7.479	0.0020	0.0000	75.538	9.194	1.6714	0.2468	0.05501	0.00015	0.003041	0.000045	414890.52	353.45	26207.38	34.77	745.81	5.19	78016.89	451.75	2567.05	13.28	0.017029	0.000010	5.464E-10	28.201	FC-2	sanidine	0.00054	0.00014	0.01210	0.00005	0.000224	0.000016	0.000695	0.000009	0.000020	0.000001	0.0002650	0.0000022	263.00	2.32
01N	2.9	61.888	12.237	0.0013	0.0000	79.708	11.300	2.0197	0.4061	0.05595	0.00023	0.002971	0.000076	181335.09	182.99	12477.95	23.65	315.36	3.23	53561.56	312.51	1435.17	10.29	0.017029	0.000010	5.464E-10	28.201	FC-2	sanidine	0.00054	0.00014	0.01210	0.00005	0.000224	0.000016	0.000695	0.000009	0.000020	0.000001	0.0002650	0.0000022	263.00	2.32
01O	3.3	54.365	12.473	0.0012	0.0000	82.632	10.255	1.7705	0.4123	0.05792	0.00027	0.003006	0.000080	122832.06	114.50	8853.92	19.97	218.66	2.34	39906.23	233.28	1037.47	7.05	0.017029	0.000010	5.464E-10	28.201	FC-2	sanidine	0.00054	0.00014	0.01210	0.00005	0.000224	0.000016	0.000695	0.000009	0.000020	0.000001	0.0002650	0.0000022	263.00	2.32
01P	3.7	67.309	12.218	0.0013	0.0000	87.055	13.084	2.1999	0.4067	0.05948	0.00024	0.002911	0.000081	180955.91	168.00	13285.84	22.21	319.71	3.03	57877.17	335.96	1496.33	10.79	0.017029	0.000010	5.464E-10	28.201	FC-2	sanidine	0.00054	0.00014	0.01210	0.00005	0.000224	0.000016	0.000695	0.000009	0.000020	0.000001	0.0002650	0.0000022	263.00	2.32
01Q	4.2	62.342	11.262	0.0012	0.0000	93.004	11.732	2.0348	0.3739	0.05766	0.00023	0.002956	0.000072	251023.15	228.92	18014.73	27.16	437.84	3.80	81175.25	469.45	2102.47	11.64	0.017029	0.000010	5.464E-10	28.201	FC-2	sanidine	0.00054	0.00014	0.01210	0.00005	0.000224	0.000016	0.000695	0.000009	0.000020	0.000001	0.0002650	0.0000022	263.00	2.32
01R	4.8	66.803	15.459	0.0011	0.0000	94.471	12.996	2.1831	0.5145	0.05953	0.00034	0.002914	0.000102	59988.68	71.33	4516.88	14.79	111.16	2.22	21659.93	130.67	537.99	4.87	0.017029	0.000010	5.464E-10	28.201	FC-2	sanidine	0.00054	0.00014	0.01210	0.00005	0.000224	0.000016	0.000695	0.000009	0.000020	0.000001	0.0002650	0.0000022	263.00	2.32
01S	5.5	68.704	17.505	0.0012	0.0000	96.541	13.522	2.2463	0.5831	0.06020	0.00030	0.002896	0.000117	83654.84	90.38	6311.30	16.64	153.07	2.56	29212.15	173.85	732.27	8.44	0.017029	0.000010	5.464E-10	28.201	FC-2	sanidine	0.00054	0.00014	0.01210	0.00005	0.000224	0.000016	0.000695	0.000009	0.000020	0.000001	0.0002650	0.0000022	263.00	2.32
01T	6.5	33.467	21.614	0.0014	0.0000	97.047	6.495	1.0837	0.7063	0.05994	0.00044	0.003132	0.000142	20530.85	47.37	1493.94	7.75	36.39	1.49	6035.47	39.28	165.58	2.70	0.017029	0.000010	5.464E-10	28.201	FC-2	sanidine	0.00054	0.00014	0.01210	0.00005	0.000224	0.000016	0.000695	0.000009	0.000020	0.000001	0.0002650	0.0000022	263.00	2.32
01U	8	46.206	12.317	0.0013	0.0000	99.324	9.798	1.5015	0.4053	0.06526	0.00029	0.003021	0.000088	84921.01	87.68	6773.69	16.39	155.97	2.38	25091.91	140.29	730.37	5.81	0.017029	0.000010	5.464E-10	28.201	FC-2	sanidine	0.00054	0.00014	0.01210	0.00005	0.000224	0.000016	0.000695	0.000009	0.000020	0.000001	0.0002650	0.0000022	263.00	2.32
01V	10	63.147	19.420	0.0013	0.0000	100.000	13.265	2.0615	0.6450	0.06435	0.00044	0.002905	0.000139	25545.86	50.18	2008.84	9.26	44.72	1.18	7429.32	45.50	214.57	3.23	0.017029	0.000010	5.464E-10	28.201	FC-2	sanidine	0.00054	0.00014	0.01210	0.00005	0.000224	0.000016	0.000695	0.000009	0.000020	0.000001	0.0002650	0.0000022	263.00	2.32

**Integrated K/Ca ±2σ**			**0.0025**	**0.0000**
**Plateau Age ±2σ**	**54.125**	**3.033**	**n=15/22**	**MSWD=0.558**
**Total Integrated Age ±2σ**	**48.493**	**3.428**		
**Isochron Age ±2σ**	**47.384**	**13.849**		**MSWD=0.540**
**$(^{40}Ar/^{36}Ar)_{trapped}$ ±2σ**	**304.813**	**14.062**		

Sample: SO249 DR51-9 **Identifier: UW145:B25**
Material: Groundmass

N	Power %	Age (Ma)	±1σ (Ma)	K/Ca	±1σ	Cum. $\%^{39}Ar$ (%)	$\%^{40}Ar$ (%)	$^{40}Ar^*/^{39}Ar_K$	±1σ	Corrected[1] $^{39}Ar/^{40}Ar$	±1σ	$^{36}Ar/^{40}Ar$	±1σ	^{40}Ar (cps)	±1σ	^{39}Ar (cps)	±1σ	^{38}Ar (cps)	±1σ	^{37}Ar (cps)	±1σ	^{36}Ar (cps)	±1σ	J	±1σ	LambdaK	MonitorAge	onitorNar	nitorMate	$(^{40}Ar/^{39}39)_K$	±1σ	$(^{38}Ar/^{39}39)_K$	±1σ	$(^{37}Ar/^{39}39)_K$	±1σ	$(^{39}Ar/^{37}37)_{Ca}$	±1σ	$(^{38}Ar/^{37}37)_{Ca}$	±1σ	$(^{36}Ar/^{37}37)_{Ca}$	±1σ	$(^{36}Ar/^{38}38)_{Cl}$	Ca/K
X 02A	0.39	24.339	19.112	0.0211	0.0003	0.593	1.559	0.7862	0.6214	0.01983	0.00008	0.003297	0.000041	103975.90	111.43	2087.52	8.39	99.98	1.55	275.35	4.21	353.98	4.26	0.017029	0.000010	5.464E-10	28.201	FC-2	sanidine	0.00054	0.00014	0.01210	0.00005	0.000224	0.000016	0.000695	0.000009	0.000020	0.000001	0.0002650	0.0000022	263.00	2.32
X 02B	0.42	26.077	13.817	0.0185	0.0002	1.454	2.044	0.8427	0.4497	0.02426	0.00008	0.003281	0.000036	123458.61	117.70	3037.87	9.36	118.69	1.95	456.22	5.13	423.54	4.47	0.017029	0.000010	5.464E-10	28.201	FC-2	sanidine	0.00054	0.00014	0.01210	0.00005	0.000224	0.000016	0.000695	0.000009	0.000020	0.000001	0.0002650	0.0000022	263.00	2.32
X 02C	0.45	31.243	10.658	0.0152	0.0001	2.737	3.254	1.0111	0.3479	0.03219	0.00010	0.003240	0.000037	138594.74	134.30	4540.71	12.43	147.60	2.16	826.45	7.03	482.60	5.14	0.017029	0.000010	5.464E-10	28.201	FC-2	sanidine	0.00054	0.00014	0.01210	0.00005	0.000224	0.000016	0.000695	0.000009	0.000020	0.000001	0.0002650	0.0000022	263.00	2.32
X 02D	0.5	15.932	9.176	0.0120	0.0001	4.012	2.157	0.5134	0.2970	0.04202	0.00012	0.003277	0.000042	105549.53	105.97	4537.13	11.62	126.10	1.99	1040.14	8.45	388.09	4.35	0.017029	0.000010	5.464E-10	28.201	FC-2	sanidine	0.00054	0.00014	0.01210	0.00005	0.000224	0.000016	0.000695	0.000009	0.000020	0.000001	0.0002650	0.0000022	263.00	2.32
X 02E	0.56	20.775	7.530	0.0105	0.0001	5.729	3.550	0.6704	0.2444	0.05295	0.00013	0.003231	0.000043	112782.20	119.17	6130.89	13.03	147.27	2.15	1599.09	11.47	429.25	4.80	0.017029	0.000010	5.464E-10	28.201	FC-2	sanidine	0.00054	0.00014	0.01210	0.00005	0.000224	0.000016	0.000695	0.000009	0.000020	0.000001	0.0002650	0.0000022	263.00	2.32
X 02F	0.63	30.441	5.475	0.0091	0.0001	8.471	6.503	0.9849	0.1786	0.06603	0.00015	0.003132	0.000039	144423.26	139.47	9831.54	18.77	219.96	2.65	2940.89	18.35	571.72	5.53	0.017029	0.000010	5.464E-10	28.201	FC-2	sanidine	0.00054	0.00014	0.01210	0.00005	0.000224	0.000016	0.000695	0.000009	0.000020	0.000001	0.0002650	0.0000022	263.00	2.32
X 02G	0.71	36.212	4.535	0.0081	0.0001	11.913	9.729	1.1735	0.1484	0.08291	0.00017	0.003024	0.000041	144395.75	146.64	12394.79	19.57	244.40	2.48	4178.07	25.83	606.40	5.64	0.017029	0.000010	5.464E-10	28.201	FC-2	sanidine	0.00054	0.00014	0.01210	0.00005	0.000224	0.000016	0.000695	0.000009	0.000020	0.000001	0.0002650	0.0000022	263.00	2.32
02H	0.8	52.857	5.067	0.0075	0.0000	15.098	17.335	1.7207	0.1673	0.10075	0.00021	0.002769	0.000056	109939.25	114.27	11495.93	19.07	212.59	2.31	4137.82	25.19	472.67	5.94	0.017029	0.000010	5.464E-10	28.201	FC-2	sanidine	0.00054	0.00014	0.01210	0.00005	0.000224	0.000016	0.000695	0.000009	0.000020	0.000001	0.0002650	0.0000022	263.00	2.32
02I	0.9	58.626	3.341	0.0072	0.0000	20.770	21.076	1.9115	0.1107	0.11027	0.00019	0.002643	0.000041	178922.07	174.10	20511.36	25.56	381.37	3.42	7687.90	44.23	785.86	6.54	0.017029	0.000010	5.464E-10	28.201	FC-2	sanidine	0.00054	0.00014	0.01210	0.00005	0.000224	0.000016	0.000695	0.000009	0.000020	0.000001	0.0002650	0.0000022	263.00	2.32
02J	1.05	54.459	3.293	0.0070	0.0000	26.493	20.907	1.7736	0.1089	0.11788	0.00021	0.002649	0.000043	168859.65	157.10	20714.93	26.64	377.90	3.17	7937.36	45.09	770.56	6.44	0.017029	0.000010	5.464E-10	28.201	FC-2	sanidine	0.00054	0.00014	0.01210	0.00005	0.000224	0.000016	0.000695	0.000009	0.000020	0.000001	0.0002650	0.0000022	263.00	2.32
02K	1.2	56.706	2.825	0.0070	0.0000	32.704	25.192	1.8480	0.0935	0.13633	0.00023	0.002506	0.000042	158455.75	149.78	22485.82	26.42	409.91	3.35	8659.30	48.86	749.92	5.71	0.017029	0.000010	5.464E-10	28.201	FC-2	sanidine	0.00054	0.00014	0.01210	0.00005	0.000224	0.000016	0.000695	0.000009	0.000020	0.000001	0.0002650	0.0000022	263.00	2.32
02L	1.4	58.298	2.992	0.0068	0.0000	39.680	27.323	1.9007	0.0991	0.14376	0.00023	0.002434	0.000048	168766.31	158.99	25280.51	28.11	481.99	3.86	9964.68	55.81	817.15	6.90	0.017029	0.000010	5.464E-10	28.201	FC-2	sanidine	0.00054	0.00014	0.01210	0.00005	0.000224	0.000016	0.000695	0.000009	0.000020	0.000001	0.0002650	0.0000022	263.00	2.32
02M	1.6	57.812	3.130	0.0065	0.0000	46.303	26.217	1.8846	0.1037	0.13913	0.00023	0.002471	0.000048	165577.94	161.33	24054.99	26.17	490.10	3.90	9942.92	57.31	814.94	6.81	0.017029	0.000010	5.464E-10	28.201	FC-2	sanidine	0.00054	0.00014	0.01210	0.00005	0.000224	0.000016	0.000695	0.000009	0.000020	0.000001	0.0002650	0.0000022	263.00	2.32
02N	1.85	57.199	3.526	0.0058	0.0000	52.104	24.416	1.8643	0.1167	0.13097	0.00024	0.002532	0.000051	154054.42	148.63	21175.82	26.80	436.03	3.30	9697.39	56.18	785.97	6.75	0.017029	0.000010	5.464E-10	28.201	FC-2	sanidine	0.00054	0.00014	0.01210	0.00005	0.000224	0.000016	0.000695	0.000009	0.000020	0.000001	0.0002650	0.0000022	263.00	2.32
02O	2.1	57.994	3.754	0.0048	0.0000	57.462	22.437	1.8906	0.1243	0.11868	0.00023	0.002598	0.000049	157029.63	156.85	19763.30	26.10	420.27	3.14	10856.87	63.18	851.58	6.27	0.017029	0.000010	5.464E-10	28.201	FC-2	sanidine	0.00054	0.00014	0.01210	0.00005	0.000224	0.000016	0.000695	0.000009	0.000020	0.000001	0.0002650	0.0000022	263.00	2.32
02P	2.4	55.153	4.698	0.0032	0.0000	65.738	17.673	1.7966	0.1553	0.09838	0.00019	0.002757	0.000051	292606.78	261.57	31380.85	33.97	726.32	4.32	24748.70	141.02	1818.65	10.86	0.017029	0.000010	5.464E-10	28.201	FC-2	sanidine	0.00054	0.00014	0.01210	0.00005	0.000224	0.000016	0.000695	0.000009	0.000020	0.000001	0.0002650	0.0000022	263.00	2.32
02Q	2.7	56.882	6.491	0.0027	0.0000	70.528	19.320	1.8538	0.2149	0.10423	0.00024	0.002702	0.000075	159828.82	144.76	18488.31	23.06	398.15	3.13	17378.01	95.08	1142.82	9.63	0.017029	0.000010	5.464E-10	28.201	FC-2	sanidine	0.00054	0.00014	0.01210	0.00005	0.000224	0.000016	0.000695	0.000009	0.000020	0.000001	0.0002650	0.0000022	263.00	2.32
02R	3.05	49.934	5.907	0.0030	0.0000	72.964	17.332	1.6243	0.1948	0.10672	0.00029	0.002769	0.000069	79384.50	92.32	9307.70	16.33	203.69	2.40	7950.42	47.24	545.22	4.39	0.017029	0.000010	5.464E-10	28.201	FC-2	sanidine	0.00054	0.00014	0.01210	0.00005	0.000224	0.000016	0.000695	0.000009	0.000020	0.000001	0.0002650	0.0000022	263.00	2.32
02S	3.4	58.358	7.014	0.0026	0.0000	76.077	17.938	1.9027	0.2324	0.09429	0.00025	0.002749	0.000073	114845.10	117.03	12038.77	20.79	278.18	2.32	11476.89	63.15	785.73	6.97	0.017029	0.000010	5.464E-10	28.201	FC-2	sanidine	0.00054	0.00014	0.01210	0.00005	0.000224	0.000016	0.000695	0.000009	0.000020	0.000001	0.0002650	0.0000022	263.00	2.32
02T	3.8	58.974	12.392	0.0021	0.0000	77.530	17.783	1.9231	0.4106	0.09248	0.00035	0.002754	0.000127	54663.00	79.75	5780.33	14.22	127.65	1.86	6848.91	38.97	431.19	6.34	0.017029	0.000010	5.464E-10	28.201	FC-2	sanidine	0.00054	0.00014	0.01210	0.00005	0.000224	0.000016	0.000695	0.000009	0.000020	0.000001	0.0002650	0.0000022	263.00	2.32
02U	4.3	63.376	8.309	0.0018	0.0000	80.610	17.599	2.0691	0.2760	0.08506	0.00027	0.002760	0.000078	125937.77	124.31	12464.96	21.37	289.42	2.66	16509.37	91.12	1024.55	7.19	0.017029	0.000010	5.464E-10	28.201	FC-2	sanidine	0.00054	0.00014	0.01210	0.00005	0.000224	0.000016	0.000695	0.000009	0.000020	0.000001	0.0002650	0.0000022	263.00	2.32
02V	4.9	49.305	9.347	0.0016	0.0000	83.196	14.258	1.6035	0.3081	0.08892	0.00032	0.002872	0.000091	101136.51	109.07	10642.54	19.65	236.20	2.75	15510.66	85.61	926.74	6.73	0.017029	0.000010	5.464E-10	28.201	FC-2	sanidine	0.00054	0.00014	0.01210	0.00005	0.000224	0.000016	0.000695	0.000009	0.000020	0.000001	0.0002650	0.0000022	263.00	2.32
02W	5.6	54.973	10.407	0.0015	0.0000	84.826	15.558	1.7906	0.3441	0.08689	0.00034	0.002828	0.000100	65232.52	83.74	6803.09	13.52	147.37	1.74	10660.56	58.95	622.08	4.83	0.017029	0.000010	5.464E-10	28.201	FC-2	sanidine	0.00054	0.00014	0.01210	0.00005	0.000224	0.000016	0.000695	0.000009	0.000020	0.000001	0.0002650	0.0000022	263.00	2.32
02X	7.5	47.145	14.422	0.0012	0.0000	85.986	12.518	1.5324	0.4748	0.08169	0.00041	0.002930	0.000130	49377.56	72.41	5072.82	12.17	109.46	1.70	9727.72	55.07	544.39	4.98	0.017029	0.000010	5.464E-10	28.201	FC-2	sanidine	0.00054	0.00014	0.01210	0.00005	0.000224	0.000016	0.000695	0.000009	0.000020	0.000001	0.0002650	0.0000022	263.00	2.32
02Y	11	64.406	13.172	0.0012	0.0000	94.603	16.939	2.1033	0.4378	0.08054	0.00031	0.002782	0.000118	372141.81	297.64	37539.37	35.87	822.95	4.45	70812.55	397.43	3947.61	32.66	0.017029	0.000010	5.464E-10	28.201	FC-2	sanidine	0.00054	0.00014	0.01210	0.00005	0.000224	0.000016	0.000695	0.000009	0.000020	0.000001	0.0002650	0.0000022	263.00	2.32
02Z	13	52.020	13.398	0.0012	0.0000	99.408	13.949	1.6931	0.4423	0.08239	0.00032	0.002882	0.000122	202851.77	174.29	20763.31	24.14	464.14	3.49	37889.82	211.62	2143.64	19.14	0.017029	0.000010	5.464E-10	28.201	FC-2	sanidine	0.00054	0.00014	0.01210	0.00005	0.000224	0.000016	0.000695	0.000009	0.000020	0.000001	0.0002650	0.0000022	263.00	2.32
02AA	17	34.795	25.136	0.0012	0.0000	100.000	8.174	1.1271	0.8220	0.07252	0.00044	0.003076	0.000200	28394.53	53.10	2578.08	9.19	65.11	1.27	4847.29	29.17	286.97	5.28	0.017029	0.000010	5.464E-10	28.201	FC-2	sanidine	0.00054	0.00014	0.01210	0.00005	0.000224	0.000016	0.000695	0.000009	0.000020	0.000001	0.0002650	0.0000022	263.00	2.32

**Integrated K/Ca ±2σ**			**0.0028**	**0.0000**
**Plateau Age ±2σ**	**56.659**	**2.122**	**n=20/27**	**MSWD=*0.336**
**Total Integrated Age ±2σ**	**53.512**	**3.278**		
**Isochron Age ±2σ**	**60.536**	**9.631**		**MSWD=*0.324**
**$(^{40}Ar/^{36}Ar)_{trapped}$ ±2σ**	**292.728**	**14.639**		

Sample: SO249-DR51-9 **Identifier: UW150:B46**
Material: Groundmass

N	Power %	Age (Ma)	±1σ (Ma)	K/Ca	±1σ	Cum. $\%^{39}Ar$ (%)	$\%^{40}Ar$ (%)	$^{40}Ar^*/^{39}Ar_K$	±1σ	Corrected[1] $^{39}Ar/^{40}Ar$	±1σ	$^{36}Ar/^{40}Ar$	±1σ	^{40}Ar (cps)	±1σ	^{39}Ar (cps)	±1σ	^{38}Ar (cps)	±1σ	^{37}Ar (cps)	±1σ	^{36}Ar (cps)	±1σ	J	±1σ	LambdaK	MonitorAge	onitorNar	nitorMate	$(^{40}Ar/^{39}39)_K$	±1σ	$(^{38}Ar/^{39}39)_K$	±1σ	$(^{37}Ar/^{39}39)_K$	±1σ	$(^{39}Ar/^{37}37)_{Ca}$	±1σ	$(^{38}Ar/^{37}37)_{Ca}$	±1σ	$(^{36}Ar/^{37}37)_{Ca}$	±1σ	$(^{36}Ar/^{38}38)_{Cl}$	Ca/K
X 01A	0.35	-64.433	32.695	0.0391	0.0004	0.341	-2.256	-1.5705	0.7829	0.01437	0.00006	0.003425	0.000038	68369.41	51.85	988.98	4.36	74.51	1.07	1245.72	10.94	237.07	2.56	0.022028	0.000006	5.464E-10	28.201	FC-2	sanidine	0.00054	0.00014	0.01210	0.00002	0.000224	0.000016	0.000702	0.000012	0.000020	0.000001	0.0002702	0.0000004	263.00	2.32
X 01B	0.4	13.233	43.507	0.0346	0.0004	0.527	0.486	0.3294	1.0870	0.01474	0.00010	0.003333	0.000054	36221.14	37.24	538.20	3.51	34.65	0.99	764.71	8.27	122.52	1.94	0.022028	0.000006	5.464E-10	28.201	FC-2	sanidine	0.00054	0.00014	0.01210	0.00002	0.000224	0.000016	0.000702	0.000012	0.000020	0.000001	0.0002702	0.0000004	263.00	2.32
X 01C	0.5	-36.084	13.480	0.0301	0.0002	1.659	-2.084	-0.8863	0.3278	0.02352	0.00006	0.003419	0.000026	138443.37	93.69	3285.88	7.71	149.63	1.52	5353.99	37.82	485.91	3.53	0.022028	0.000006	5.464E-10	28.201	FC-2	sanidine	0.00054	0.00014	0.01210	0.00002	0.000224	0.000016	0.000702	0.000012	0.000020	0.000001	0.0002702	0.0000004	263.00	2.32
X 01D	0.6	-7.787	7.815	0.0199	0.0001	4.720	-0.653	-0.1927	0.1930	0.03390	0.00005	0.003371	0.000022	259835.26	151.64	8934.02	12.89	306.98	2.14	21847.39	145.27	927.29	5.59	0.022028	0.000006	5.464E-10	28.201	FC-2	sanidine	0.00054	0.00014	0.01210	0.00002	0.000224	0.000016	0.000702	0.000012	0.000020	0.000001	0.0002702	0.0000004	263.00	2.32
X 01E	0.72	13.739	4.535	0.0127	0.0001	11.557	1.742	0.3421	0.1133	0.05093	0.00006	0.003291	0.000019	386388.55	215.41	20124.79	18.16	540.46	2.56	76323.39	506.75	1451.17	7.21	0.022028	0.000006	5.464E-10	28.201	FC-2	sanidine	0.00054	0.00014	0.01210	0.00002	0.000224	0.000016	0.000702	0.000012	0.000020	0.000001	0.0002702	0.0000004	263.00	2.32
X 01F	0.85	35.094	4.136	0.0097	0.0001	19.970	5.404	0.8789	0.1046	0.06151	0.00007	0.003168	0.000021	393654.65	207.46	24938.67	20.65	598.71	2.96	122866.28	823.63	1536.65	8.10	0.022028	0.000006	5.464E-10	28.201	FC-2	sanidine	0.00054	0.00014	0.01210	0.00002	0.000224	0.000016	0.000702	0.000012	0.000020	0.000001	0.0002702	0.0000004	263.00	2.32
01G	1	51.507	4.209	0.0089	0.0001	29.061	9.534	1.2958	0.1074	0.07360	0.00009	0.003030	0.000026	355472.99	246.10	27025.64	22.22	605.96	3.05	145589.06	1331.15	1420.22	8.73	0.022028	0.000006	5.464E-10	28.201	FC-2	sanidine	0.00054	0.00014	0.01210	0.00002	0.000224	0.000016	0.000702	0.000012	0.000020	0.000001	0.0002702	0.0000004	263.00	2.32
01H	1.15	55.915	3.064	0.0092	0.0001	37.838	12.904	1.4084	0.0784	0.09167	0.00011	0.002917	0.000024	275607.69	154.97	26059.11	20.29	573.77	3.06	134828.71	918.44	1122.01	6.16	0.022028	0.000006	5.464E-10	28.201	FC-2	sanidine	0.00054	0.00014	0.01210	0.00002	0.000224	0.000016	0.000702	0.000012	0.000020	0.000001	0.0002702	0.0000004	263.00	2.32
01I	1.3	55.841	2.886	0.0090	0.0001	45.514	14.476	1.4065	0.0738	0.10298	0.00013	0.002864	0.000025	214581.32	123.19	22807.33	19.49	528.56	2.81	120342.20	819.71	898.67	5.02	0.022028	0.000006	5.464E-10	28.201	FC-2	sanidine	0.00054	0.00014	0.01210	0.00002	0.000224	0.000016	0.000702	0.000012	0.000020	0.000001	0.0002702	0.0000004	263.00	2.32
01J	1.5	54.916	3.717	0.0077	0.0001	51.826	11.817	1.3828	0.0950	0.08549	0.00011	0.002953	0.000027	212524.54	122.27	18857.34	17.42	475.21	2.48	115816.69	792.53	901.23	5.39	0.022028	0.000006	5.464E-10	28.201	FC-2	sanidine	0.00054	0.00014	0.01210	0.00002	0.000224	0.000016	0.000702	0.000012	0.000020	0.000001	0.0002702	0.0000004	263.00	2.32
01K	1.7	50.611	4.563	0.0060	0.0000	57.365	8.958	1.2729	0.1164	0.07040	0.00011	0.003049	0.000027	226425.90	130.78	16723.22	17.58	430.94	2.59	130358.58	910.45	998.64	5.74	0.022028	0.000006	5.464E-10	28.201	FC-2	sanidine	0.00054	0.00014	0.01210	0.00002	0.000224	0.000016	0.000702	0.000012	0.000020	0.000001	0.0002702	0.0000004	263.00	2.32
01L	1.95	57.531	4.917	0.0046	0.0000	63.861	9.637	1.4497	0.1259	0.06650	0.00011	0.003026	0.000028	281147.79	158.19	19897.14	18.52	511.00	2.81	198791.25	1387.54	1321.19	7.03	0.022028	0.000006	5.464E-10	28.201	FC-2	sanidine	0.00054	0.00014	0.01210	0.00002	0.000224	0.000016	0.000702	0.000012	0.000020	0.000001	0.0002702	0.0000004	263.00	2.32
01M	2.2	60.279	6.762	0.0032	0.0000	70.902	9.760	1.5201	0.1733	0.06423	0.00014	0.003022	0.000037	315478.14	234.56	22169.48	18.72	554.65	2.88	313805.65	2197.54	1696.29	10.38	0.022028	0.000006	5.464E-10	28.201	FC-2	sanidine	0.00054	0.00014	0.01210	0.00002	0.000224	0.000016	0.000702	0.000012	0.000020	0.000001	0.0002702	0.0000004	263.00	2.32
01N	2.45	59.322	9.010	0.0035	0.0000	72.752	10.050	1.4956	0.2309	0.06722	0.00019	0.003013	0.000052	79228.37	71.46	5786.34	11.05	136.65	1.56	75821.36	532.44	418.29	3.89	0.022028	0.000006	5.464E-10	28.201	FC-2	sanidine	0.00054	0.00014	0.01210	0.00002	0.000224	0.000016	0.000702	0.000012	0.000020	0.000001	0.0002702	0.0000004	263.00	2.32
01O	2.7	56.592	14.247	0.0034	0.0000	73.414	8.972	1.4257	0.3645	0.06295	0.00025	0.003049	0.000077	30262.14	38.42	2074.78	6.28	48.21	0.95	27910.92	197.14	158.40	2.27	0.022028	0.000006	5.464E-10	28.201	FC-2	sanidine	0.00054	0.00014	0.01210	0.00002	0.000224	0.000016	0.000702	0.000012	0.000020	0.000001	0.0002702	0.0000004	263.00	2.32
01P	3.2	60.188	10.221	0.0024	0.0000	75.129	8.421	1.5178	0.2620	0.05550	0.00018	0.003067	0.000048	88954.28	79.16	5544.28	10.37	146.59	1.54	99532.27	702.96	508.82	3.95	0.022028	0.000006	5.464E-10	28.201	FC-2	sanidine	0.00054	0.00014	0.01210	0.00002	0.000224	0.000016	0.000702	0.000012	0.000020	0.000001	0.0002702	0.0000004	263.00	2.32
01Q	3.8	59.815	9.231	0.0023	0.0000	78.175	8.835	1.5082	0.2366	0.05860	0.00018	0.003053	0.000046	149583.16	95.17	9910.33	13.02	259.21	2.12	187390.25	1781.63	901.33	5.39	0.022028	0.000006	5.464E-10	28.201	FC-2	sanidine	0.00054	0.00014	0.01210	0.00002	0.000224	0.000016	0.000702	0.000012	0.000020	0.000001	0.0002702	0.0000004	263.00	2.32
01R	4.6	64.801	9.208	0.0020	0.0000	83.225	9.654	1.6362	0.2366	0.05902	0.00019	0.003026	0.000046	246238.70	143.32	16769.54	16.50	431.51	2.55	365318.04	3471.87	1612.21	7.76	0.022028	0.000006	5.464E-10	28.201	FC-2	sanidine	0.00054	0.00014	0.01210	0.00002	0.000224	0.000016	0.000702	0.000012	0.000020	0.000001	0.0002702	0.0000004	263.00	2.32
01S	5.5	57.412	11.772	0.0016	0.0000	85.816	8.878	1.4467	0.3013	0.06139	0.00025	0.003052	0.000061	121492.01	83.42	8868.34	12.63	205.70	1.73	230018.91	2186.40	916.93	5.30	0.022028	0.000006	5.464E-10	28.201	FC-2	sanidine	0.00054	0.00014	0.01210	0.00002	0.000224	0.000016	0.000702	0.000012	0.000020	0.000001	0.0002702	0.0000004	263.00	2.32
01T	6.7	35.314	14.211	0.0013	0.0000	90.067	5.632	0.8845	0.3594	0.06370	0.00032	0.003161	0.000076	192048.83	152.98	15159.88	16.32	344.33	2.29	476689.29	4883.77	1739.68	8.74	0.022028	0.000006	5.464E-10	28.201	FC-2	sanidine	0.00054	0.00014	0.01210	0.00002	0.000224	0.000016	0.000702	0.000012	0.000020	0.000001	0.0002702	0.0000004	263.00	2.32
01U	8	29.332	15.715	0.0011	0.0000	95.055	4.900	0.7334	0.3961	0.06683	0.00038	0.003185	0.000088	214806.27	168.79	18280.81	18.20	410.60	2.48	638658.25	6553.23	2202.20	10.52	0.022028	0.000006	5.464E-10	28.201	FC-2	sanidine	0.00054	0.00014	0.01210	0.00002	0.000224	0.000016	0.000702	0.000012	0.000020	0.000001	0.0002702	0.0000004	263.00	2.32
01V	9.5	41.291	13.749	0.0013	0.0000	99.825	7.040	1.0359	0.3488	0.06799	0.00034	0.003114	0.000079	201942.60	157.08	17008.14	17.65	383.43	2.56	533515.97	5465.95	1897.19	8.99	0.022028	0.000006	5.464E-10	28.201	FC-2	sanidine	0.00054	0.00014	0.01210	0.00002	0.000224	0.000016	0.000702	0.000012	0.000020	0.000001	0.0002702	0.0000004	263.00	2.32
01W	11.5	121.796	43.501	0.0015	0.0000	100.000	19.087	3.1239	1.1533	0.06112	0.00054	0.002710	0.000236	8237.49	26.36	602.56	3.66	16.45	0.68	16135.45	159.83	60.70	1.90	0.022028	0.000006	5.464E-10	28.201	FC-2	sanidine	0.00054	0.00014	0.01210	0.00002	0.000224	0.000016	0.000702	0.000012	0.000020	0.000001	0.0002702	0.0000004	263.00	2.32

**Integrated K/Ca ±2σ**			**0.0030**	**0.0000**
**Plateau Age ±2σ**	**55.123**	**2.724**	**n=17/23**	**MSWD=0.784**
**Total Integrated Age ±2σ**	**45.519**	**3.402**		
**Isochron Age ±2σ**	**54.175**	**11.752**		**MSWD=0.842**
**$(^{40}Ar/^{36}Ar)_{trapped}$ ±2σ**	**299.195**	**8.741**		

Sample: SO249-DR51-6 **Identifier: UW164:A01**
Material: Plagioclase

N	Power %	Age (Ma)	±1σ (Ma)	K/Ca	±1σ	Cum. $\%^{39}Ar$ (%)	$\%^{40}Ar^*$ (%)	$^{40}Ar^*/^{39}Ar_K$	±1σ	$^{39}Ar/^{40}Ar$	±1σ	$^{36}Ar/^{40}Ar$	±1σ	Corrected[1] ^{40}Ar (Volts)	±1σ	^{39}Ar (Volts)	±1σ	^{38}Ar (Volts)	±1σ	^{37}Ar (Volts)	±1σ	^{36}Ar (Volts)	±1σ	J	±1σ	LambdaK	MonitorAge	onitorNar	nitorMate	$(^{40}Ar/^{39}39)_K$	±1σ	$(^{38}Ar/^{39}39)_K$	±1σ	$(^{37}Ar/^{39}39)_K$	±1σ	$(^{39}Ar/^{37}37)_{Ca}$	±1σ	$(^{38}Ar/^{37}37)_{Ca}$	±1σ	$(^{36}Ar/^{37}37)_{Ca}$	±1σ	$(^{36}Ar/^{38}38)_{Cl}$	Ca/K
X 01A	1.8	53.729	8.981	0.0194	0.0001	0.960	1.287	2.1846	0.3705	0.00589	0.00003	0.003306	0.000006	0.07	0.00	0.00	0.00	0.00	0.00	0.00	0.00	0.00	0.00	0.013638	0.000006	5.464E-10	28.201	FC-2	sanidine	0.00073	0.00009	0.01215	0.00003	0.000224	0.000016	0.000702	0.000012	0.000020	0.000001	0.000270	0.000000	263.00	2.32
X 01B	2	31.772	3.016	0.0162	0.0000	2.893	2.151	1.2841	0.1229	0.01675	0.00003	0.003277	0.000006	0.05	0.00	0.00	0.00	0.00	0.00	0.00	0.00	0.00	0.00	0.013638	0.000006	5.464E-10	28.201	FC-2	sanidine	0.00073	0.00009	0.01215	0.00003	0.000224	0.000016	0.000702	0.000012	0.000020	0.000001	0.000270	0.000000	263.00	2.32
X 01C	2.2	36.086	2.984	0.0147	0.0000	4.983	2.322	1.4601	0.1219	0.01590	0.00002	0.003272	0.000006	0.06	0.00	0.00	0.00	0.00	0.00	0.00	0.00	0.00	0.00	0.013638	0.000006	5.464E-10	28.201	FC-2	sanidine	0.00073	0.00009	0.01215	0.00003	0.000224	0.000016	0.000702	0.000012	0.000020	0.000001	0.000270	0.000000	263.00	2.32
X 01D	2.5	44.425	2.071	0.0131	0.0000	8.169	3.947	1.8017	0.0850	0.02191	0.00002	0.003217	0.000005	0.06	0.00	0.00	0.00	0.00	0.00	0.00	0.00	0.00	0.00	0.013638	0.000006	5.464E-10	28.201	FC-2	sanidine	0.00073	0.00009	0.01215	0.00003	0.000224	0.000016	0.000702	0.000012	0.000020	0.000001	0.000270	0.000000	263.00	2.32
X 01E	2.9	48.202	1.545	0.0113	0.0000	13.944	5.214	1.9569	0.0636	0.02664	0.00002	0.003175	0.000005	0.09	0.00	0.00	0.00	0.00	0.00	0.01	0.00	0.00	0.00	0.013638	0.000006	5.464E-10	28.201	FC-2	sanidine	0.00073	0.00009	0.01215	0.00003	0.000224	0.000016	0.000702	0.000012	0.000020	0.000001	0.000270	0.000000	263.00	2.32
01F	3.4	56.397	1.865	0.0099	0.0000	20.623	4.659	2.2947	0.0771	0.02030	0.00002	0.003193	0.000004	0.14	0.00	0.00	0.00	0.00	0.00	0.01	0.00	0.00	0.00	0.013638	0.000006	5.464E-10	28.201	FC-2	sanidine	0.00073	0.00009	0.01215	0.00003	0.000224	0.000016	0.000702	0.000012	0.000020	0.000001	0.000270	0.000000	263.00	2.32
01G	3.9	57.550	2.235	0.0088	0.0000	28.441	4.005	2.3424	0.0924	0.01710	0.00001	0.003215	0.000004	0.20	0.00	0.00	0.00	0.00	0.00	0.01	0.00	0.00	0.00	0.013638	0.000006	5.464E-10	28.201	FC-2	sanidine	0.00073	0.00009	0.01215	0.00003	0.000224	0.000016	0.000702	0.000012	0.000020	0.000001	0.000270	0.000000	263.00	2.32
01H	4.4	57.439	1.449	0.0085	0.0000	35.665	7.008	2.3378	0.0599	0.02998	0.00003	0.003115	0.000005	0.10	0.00	0.00	0.00	0.00	0.00	0.01	0.00	0.00	0.00	0.013638	0.000006	5.464E-10	28.201	FC-2	sanidine	0.00073	0.00009	0.01215	0.00003	0.000224	0.000016	0.000702	0.000012	0.000020	0.000001	0.000270	0.000000	263.00	2.32
01I	5.1	56.329	1.795	0.0084	0.0000	43.969	4.920	2.2920	0.0742	0.02147	0.00002	0.003185	0.000004	0.17	0.00	0.00	0.00	0.00	0.00	0.02	0.00	0.00	0.00	0.013638	0.000006	5.464E-10	28.201	FC-2	sanidine	0.00073	0.00009	0.01215	0.00003	0.000224	0.000016	0.000702	0.000012	0.000020	0.000001	0.000270	0.000000	263.00	2.32
01J	5.8	57.295	1.921	0.0085	0.0000	51.129	4.797	2.3318	0.0794	0.02057	0.00002	0.003189	0.000004	0.15	0.00	0.00	0.00	0.00	0.00	0.01	0.00	0.00	0.00	0.013638	0.000006	5.464E-10	28.201	FC-2	sanidine	0.00073	0.00009	0.01215	0.00003	0.000224	0.000016	0.000702	0.000012	0.000020	0.000001	0.000270	0.000000	263.00	2.32
01K	6.6	55.313	1.472	0.0087	0.0000	57.993	6.889	2.2500	0.0608	0.03062	0.00004	0.003119	0.000005	0.10	0.00	0.00	0.00	0.00	0.00	0.01	0.00	0.00	0.00	0.013638	0.000006	5.464E-10	28.201	FC-2	sanidine	0.00073	0.00009	0.01215	0.00003	0.000224	0.000016	0.000702	0.000012	0.000020	0.000001	0.000270	0.000000	263.00	2.32
01L	7.5	55.097	1.369	0.0090	0.0000	64.808	7.908	2.2411	0.0565	0.03529	0.00004	0.003085	0.000006	0.08	0.00	0.00	0.00	0.00	0.00	0.01	0.00	0.00	0.00	0.013638	0.000006	5.464E-10	28.201	FC-2	sanidine	0.00073	0.00009	0.01215	0.00003	0.000224	0.000016	0.000702	0.000012	0.000020	0.000001	0.000270	0.000000	263.00	2.32
01M	8.5	54.099	1.266	0.0086	0.0000	71.316	7.688	2.1999	0.0522	0.03495	0.00004	0.003092	0.000005	0.08	0.00	0.00	0.00	0.00	0.00	0.01	0.00	0.00	0.00	0.013638	0.000006	5.464E-10	28.201	FC-2	sanidine	0.00073	0.00009	0.01215	0.00003	0.000224	0.000016	0.000702	0.000012	0.000020	0.000001	0.000270	0.000000	263.00	2.32
01N	9.7	56.185	1.316	0.0074	0.0000	77.155	7.722	2.2860	0.0544	0.03378	0.00003	0.003091	0.000005	0.07	0.00	0.00	0.00	0.00	0.00	0.01	0.00	0.00	0.00	0.013638	0.000006	5.464E-10	28.201	FC-2	sanidine	0.00073	0.00009	0.01215	0.00003	0.000224	0.000016	0.000702	0.000012	0.000020	0.000001	0.000270	0.000000	263.00	2.32
01O	11	56.916	2.059	0.0055	0.0000	83.776	4.049	2.3162	0.0851	0.01748	0.00002	0.003214	0.000004	0.16	0.00	0.00	0.00	0.00	0.00	0.02	0.00	0.00	0.00	0.013638	0.000006	5.464E-10	28.201	FC-2	sanidine	0.00073	0.00009	0.01215	0.00003	0.000224	0.000016	0.000702	0.000012	0.000020	0.000001	0.000270	0.000000	263.00	2.32
01P	12.5	54.001	1.809	0.0041	0.0000	89.910	5.681	2.1958	0.0747	0.02587	0.00004	0.003159	0.000006	0.10	0.00	0.00	0.00	0.00	0.00	0.02	0.00	0.00	0.00	0.013638	0.000006	5.464E-10	28.201	FC-2	sanidine	0.00073	0.00009	0.01215	0.00003	0.000224	0.000016	0.000702	0.000012	0.000020	0.000001	0.000270	0.000000	263.00	2.32
01Q	15	56.569	2.445	0.0031	0.0000	96.036	4.193	2.3019	0.1010	0.01822	0.00004	0.003209	0.000005	0.14	0.00	0.00	0.00	0.00	0.00	0.03	0.00	0.00	0.00	0.013638	0.000006	5.464E-10	28.201	FC-2	sanidine	0.00073	0.00009	0.01215	0.00003	0.000224	0.000016	0.000702	0.000012	0.000020	0.000001	0.000270	0.000000	263.00	2.32
01R	25	54.741	2.410	0.0028	0.0000	100.000	4.450	2.2264	0.0995	0.01999	0.00004	0.003200	0.000006	0.09	0.00	0.00	0.00	0.00	0.00	0.02	0.00	0.00	0.00	0.013638	0.000006	5.464E-10	28.201	FC-2	sanidine	0.00073	0.00009	0.01215	0.00003	0.000224	0.000016	0.000702	0.000012	0.000020	0.000001	0.000270	0.000000	263.00	2.32

**Integrated K/Ca ±2σ**			**0.0064**	**0.0000**	**SD**
**Plateau Age ±2σ**	**55.820**	**0.930**	**n=13/18**	**MSWD=0.544**	
**Total Integrated Age ±2σ**	**54.355**	**0.937**			
**Isochron Age ±2σ**	**53.712**	**2.804**		**MSWD=0.616**	
**$(^{40}Ar/^{36}Ar)_{trapped}$ ±2σ**	**299.285**	**0.898**			

Notes:

[1]Corrected: Isotopic intensities corrected for blank, baseline, radioactive decay and detector intercalibration, not for interfering reactions.

X symbol preceding sample ID denotes analyses excluded from plateau age calculations.
Errors quoted for individual analyses include analytical error only, without interfering reaction or J uncertainties.
Integrated age calculated by summing isotopic measurements of all steps.
Plateau age error is inverse-variance-weighted mean error (Taylor, 1982) times root MSWD where MSWD>1.
Decay constants and isotopic abundances after Min et al. (2000)
Ages calculated relative to 28.201 Ma FC-2 Fish Canyon Tuff sanidine standard (Kuiper et al., 2008)

Supplementary Table 3: Whole rock major (in weight %) and trace element concentrations (in ppm).

Location			Murray Canyon		Agattu Forearc				Kresta Ridge					Medny Island					
Sample ID	Unit	Method	SO249 DR28-2	SO249 DR28-3#	SO249 DR51-2	SO249 DR51-6	SO249 DR51-7	SO249 DR51-9	SO249 DR40-1	SO249 DR40-2	SO249 DR40-2xen	SO249 DR40-4	SO249 DR40-11	K8-8	K8-9	K8-10	K8-11	K8-15	K8-18
Rock Type			lava	lava	lava	lava	lava	lava	plutonic	plutonic	volc xenolith	plutonic	lava	lava	lava	lava	lava	lava	lava
Lat °N		GPS	51.693	51.693	52.260	52.260	52.260	52.260	53.380	53.380	53.380	53.380	53.380	54.854	54.852	54.852	54.852	54.870	54.869
Long °E		GPS	176.781	176.781	172.970	172.970	172.970	172.970	171.218	171.218	171.218	171.218	171.218	167.446	167.446	167.445	167.445	167.444	167.445
SiO2	g/100g	XRF	51.13	50.53	49.80	48.23	48.76	48.13	71.08	70.83	51.2	48.20	52.30	49.01	49.31	49.7	70.79	49.27	53.38
Al2O3	g/100g	XRF	14.44	15.05	13.25	14.37	14.27	16.22	14.38	14.82	15.1	17.34	14.90	17.25	17.04	17.34	11.83	19.97	16.92
Fe2O3	g/100g	XRF	9.34	9.30	9.43	11.12	10.40	9.72	2.64	2.64	12.53	9.72	11.36	11.16	11.25	11.12	2.43	7.64	8.58
MgO	g/100g	XRF	7.41	5.42	7.77	7.49	7.72	6.67	0.72	0.7	4.28	7.04	4.58	6.80	6.64	6.66	0.35	6.46	6.14
MnO	g/100g	XRF	0.16	0.24	0.15	0.33	0.15	0.19	0.08	0.06	0.2	0.18	0.19	0.2	0.22	0.19	0.05	0.19	0.2
CaO	g/100g	XRF	8.15	4.74	9.11	11.59	11.18	12.46	2.25	1.88	6.64	7.78	5.49	10.85	10.72	10.74	5.01	10.14	8.74
Na2O	g/100g	XRF	4.39	6.09	4.71	2.93	3.16	2.82	4.76	5.44	6.11	2.62	6.68	2.22	2.34	2.3	5.07	2.76	3.13
K2O	g/100g	XRF	0.42	2.04	0.09	0.05	0.17	0.04	1.96	1.23	0.12	1.35	0.05	0.16	0.16	0.2	0.2	0.13	0.18
TiO2	g/100g	XRF	0.85	0.71	1.19	1.35	1.34	1.13	0.35	0.31	1.87	1.01	1.61	0.69	0.68	0.69	0.37	0.79	0.65
P2O5	g/100g	XRF	0.08	0.07	0.09	0.10	0.09	0.08	0.09	0.09	0.23	0.16	0.29	0.13	0.12	0.12	0.14	0.14	0.13
SO3	g/100g	XRF	0.00	0.01	0.23	0.09	0.03	0.04	0.00	0	0.32	0.01	0.14						
LOI	g/100g	XRF	3.1	4.78	3.56	1.58	1.93	1.57	1.02	1.14	2.22	4.40	2.32	1.28	1.02	0.99	2.67	2.01	1.60
SUM	g/100g	XRF	99.53	99.08	99.47	99.35	99.32	99.18	99.41	99.14	100.82	99.80	99.99	98.63	98.37	98.94	98.67	98.73	98.79
Li	µg/g	ICPMS	18.1	10.7	6.31	7.73	6.29	6.52	2.06	2.40	6.22	8.02	6.15	9.3	7.5	5.2	43.8	6.9	10.5
Sc	µg/g	ICPMS	42.9	40.9	43.8	47.1	46.6	42.3	6.12	7.81	38.2	28.6	31.6	38.2	38.6	42.5	8.5	46.9	38.2
V	µg/g	ICPMS	258	225	295	336	327	278	40	37	418	212	270	341	337	340	57	319	308
Cr	µg/g	ICPMS	21.1	32.1	150	173	173	286	4.7	1.5	13.0	212	33.6	62	63	53	2	13	47
Co	µg/g	ICPMS	43.6	53.8	43.9	46.3	44.8	40.9	3.5	3.3	30.8	32.3	28.0	37.8	38.4	37.2	0.3	37.1	31.8
Ni	µg/g	ICPMS	24.6	54.7	27.5	46.7	46.9	38.8	1.0	0.8	13.8	83.0	15.9	37	40	36	1	25	26
Cu	µg/g	ICPMS	17	123	88	81	82	91	2.53	2.13	121	47	59	150	152	186	307	164	208
Zn	µg/g	ICPMS	78.0	157	69.0	79.2	79.6	67.8	26.9	29.1	102	91.1	79.5	82.0	83.6	81.7	18.9	85.6	82.9
Ga	µg/g	ICPMS	14.9	11.2	13.0	19.8	16.7	17.1	13.7	15.0	19.4	15.9	18.5	17.4	18.1	18.5	8.8	19.9	18.5
Rb	µg/g	ICPMS	7.29	27.0	0.96	0.31	1.42	0.40	19.9	16.1	2.39	20.8	0.47	1.8	1.2	1.3	0.8	0.6	1.0
Sr	µg/g	ICPMS	180	67.7	146	136	134	125	185	239	121	236	57.89	387	369	408	138	452	373
Y	µg/g	ICPMS	20.9	22.1	26.0	29.8	30.2	25.8	15.7	20.7	32.1	19.8	33.0	16.2	14.1	15.9	32.9	17.6	17.7
Zr	µg/g	ICPMS	61.7	49.6	63.1	71.9	72.6	59.0	108	131	85.5	65.3	87.8	51	48	52	119	52	63
Nb	µg/g	ICPMS	0.59	0.55	1.19	1.20	1.21	0.89	3.59	3.88	1.31	3.25	1.43	0.9	0.8	0.9	1.9	0.9	1.1
Ba	µg/g	ICPMS	25.1	93.6	31.4	9.52	39.5	5.99	336	210	64.8	319	18.9	50	59	84	82	64	83
La	µg/g	ICPMS	2.36	3.26	2.22	2.29	2.40	1.90	11.0	11.2	3.95	4.26	5.25	5.5	5.2	5.4	8.3	4.6	5.8
Ce	µg/g	ICPMS	7.26	5.71	7.03	7.50	7.68	6.28	22.6	23.8	11.5	10.7	15.0	12.8	12.0	12.1	20.4	10.8	13.6
Pr	µg/g	ICPMS	1.28	1.48	1.27	1.39	1.41	1.17	2.89	3.16	2.09	1.69	2.59	2.1	1.9	1.9	3.1	1.8	2.2
Nd	µg/g	ICPMS	7.04	8.03	7.28	8.01	8.17	6.83	11.3	13.0	11.7	8.61	14.0	10.1	9.5	9.6	14.5	9.3	10.5
Sm	µg/g	ICPMS	2.41	2.69	2.72	3.02	3.08	2.61	2.36	2.77	3.88	2.59	4.45	2.73	2.56	2.64	3.75	2.62	2.78
Eu	µg/g	ICPMS	0.91	0.94	1.08	1.17	1.20	1.04	0.86	0.89	1.59	0.982	1.69	0.88	0.84	0.85	1.03	0.91	0.86
Gd	µg/g	ICPMS	3.10	3.55	3.69	4.17	4.21	3.57	2.38	3.02	5.13	3.11	5.38	2.86	2.69	2.85	4.12	2.82	3.00
Tb	µg/g	ICPMS	0.546	0.624	0.676	0.764	0.772	0.663	0.388	0.488	0.879	0.530	0.906	0.46	0.43	0.43	0.72	0.47	0.49
Dy	µg/g	ICPMS	3.61	4.13	4.59	5.19	5.25	4.48	2.44	3.19	5.74	3.46	5.87	2.88	2.67	2.77	5.03	2.98	3.07
Ho	µg/g	ICPMS	0.748	0.865	0.974	1.10	1.12	0.951	0.520	0.684	1.17	0.733	1.21	0.60	0.55	0.57	1.15	0.65	0.64
Er	µg/g	ICPMS	2.09	2.38	2.73	3.10	3.15	2.68	1.54	2.18	3.50	2.07	3.35	1.67	1.52	1.65	3.37	1.94	1.79
Tm	µg/g	ICPMS	0.309	0.344	0.407	0.464	0.467	0.400	0.251	0.360	0.507	0.303	0.494	0.25	0.23	0.24	0.51	0.29	0.27
Yb	µg/g	ICPMS	2.00	2.22	2.70	3.04	3.11	2.64	1.82	2.65	3.33	2.03	3.18	1.65	1.51	1.59	3.24	1.92	1.85
Lu	µg/g	ICPMS	0.300	0.335	0.411	0.458	0.475	0.403	0.304	0.423	0.502	0.309	0.490	0.25	0.23	0.25	0.49	0.28	0.28
Hf	µg/g	ICPMS	1.68	1.37	1.78	2.00	2.02	1.65	2.73	3.61	2.41	1.58	2.35	1.54	1.51	1.53	3.36	1.67	1.91
Ta	µg/g	ICPMS	0.047	0.048	0.092	0.095	0.096	0.071	0.357	0.506	0.093	0.202	0.108	0.07	0.06	0.05	0.14	0.05	0.08
Pb	µg/g	ICPMS	0.712	0.676	0.485	0.300*	0.333	0.251*	1.40	1.34	1.38	1.32	1.68	1.89	1.70	1.95	1.72	0.88	3.02
Th	µg/g	ICPMS	0.114	0.119	0.086	0.077	0.076	0.058	1.79	1.87	0.266	0.213	0.297	0.90	0.79	0.80	1.05	0.88	1.00
U	µg/g	ICPMS	0.151	0.141	0.286	0.026*	0.169	0.019*	0.488	0.725	0.159	0.114	0.202	0.33	0.28	0.29	0.45	0.34	0.48

Supplementary Table 4: Whole rock Sr-Nd-Pb isotope compositions.

Location			Murray Canyon			Agattu Forearc				Kresta Ridge				Medny Island					
Sample ID	Unit	Method	SO249 DR28-2	SO249 DR28-3	TN-182 -30-003°	SO249 DR51-2	SO249 DR51-6	SO249 DR51-7	SO249 DR51-9	SO249 DR40-1	SO249 DR40-2xen	SO249 DR40-4	SO249 DR40-11	K8-8	K8-9	K8-10	K8-11	K8-15	K8-18
Rock Type			lava	lava	lava	lava	lava	lava	lava	plutonic	volc xenolith	plutonic	lava	lava	lava	lava	lava	lava	lava
Lat °N		GPS	51.693	51.693		52.260	52.260	52.260	52.260	53.380	53.380	53.380	53.380	54.854	54.852	54.852	54.852	54.870	54.869
Long °E		GPS	176.781	176.781		172.970	172.970	172.970	172.970	171.218	171.218	171.218	171.218	167.446	167.446	167.445	167.445	167.444	167.445
87Sr/86Sr		TIMS	0.703721	0.705632	0.704197	0.703348	0.702710	0.702779	0.702533	0.703619	0.704255	0.703101	0.704970	0.703212	0.703215	0.703212	0.703387	0.703227	0.703252
2SE		TIMS	0.000005	0.000005	0.000005	0.000005	0.000005	0.000005	0.000005	0.000005	0.000005	0.000005	0.000005	0.000003	0.000005	0.000006	0.000003	0.000005	0.000003
87Sr/86Sr		TIMS	0.703823	0.705357		0.703422	0.702836	0.702876	0.702540	0.703548	0.704264	0.703222	0.705032						
2SE		TIMS	0.000004	0.000004		0.000005	0.000005	0.000005	0.000005	0.000005	0.000006	0.000005	0.000005						
87Sr/86Sr		TIMS	0.703721	0.705357	0.704197	0.703348	0.702711	0.702779	0.702533	0.703548	0.704264	0.703101	0.704970	0.703212	0.703215	0.703212	0.703387	0.703227	0.703252
2SE		TIMS	0.000005	0.000004	0.000005	0.000005	0.000005	0.000005	0.000005	0.000005	0.000006	0.000005	0.000005	0.000003	0.000005	0.000006	0.000003	0.000005	0.000003
143Nd/144Nd		TIMS	0.513192	0.513176	0.513206	0.513229	0.513239	0.513221	0.513228	0.513135	0.513152	0.513158	0.513169	0.513045	0.513039	0.513049	0.513117	0.513048	0.513049
2SE		TIMS	0.000005	0.000005	0.000005	0.000005	0.000005	0.000005	0.000005	0.000004	0.000005	0.000005	0.000004	0.000002	0.000005	0.000004	0.000002	0.000005	0.000003
εNd		TIMS	10.97	10.65	11.24	11.68	11.89	11.53	11.66	9.85	10.19	10.30	10.51	8.10	7.99	8.17	9.49	8.15	8.18
206Pb/204Pb		TIMS	18.2219	18.2447	18.3278	18.4634	18.1582	18.3631	18.1723	18.3621	18.3121	18.2727	18.3023	18.5935	18.5770	18.6136	18.5399	18.6486	18.7176
2SE		TIMS	0.0017	0.0008	0.0012	0.0009	0.0030	0.0016	0.0028	0.0023	0.0012	0.0008	0.0011	0.0011	0.0008	0.0013	0.0008	0.0007	0.0019
207Pb/204Pb		TIMS	15.4436	15.4550	15.4538	15.4440	15.4316	15.4406	15.4283	15.4734	15.4720	15.4713	15.4688	15.5362	15.5337	15.5342	15.5158	15.5424	15.5542
2SE		TIMS	0.0016	0.0008	0.0011	0.0009	0.0025	0.0016	0.0022	0.0020	0.0013	0.0008	0.0010	0.0010	0.0008	0.0011	0.0007	0.0006	0.0021
208Pb/204Pb		TIMS	37.5530	37.6154	37.5861	37.5356	37.4823	37.4807	37.4393	37.8542	37.7970	37.8156	37.7993	38.1954	38.1719	38.1742	38.0641	38.2169	38.2773
2SE		TIMS	0.0042	0.0025	0.0029	0.0029	0.0062	0.0047	0.0057	0.0050	0.0039	0.0027	0.0027	0.0028	0.0023	0.0026	0.0020	0.0015	0.0072
Age	Ma		50	50	50	50	50	50	50	50	50	50	50	50	50	50	50	50	50
87Rb/86Sr			0.117	1.156	0.042	0.019	0.007	0.031	0.009	0.311	0.057	0.255	0.023	0.014	0.009	0.009	0.016	0.004	0.008
87Sr/86Sr (t)			0.70364	0.70454	0.70319	0.70333	0.70271	0.70276	0.70253	0.70333	0.70422	0.70292	0.70495	0.70320	0.70321	0.70321	0.70338	0.70322	0.70325
147Sm/144Nd			0.206	0.201	0.152	0.225	0.227	0.227	0.230	0.125	0.199	0.181	0.191	0.162	0.162	0.165	0.156	0.169	0.160
143Nd/144Nd (t)			0.51312	0.51311	0.51312	0.51316	0.51316	0.51315	0.51315	0.51309	0.51309	0.51310	0.51311	0.51299	0.51299	0.51300	0.51307	0.51299	0.51300
εNd			10.97	10.65	10.56	11.68	11.89	11.53	11.66	9.85	10.19	10.30	10.51	8.10	7.99	8.17	9.49	8.15	8.18
εNd(t)			10.90	10.62	10.84	11.50	11.69	11.34	11.45	10.30	10.17	10.40	10.54	8.32	8.20	8.37	9.75	8.32	8.41
2SE (εNd)			0.09	0.10	0.10	0.10	0.09	0.09	0.09	0.09	0.10	0.10	0.08	0.04	0.11	0.08	0.04	0.09	0.05
238U/204Pb			13.25	13.03	33.09	36.91	5.32*	31.64	4.67*	21.93	7.23	5.45	7.53	11.02	10.50	9.60	16.47	24.32	10.07
235U/204Pb			0.10	0.09	0.24	0.27	0.04*	0.23	0.03*	0.16	0.05	0.04	0.05	0.08	0.08	0.07	0.12	0.18	0.07
232Th/204Pb			10.32	11.41	64.56	11.50	16.47*	14.76	14.95*	82.88	12.49	10.47	11.43	31.21	30.57	27.03	39.72	65.45	21.71
232Th/238U			0.78	0.88	1.95	0.31	3.09*	0.47	3.2*	3.78	1.73	1.92	1.52	2.83	2.91	2.82	2.41	2.69	2.16
206Pb/204Pb (t)			18.12	18.14	18.00	18.18	18.12	18.12	18.14	18.19	18.26	18.23	18.24	18.51	18.50	18.54	18.41	18.46	18.64
207Pb/204Pb (t)			15.44	15.45	15.44	15.43	15.43	15.43	15.43	15.47	15.47	15.47	15.47	15.53	15.53	15.53	15.51	15.53	15.55
208Pb/204Pb (t)			37.53	37.59	37.44	37.51	37.44	37.44	37.40	37.65	37.77	37.79	37.77	38.12	38.10	38.11	37.97	38.05	38.22

#Major elements from Bezard et al. (5)

°Rb, Sr, Sm, Nd, U, Th and Pb concentrations from Jicha et al (4).

*Due to U uptake and possible Pb mobilization during seafloor alteration, parent/daughter ratios for samples with mu>37 were calculated assuming U=Nb/47 and Pb=Ce/25 after Hofmann et al., 1986.

Supplementary Table 5: Trace element concentrations (in ppm) for all reference materials and blanks processed with the samples.

	Li	Sc	V	Cr	Co	Ni	Cu	Zn	Ga	Rb	Sr	Y	Zr	Nb	Sn	Sb	Cs	Ba	La	Ce	Pr	Nd	Sm
**BHVO-2 normal digestion**																							
38790	4.85	32.8	324.0	292.2	45.6	121.0	135.0	100.3	22.0	9.3	399.8	26.1	170.0	18.6	1.76	0.13	0.11	131.0	15.8	38.4	5.46	25.4	6.31
39420	4.63	32.2	320.3	292.5	45.7	117.0	129.2	102.3	21.5	9.2	390.7	26.0	168.0	17.9	1.69	0.12	0.11	127.0	15.6	38.1	5.42	25.2	6.28
41565	4.66	31.9	324.4	287.8	44.3	120.3	131.1	176.1	21.7	9.1	382.7	25.9	169.8	17.8	1.73	0.13	0.11	129.6	15.6	37.5	5.34	25.0	6.21
41670	4.72	32.0	328.2	290.0	44.5	120.8	132.2	104.4	21.8	9.2	389.8	26.1	171.1	17.9	1.73	0.15	0.11	131.5	15.8	37.9	5.40	25.3	6.30
**BIR-1 normal digestion**																							
39419	3.37	41.0	310.2	370.0	51.2	164.1	113.5	67.4	15.0	0.2	104.8	15.3	14.8	0.5	0.66	0.46	0.01	6.3	0.6	1.9	0.38	2.5	1.14
41564	3.36	44.1	331.6	385.4	52.0	167.6	120.2	70.1	15.8	0.2	105.6	15.8	14.8	0.5	0.76	0.62	0.01	6.4	0.6	1.9	0.37	2.4	1.13
41669	3.36	44.2	327.6	379.9	51.2	164.6	118.9	67.9	15.7	0.2	105.7	15.7	14.7	0.5	0.67	0.53	0.01	6.3	0.6	1.9	0.37	2.4	1.12
**BIR-1 bomb digestion**																							
39144	3.28	42.5	309.2	376.7	50.7	165.1	119.6	69.0	15.4	0.2	106.4	15.4	14.6	0.5	0.67	0.51	0.01	6.2	0.6	1.9	0.37	2.4	1.12
**BHVO-2 LA-ICP-MS on nano-particulate pressed powder tablets**																							
	4.07	33	289	273	40.9	103	117	99.1	20.4	8.4	401	26.9	178	17.5	2.16	0.14	0.086	132.5	15.5	36.7	5.28	25.1	6.20
	4.05	33	296	270	40.8	102	113	92.5	19.5	8.4	395	25.7	174	17.4	2.14	0.13	0.088	132.6	15.1	37.2	5.22	24.5	6.11
	4.44	33	306	287	44.1	111	128	101.7	22.4	9.9	400	27.1	178	17.5	2.35	-	0.098	133.9	15.4	37.3	5.20	25.3	6.20
	4.47	33	313	287	44.0	113	129	99.5	22.2	9.6	404	26.4	177	17.6	2.20	-	0.098	137.2	15.1	38.4	5.32	25.0	6.24
	4.69	33	327	305	45.9	118	134	107.7	23.4	10.1	400	26.4	176	17.9	2.41	-	0.100	135.8	15.3	38.4	5.26	24.8	5.80
	4.41	33	325	294	46.1	115	131	104.6	22.7	9.9	399	26.5	176	18.1	2.14	-	0.103	134.7	15.2	38.4	5.29	24.5	6.12
	4.47	33	313	285	44.3	113	127	99.0	21.7	9.6	398	26.1	177	17.7	2.50	-	0.106	134.9	15.0	37.9	5.30	25.0	6.26
	4.48	33	318	288	44.6	112	129	101.8	22.4	9.8	401	26.1	177	17.8	2.53	-	0.100	137.5	15.0	38.9	5.35	25.4	6.56
	4.38	34	306	289	43.0	109	123	95.3	21.3	9.4	394	25.6	176	17.9	2.28	-	0.100	133.0	14.8	36.6	5.22	24.7	6.06
	4.32	34	311	292	43.1	108	125	94.6	21.8	9.5	400	26.4	181	18.2	2.40	-	0.109	133.5	14.9	37.2	5.32	24.9	6.24
**KL2-G LA-ICP-MS on nano-particulate pressed powder tablets**																							
	5.75	34	300	271	42.5	98	86	106.2	21.0	9.1	371	27.7	167	15.3	1.61	0.11	0.130	126.8	13.8	32.9	4.70	22.8	5.92
	5.60	32	307	271	41.5	99	85	105.7	21.4	8.9	359	25.3	154	14.7	1.58	0.11	0.130	125.9	12.9	33.0	4.60	21.7	5.39
	5.59	32	302	274	42.4	101	89	108.2	21.4	8.8	350	25.1	154	14.2	1.89	0.10	0.121	121.0	12.6	31.9	4.55	21.6	5.39
	5.76	32	305	277	42.4	102	88	107.5	21.2	8.8	353	25.0	154	14.2	1.85	0.11	0.114	121.0	12.5	32.1	4.57	21.3	5.54
**Blanks normal digestion**																							
38788	0.058	0.014	0.013	0.142	0.007	0.071	0.208	0.754	0.006	0.005	3.992	0.022	0.078	0.000	0.060	0.001	0.001	0.107	0.029	0.046	0.005	0.016	0.003
38789	0.026	0.014	0.007	0.107	0.005	0.055	0.197	0.282	0.003	0.002	1.571	0.009	0.026	0.000	0.029	0.001	0.000	0.039	0.012	0.019	0.002	0.006	0.001
39417	0.003	0.002	0.010	0.111	0.002	0.012	0.016	0.028	0.000	0.000	0.021	0.000	0.002	0.001	0.006	0.000	0.000	0.002	0.000	0.000	0.000	0.000	0.000
39418	0.104	0.006	0.018	0.164	0.003	0.018	0.026	0.473	0.014	0.008	2.388	0.013	0.053	0.001	0.032	0.001	0.002	0.055	0.017	0.028	0.003	0.009	0.002
41562	0.046	0.047	0.016	1.318	0.021	0.039	0.069	0.407	0.008	0.007	0.388	0.004	0.033	0.003	0.019	0.007	0.002	0.040	0.005	0.008	0.002	0.010	0.009
41563	0.046	0.048	0.022	1.340	0.019	0.055	0.096	1.081	0.007	0.010	0.078	0.002	0.014	0.003	0.015	0.007	0.002	0.089	0.002	0.003	0.001	0.008	0.009
**Blanks Paar-bomb digestion**																							
39143	0.014	0.007	0.017	0.214	0.007	0.039	0.225	0.280	0.002	0.001	0.007	0.000	0.008	0.002	0.022	0.002	0.000	0.009	0.001	0.002	0.000	0.001	0.001

Supplementary Table 5 continued: Trace element concentrations in ppm for all reference materials and blanks processed with the samples.

	Eu	Gd	Tb	Dy	Ho	Er	Tm	Yb	Lu	Hf	Ta	Pb	Th	U
**BHVO-2 normal digestion**														
38790	2.15	6.34	0.96	5.42	1.00	2.48	0.33	2.03	0.29	4.42	1.15	1.60	1.21	0.41
39420	2.11	6.25	0.95	5.40	0.99	2.46	0.33	2.01	0.29	4.26	1.11	1.63	1.19	0.40
41565	2.10	6.26	0.94	5.38	0.98	2.45	0.33	2.02	0.29	4.26	1.13	1.64	1.20	0.42
41670	2.11	6.29	0.95	5.39	0.98	2.47	0.33	2.01	0.29	4.27	1.13	1.90	1.19	0.41
**BIR-1 normal digestion**														
39419	0.54	1.84	0.37	2.65	0.59	1.68	0.25	1.68	0.26	0.57	0.04	3.10	0.03	0.01
41564	0.54	1.81	0.36	2.61	0.58	1.65	0.25	1.66	0.26	0.56	0.04	3.48	0.03	0.01
41669	0.53	1.81	0.36	2.60	0.57	1.65	0.25	1.65	0.25	0.56	0.04	3.07	0.03	0.01
**BIR-1 bomb digestion**														
39144	0.53	1.79	0.36	2.59	0.57	1.63	0.25	1.64	0.25	0.56	0.04	2.91	0.03	0.01
**BHVO-2 LA-ICP-MS on nano-particulate pressed powder tablets**														
	2.11	6.41	0.96	5.46	0.99	2.73	0.34	2.15	0.30	4.52	1.17	1.61	1.22	0.42
	2.00	6.25	0.94	5.32	0.95	2.56	0.35	1.96	0.28	4.40	1.15	1.59	1.16	0.39
	2.06	6.51	0.98	5.44	0.98	2.64	0.35	2.10	0.29	4.51	1.18	1.68	1.18	0.40
	2.06	6.30	0.94	5.31	0.99	2.58	0.34	2.08	0.31	4.44	1.15	1.65	1.18	0.42
	2.07	6.34	0.96	5.32	1.00	2.55	0.34	2.17	0.29	4.47	1.16	1.91	1.18	0.44
	2.04	6.51	0.94	5.31	0.96	2.54	0.34	2.03	0.29	4.38	1.13	1.57	1.15	0.41
	2.16	6.37	0.96	5.59	0.98	2.69	0.36	2.13	0.29	4.69	1.16	1.71	1.19	0.43
	2.20	6.46	0.97	5.58	1.01	2.66	0.35	2.15	0.29	4.60	1.16	1.70	1.22	0.44
	2.05	6.33	0.96	5.55	0.96	2.50	0.33	2.07	0.29	4.52	1.12	1.58	1.17	0.39
	2.17	6.50	0.95	5.51	1.00	2.56	0.33	2.14	0.30	4.46	1.12	1.56	1.17	0.39
**KL2-G LA-ICP-MS on nano-particulate pressed powder tablets**														
	2.04	6.40	0.95	5.40	1.04	2.68	0.35	2.17	0.30	4.27	1.01	1.85	1.04	0.56
	1.87	5.92	0.87	5.09	0.95	2.50	0.33	2.05	0.29	3.87	0.95	1.82	0.97	0.55
	1.94	5.94	0.90	5.16	0.93	2.47	0.33	2.01	0.28	3.87	0.89	1.86	0.95	0.55
	1.95	5.88	0.91	5.27	0.95	2.58	0.34	2.12	0.28	4.03	0.91	1.88	0.93	0.56
**Blanks normal digestion**														
38788	0.001	0.003	0.000	0.003	0.001	0.002	0.000	0.002	0.000	0.002	0.010	0.030	0.003	0.003
38789	0.000	0.001	0.000	0.001	0.000	0.001	0.000	0.001	0.000	0.001	0.002	0.016	0.001	0.001
39417	0.000	0.000	0.000	0.000	0.000	0.000	0.000	0.000	0.000	0.000	0.001	0.002	0.000	0.000
39418	0.000	0.002	0.000	0.002	0.000	0.001	0.000	0.001	0.000	0.001	0.035	0.014	0.004	0.002
41562	0.003	0.009	0.001	0.005	0.001	0.004	0.001	0.007	0.002	0.006	0.009	0.013	0.003	0.003
41563	0.003	0.008	0.001	0.006	0.001	0.004	0.001	0.006	0.001	0.006	0.003	0.014	0.002	0.002
**Blanks Paar-bomb digestion**														
39143	0.000	0.001	0.000	0.000	0.000	0.000	0.000	0.000	0.000	0.001	0.001	0.012	0.000	0.000
